# One-Pot Synthesis and Anticancer Activity Evaluation of Indolo[1,2-*a*]quinoline-7-carbonitrile Derivatives

**DOI:** 10.3390/molecules31203731

**Published:** 2026-10-17

**Authors:** Kredmanee Yimnoi, Norasian Boosaman, Nutthawat Chuanopparat, Kedchin Jearawuttanakul, Sawinee Seemakhan, Tanawadee Khumpanied, Warawuth Wannalo, Suparerk Borwornpinyo, Paiboon Ngernmeesri

**Affiliations:** 1 Department of Chemistry and Center of Excellence for Innovation in Chemistry, Faculty of Science, Kasetsart University, Bangkok 10900, Thailand; kredmanee.yi@ku.th (K.Y.); norasian.boos@ku.th (N.B.); fscinwc@ku.ac.th (N.C.); 2 Excellent Center for Drug Discovery, Faculty of Science, Mahidol University, Bangkok 10400, Thailand; 3 Department of Biotechnology, Faculty of Science, Mahidol University, Bangkok 10400, Thailand

**Keywords:** indolo[1,2-*a*]quinoline, cascade reaction, one-pot synthesis, metal-free synthesis, small-molecule anticancer agents, colorectal cancer, breast cancer, molecular docking

## Abstract

Indolo[1,2-*a*]quinoline derivatives represent an attractive heterocyclic scaffold in anticancer drug discovery. Herein, fifty-four novel indolo[1,2-*a*]quinoline-7-carbonitrile analogs were synthesized via a transition-metal-free cascade reaction involving nucleophilic aromatic substitution and Knoevenagel condensation, affording yields up to 87%. All compounds were evaluated for their antiproliferative activities against human colorectal (HCT-116 and HT-29) and breast (MCF-7) cancer cell lines using the MTT assay. Eleven analogs exhibited high potency with IC_50_ values ranging from 2.16 to 8.18 µM. Notably, 2-bromoindolo[1,2-*a*]quinoline-7-carbonitrile displayed broad-spectrum cytotoxicity across HCT-116, HT-29, and MCF-7 lines (IC_50_ = 3.64 ± 0.72, 5.29 ± 1.83, and 6.06 ± 0.16 µM, respectively). To explore potential binding modes and rationalize structural features, molecular docking simulations were conducted against CDK2, CDK6, and EGFR kinase models. The in silico results revealed strong predicted binding affinities (−7.73 to −9.49 kcal/mol), driven predominantly by C7-cyano hydrogen bonding and hydrophobic interactions, providing hypothetical insights into how these scaffolds might interact with ATP-binding pockets. These findings demonstrate that the indolo[1,2-*a*]quinoline-7-carbonitrile framework holds significant promise as a pharmacophore for further mechanistic and drug development studies.

## 1. Introduction

**Figure 1:**
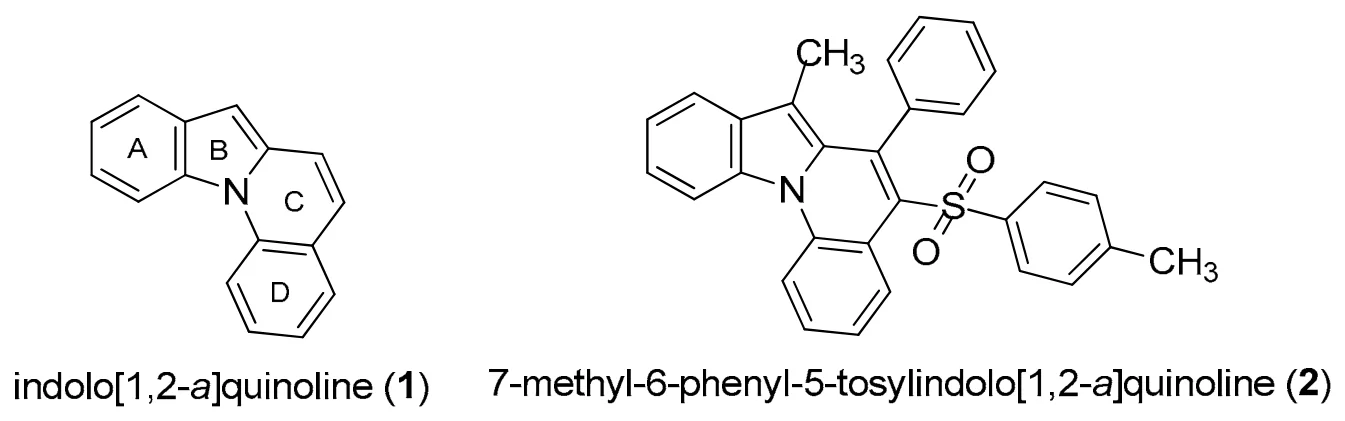
Structures of indolo[1,2-*a*]quinoline (**1**) and the potent EGFR tyrosine kinase inhibitor 7-methyl-6-tosylindolo[1,2-*a*]quinoline (**2**).

Nitrogen-containing heterocyclic compounds represent a fundamentally important class of scaffolds in both modern organic synthesis and medicinal chemistry [1,2,3]. Among these molecular systems, fused quinoline derivatives, particularly indole derivatives such as indolo[1,2-*a*]quinoline (**1**) (Figure 1), have attracted the attention of organic chemists for several decades because of their wide spectrum of pharmacological and biological properties [4]. This unique, rigid, and highly planar tetracyclic framework, especially represented by indolo[1,2-*a*]quinoline (**1**), serves as a key building block for advanced functional materials. For instance, indolo[1,2-*a*]quinoline derivatives have been successfully utilized as organic semiconductors [5,6], luminescent materials for organic light-emitting devices [7,8,9], and organic sensitizers for dye-sensitized solar cells [10]. Moreover, their robust conjugation and aggregation-induced emission characteristics make them ideal candidates for bioimaging applications in live cells [11]. Recently, non-coplanar diarylphosphinoyl indolo[1,2-*a*]quinoline derivatives have also been developed to further expand the stereochemical diversity and optoelectronic applications of this core [12].

In medicinal chemistry, indolo[1,2-*a*]quinolines and closely related compounds have demonstrated remarkable biological activities, including antimalarial [13], antimicrobial [14,15,16,17], anti-inflammatory [17,18,19], antiviral [16] and anticancer properties [16,20,21]. A key breakthrough in recent years has identified sulfonylated and trifluoromethylthiolated indolo[1,2-*a*]quinolines as highly effective inhibitors of epidermal growth factor receptor tyrosine kinase (EGFR-TK) [22]. The leading candidate (**2**) exhibited outstanding EGFR-TK inhibitory activity with an IC_50_ value of 0.28 ± 0.05 µM. This compound showed cytotoxic potency against the EGFR-overexpressing human lung adenocarcinoma (A549) cell line comparable to afatinib, a tyrosine kinase inhibitor drug, while maintaining remarkably low toxicity toward normal Vero cells. These findings highlight the immense clinical potential of the indolo[1,2-*a*]quinoline core as a privileged scaffold for targeted cancer therapies.

Given their structural complexity and valuable applications, the construction of the indolo[1,2-*a*]quinoline skeleton has been a major objective for synthetic chemists. Since the first reported synthesis in 1983 via flash vacuum pyrolysis of 2-benzylpyridine *N*-oxides at temperatures up to 1300 °C [23], numerous methodologies have been developed [4]. Over the past two decades, transition-metal-catalyzed cyclizations have emerged as the dominant tools to construct the tetracyclic skeleton. Among these, palladium-catalyzed reactions have been widely utilized [24,25,26,27,28,29,30,31,32]. Lautens and co-workers pioneered several Pd-catalyzed direct arylation and tandem cyclizations, including the modified Catellani *ortho*-alkylation of bromoalkyl indoles [30], a Pd-catalyzed annulation of haloaryl heterocycles with strained alkenes to construct polycyclic and pyrroloquinoline frameworks [31], and a water-promoted Suzuki-Miyaura tandem coupling of dibromoolefins [32]. Gold catalysis has also proven highly efficient for hydroamination and cycloisomerization reactions. For instance, Shibata and co-workers demonstrated that nitrogen-tethered 1,8-diynes could undergo divergent gold-catalyzed cyclization, where a Johnphos-coordinated gold catalyst selectively promoted a 6-endo-dig cycloisomerization to yield indolo[1,2-*a*]quinolines [33]. Furthermore, Brambilla and co-workers reported a robust gold-catalyzed terminal alkyne oxidation of 1-(2-ethynylphenyl)-1*H*-indoles followed by acid-catalyzed intramolecular cyclization [34]. Other transition metals, including Rh(III) using aldehydes as traceless directing groups [35], cheap Ni(II)/Zn catalytic systems [36], and Mn [37], have also been successfully employed.

**Scheme 1:**
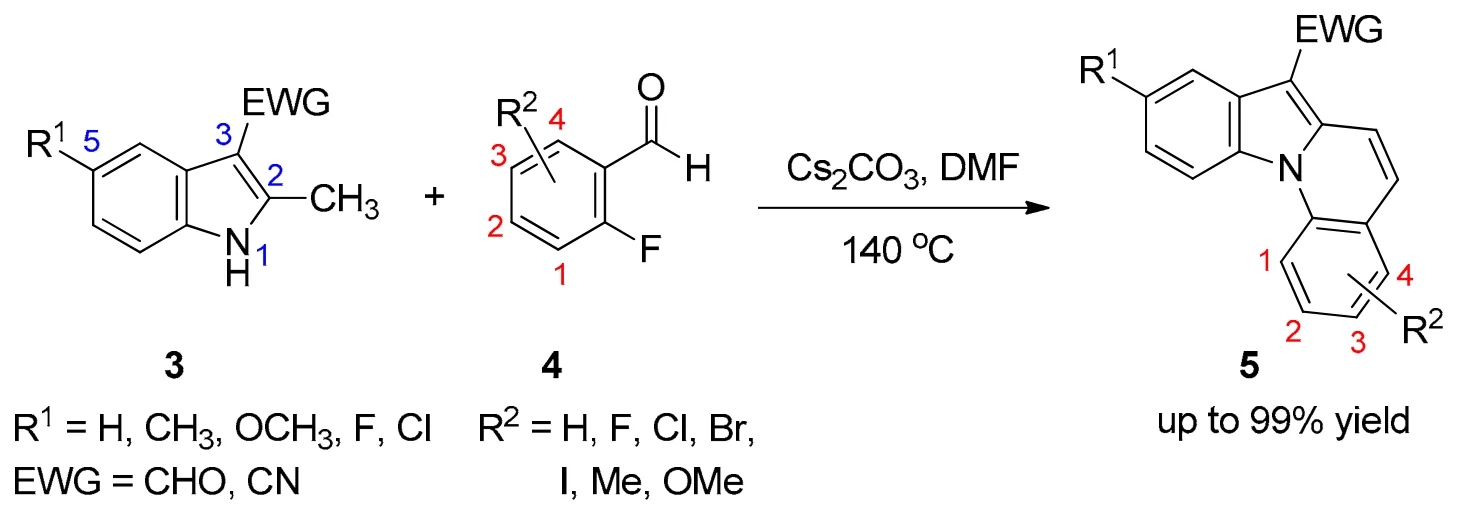
Synthesis of indolo[1,2-*a*]quinoline derivatives previously reported by our research group.

We have recently reported the transition-metal-free, one-pot synthesis of indolo[1,2-*a*]quinoline derivatives via a tandem nucleophilic aromatic substitution and Knoevenagel condensation between indoles **3** and 2-fluorobenzaldehydes **4** (Scheme 1) [38]. Performed using Cs_2_CO_3_ as the base in DMF at 140 °C, the reaction afforded most of the desired products in moderate to excellent yields (up to 99%). Notably, an electron-withdrawing group (EWG) at C3 of the indole substrate, specifically a formyl (–CHO) or cyano (–CN) group, was essential to enhance the acidity of the C2-methyl group, with formyl-substituted indoloquinolines constituting the majority of the synthesized compounds.

**Figure 2:**
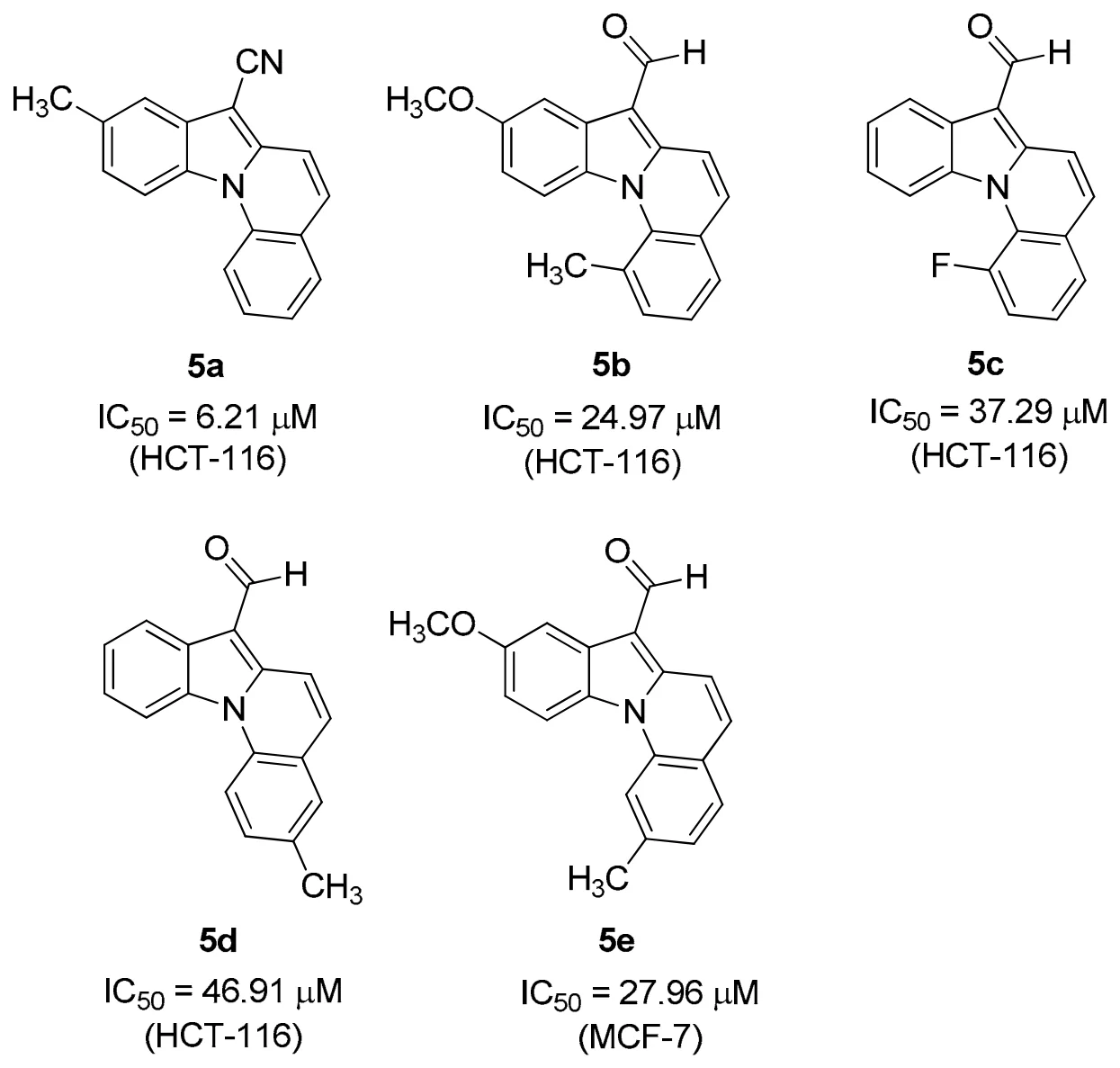
Synthesis of indolo[1,2-*a*]quinoline derivatives previously reported by our research group.

Evaluation of all synthesized derivatives against colorectal cancer (HCT-116) and breast cancer (MCF-7 and MDA-MB-231) cell lines revealed that compound **5a** exhibited the highest potency against HCT-116 cells, with an IC_50_ value of 6.21 µM, which was comparable to that of doxorubicin (IC_50_ = 6.65 µM) used as a positive control (Figure 2) [20]. Compounds **5b**–**5d** also inhibited HCT-116 cell proliferation, albeit with lower activity (IC_50_ = 24.97, 37.29, and 46.91 µM, respectively). Additionally, compound **5d** displayed moderate anticancer activity against MCF-7 cells (IC_50_ = 27.96 µM). Prompted by these findings, we sought to further investigate the anticancer properties of indolo[1,2-*a*]quinoline derivatives bearing a cyano group as the electron-withdrawing group, structurally analogous to compound **5a**.

## 2. Results and Discussion

### 2.1. Chemical Synthesis

**Scheme 2:**
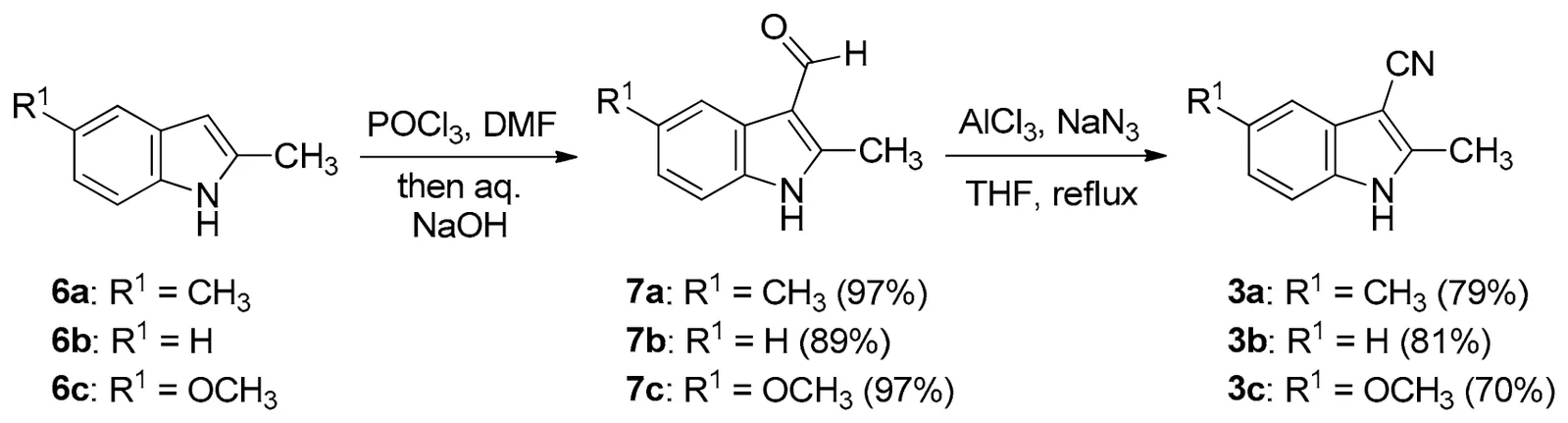
Preparation of indole-3-carbonitriles **3a**, **3b** and **3c**.

Initially, 2,5-dimethylindole-3-carbonitrile (**3a**), 2-methylindole-3-carbonitrile (**3b**), and 5-methoxy-2-methylindole-3-carbonitrile (**3c**) were prepared following a reported protocol (Scheme 2). The synthetic sequence commenced with a Vilsmeier–Haack reaction to install a formyl group at C3 [39], affording indole-3-carboxaldehydes **7a**–**7c** in 97%, 89%, and 97% yields, respectively. Subsequent transformation of the formyl moiety into a cyano group using AlCl_3_ and NaN_3_ [40] provided the corresponding carbonitriles **3a**, **3b**, and **3c** in 79%, 81%, and 70% yields, respectively.

Although the reaction of indoles **3a**, **3b**, and **3c** with 2-fluorobenzaldehyde (**4a**) afforded 9-methylindolo[1,2-*a*]quinoline-7-carbonitrile (**5a**), indolo[1,2-*a*]quinoline-7-carbonitrile (**5ba**) and 9-methoxyindolo[1,2-*a*]quinoline-7-carbonitrile (**5ca**), in good to excellent yield (98%, 78%, and 98%, respectively) under the initial conditions as shown in Scheme 1 [38], other 2-fluorobenzaldehyde derivatives proved less effective. As a precaution, we evaluated lower reaction temperatures using **3b** and **4a**. Reducing the temperature from 140 °C to 130 °C maintained a comparable yield (78% vs. 80%), whereas a further reduction to 120 °C led to a slight yield decrease (74%). Consequently, 130 °C was selected as the optimal temperature to minimize product degradation while maintaining high chemical yield.

With the optimized reaction conditions in hand (1.0 equiv of indole substrate, 1.6 equiv of 2-fluorobenzaldehyde derivative **4b**–**4u**, and 3.0 equiv of Cs_2_CO_3_ in DMF at 130 °C), we investigated the substrate scope to prepare various indolo[1,2-*a*]quinoline-7-carbonitrile derivatives (Table 1). Benzaldehydes possessing both electron-withdrawing groups (F, Cl, Br, and I) and electron-donating groups (Me and OMe) at diverse positions on the benzene ring were employed. Reaction of 2,5-dimethylindole-3-carbonitrile (**3a**) with benzaldehydes **4b**–**4u** afforded target products **5ab**–**5au** in 10–83% yields (Table 1, entries 1–20), though benzaldehyde **4d** (entry 3) failed to give the desired product. Similarly, 2-methylindole-3-carbonitrile (**3b**) yielded indoloquinolines **5bb**–**5bu** in 16–76% yields (entries 21–40), with the exception of benzaldehyde **4j** (entry 29). Furthermore, treatment of 5-methoxy-2-methylindole-3-carbonitrile (**3c**) provided derivatives **5cb**–**5cu** in 11–87% yields (entries 41–60), whereas benzaldehydes **4j**, **4l**, **4n**, and **4p** (entries 49, 51, 53, and 55) were unsuccessful.

It is worth noting that when the crude residue obtained after workup and solvent evaporation was a solid, the target compounds could be efficiently purified by simple recrystallization from a mixture of hexane and dichloromethane. For instance, derivative **5au** was isolated in a high yield of 83% via recrystallization (entry 20). This operational simplicity offers a significant practical advantage over conventional column chromatography, making it highly suitable for large-scale production. Conversely, when the crude products were obtained as oils, attempts at purification by recrystallization were unsuccessful, necessitating flash column chromatography.

Theoretically, the presence of an electron-withdrawing group at the *ortho* or *para* position relative to the fluorine leaving group is expected to facilitate nucleophilic aromatic substitution reaction and enhance product yields, whereas an electron-donating group typically exhibits the opposite effect. However, across the investigated substrate scope, no clear or consistent correlation between electronic effects and product yields was observed. While benzaldehydes bearing substituents adjacent to the formyl group (–CHO) tended to give relatively better yields in several instances, steric influences alone do not fully account for the observed variations. For substrates that failed to afford the target products (entries 3, 29, 49, 51, 53, and 55) or provided low-to-moderate yields, the primary limiting factors were attributed to competitive thermal decomposition and the formation of complex, unidentifiable side-product mixtures that could not be isolated despite repeated purification efforts. These observations highlight the inherent sensitivity of certain functionalized substrates to the basic environment under elevated temperature conditions (130 °C), defining the current operational scope of this cascade methodology.

### 2.2. Anticancer Evaluation

**Figure 3:**
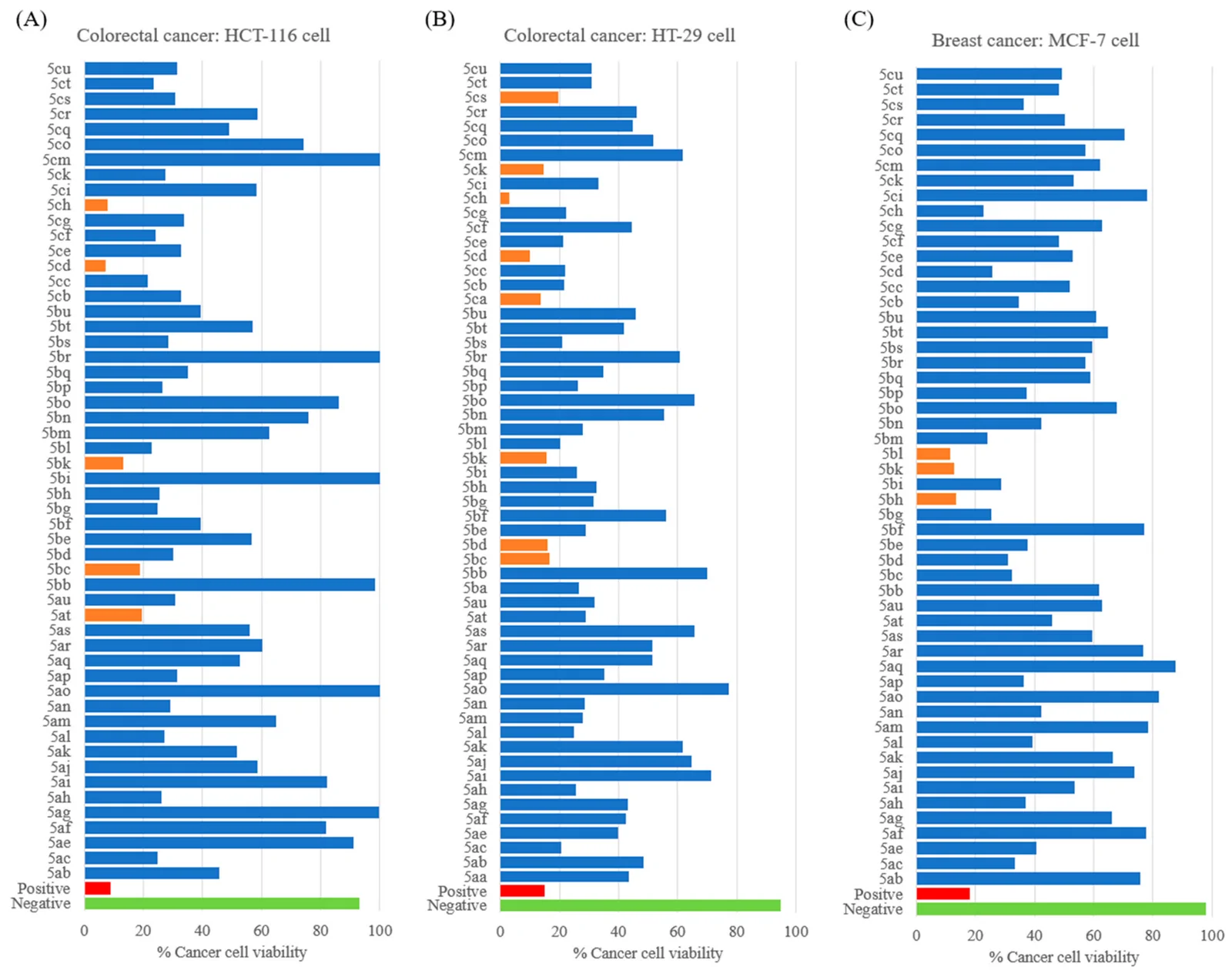
Primary screening data of indolo[1,2-*a*]quinoline derivatives and doxorubicin (positive control) for cytotoxic activity against HCT-116 (**A**), HT-29 (**B**), and MCF-7 (**C**) cancer cells (negative control: DMSO). The Z′-factor values for each assay were 0.865, 0.809, and 0.880, respectively, indicating excellent assay quality and robustness for high-throughput screening (Z′ ≥ 0.5).

Indolo[1,2-*a*]quinoline-7-carbonitrile derivatives (**5ab−5au**, **5bb−5bu**, and **5cb−5cu**) were evaluated for their in vitro cytotoxic activities against three human cancer cell lines: colorectal cancer (HCT-116 and HT-29) and breast cancer (MCF-7). Additionally, compounds **5a**, **5ba**, and **5ca**, which had not been previously evaluated against HT-29 cells, were included in this screen to provide a comprehensive cytotoxicity profile. During the primary screening at a single concentration of 10 µM, several compounds demonstrated remarkable antiproliferative potential, displaying cell viability percentages comparable to or even exceeding those of the reference drug, doxorubicin (Figure 3). Applying a cell viability threshold of ≤20% (corresponding to ≥80% growth inhibition at 10 µM) prioritized specific hit compounds for subsequent half-maximal inhibitory concentration (IC_50_) determination (Table 2).

Quantitative evaluation revealed promising potent active leads across all cell lines. Against HCT-116 cells, compound **5ch** emerged as the most potent derivative, displaying an impressive IC_50_ value of 2.16 ± 1.37 µM (with other hits **5at**, **5bc**, **5bk**, and **5cd** yielding IC_50_ values ranging from 3.51 to 4.95 µM). For HT-29 cells, **5cs** exhibited the highest cytotoxicity with an IC_50_ of 2.27 ± 1.21 µM, closely followed by **5ch** (IC_50_ = 2.40 ± 1.03 µM), while the remaining selected hits (**5bc**, **5bd**, **5bk**, **5ca**, **5cd**, and **5ck**) demonstrated IC_50_ values between 3.93 and 8.18 µM. For breast cancer (MCF-7), compound **5bl** showed the most superior activity with an IC_50_ of 3.84 µM (SD < 0.01 µM), whereas **5bh** and **5bk** displayed IC_50_ values of 5.68 ± 1.62 and 6.06 ± 0.16 µM, respectively. Notably, compound **5bk** demonstrated broad-spectrum cytotoxicity across all three lines. It should be noted that the current study focused primarily on evaluating the preliminary cytotoxic efficacy (IC_50_) of the novel derivatives against cancer cell lines to identify potential lead compounds. Further investigation regarding their cytotoxicity on non-cancerous/normal cell lines and determination of the selectivity index (SI) will be warranted in future lead optimization studies.

### 2.3. Structure-Activity Relationship (SAR) Analysis

Systematic analysis of the primary screening data and half-maximal inhibitory concentration (IC_50_) values revealed clear structure–activity relationships (SAR) governed by the functional groups at C-7 and C-9, as well as the substitution pattern on the outer fused aromatic ring (R^2^). A crucial structural feature for potent anticancer activity is the presence of the C-7 cyano group (-CN). Comparison between C-7 nitrile derivatives, such as **5a** (IC_50_ = 6.21 µM against HCT-116), and their formyl counterparts (**5b**–**5e**, IC_50_ = 24.97–46.91 µM) demonstrated that replacement of the cyano group with a formyl group (–CHO) resulted in a dramatic 4- to 7-fold loss of cytotoxicity. This highlights the pivotal role of the compact, linear geometry and hydrogen-bond-accepting character of the nitrile moiety, which likely allows optimal spatial fit within the binding site compared to the sterically bulkier formyl group.

Regarding modifications at the C-9 position (R^1^), derivatives bearing a hydrogen atom (R^1^ = H, **5b** series) or an electron-donating methoxy group (R^1^ = OMe, **5c** series) generally displayed superior broad-spectrum cytotoxicity compared to their methyl-substituted counterparts (R^1^ = Me, **5a** series). Primary screening data across all three cell lines demonstrated that the overall average cell viability progressively decreased from 52.63% for the **5a** series (R^1^ = Me) to 42.50% for the **5b** series (R^1^ = H) and 38.95% for the **5c** series (R^1^ = OMe), confirming that an electron-donating methoxy group or hydrogen atom at C-9 enhances antiproliferative efficacy. Remarkably, applying the cell viability threshold of ≤20% identified only 1 hit compound from the **5a** series (**5at**), whereas the **5b** and **5c** series yielded 5 hit compounds each (**5bc**, **5bd**, **5bh**, **5bk**, **5bl** and **5ca**, **5cd**, **5ch**, **5ck**, **5cs**, respectively) for subsequent IC_50_ determination.

**Figure 4:**
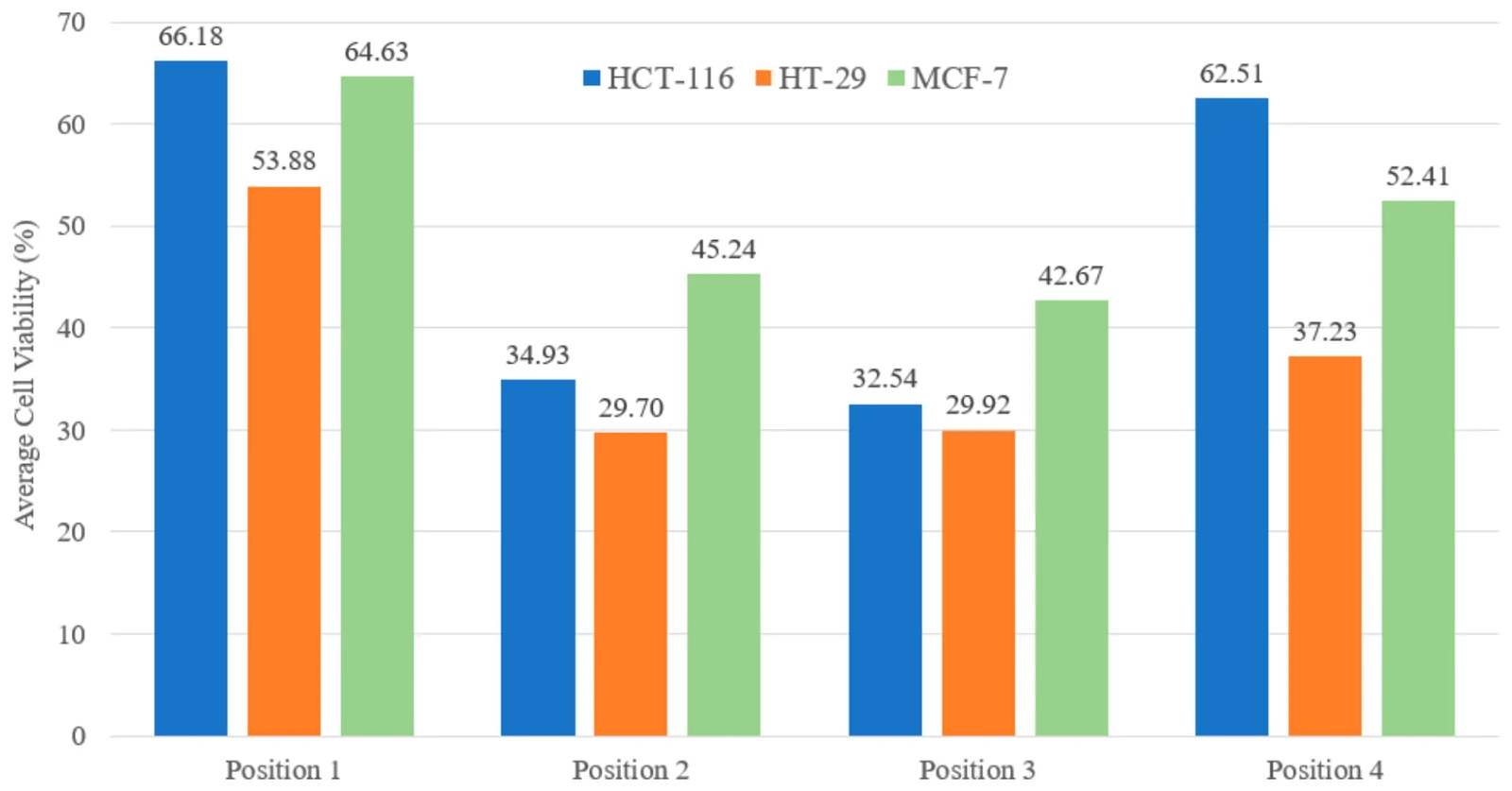
Average cell viability (%) of HCT-116, HT-29, and MCF-7 cancer cell lines according to the substitution position on the outer D-ring.

The position and nature of substituents on the outer aromatic D-ring further dictated cytotoxicity in a position-dependent manner. As shown in Figure 4, primary screening averages revealed that substitution at the C3 and C2 positions conferred the highest antiproliferative potency, exhibiting average cell viabilities of 32.54% and 34.93% in HCT-116, 29.92% and 29.70% in HT-29, and 42.67% and 45.24% in MCF-7 cells, respectively. In contrast, substitution at C4 resulted in moderate potency (cell viability: 62.51% in HCT-116, 37.23% in HT-29, and 52.41% in MCF-7), while substitution at C1 produced the lowest cytotoxicity (cell viability: 66.18% in HCT-116, 53.88% in HT-29, and 64.63% in MCF-7), indicating steric disfavor at the C1 position. Notably, halogenation (Cl, F, Br) at C2 or C3 generally conferred superior antiproliferative activity, yielding top-performing hits such as **5ch** (3-Cl), **5cd** (3-F), and **5bk** (2-Br). Nevertheless, alkoxy substitutions at these positions also maintained favorable potency, such as **5cs** (2-OMe) and **5at** (3-OMe), highlighting the overall acceptability of hydrogen-bond-accepting or electron-donating features at C2 or C3.

### 2.4. Docking Study

**Figure 5:**
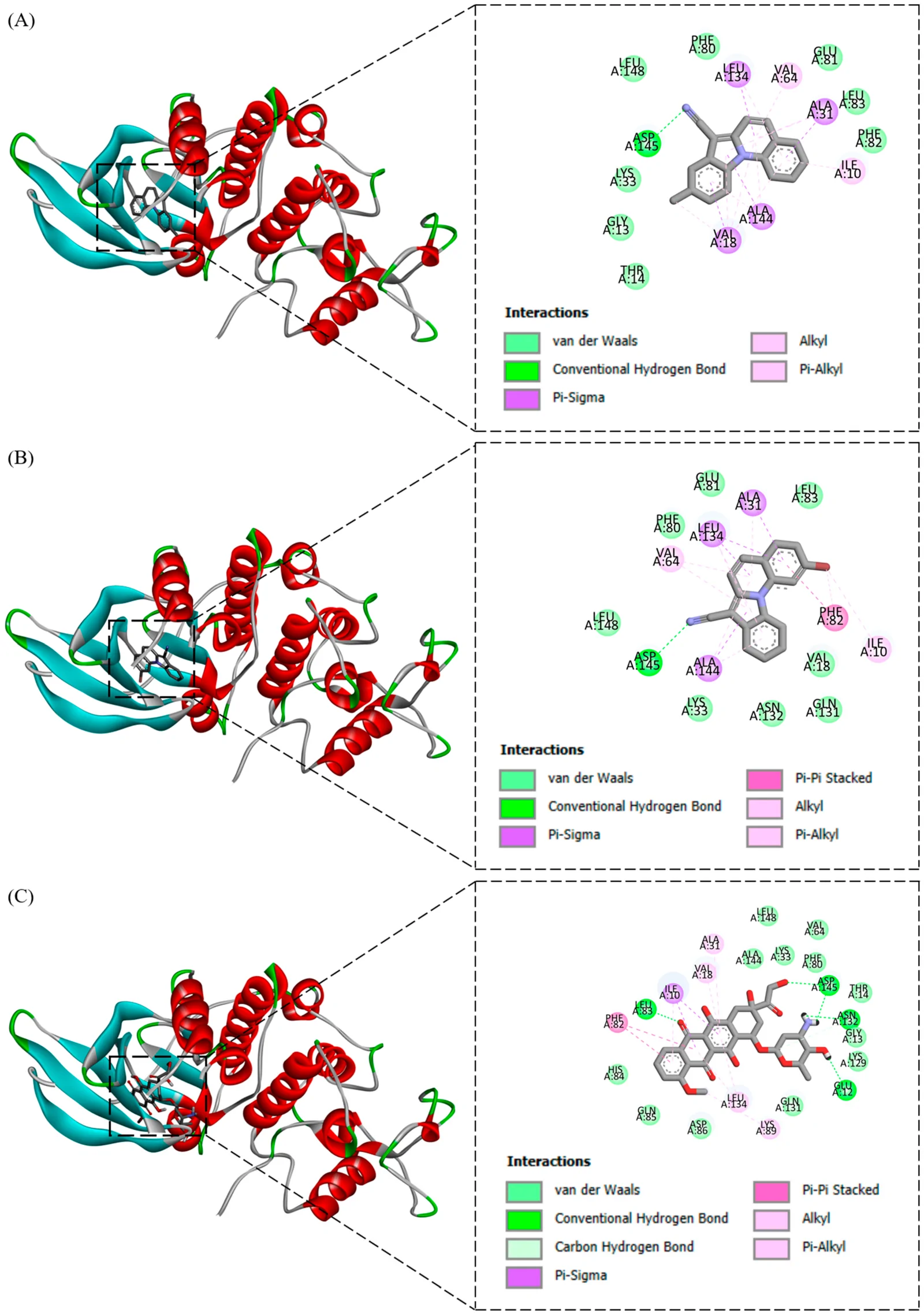
Predicted 3D binding interactions of compounds **5a** (**A**), **5bk** (**B**), and doxorubicin (**C**) with CDK2 (PDB ID: 1DI8), showing 3D docking poses (**left**) and 2D ligand-residue interaction diagrams (**right**).

To investigate the binding interactions and potential binding modes of the indolo[1,2-*a*]quinoline-7-carbonitrile derivatives against representative kinase models relevant to human colorectal (HCT-116 and HT-29) and breast (MCF-7) cancer cell lines, molecular docking simulations were performed using AutoDock 4.2.6, followed by binding-energy estimation and interaction analysis using BIOVIA Discovery Studio Visualizer. The rigid, planar heterocyclic scaffold of these compounds is expected to favor accommodation within the hydrophobic region of the ATP-binding pocket of protein kinases. Cyclin-dependent kinase 2 (CDK2) is a key regulator of the G1/S phase transition and contributes to cell-cycle progression and DNA replication through the phosphorylation of multiple regulatory substrates. Dysregulation of CDK2 has been associated with tumor progression in colorectal cancer, while pharmacological inhibition of CDK2 can induce G1/S cell-cycle arrest and apoptosis in colorectal cancer cells such as HCT-116 [41]. Accordingly, the crystal structure of CDK2 in complex with a planar 4-anilinoquinazoline inhibitor (PDB ID: 1DI8) was selected as a representative receptor model [42,43]. This structure provides a well-defined ATP-binding pocket in which the co-crystallized 4-anilinoquinazoline inhibitor occupies the kinase active site, with its quinazoline ring system positioned along the peptide strand and its anilino substituent extending into a hydrophobic pocket. To validate the docking protocol, the native co-crystallized ligand (DTQ) was re-docked into the active site of CDK2, yielding an RMSD value of 1.43 Å (Table 3), which is well within the acceptable threshold (<2.0 Å) and confirms the reliability of the grid parameters. Using this validated protocol, preliminary docking of compound **5a** revealed a strong predicted binding affinity, with a calculated binding energy of −8.67 kcal/mol. Specifically, the cyano group at the C7 position forms a hydrogen bond with the ASP:145 residue, whereas the rigid aromatic core establishes π–σ interactions with ALA:144, LEU:134, VAL:18, and VAL:64, together with additional alkyl interactions involving ILE:10 and ALA:31 (Figure 5A).

Similar interaction patterns were observed for compounds **5at**, **5bc**, **5bk**, **5cd**, and **5ch** (Table 3). These derivatives exhibited strong predicted binding affinities, with docking scores ranging from −8.06 to −8.79 kcal/mol (−8.58, −8.06, −8.79, −8.33, and −8.39 kcal/mol, respectively). Hydrogen bonding of the cyano group and hydrophobic interactions of the core aromatic framework served as the primary predicted driving forces for binding. Figure 5B illustrates the predicted binding mode of the most promising candidate, compound **5bk**, within the CDK2 active site model. In addition to a hydrogen bond with ASP:145, **5bk** established halogen and hydrophobic interactions between its bromine atom and adjacent residues (PHE:82 and ILE:10). Its rigid aromatic core further engaged in π–σ interactions with VAL:64, LEU:134, and ALA:144, while additional alkyl/π–alkyl interactions were observed with ILE:10 and ALA:31.

For comparative purposes, doxorubicin, which served as the positive control in the in vitro cytotoxicity assays, was also docked into the CDK2 active site. Although doxorubicin primarily exerts its cytotoxicity via DNA intercalation and topoisomerase II inhibition rather than direct kinase targeting [44], it exhibited a calculated binding energy of −8.83 kcal/mol with CDK2. This interaction was stabilized by four conventional hydrogen bonds with LEU:83, ASP:145, ASN:132, and GLU:12, along with hydrophobic contacts involving LYS:89, LEU:134, PHE:82, ILE:10, VAL:18, and ALA:31. The binding interaction network of doxorubicin with CDK2 is illustrated in Figure 5C.

**Figure 6:**
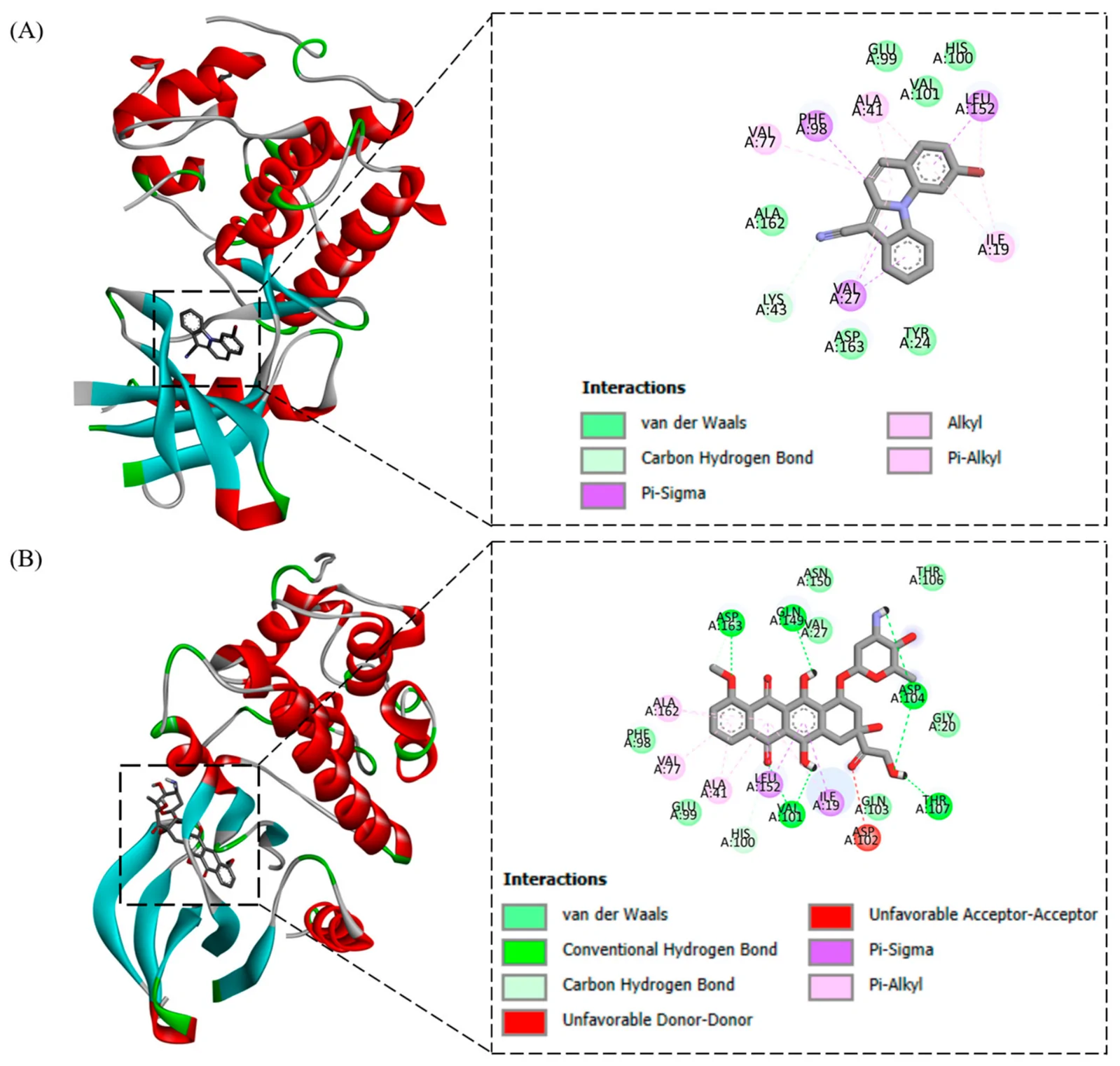
Predicted 3D binding interactions of compound **5bk** (**A**) and doxorubicin (**B**) with CDK6 (PDB ID: 5L2I), showing 3D docking poses (**left**) and 2D ligand-residue interaction diagrams (**right**).

For HT-29 colorectal cancer cells, cyclin-dependent kinases 4 and 6 (CDK4/6) form active complexes with D-type cyclins to regulate early G1-to-S phase progression through the phosphorylation of the retinoblastoma (Rb) protein. Dysregulation of the Cyclin D–CDK4/6–Rb pathway promotes hyperphosphorylation of Rb and uncontrolled cell proliferation in colorectal cancer (CRC). Pharmacological or molecular inhibition of CDK4/6 maintains Rb in a hypophosphorylated state, leading to G1-phase cell-cycle arrest and marked suppression of CRC cell viability, including HT-29 [45]. Consequently, the crystal structure of CDK6 in complex with the kinase inhibitor palbociclib (PDB ID: 5L2I) was selected as a representative receptor model [46]. This structure provides a structurally defined ATP-binding pocket capable of accommodating planar heterocyclic scaffolds through interactions with residues lining the binding site. To validate the docking protocol, the native co-crystallized ligand (palbociclib, LQQ) was re-docked into the active site of CDK6, yielding an RMSD value of 0.83 Å (Table 4), which is well within the acceptable threshold (<2.0 Å) and confirms the reliability of the grid parameters. Following this validated protocol, the active derivatives displayed favorable predicted binding affinities, with calculated binding energies ranging from −7.73 to −9.49 kcal/mol. Among these, compounds **5bk**, **5ck**, **5cs**, and **5ch** exhibited particularly strong binding scores (−9.49, −8.56, −8.48, and −8.04 kcal/mol, respectively). Most active derivatives formed key hydrogen bonds with residues within the binding site, such as VAL:101, HIS:100, HIS:101, and ASP:163. Notably, compound **5bk** exhibited the highest predicted binding affinity (−9.49 kcal/mol) and formed a hydrogen bond with LYS:43, along with hydrophobic interactions involving VAL:27, ILE:19, LEU:152, ALA:41, VAL:77, and PHE:98 (Figure 6A). Meanwhile, docking of the reference drug doxorubicin against CDK6 yielded a calculated binding energy of −10.96 kcal/mol. Doxorubicin formed six conventional hydrogen bonds with ASP:163, GLN:149, ASP:104, THR:107, VAL:101, and HIS:100, as well as hydrophobic interactions with ALA:162, VAL:77, ALA:41, LEU:152, and ILE:19. Detailed predicted binding modes are depicted in Figure 6B.

**Figure 7:**
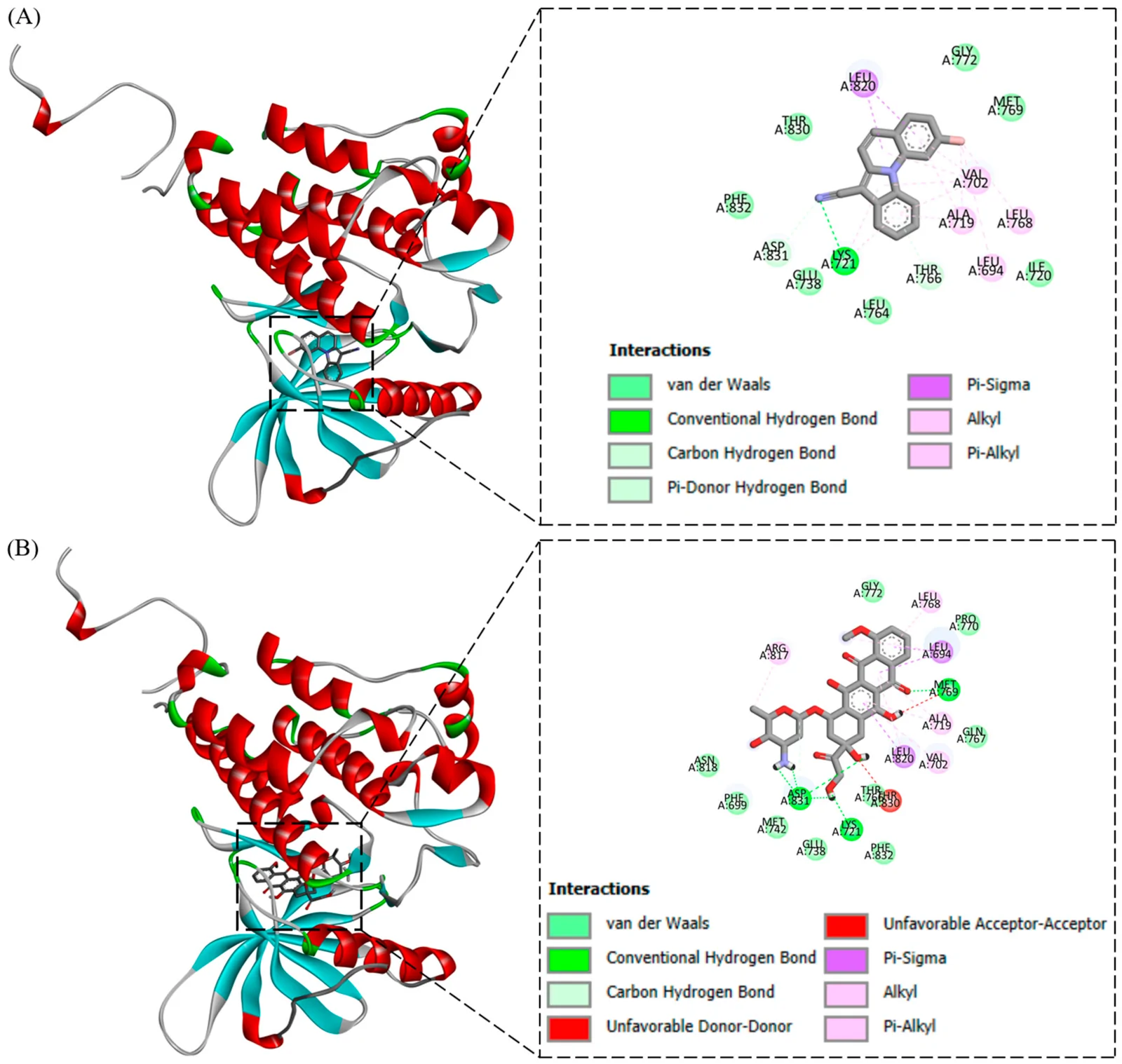
Predicted 3D binding interactions of compound **5bk** (**A**) and doxorubicin (**B**) with EGER kinase (PDB: 1M17), showing 3D docking poses (**left**) and 2D ligand-residue interaction diagrams (**right**).

For MCF-7 breast cancer cells, epidermal growth factor receptor (EGFR) kinase was selected as a prospective model target because EGFR signaling regulates cellular proliferation and survival through downstream pathways, including the Ras/MAPK and PI3K/Akt pathways. Dysregulated EGFR signaling contributes to breast cancer progression and may promote tumor growth and resistance to endocrine therapy, whereas inhibition of EGFR kinase activity can suppress downstream signaling and promote cell-cycle arrest and apoptosis [47]. The crystal structure of the EGFR kinase domain in complex with the quinazoline-based inhibitor gefitinib (PDB ID: 1M17) was selected as a representative receptor model [48]. This structure contains a well-defined ATP-binding pocket in which planar heterocyclic inhibitors can be accommodated through hydrophobic interactions and contacts with residues in the hinge region and adjacent binding-site regions. To validate the docking protocol, the native co-crystallized ligand (erlotinib, AQ4) was re-docked into the active site of EGFR, yielding an RMSD value of 1.64 Å (Table 5), which successfully validates the docking parameters (<2.0 Å). Following this validated setup, derivatives **5bh**, **5bk**, and **5bl** displayed favorable calculated binding energies of −8.40, −8.02, and −8.61 kcal/mol, respectively. Among these, compound **5bl**, which showed the highest cytotoxicity against MCF-7 cells (IC_50_ = 3.84 µM, SD < 0.01 µM), also exhibited the most favorable predicted binding energy (−8.61 kcal/mol). The derivatives were stabilized within the active site through a combination of hydrogen-bonding, hydrophobic, electrostatic, and sulfur interactions involving residues lining the ATP-binding pocket. Specifically, detailed analysis of derivative **5bk** revealed three predicted hydrogen bonds involving ASP:831, LYS:721, and THR:766 along with hydrophobic interactions with LEU:820, VAL:702, ALA:719, LEU:768, LEU:694, and LYS:721 (Figure 7A). Similarly, molecular docking of doxorubicin into the active site of EGFR kinase yielded a calculated binding energy of −11.69 kcal/mol. The interaction profile revealed three conventional hydrogen bonds involving ASP:831, LYS:721, and MET:769, as well as hydrophobic interactions with LEU:820, VAL:702, ALA:719, LEU:694, LEU:768, and ARG:817. Figure 7B presents the corresponding 3D predicted binding mode and 2D interaction diagram of doxorubicin within the EGFR active site model.

### 2.5. Drug-Likeness Analysis and ADME Prediction

Oral administration of anticancer agents offers significant clinical advantages over intravenous chemotherapy, including enhanced patient compliance, reduced hospitalization costs, and improved quality of life. Therefore, evaluating the drug-likeness and absorption, distribution, metabolism, and excretion (ADME) profiles of the active compounds using the SwissADME web tool (http://swissadme.ch/ (accessed on 30 September 2026)) provides critical insights into their potential as orally bioavailable chemotherapeutic leads. Lipinski’s rule of five (RO5) was applied as a primary benchmark to assess oral absorption potential, evaluating molecular weight (≤500 g/mol), consensus Log P (≤5), hydrogen bond donors (≤5), and hydrogen bond acceptors (≤10). As summarized in Table 6, all evaluated compounds strictly complied with Lipinski’s rule of five without a single violation. Their molecular weights ranged from 260.27 to 351.20 g/mol, consensus Log P values were between 3.31 and 4.04, hydrogen bond acceptors ranged from 1 to 3, and hydrogen bond donors were 0 across all candidates. Additionally, the compounds exhibited 0 to 2 rotatable bonds and topological polar surface area (TPSA) values between 28.20 and 46.66 Å^2^, indicating high structural rigidity paired with optimal conformational flexibility, leading to a uniform moderate bioavailability score of 0.55.

**Figure 8:**
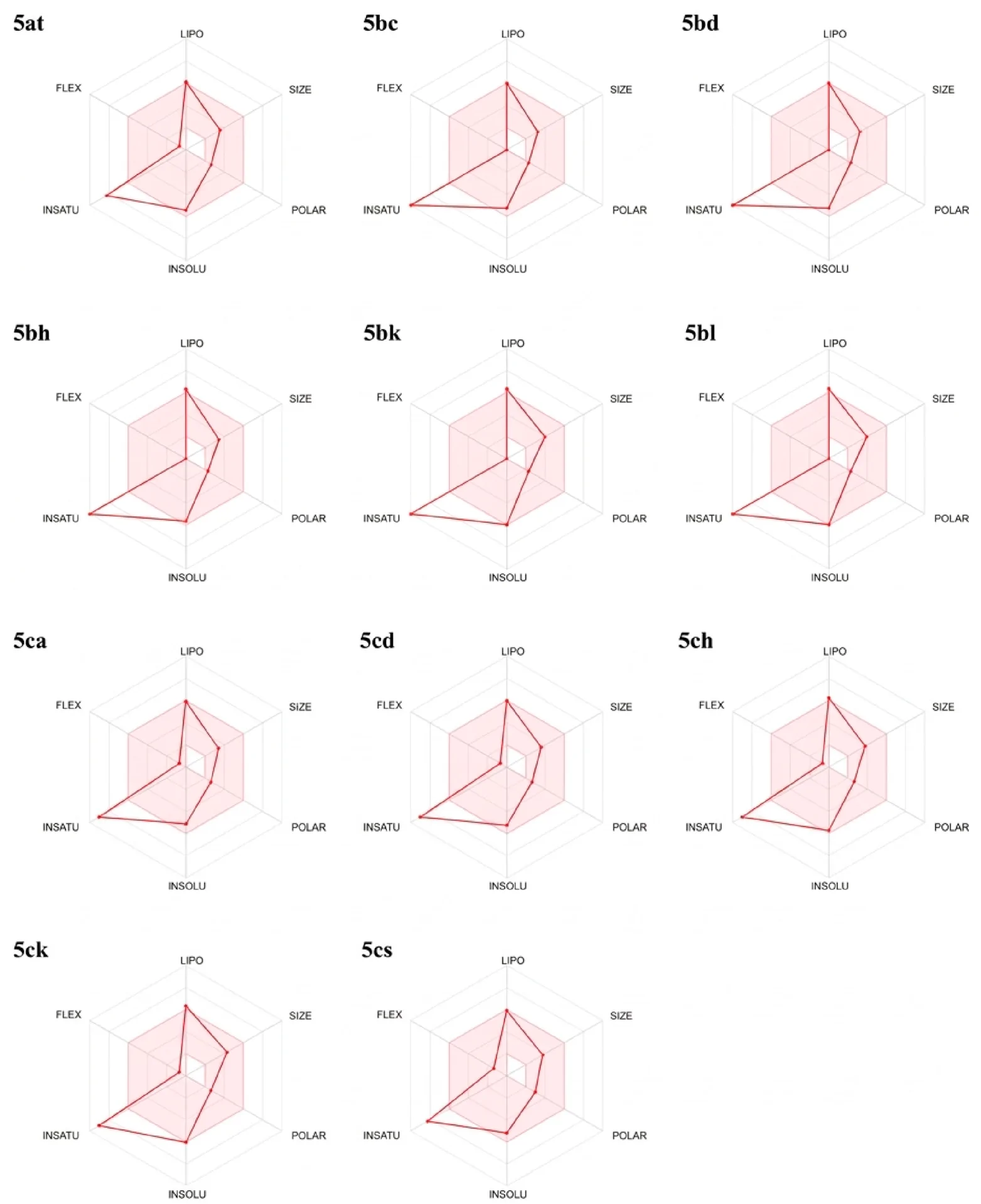
Bioavailability radar charts of the synthesized candidates (**5at**, **5bc**, **5bd**, **5bh**, **5bk**, **5bl**, **5ca**, **5cd**, **5ch**, **5ck**, and **5cs**) illustrating the oral drug-likeness parameters.

The predicted ADME profiles provided deeper insights into their absorption, distribution, metabolism, and excretion behavior. All compounds demonstrated high predicted gastrointestinal (GI) absorption without acting as P-glycoprotein (P-gp) substrates, suggesting efficient intestinal uptake and minimal risk of efflux-mediated resistance. Furthermore, none of the molecules triggered Pan-Assay Interference Compounds (PAINS) alerts, confirming their specificity as true active hits without non-specific reactivity. However, regarding central nervous system distribution, all targeted compounds were predicted to be blood–brain barrier (BBB) permeants, suggesting potential brain tissue penetration. The oral drug-likeness features were visually evaluated using bioavailability radar charts (Figure 8). All candidates fall well within the optimal pink-shaded boundaries across most oral drug-likeness parameters, including lipophilicity, size, polarity, solubility, and flexibility. However, a deviation was observed in the unsaturation parameter (INSATU), where the values fell outside the pink area due to the highly aromatic nature inherent to these conjugated frameworks. Despite this exception, the overall in silico ADME predictions demonstrate that the synthesized series possesses highly favorable pharmacokinetic profiles and oral drug-likeness, supporting their potential as promising lead candidates for further medicinal chemistry optimization against breast and colon cancers.

Beyond basic physicochemical parameters, the predicted ADME and pharmacokinetic profiles further highlighted the drug-like properties of these derivatives. All compounds were predicted to possess high gastrointestinal (GI) absorption without being identified as P-glycoprotein (P-gp) substrates, suggesting efficient intestinal uptake and a low likelihood of efflux-mediated drug resistance. Furthermore, no Pan-Assay Interference Compounds (PAINS) alerts were detected, confirming that the observed bioactivities stem from specific target interactions rather than non-specific assay interference. Regarding central nervous system distribution, all candidates were predicted to be blood–brain barrier (BBB) permeants, indicating potential for brain tissue penetration. Visual inspection of the oral drug-likeness via bioavailability radar charts (Figure 8) showed that most parameters, including lipophilicity, molecular size, polarity, solubility, and flexibility, fell well within the optimal pink-shaded area. Although a deviation was observed in the unsaturation parameter (INSATU) due to the highly aromatic and conjugated nature of the scaffold, the overall in silico findings suggest that the synthesized series possesses highly favorable pharmacokinetic profiles and drug-likeness, strongly supporting their further development as promising oral chemotherapeutic leads against colorectal and breast cancers.

## 3. Materials and Methods

### 3.1. General Information

^1^H NMR spectra were recorded at 400 MHz, and ^13^C NMR spectra were recorded at 100 MHz, using a Bruker 400 MHz spectrometer (Bruker Corporation, Ettlingen, Germany). ^19^F NMR spectra were recorded at 470 MHz using a Bruker 500 MHz spectrometer (Bruker Corporation, Ettlingen, Germany). Chemical shifts (*δ*) are reported in parts per million relative to the solvent residue signal of CDCl_3_ (δ 7.26 ppm) or DMSO-*d*_6_ (δ 2.50 ppm) for ^1^H NMR spectra, relative to the center line of CDCl_3_ (δ 77.16 ppm) or DMSO-*d*_6_ (δ 39.52 ppm) for ^13^C NMR spectra, and relative to the center line of fluorobenzene (δ -113.10 ppm) as an internal standard for ^19^F NMR spectra. Multiplicities are given as the following abbreviations: s (singlet), d (doublet), t (triplet), q (quartet), m (multiplet), br (broad), and combinations thereof. Coupling constants (*J*) are reported in hertz (Hz). High-resolution mass spectra (HRMS) were recorded on a quadrupole-time-of-flight (QTOF) micro-TOPQIII mass spectrometer equipped with an electrospray ionization (ESI) source (Bruker Daltonics, Billerica, MA, USA). HRMS data were given in *m*/*z* within a tolerance of 10 ppm of the theoretically calculated value, and measurements were given in Da. IR spectra were recorded on a PerkinElmer attenuated total reflectance (ATR) spectrophotometer (PerkinElmer, Waltham, MA, USA). Melting points were determined using a melting point apparatus with hot-stage microscopy from Bibby Stuart Scientific Company (Staffordshire, UK). All reactions were monitored by TLC performed on a silica gel 60Ȧ F_254_ aluminum sheet (Merck, Darmstadt, Germany). Visualization of the developed chromatogram was performed by UV absorbance at 254 nm and 368 nm. Flash column chromatography was performed on silica gel F_60_ (mesh size 40–63 µm) purchased from SiliCycle Inc (Quebec City, QC, Canada). All commercially available reagents (Oakwood Chemical, Estill, SC, USA and Combi-Blocks, San Diego, CA, USA) were used without further purification unless otherwise noted. Anhydrous reactions were performed using flame-dried glassware (NK Laboratory, Nonthaburi, Thailand) and standard syringe-septum techniques. Tetrahydrofuran (THF, Fisher Scientific, Waltham, MA, USA) was dried by distillation from sodium benzophenone ketyl under a nitrogen atmosphere. *N*,*N*-Dimethylformamide (DMF, RCI Labscan, Bangkok, Thailand) was dried over 3Ȧ molecular sieves for 3 days, followed by distillation under high vacuum, and stored over 3Ȧ molecular sieves under a nitrogen atmosphere. Other commercial grade solvents were purchased from Srithongtaveep (Nakhon Pathom, Thailand) and used without further purification.

### 3.2. Synthetic Procedures

#### 3.2.1. Typical Procedure for the Synthesis of Indole-3-carbaldehydes **7a**–**7c**

A Schlenk flask containing *N*,*N*-dimethylformamide (DMF, 11.0 mL) was connected to a condenser, placed under a nitrogen atmosphere, and cooled in an ice bath. Phosphoryl chloride (1.2 equiv) was added dropwise, and the mixture was stirred at 0–5 °C for 20 min. A solution of methylindole **6a**, **6b**, or **6c** (1 equiv) in DMF (11.0 mL) was then added to the reaction mixture. The temperature was raised and maintained at 35 °C for 40 min, after which the mixture was allowed to cool to room temperature. Ice (~25 g) was added, followed by 5 M aqueous NaOH (70 mL). The mixture was then heated at 95 °C for 40 min, cooled to room temperature, and treated with another portion of ice (~25 g). Stirring was continued for an additional 40 min, and the resulting precipitate was collected by filtration and washed several times with distilled water to afford the desired indole derivative.

#### 3.2.2. 2,5-Dimethyl-1*H*-indole-3-carbaldehyde (**7a**)

The reaction of **6a** (5.00 g, 34.4 mmol) with phosphoryl chloride (3.85 mL, 41.3 mmol) afforded **7a** as a reddish brown solid (5.79 g, 97% yield); TLC: R*_f_* = 0.30 (EtOAc/hexane = 1:1); m.p. 223–224 °C; ^1^H NMR (400 MHz, DMSO-*d*_6_) δ 11.86 (br s, 1H), 10.02 (s, 1H), 7.85 (s, 1H), 7.26 (d, *J* = 8.2 Hz, 1H), 6.99 (dd, *J* = 8.3, 1.7 Hz, 1H), 2.65 (s, 3H), 2.38 (s, 3H); ^13^C NMR (100 MHz, DMSO-*d*_6_) δ 184.1, 148.4, 133.6, 130.7, 125.9, 123.9, 119.9, 113.4, 111.0, 21.2, 11.5; IR (neat) 3155 (N–H), 2807 (ald C–H), 1631 (C=O), 1615 (Ar C=C) cm^−1^.

#### 3.2.3. 2-Methyl-1*H*-indole-3-carbaldehyde (**7b**)

The reaction of **6b** (5.00 g, 38.1 mmol) with phosphoryl chloride (42.6 mL, 45.7 mmol) afforded **7b** as a dark brown solid, (5.43 g, 89% yield); R*_f_* = 0.30 (EtOAc/hexane = 1:1); m.p. 203–204 °C; ^1^H NMR (400 MHz, DMSO-*d*_6_) δ 11.98 (br s, 1H), 10.05 (s, 1H), 8.06–8.01 (m, 1H), 7.40–7.36 (m, 1H), 7.19–7.12 (m, 2H), 2.68 (s, 3H); ^13^C{^1^H} NMR (100 MHz, DMSO-*d*_6_) δ 184.3, 148.6, 135.4, 125.6, 122.7, 121.9, 120.0, 113.7, 111.4, 11.5; IR (neat) 3264 (N–H), 2809 (ald C–H), 1634 (C=O), 1620 (Ar C=C) cm^−1^.

#### 3.2.4. 5-Methoxy-2-methyl-1*H*-indole-3-carbaldehyde (**7c**)

The reaction of **6c** (5.00 g, 31.0 mmol) with phosphoryl chloride (3.47 mL, 37.2 mmol) afforded **7c** as a light brown solid (5.69 g, 97% yield); R*_f_* = 0.30 (EtOAc/hexane = 1:1); m.p. 189–190 °C; ^1^H NMR (400 MHz, DMSO-*d*_6_) δ 11.84 (br s, 1H), 10.01 (s, 1H), 7.57 (d, *J* = 2.5 Hz, 1H), 7.27 (d, *J* = 8.7 Hz, 1H), 6.79 (dd, *J* = 8.7, 2.6 Hz, 1H), 3.77 (s, 3H), 2.65 (s, 3H); ^13^C{^1^H} NMR (100 MHz, DMSO-*d*_6_) δ 184.1, 155.5, 148.6, 130.1, 126.4, 113.7, 112.1, 111.9, 102.4, 55.3, 11.5; IR (neat) 3171 (N–H), 2827 (ald C–H), 1620 (C=O) cm^−1^.

#### 3.2.5. Typical Procedure for the Synthesis of Indole-3-carbonitriles **3a**–**3c**

To a solution of anhydrous aluminum chloride (AlCl_3_, 2 equiv) in tetrahydrofuran (THF) in a Schlenk flask under a nitrogen atmosphere, indole **7a**, **7b**, or **7c** (1 equiv) was added, followed by sodium azide (NaN_3_, 4 equiv). The reaction mixture was heated at reflux, and the progress was monitored by thin-layer chromatography (TLC). Upon completion, the solvent was removed under reduced pressure. The residue was treated with 10% aqueous HCl (25 mL), and the resulting mixture was extracted with dichloromethane (DCM, 3 × 100 mL). The combined organic layers were dried over anhydrous sodium sulfate and concentrated under reduced pressure. The crude residue was purified by column chromatography on silica gel using hexane/ethyl acetate (3:1) as the eluent to afford the desired indole derivative.

#### 3.2.6. 2,5-Dimethyl-1*H*-indole-3-carbonitrile (**3a**)

The reaction of **7a** (2.00 g, 11.6 mmol) with AlCl_3_ (3.08 g, 21.3 mmol) and NaN_3_ (3.00 g, 46.2 mmol) in THF (50 mL) was carried out for 16 h, affording indole **3a** as a reddish-brown solid (1.59 g, 81% yield); TLC: R*_f_* = 0.50 (EtOAc/hexane = 1:1); m.p. 192–193 °C; ^1^H NMR (400 MHz, DMSO-*d*_6_) δ 11.94 (br s, 1H), 7.30 (d, *J* = 8.5 Hz, 1H), 7.29 (s, 1H), 7.01 (dd, *J* = 8.3, 1.7 Hz, 1H), 2.51 (s, 3H), 2.38 (s, 3H); ^13^C{^1^H} NMR (100 MHz, DMSO-*d*_6_) δ 145.8, 133.1, 130.4, 127.6, 124.1, 117.4, 116.7, 111.7, 82.3, 21.1, 12.6; IR (neat) 3248 (N–H), 2214 (C≡N) cm^−1^.

#### 3.2.7. 2-Methyl-1*H*-indole-3-carbonitrile (**3b**)

The reaction of **7b** (2.00 g, 12.6 mmol) with AlCl_3_ (3.35 g, 25.1 mmol) and NaN_3_ (3.27 g, 50.3 mmol) in THF (50 mL) was carried out for 16 h, affording indole **3b** as a reddish-brown solid (1.59 g, 81% yield); TLC: R*_f_* = 0.50 (EtOAc/hexane = 1:1); m.p. 210–211 °C; ^1^H NMR (400 MHz, DMSO-*d*_6_) δ 12.08 (br s, 1H), 7.52–7.49 (m, 1H), 7.44–7.42 (m, 1H), 7.22–7.14 (m, 2H), 2.54 (s, 3H); ^13^C{^1^H} NMR (100 MHz, DMSO-*d*_6_) δ 146.0, 134.9, 127.2, 122.7, 121.4, 117.7, 116.5, 112.1, 82.8, 12.6; IR (neat) 3229 (N–H), 2214 (C≡N) cm^−1^.

#### 3.2.8. 5-Methoxy-2-methyl-1*H*-indole-3-carbonitrile (**3c**)

The reaction of **7c** (1.00 g, 5.29 mmol) with AlCl_3_ (1.41 g, 10.6 mmol) and NaN_3_ (1.38 g, 21.2 mmol) in THF (25 mL) was carried out for 16 h, affording indole **3c** as a reddish-brown solid (707 mg, 72% yield); TLC: R*_f_* = 0.50 (EtOAc/hexane = 1:1); m.p. 181–182 °C; ^1^H NMR (400 MHz, DMSO-*d*_6_) δ 11.93 (br s, 1H), 7.31 (d, *J* = 8.8 Hz, 1H), 6.97 (d, *J* = 2.4 Hz, 1H), 6.81 (dd, *J* = 8.8, 2.5 Hz, 1H), 3.78 (s, 3H), 3.50 (s, 3H); ^13^C{^1^H}NMR (100 MHz, DMSO-*d*_6_) δ 155.1, 146.0, 129.6, 128.1, 116.8, 113.0, 112.5, 99.7, 82.8, 55.4, 12.6; IR (neat) 3261 (N–H), 2211 (C≡N) cm^−1^.

#### 3.2.9. Typical Procedure for the Synthesis of Indolo[1,2-*a*]quinoline Derivatives **5ab**–**5cu**

To a mixture of indole-3-carbonitrile **3a**, **3b**, or **3c** (1.00 mmol) and Cs_2_CO_3_ (3.00 mmol) in DMF (3 mL) in a Schlenk flask equipped with a condenser under a nitrogen atmosphere, a 2-fluorobenzaldehyde derivative (**4b**–**4u**) (1.6 mmol). The reaction mixture was then heated at 130 °C and monitored by thin-layer chromatography (TLC) every 30 min. Upon completion, the mixture was allowed to cool to room temperature, diluted with water (100 mL), and extracted with ethyl acetate (3 × 100 mL). The combined organic layers were dried over anhydrous sodium sulfate and concentrated under reduced pressure. The crude residue was purified by flash column chromatography on silica gel (hexane/dichloromethane = 5:1) (method A) or by crystallization from hexane/dichloromethane (method B) to afford the desired product (**5ab**–**5cu**).

#### 3.2.10. 1-Fluoro-9-methylindolo[1,2-*a*]quinoline-7-carbonitrile (**5ab**)

The mixture was heated for 0.5 h and purified by method B to give a greenish yellow solid (109 mg, 40%); TLC: R*_f_* = 0.40 (dichloromethane/hexane = 1:1); m.p. 195–196 °C; ^1^H NMR (400 MHz, DMSO-*d*_6_) δ 8.05 (t, *J* = 8.5 Hz, 1H), 7.85 (dd, *J* = 7.7, 1.4 Hz, 1H), 7.82 (dd, *J* = 9.4, 1.9 Hz, 1H), 7.72 (d, *J* = 9.2 Hz, 1H), 7.68 (dd, *J* = 8.1, 1.5 Hz, 1H), 7.64–7.57 (m, 2H), 7.31 (dd, *J* = 8.9, 2.0 Hz, 1H) (The methyl singlet (~2.57 ppm) is obscured by the residual DMSO-*d*_6_ solvent peak); ^1^H NMR (400 MHz, CDCl_3_) δ 8.01 (dd, *J* = 8.9, 7.3 Hz, 1H), 7.64 (s, 1H), 7.56 (d, *J* = 9.3 Hz, 1H), 7.51–7.49 (m, 1H), 7.43–7.37 (m, 3H), 7.23 (dd, *J* = 8.9, 1.8 Hz, 1H), 2.54 (s, 3H); ^13^C{^1^H} NMR (100 MHz, DMSO-*d*_6_) δ 150.8 (d, *J* = 248.7 Hz), 141.8, 134.1, 132.0, 128.6, 128.5, 127.1 (d, *J* = 2.6 Hz), 126.0 (d, *J* = 8.7 Hz), 125.3 (d, *J* = 2.9 Hz), 125.2 (d, *J* = 4.5 Hz), 122.1 (d, *J* = 11.4 Hz), 118.0, 117.3 (d, *J* = 22.4 Hz), 117.1, 116.5 (d, *J* = 30.1 Hz), 115.3, 79.4, 20.9; ^19^F NMR δ −110.49–−110.54 (m); HRMS (ESI) *m*/*z* [M+Na]^+^ calcd for C_18_H_11_FN_2_Na^+^ 297.0798, found 297.0814; IR (neat) 2209 (C≡N) cm^−1^.

#### 3.2.11. 2-Fluoro-9-methylindolo[1,2-*a*]quinoline-7-carbonitrile (**5ac**)

The mixture was heated for 3.5 h and purified by method A to give a greenish yellow solid (69 mg, 25%); TLC: R*_f_* = 0.30 (dichloromethane/hexane = 1:1); m.p. 229–230 °C; ^1^H NMR (400 MHz, DMSO-*d*_6_) δ 8.62 (d, *J* = 8.9 Hz, 1H), 8.53 (d, *J* = 11.2 Hz, 1H), 8.10 (t, *J* = 7.7 Hz, 1H), 7.83 (d, *J* = 9.4 Hz, 1H), 7.65–7.63 (m, 2H), 7.47 (t, *J* = 8.1 Hz, 1H), 7.36 (d, *J* = 8.8 Hz, 1H), 2.52 (s, 3H); ^13^C{^1^H} NMR (100 MHz, DMSO-*d*_6_) δ 162.8 (d, *J* = 248.0 Hz), 141.5, 135.7 (d, *J* = 11.5 Hz), 134.4, 132.1 (d, *J* = 10.0 Hz), 130.2, 128.9, 128.8, 125.8, 120.7 (d, *J* = 2.3 Hz), 118.5, 115.51, 115.50, 115.2 (d, *J* = 2.6 Hz), 112.8 (d, *J* = 22.6 Hz), 103.4 (d, *J* = 28.0 Hz), 78.1, 20.9; ^19^F NMR δ −106.56 (dt, *J* = 11.1, 7.1 Hz); HRMS (ESI) *m*/*z* [M+Na]^+^ calcd for C_18_H_11_FN_2_Na^+^ 297.0798, found 297.0816; IR (neat) 2204 (C≡N) cm^−1^.

#### 3.2.12. 4-Fluoro-9-methylindolo[1,2-*a*]quinoline-7-carbonitrile (**5ae**)

The mixture was heated for 0.5 h and purified by method B to give a greenish yellow solid (177 mg, 65%);TLC: R*_f_* = 0.40 (dichloromethane/hexane = 1:1); m.p. 218–219 °C; ^1^H NMR (400 MHz, CDCl_3_) δ 8.40 (d, *J* = 8.7 Hz, 1H), 8.31 (d, *J* = 8.8 Hz, 1H), 7.79 (d, *J* = 9.5 Hz, 1H), 7.76 (s, 1H), 7.71–7.66 (m, 2H), 7.35 (dd, *J* = 8.9, 1.8 Hz, 1H), 7.19 (d, *J* = 8.7 Hz, 1H), 2.58 (s, 3H); ^13^C{^1^H} NMR (100 MHz, DMSO-*d*_6_) δ 158.4 (d, *J* = 250.0 Hz), 141.1, 135.7 (d, *J* = 5.5 Hz), 134.5, 131.6 (d, *J* = 9.9 Hz), 130.4, 128.8, 126.2, 120.7 (d, *J* = 7.2 Hz), 118.7, 116.9 (d, *J* = 1.9 Hz), 115.6, 115.4, 113.2 (d, *J* = 18.7 Hz), 112.8 (d, *J* = 3.5 Hz), 110.6 (d, *J* = 20.2 Hz), 78.9, 20.9; ^19^F NMR δ −110.49–−110.54 (m); HRMS (ESI) *m*/*z* [M+Na]^+^ calcd for C_18_H_11_FN_2_Na^+^ 297.0798, found 297.0818; IR (neat) 2207 (C≡N) cm^−1^.

#### 3.2.13. 1-Chloro-9-methylindolo[1,2-*a*]quinoline-7-carbonitrile (**5af**)

The mixture was heated for 1 h and purified by method B to give a greenish yellow solid (78 mg, 27%); TLC: R*_f_* = 0.40 (dichloromethane/hexane = 1:1); m.p. 201–202 °C; ^1^H NMR (400 MHz, DMSO-*d*_6_) δ 8.01 (dd, *J* = 7.7, 1.4 Hz, 1H), 7.89–7.86 (m, 2H), 7.80 (d, *J* = 9.3 Hz, 1H), 7.75 (d, *J* = 9.3 Hz, 1H), 7.66 (dt, *J* = 1.7, 0.9 Hz, 1H), 7.63 (t, *J* = 7.8 Hz, 1H), 7.32 (dd, *J* = 8.9, 1.7 Hz, 1H), 2.53 (s, 3H); ^13^C{^1^H}NMR (100 MHz, DMSO-*d*_6_) δ 142.6, 134.0, 132.21, 132.16, 130.8, 128.4, 128.3, 127.8, 127.7, 126.5, 124.0, 121.6, 118.7, 117.9, 117.0, 115.4, 79.4, 21.0; HRMS (ESI) *m*/*z* [M+Na]^+^ calcd for C_18_H_11_ClN_2_Na^+^ 313.0503, found 313.0486; IR (neat) 2208 (C≡N) cm^−1^.

#### 3.2.14. 2-Chloro-9-methylindolo[1,2-*a*]quinoline-7-carbonitrile (**5ag**)

The mixture was heated for 24 h and purified by method A to give a greenish yellow solid (48 mg, 17%); TLC: R*_f_* = 0.40 (dichloromethane/hexane = 1:1); m.p. 266–267 °C; ^1^H NMR (400 MHz, CDCl_3_) δ 8.52 (d, *J* = 1.9 Hz, 1H), 8.21 (d, *J* = 8.7 Hz, 1H), 7.73 (s, 1H), 7.70 (d, *J* = 8.4 Hz, 1H), 7.59 (d, *J* = 9.3 Hz, 1H), 7.45 (d, *J* = 9.2 Hz, 1H), 7.43 (dd, *J* = 8.5, 1.8 Hz, 1H), 7.34 (dd, *J* = 8.8, 1.8 Hz, 1H), 2.58 (s, 3H); ^13^C{^1^H} NMR (100 MHz, CDCl_3_) δ 141.8, 136.4, 135.9, 134.8, 130.9, 130.6, 129.8, 127.6, 126.0, 125.0, 122.9, 120.0, 117.1, 116.2, 115.9, 114.5, 80.2, 21.6; HRMS (ESI) *m*/*z* [M+Na]^+^ calcd for C_18_H_11_ClN_2_Na^+^ 313.0503, found 313.0497; IR (neat) 2206 (C≡N) cm^−1^.

#### 3.2.15. 3-Chloro-9-methylindolo[1,2-*a*]quinoline-7-carbonitrile (**5ah**)

The mixture was heated for 24 h and purified by method A to give a greenish yellow solid (29 mg, 10%); TLC: R*_f_* = 0.50 (dichloromethane/hexane = 1:1), m.p. 230–231 °C; ^1^H NMR (400 MHz, CDCl_3_) δ 8.44 (d, *J* = 9.1 Hz, 1H), 8.18 (d, *J* = 8.8 Hz, 1H), 7.71–7.70 (m, 2H), 7.63–7.58 (m, 2H), 7.36 (d, *J* = 9.3 Hz, 1H), 7.30 (dd, *J* = 8.8, 1.8 Hz, 1H), 2.56 (s, 3H).^13^C{^1^H} NMR (100 MHz, DMSO-*d*_6_) δ 141.1, 134.3, 133.6, 130.4, 130.1, 128.78, 128.75, 128.7, 128.3, 126.0, 125.5, 118.7, 118.3, 117.4, 115.44, 115.41, 78.5, 20.9; HRMS (ESI) *m*/*z* [M+Na]^+^ calcd for C_18_H_11_ClN_2_Na^+^ 313.0503, found 313.0500; IR (neat) 2206 (C≡N) cm^−1^.

#### 3.2.16. 4-Chloro-9-methylindolo[1,2-*a*]quinoline-7-carbonitrile (**5ai**)

The mixture was heated for 0.5 h and purified by method B to give a greenish yellow solid (215 mg, 74%); TLC: R*_f_* = 0.50 (dichloromethane/hexane = 1:1); m.p. 244–245 °C; ^1^H NMR (400 MHz, CDCl_3_) δ 8.50 (d, *J* = 8.6 Hz, 1H), 8.26 (d, *J* = 8.8 Hz, 1H), 7.95 (d, *J* = 9.7 Hz, 1H), 7.72 (s, 1H), 7.66 (dd, *J* = 9.6, 1.1 Hz, 1H), 7.62 (t, *J* = 8.2 Hz, 1H), 7.52 (d, *J* = 7.8 Hz, 1H), 7.32 (dd, *J* = 8.8, 1.8 Hz, 1H), 2.57 (s, 3H); ^13^C{^1^H} NMR (100 MHz, CDCl_3_) δ 141.4, 137.1, 134.7, 133.8, 131.2, 130.0, 129.9, 126.1, 125.4, 124.2, 122.4, 120.0, 117.9, 115.9, 114.8(2C), 80.2, 21.6; HRMS (ESI) *m*/*z* [M+Na]^+^ calcd for C_18_H_11_ClN_2_Na^+^ 313.0503, found 313.0503; IR (neat) 2209 (C≡N) cm^−1^.

#### 3.2.17. 1-Bromo-9-methylindolo[1,2-*a*]quinoline-7-carbonitrile (**5aj**)

The mixture was heated for 3 h and purified by method B to give a greenish yellow solid (45 mg, 13%); TLC: R*_f_* = 0.40 (dichloromethane/hexane = 1:1); m.p. 218–219 °C; ^1^H NMR (400 MHz, CDCl_3_) δ 7.87 (d, *J* = 8.7 Hz, 1H), 7.86 (dd, *J* = 7.9, 1.4 Hz, 1H), 7.72–7.66 (m, 2H), 7.59 (d, *J* = 9.2 Hz, 1H), 7.350 (d, *J* = 9.3 Hz, 1H), 7.347 (t, *J* = 7.8 Hz, 1H), 7.24 (dd, *J* = 8.7, 1.7 Hz, 1H), 2.57 (s, 3H); ^13^C{^1^H} NMR (100 MHz, DMSO-*d*_6_) δ 142.6, 135.9, 134.1, 132.3, 131.6, 128.4, 128.3, 128.2, 127.9, 126.8, 123.7, 119.3, 117.9, 116.8, 115.4, 110.1, 79.2, 21.0; HRMS (ESI) *m*/*z* [M+Na]^+^ calcd for C_18_H_11_BrN_2_Na^+^ requires 356.9998, found 356.9990; IR (neat) 2208 (C≡N) cm^−1^.

#### 3.2.18. 2-Bromo-9-methylindolo[1,2-*a*]quinoline-7-carbonitrile (**5ak**)

The mixture was heated for 2 h and purified by method B to give a greenish yellow solid (87 mg, 26%); TLC: R*_f_* = 0.30 (dichloromethane/hexane = 1:1); m.p. 273–274 °C; ^1^H NMR (400 MHz, CDCl_3_) δ 8.70 (s, 1H), 8.22 (d, *J* = 8.8 Hz, 1H), 7.74 (s, 1H), 7.65–7.56 (m, 3H), 7.45 (d, *J* = 9.3 Hz, 1H), 7.36 (dd, *J* = 8.7, 1.8 Hz, 1H), 2.59 (s, 3H); ^13^C{^1^H} NMR (100 MHz, CDCl_3_) δ 141.7, 136.6, 134.8, 130.9, 130.7, 129.8, 127.8, 127.7, 126.1, 123.9, 123.2, 120.0, 119.1, 117.3, 115.9, 114.5, 80.3, 21.6; HRMS (ESI) *m*/*z* [M+Na]^+^ calcd for C_18_H_11_BrN_2_Na^+^ 356.9998, found 356.9990; IR (neat) 2206 (C≡N) cm^−1^.

#### 3.2.19. 3-Bromo-9-methylindolo[1,2-*a*]quinoline-7-carbonitrile (**5al**)

The mixture was heated for 1 h and purified by method B to give a greenish yellow solid (175 mg, 52%); TLC: R*_f_* = 0.40 (dichloromathane/hexane = 1:1); m.p. 258–259 °C; ^1^H NMR (400 MHz, DMSO-*d*_6_) δ 8.79 (d, *J* = 9.1 Hz, 1H), 8.62 (d, *J* = 8.8 Hz, 1H), 8.30 (d, *J* = 2.4 Hz, 1H), 7.92 (dd, *J* = 9.1, 2.5 Hz, 1H), 7.81 (d, *J* = 9.4 Hz, 1H), 7.77 (d, *J* = 9.4 Hz, 1H), 7.70 (d, *J* = 1.7 Hz, 1H), 7.41 (dd, *J* = 8.8, 1.8 Hz, 1H), 2.54 (s, 3H); ^13^C{^1^H} NMR (100 MHz, DMSO-*d*_6_) δ 141.1, 134.4, 134.0, 132.9, 131.7, 130.4, 128.8, 128.2, 126.0, 125.9, 118.7, 118.5, 117.4, 116.8, 115.48, 115.45, 78.5, 20.9; HRMS (ESI) *m*/*z* [M+Na]^+^ calcd for C_18_H_11_BrN_2_Na^+^ 356.9998, found 357.0001; IR (neat) 2204 (C≡N) cm^−1^.

#### 3.2.20. 4-Bromo-9-methylindolo[1,2-*a*]quinoline-7-carbonitrile (**5am**)

The mixture was heated for 1.5 h and purified by method B to give a greenish yellow solid (257 mg, 77%); TLC: R*_f_* = 0.40 (dichloromethane/hexane = 1:1); m.p. 248–249 °C; ^1^H NMR (400 MHz, CDCl_3_) δ 8.57 (d, *J* = 8.5 Hz, 1H), 8.28 (d, *J* = 8.8 Hz, 1H), 7.95 (d, *J* = 9.7 Hz, 1H), 7.74 (s, 1H), 7.73 (d, *J* = 7.9 Hz, 1H), 7.67 (d, *J* = 9.7 Hz, 1H), 7.55 (t, *J* = 8.2 Hz, 1H), 7.33 (dd, *J* = 8.8, 1.8 Hz, 1H), 2.58 (s, 3H); ^13^C{^1^H} NMR (100 MHz, CDCl_3_) δ 141.5, 137.1, 134.7, 131.1, 130.2, 130.1, 128.9, 127.0, 126.1, 124.4, 123.7, 120.1, 118.2, 115.9, 115.5, 114.8, 80.2, 21.6; HRMS (ESI) *m*/*z* [M+Na]^+^ calcd for C_18_H_11_BrN_2_Na^+^ 356.9998, found 356.9993; IR (neat) 2206 (C≡N) cm^−1^.

#### 3.2.21. 3-Iodo-9-methylindolo[1,2-*a*]quinoline-7-carbonitrile (**5an**)

The mixture was heated for 3 h and purified by method A to give a greenish yellow solid (145 mg, 38%); TLC: R*_f_* = 0.30 (dichloromethane/hexane = 1:1), m.p. 275–276 °C; ^1^H NMR (400 MHz, CDCl_3_) δ 8.32 (d, *J* = 8.9 Hz, 1H), 8.24 (d, *J* = 8.8 Hz, 1H), 8.10 (d, *J* = 2.1 Hz, 1H), 7.95 (dd, *J* = 8.9, 2.1 Hz, 1H), 7.75 (s, 1H), 7.62 (d, *J* = 9.3 Hz, 1H), 7.38 (d, *J* = 9.3 Hz, 1H), 7.34 (dd, *J* = 8.8, 1.8 Hz, 1H), 2.58 (s, 3H); ^13^C{^1^H} NMR (100 MHz, CDCl_3_) δ 141.5, 138.4, 138.0, 135.5, 134.7, 131.0, 129.9, 127.0, 126.4, 126.1, 120.1, 118.0, 117.7, 115.9, 114.6, 87.9, 80.4, 21.6; HRMS (ESI) *m*/*z* [M+Na]^+^ calcd for C_18_H_11_IN_2_Na^+^ 404.9859, found 404.9865; IR (neat) 2206 (C≡N) cm^−1^.

#### 3.2.22. 1,9-Dimethylindolo[1,2-*a*]quinoline-7-carbonitrile (**5ao**)

The mixture was heated for 3.5 h and purified by method A to give a greenish yellow solid (60 mg, 22%); TLC: R*_f_* = 0.40 (dichloromethane/hexane = 1:1); m.p. 163–164 °C; ^1^H NMR (400 MHz, CDCl_3_) δ 7.70 (s, 1H), 7.56 (dd, *J* = 7.5, 1.6 Hz, 1H), 7.54–7.51 (m, 2H), 7.49 (dd, *J* = 7.6, 1.6 Hz, 1H), 7.42 (t, *J* = 7.5 Hz, 1H), 7.40 (d, *J* = 9.2 Hz, 1H), 7.21 (dd, *J* = 8.6, 1.7 Hz, 1H), 2.56 (s, 3H), 2.51 (s, 3H); ^13^C{^1^H} NMR (100 MHz, CDCl_3_) δ 143.5, 133.9, 133.7, 133.5, 133.2, 129.4, 128.6, 127.0, 126.4, 126.2, 125.1, 124.1, 119.2, 116.6, 116.3, 116.2, 79.6, 22.3, 21.6; HRMS (ESI) *m*/*z* [M+Na]^+^ calcd for C_19_H_14_N_2_Na^+^ 293.1049, found 293.1058; IR (neat) 2201 (C≡N) cm^−1^.

#### 3.2.23. 2,9-Dimethylindolo[1,2-*a*]quinoline-7-carbonitrile (**5ap**)

The mixture was heated for 24 h and purified by method A to give a greenish yellow solid (37 mg, 14%); TLC: R*_f_* = 0.30 (dichloromethane/hexane = 1:1); m.p. 216–217 °C; ^1^H NMR (400 MHz, CDCl_3_) δ 8.21 (s, 1H), 8.16 (d, *J* = 8.7 Hz, 1H), 7.62 (s, 1H), 7.57 (d, *J* = 7.9 Hz, 1H), 7.42 (d, *J* = 9.3 Hz, 1H), 7.35 (d, *J* = 9.3 Hz, 1H), 7.23–7.21 (m, 2H), 2.60 (s, 3H), 2.53 (s, 3H); ^13^C{^1^H} NMR (100 MHz, CDCl_3_) δ 142.3, 140.8, 136.2, 134.2, 131.1, 130.0, 129.5, 128.4, 125.8, 125.3, 122.1, 119.7, 116.5, 116.3, 115.8, 114.9, 79.0, 22.6, 21.6; HRMS (ESI) *m*/*z* [M+Na]^+^ calcd for C_19_H_14_N_2_Na^+^ 293.1049, found 293.1059; IR (neat) 2200 (C≡N) cm^−1^.

#### 3.2.24. 3,9-Dimethylindolo[1,2-*a*]quinoline-7-carbonitrile (**5aq**)

The mixture was heated for 5.5 h and purified by method B to give a greenish yellow solid (49 mg, 18%); TLC: R*_f_* = 0.40 (dichloromethane/hexane = 1:1); m.p. 179–180 °C; ^1^H NMR (400 MHz, CDCl_3_) δ 8.38 (d, *J* = 8.6 Hz, 1H), 8.22 (d, *J* = 8.8 Hz, 1H), 7.68 (s, 1H), 7.52 (d, *J* = 9.5 Hz, 1H), 7.50 (s, 1H), 7.47 (dd, *J* = 8.7, 2.1 Hz, 1H), 7.38 (d, *J* = 9.3 Hz, 1H), 7.26 (dd, *J* = 8.8, 1.8 Hz, 1H), 2.55 (s, 3H), 2.49 (s, 3H); ^13^C{^1^H} NMR (100 MHz, CDCl_3_) δ 141.9, 134.3, 134.14, 134.10, 131.2, 131.1, 129.9, 129.6, 128.4, 125.4, 124.4, 119.8, 116.8, 116.5, 115.9, 114.7, 79.0, 21.6, 21.0; HRMS (ESI) *m*/*z* [M+Na]^+^ calcd for C_19_H_14_N_2_Na^+^ 293.1049, found 293.1069; IR (neat) 2201 (C≡N) cm^−1^.

#### 3.2.25. 1-Methoxy-9-methylindolo[1,2-*a*]quinoline-7-carbonitrile (**5ar**)

The mixture was heated for 24 h and purified by method B to give a greenish yellow solid (96 mg, 34%); TLC: R*_f_* = 0.30 (dichloromethane/hexane = 1:1); m.p. 164–165 °C; ^1^H NMR (400 MHz, CDCl_3_) δ 7.92 (d, *J* = 8.9 Hz, 1H), 7.68 (d, *J* = 1.8 Hz, 1H), 7.61 (d, *J* = 9.3 Hz, 1H), 7.44 (t, *J* = 7.8 Hz, 1H), 7.42 (d, *J* = 9.3 Hz, 1H), 7.37 (dd, *J* = 7.8, 1.5 Hz, 1H), 7.23 (dd, *J* = 8.1, 1.5 Hz, 1H), 7.21 (dd, *J* = 9.4, 1.8 Hz, 1H), 3.98 (s, 3H), 2.56 (s, 3H); ^13^C{^1^H} NMR (100 MHz, CDCl_3_) δ 149.5, 142.9, 133.8, 133.6, 129.5, 127.9, 127.2, 125.5, 125.2, 124.0, 121.2, 118.6, 118.5, 117.4, 116.6, 112.4, 79.9, 55.9, 21.5; HRMS (ESI) *m*/*z* [M+Na]^+^ calcd for C_19_H_14_N_2_NaO^+^ 309.0998, found 309.1000; IR (neat) 2201 (C≡N) cm^−1^.

#### 3.2.26. 2-Methoxy-9-methylindolo[1,2-*a*]quinoline-7-carbonitrile (**5as**)

The mixture was heated for 2 h and purified by method B to give a greenish yellow solid (184 mg, 64%); TLC: R*_f_* = 0.30 (dichloromethane/hexane = 1:1); m.p. 206–207 °C; ^1^H NMR (400 MHz, CDCl_3_) δ 8.26 (d, *J* = 8.8 Hz, 1H), 8.05 (d, *J* = 2.3 Hz, 1H), 7.72 (s, 1H), 7.70 (d, *J* = 8.7 Hz, 1H), 7.47 (d, *J* = 9.3 Hz, 1H), 7.44 (d, *J* = 9.3 Hz, 1H), 7.29 (dd, *J* = 8.8, 1.8 Hz, 1H), 7.05 (dd, *J* = 8.7, 2.3 Hz, 1H), 4.03 (s, 3H), 2.57 (s, 3H); ^13^C{^1^H} NMR (100 MHz, CDCl_3_) δ 161.3, 142.6, 137.2, 134.3, 130.92, 130.88, 130.1, 128.4, 125.2, 119.7, 118.3, 116.5, 114.6, 114.1, 111.5, 101.4, 78.9, 56.0, 21.6; HRMS (ESI) *m*/*z* [M+Na]^+^ calcd for C_19_H_14_N_2_NaO^+^ 309.0998, found 309.1001; IR (neat) 2204 (C≡N) cm^−1^.

#### 3.2.27. 3-Methoxy-9-methylindolo[1,2-*a*]quinoline-7-carbonitrile (**5at**)

The mixture was heated for 24 h and purified by method B to give a greenish yellow solid (189 mg, 66%); TLC: R*_f_* = 0.30 (dichloromethane/hexane = 1:1); m.p. 178–179 °C; ^1^H NMR (400 MHz, CDCl_3_) δ 8.51 (d, *J* = 9.3 Hz, 1H), 8.27 (d, *J* = 8.8 Hz, 1H), 7.73 (s, 1H), 7.61 (d, *J* = 9.3 Hz, 1H), 7.44 (d, *J* = 9.3 Hz, 1H), 7.30 (d, *J* = 9.2 Hz, 1H), 7.29 (d, *J* = 9.3 Hz, 1H), 7.20 (d, *J* = 2.9 Hz, 1H), 3.94 (s, 3H), 2.57 (s, 3H); ^13^C{^1^H} NMR (100 MHz, CDCl_3_) δ 156.1, 141.4, 134.1, 130.9, 130.5, 129.8, 128.2, 125.7, 125.4, 119.8, 118.1, 117.33, 117.28, 116.5, 114.5, 111.6, 78.8, 55.9, 21.6; HRMS (ESI) *m*/*z* [M+Na]^+^ calcd for C_19_H_14_N_2_NaO^+^ 309.0998, found 309.1002; IR (neat) 2209 (C≡N) cm^−1^.

#### 3.2.28. 4-Methoxy-9-methylindolo[1,2-*a*]quinoline-7-carbonitrile (**5au**)

The mixture was heated for 0.5 h and purified by method B to give a greenish yellow solid (237 mg, 83%); TLC: R*_f_* = 0.30 (dichloromethane/hexane = 1:1); m.p. 245–246 °C; ^1^H NMR (400 MHz, CDCl_3_) δ 8.31 (d, *J* = 8.8 Hz, 1H), 8.19 (d, *J* = 8.7 Hz, 1H), 7.99 (d, *J* = 9.6 Hz, 1H), 7.73 (s, 1H), 7.64 (t, *J* = 8.4 Hz, 1H), 7.57 (d, *J* = 9.6 Hz, 1H), 7.29 (dd, *J* = 8.8, 1.8 Hz, 1H), 6.92 (d, *J* = 8.2 Hz, 1H), 4.02 (s, 3H), 2.57 (s, 3H); ^13^C{^1^H} NMR (100 MHz, CDCl_3_) δ 156.7, 142.3, 136.8, 134.3, 131.2, 130.5, 130.0, 125.3, 122.6, 119.6, 116.6, 115.4, 115.1, 115.0, 108.6, 105.2, 78.8, 56.2, 21.6; HRMS (ESI) *m*/*z* [M+Na]^+^ calcd for C_19_H_14_N_2_NaO^+^ 309.0998, found 309.1009; IR (neat) 2198 (C≡N) cm^−1^.

#### 3.2.29. 1-Fluoroindolo[1,2-*a*]quinoline-7-carbonitrile (**5bb**)

The mixture was heated for 2.5 h and purified by method A to give a greenish yellow solid (131 mg, 50%); TLC: R*_f_* = 0.20 (dichloromethane/hexane = 1:1); m.p. 137–138 °C; ^1^H NMR (400 MHz, CDCl_3_) δ 8.20 (td, *J* = 7.8, 1.4 Hz, 1H), 7.93 (dd, *J* = 7.4, 1.7 Hz, 1H), 7.66 (d, *J* = 9.3 Hz, 1H), 7.59–7.57 (m, 1H), 7.53–7.44 (m, 5H); ^13^C{^1^H} NMR (100 MHz, CDCl_3_) δ 151.6 (d, *J* = 250.9 Hz), 142.2, 134.5, 129.1, 127.7 (d, *J* = 3.0 Hz), 127.7 (d, *J* = 2.7 Hz), 125.6 (d, *J* = 8.3 Hz), 124.8 (d, *J* = 3.0 Hz), 124.4 (d, *J* = 1.2 Hz), 123.7 (d, *J* = 4.4 Hz), 123.3 (d, *J* = 11.2 Hz), 119.6, 117.9 (d, *J* = 1.5 Hz), 117.1 (d, *J* = 23.0 Hz), 116.8 (d, *J* = 30.8 Hz), 115.9, 81.8; ^19^F NMR (470 MHz, CDCl_3_) δ −109.92–−109.98 (m); HRMS (ESI) *m*/*z* [M+Na]^+^ calcd for C_17_H_9_FN_2_Na^+^ 283.0642, found 283.0648; IR (neat) 2215 (C≡N) cm^−1^.

#### 3.2.30. 2-Fluoroindolo[1,2-*a*]quinoline-7-carbonitrile (**5bc**)

The reaction mixture was performed for 3.5 h, and the crude residue was purified by flash column chromatography on silica gel to give a greenish yellow solid (47 mg, 18%); TLC: R*_f_* = 0.20 (dichloromethane/hexane = 1:1); m.p. 227–228 °C; ^1^H NMR (400 MHz, CDCl_3_) δ 8.40–8.36 (m, 1H), 8.33 (dd, *J* = 10.9, 2.3 Hz, 1H), 8.00–7.96 (m, 1H), 7.79 (dd, *J* = 8.7, 6.3 Hz, 1H), 7.61 (d, *J* = 9.4 Hz, 1H), 7.58–7.54 (m, 2H), 7.51 (d, *J* = 9.4 Hz, 1H), 7.23 (ddd, *J* = 8.6, 7.8, 2.3 Hz, 1H); ^13^C{^1^H} NMR (100 MHz, CDCl_3_) δ 163.4 (d, *J* = 250.2 Hz), 142.0, 136.8 (d, *J* = 11.0 Hz), 132.6, 131.5 (d, *J* = 9.9 Hz), 129.5, 128.1 (d, *J* = 1.6 Hz), 124.7, 124.2, 121.0 (d, *J* = 2.5 Hz), 120.4, 116.1 (d, *J* = 2.9 Hz), 115.9, 114.8, 112.8 (d, *J* = 22.7 Hz), 103.6 (d, *J* = 28.1 Hz), 80.5; ^19^F NMR (470 MHz, CDCl_3_) δ −106.35 (dt, *J* = 10.5, 7.2 Hz); HRMS (ESI) *m*/*z* [M+Na]^+^ calcd for C_17_H_9_FN_2_Na^+^ 283.0642, found 283.0649; IR (neat) 2217 (C≡N) cm^−1^.

#### 3.2.31. 3-Fluoroindolo[1,2-*a*]quinoline-7-carbonitrile (**5bd**)

The reaction mixture was performed for 4 h, and the crude residue was purified by crystallization from hexane/dichloromethane to give a greenish yellow solid (96 mg, 37%); TLC: R*_f_* = 0.20 (dichloromethane/hexane = 1:1); m.p. 216–217 °C; ^1^H NMR (400 MHz, CDCl_3_) δ 8.53 (dd, *J* = 9.2, 4.3 Hz, 1H), 8.35–8.31 (m, 1H), 7.94–7.91 (m, 1H), 7.61 (d, *J* = 9.3 Hz, 1H), 7.53–7.47 (m, 2H), 7.44–7.39 (m, 3H); ^13^C{H} NMR (100 MHz, CDCl_3_) δ 159.0 (d, *J* = 246.1 Hz), 141.5, 132.7, 132.6 (d, *J* = 2.3 Hz), 129.4, 127.8 (d, *J* = 2.9 Hz), 126.1 (d, *J* = 8.5 Hz), 124.4 (d, *J* = 19.8 Hz), 120.5, 118.2, 117.84, 117.76, 117.5, 115.9, 115.0 (d, *J* = 22.6 Hz), 114.8, 80.5; ^19^F NMR (470 MHz, CDCl_3_) δ −116.72 (td, *J* = 7.9, 4.3 Hz); HRMS (ESI) *m*/*z* [M+Na]^+^ calcd for C_17_H_9_FN_2_Na^+^ 283.0642, found 283.0647; IR (neat) 2196 (C≡N) cm^−1^.

#### 3.2.32. 4-Fluoroindolo[1,2-*a*]quinoline-7-carbonitrile (**5be**)

The reaction mixture was performed for 3 h, and the crude residue was purified by crystallization from hexane/dichloromethane to give a greenish yellow solid (98 mg, 38%); TLC: R*_f_* = 0.20 (dichloromethane/hexane = 1:1); m.p. 205–206 °C; ^1^H NMR (400 MHz, CDCl_3_) δ 8.47–8.42 (m, 1H), 8.01–7.97 (m, 1H), 7.82 (d, *J* = 9.5 Hz, 1H), 7.72–7.67 (m, 2H), 7.58–7.52 (m, 2H), 7.22 (t, *J* = 8.7 Hz, 1H); ^13^C{^1^H} NMR (100 MHz, CDCl_3_) δ 159.4 (d, *J* = 252.7 Hz), 141.7, 136.9 (d, *J* = 5.5 Hz), 132.8, 130.6 (d, *J* = 9.9 Hz), 129.5, 124.7, 124.4, 120.7 (d, *J* = 7.3 Hz), 120.4, 117.2 (d, *J* = 1.9 Hz), 115.8, 115.1, 114.4 (d, *J* = 18.4 Hz), 112.0 (d, *J* = 3.9 Hz), 110.6 (d, *J* = 20.8 Hz), 81.1; ^19^F NMR (470 MHz, CDCl_3_) δ -116.77 (dd, *J* = 9.4, 6.1 Hz). HRMS (ESI) *m*/*z* [M+Na]^+^ calcd for C_17_H_9_FN_2_Na^+^ 283.0642, found 283.0648; IR (neat) 2207 (C≡N) cm^−1^.

#### 3.2.33. 1-Chloroindolo[1,2-*a*]quinoline-7-carbonitrile (**5bf**)

The reaction mixture was performed for 3 h, and the crude residue was purified by flash column chromatography on silica gel to give a greenish yellow solid (125 mg, 45%); TLC: R*_f_* = 0.20 (dichloromethane/hexane = 1:1); m.p. 189–190 °C; ^1^H NMR (400 MHz, CDCl_3_) δ 7.98–7.93 (m, 2H), 7.70 (dd, *J* = 7.7, 1.5 Hz, 1H), 7.69 (dd, *J* = 7.9, 1.5 Hz, 1H), 7.65 (d, *J* = 9.2 Hz, 1H), 7.53–7.42 (m, 4H); ^13^C{^1^H} NMR (100 MHz, CDCl_3_) δ 143.1, 134.6, 132.2, 132.0, 129.0, 128.1, 127.4, 127.2, 126.0, 124.3, 123.0, 122.4, 119.5, 119.0, 117.7, 115.9, 81.7; HRMS (ESI) *m*/*z* [M+Na]^+^ calcd for C_17_H_9_ClN_2_Na^+^ 299.0346, found 299.0351; IR (neat) 2206 (C≡N) cm^−1^.

#### 3.2.34. 2-Chloroindolo[1,2-*a*]quinoline-7-carbonitrile (**5bg**)

The reaction mixture was performed for 4 h, and the crude residue was purified by crystallization from hexane/dichloromethane to give a greenish yellow solid (43 mg, 16%); R*_f_* = 0.10 (dichloromethane/hexane = 1:1); m.p. 224–225 °C; ^1^H NMR (400 MHz, CDCl_3_) δ 8.60 (d, *J* = 1.9 Hz, 1H), 8.41–8.37 (m, 1H), 8.00–7.96 (m, 1H), 7.72 (d, *J* = 8.4 Hz, 1H), 7.63 (d, *J* = 9.3 Hz, 1H), 7.58–7.54 (m, 2H), 7.49 (d, *J* = 9.4 Hz, 1H), 7.45 (dd, *J* = 8.4, 1.8 Hz, 1H); ^13^C{^1^H} NMR (100 MHz, CDCl_3_) δ 141.8, 136.5, 136.0, 132.6, 130.7, 129.5, 127.9, 125.2, 124.8, 124.4, 123.0, 120.4, 117.1, 116.3, 115.8, 114.9, 80.8; HRMS (ESI) *m*/*z* [M+H]^+^ calcd for C_17_H_10_ClN_2_^+^ 277.0527, found 277.0537; IR (neat) 2210 (C≡N) cm^−1^.

#### 3.2.35. 3-Chloroindolo[1,2-*a*]quinoline-7-carbonitrile (**5bh**)

The reaction mixture was performed for 5 h, and the crude residue was purified by flash column chromatography on silica gel to give a greenish yellow solid (119 mg, 43%); TLC: R*_f_* = 0.10 (dichloromethane/hexane = 1:1); m.p. 233–234 °C; ^1^H NMR (400 MHz, CDCl_3_) δ 8.57 (d, *J* = 9.0 Hz, 1H), 8.43–8.38 (m, 1H), 8.01–7.96 (m, 1H), 7.77 (d, *J* = 2.4 Hz, 1H), 7.68 (d, *J* = 9.2 Hz, 1H), 7.67 (d, *J* = 9.0 Hz, 1H), 7.58–7.51 (m, 2H), 7.44 (d, *J* = 9.4 Hz, 1H); ^13^C{^1^H} NMR (100 MHz, CDCl_3_) δ 141.5, 134.5, 132.7, 130.1, 130.0, 129.4, 128.9, 127.5, 125.8, 124.6, 124.4, 120.5, 118.2, 117.4, 115.8, 114.9, 80.9; HRMS (ESI) *m*/*z* [M+Na]^+^ calcd for C_17_H_9_ClN_2_Na^+^ 299.0346, found 299.0338; IR (neat) 2205 (C≡N) cm^−1^.

#### 3.2.36. 4-Chloroindolo[1,2-*a*]quinoline-7-carbonitrile (**5bi**)

The reaction mixture was performed for 3 h, and the crude residue was purified by crystallization from hexane/dichloromethane to give a greenish yellow solid (210 mg, 76%); R*_f_* = 0.20 (dichloromethane/hexane = 1:1); m.p. 234–235 °C; ^1^H NMR (400 MHz, CDCl_3_) δ 8.51 (d, *J* = 8.5 Hz, 1H), 8.40–8.36 (m, 1H), 7.97–7.94 (m, 2H), 7.66 (d, *J* = 9.7 Hz, 1H), 7.61 (t, *J* = 8.2 Hz, 1H), 7.55–7.49 (m, 3H); ^13^C{^1^H} NMR (100 MHz, CDCl_3_) δ 141.5, 137.1, 133.9, 132.8, 130.0, 129.6, 125.6, 124.7, 124.5, 124.4, 122.5, 120.5, 117.9, 115.7, 115.2, 114.9, 80.8; HRMS (ESI) *m*/*z* [M+Na]^+^ calcd for C_17_H_9_ClN_2_Na^+^ 299.0346, found 299.0346; IR (neat) 2206 (C≡N) cm^−1^.

#### 3.2.37. 2-Bromoindolo[1,2-*a*]quinoline-7-carbonitrile (**5bk**)

The reaction mixture was performed for 4 h, and the crude residue was purified by flash column chromatography on silica gel to give a greenish yellow solid (62 mg, 19%); TLC: R*_f_* = 0.20 (dichloromethane/hexane = 1:1); m.p. 223–224 °C; ^1^H NMR (400 MHz, CDCl_3_) δ 8.77 (d, *J* = 1.7 Hz, 1H), 8.41–8.36 (m, 1H), 7.99–7.96 (m, 1H), 7.66 (d, *J* = 8.3 Hz, 1H), 7.65 (d, *J* = 9.3 Hz, 1H), 7.60 (dd, *J* = 8.4, 1.6 Hz, 1H), 7.58–7.53 (m, 2H), 7.48 (d, *J* = 9.3 Hz, 1H); ^13^C{^1^H} NMR (100 MHz, CDCl_3_) δ 141.7, 136.6, 132.6, 130.8, 129.5, 128.00, 127.99, 124.8, 124.5, 124.0, 123.3, 120.4, 119.2, 117.3, 115.7, 114.9, 80.9; HRMS (ESI) *m*/*z* [M+H]^+^ calcd for C_17_H_10_BrN_2_^+^ 321.0022 found 321.0024; IR (neat) 2207 (C≡N) cm^−1^.

#### 3.2.38. 3-Bromoindolo[1,2-*a*]quinoline-7-carbonitrile (**5bl**)

The reaction mixture was performed for 4 h, and the crude residue was purified by crystallization from hexane/dichloromethane to give a greenish yellow solid (123 mg, 38%); TLC: R*_f_* = 0.20 (dichloromethane/hexane = 1:1); m.p. 230–231 °C; ^1^H NMR (400 MHz, CDCl_3_) δ 8.51 (d, *J* = 9.0 Hz, 1H), 8.42–8.38 (m, 1H), 8.01–7.97 (m, 1H), 7.93 (d, *J* = 2.4 Hz, 1H), 7.81 (dd, *J* = 9.0, 2.4 Hz, 1H), 7.67 (d, *J* = 9.4 Hz, 1H), 7.58–7.52 (m, 2H), 7.44 (d, *J* = 9.4 Hz, 1H); ^13^C{^1^H} NMR (100 MHz, CDCl_3_) δ 141.3, 134.7, 132.7, 132.5, 131.8, 129.3, 127.3, 126.0, 124.6, 124.4, 120.4, 117.9, 117.6, 117.5, 115.7, 114.8, 80.8; HRMS (ESI) *m*/*z* [M+Na]^+^ calcd for C_17_H_9_BrN_2_Na^+^ 342.9841, found 342.9857; IR (neat) 2206 (C≡N) cm^−1^.

#### 3.2.39. 4-Bromoindolo[1,2-*a*]quinoline-7-carbonitrile (**5bm**)

The reaction mixture was performed for 1 h, and the crude residue was purified by crystallization from hexane/dichloromethane to give a greenish yellow solid (192 mg, 60%); TLC: R*_f_* = 0.30 (dichloromethane/hexane = 1:1); m.p. 246–247 °C; ^1^H NMR (400 MHz, CDCl_3_) δ 8.56 (d, *J* = 8.6 Hz, 1H), 8.40–8.36 (m, 1H), 7.97–7.93 (m, 2H), 7.72 (d, *J* = 7.8 Hz, 1H), 7.65 (d, *J* = 9.7 Hz, 1H), 7.56–7.49 (m, 3H); ^13^C{^1^H} NMR (100 MHz, CDCl_3_) δ 141.5, 137.2, 132.8, 130.3, 129.7, 129.1, 127.4, 124.7, 124.4, 123.8, 120.5, 118.2, 115.7, 115.6, 115.2, 80.8; HRMS (ESI) *m*/*z* [M+Na]^+^ calcd for C_17_H_9_BrN_2_Na^+^ 342.9841, found 342.9860; IR (neat) 2205 (C≡N) cm^−1^.

#### 3.2.40. 3-Iodoindolo[1,2-*a*]quinoline-7-carbonitrile (**5bn**)

The reaction mixture was performed for 3 h, and the crude residue was purified by flash column chromatography on silica gel to give a greenish yellow solid (134 mg, 36%); TLC: R*_f_* = 0.20 (Dichloromathane/hexane = 1:1); m.p. 257–258 °C; ^1^H NMR (400 MHz, CDCl_3_) δ 8.40–8.38 (m, 2H), 8.13 (d, *J* = 2.1 Hz, 1H), 8.01–7.97 (m, 2H), 7.66 (d, *J* = 9.4 Hz, 1H), 7.58–7.52 (m, 2H), 7.42 (d, *J* = 9.4 Hz, 1H); ^13^C{^1^H} NMR (100 MHz, CDCl_3_) δ 141.5, 138.5, 138.1, 135.5, 132.7, 129.5, 127.3, 126.5, 124.6, 124.4, 120.5, 118.0, 117.8, 115.8, 115.0, 88.2, 81.0; HRMS (ESI) *m*/*z* [M+Na]^+^ calcd for C_17_H_9_IN_2_Na^+^ 390.9703, found 390.9725; IR (neat) 2203 (C≡N) cm^−1^.

#### 3.2.41. 1-Methylindolo[1,2-*a*]quinoline-7-carbonitrile (**5bo**)

The reaction mixture was performed for 2.5 h, and the crude residue was purified by flash column chromatography on silica gel to give a greenish yellow solid (106 mg, 41%); TLC: R*_f_* = 0.20 (dichloromethane/hexane = 1:1); m.p. 150–151 °C; ^1^H NMR (400 MHz, CDCl_3_) δ 7.92 (dd, *J* = 7.6, 1.3 Hz, 1H), 7.63 (d, *J* = 8.4 Hz, 1H), 7.57 (dd, *J* = 7.5, 1.6 Hz, 1H), 7.54–7.49 (m, 2H), 7.47–7.37 (m, 4H), 2.50 (s, 3H); ^13^C{^1^H} NMR (100 MHz, CDCl_3_) δ 143.5, 135.1, 133.7, 133.3, 129.1, 128.9, 127.2, 126.5, 126.2, 125.3, 124.0, 122.4, 119.7, 116.5, 116.4, 116.3, 80.2, 22.4; HRMS (ESI) *m*/*z* [M+Na]^+^ calcd for C_18_H_12_N_2_Na^+^ 279.0893, found 279.0902; IR (neat) 2202 (C≡N) cm^−1^.

#### 3.2.42. 2-Methylindolo[1,2-*a*]quinoline-7-carbonitrile (**5bp**)

The reaction mixture was performed for 22 h, and the crude residue was purified by flash column chromatography on silica gel to give a greenish yellow solid (42 mg, 16%); TLC: R*_f_* = 0.20 (dichloromethane/hexane = 1:1); m.p. 195–196 °C; ^1^H NMR (400 MHz, CDCl_3_) δ 8.52–8.47 (m, 1H), 8.46 (s, 1H), 7.98–7.96 (m, 1H), 7.69 (d, *J* = 7.9 Hz, 1H), 7.59 (d, *J* = 9.3 Hz, 1H), 7.55–7.50 (m, 3H), 7.31 (dd, *J* = 8.0, 1.4 Hz, 1H), 2.66 (s, 3H); ^13^C{^1^H} NMR (100 MHz, CDCl_3_) δ 142.3, 141.0, 136.2, 132.8, 129.63, 129.60, 128.8, 126.0, 124.3, 123.7, 122.2, 120.2, 116.4, 116.3, 115.8, 115.2, 79.6, 22.6; HRMS (ESI) *m*/*z* [M+Na]^+^ calcd for C_18_H_12_N_2_Na^+^ 279.0893, found 279.0905; IR (neat) 2199 (C≡N) cm^−1^.

#### 3.2.43. 3-Methylindolo[1,2-*a*]quinoline-7-carbonitrile (**5bq**)

The reaction mixture was performed for 24 h, and the crude residue was purified by flash column chromatography on silica gel to give a greenish yellow solid (109 mg, 43%); TLC: R_f_ = 0.20 (dichloromethane/hexane = 1:1); m.p. 224–225 °C; ^1^H NMR (400 MHz, CDCl_3_) δ 8.52 (d, *J* = 8.6 Hz, 1H), 8.49–8.42 (m, 1H), 8.03–7.92 (m, 1H), 7.61 (d, *J* = 9.3 Hz, 1H), 7.58 (d, *J* = 2.1 Hz, 1H), 7.55–7.55 (m, 3H), 7.47 (d, *J* = 9.4 Hz, 1H), 2.52 (s, 3H); ^13^C{^1^H} NMR (100 MHz, CDCl_3_) δ 142.0, 134.5, 134.1, 132.7, 131.3, 129.7, 129.5, 128.8, 124.5, 124.2, 123.8, 120.2, 116.8, 116.3, 116.0, 115.1, 79.6, 21.0; HRMS (ESI) *m*/*z* [M+Na]^+^ calcd for C_18_H_12_N_2_Na^+^ 279.0893, found 279.0907; IR (neat) 2199 (C≡N) cm^−1^.

#### 3.2.44. 1-Methoxyindolo[1,2-*a*]quinoline-7-carbonitrile (**5br**)

The reaction mixture was performed for 2.5 h, and the crude residue was purified by flash column chromatography on silica gel to give a greenish yellow solid (73 mg, 27%); TLC: R*_f_* = 0.10 (dichloromethane/hexane = 1:1); m.p. 123–124 °C; ^1^H NMR (400 MHz, CDCl_3_) δ 8.02 (d, *J* = 8.7 Hz, 1H), 7.91 (d, *J* = 7.9 Hz, 1H), 7.62 (d, *J* = 9.3 Hz, 1H), 7.48–7.43 (m, 3H), 7.41–7.37 (m, 2H), 7.24 (dd, *J* = 7.9, 1.4 Hz, 1H), 3.99 (s, 4H); ^13^C{^1^H} NMR (100 MHz, CDCl_3_) δ 149.5, 142.9, 135.4, 129.2, 128.2, 127.3, 125.7, 125.1, 123.7, 122.2, 121.2, 119.1, 118.8, 117.4, 116.5, 112.4, 80.5, 55.8; HRMS (ESI) *m*/*z* [M+Na]^+^ calcd for C_18_H_12_N_2_NaO^+^ 295.0842, found 295.0849; IR (neat) 2204 (C≡N) cm^−1^.

#### 3.2.45. 2-Methoxyindolo[1,2-*a*]quinoline-7-carbonitrile (**5bs**)

The reaction mixture was performed for 3 h, and the crude residue was purified by crystallization from hexane/dichloromethane to give a greenish yellow solid (109 mg, 40%); TLC: Greenish yellow solid, yield 40%, R*_f_* = 0.20 (dichloromethane/hexane = 1:1), m.p. 204–205 °C; ^1^H NMR (400 MHz, CDCl_3_) δ 8.42 (d, *J* = 7.6 Hz, 1H), 8.10 (d, *J* = 2.3 Hz, 1H), 7.96 (dd, *J* = 6.9, 1.9 Hz, 1H), 7.71 (d, *J* = 8.7 Hz, 1H), 7.55–7.46 (m, 4H), 7.07 (dd, *J* = 8.7, 2.3 Hz, 1H), 4.04 (s, 3H); ^13^C{^1^H} NMR (100 MHz, CDCl_3_) δ 161.4, 142.6, 137.3, 132.6, 131.0, 129.8, 128.7, 124.4, 123.6, 120.1, 118.4, 116.4, 114.9, 114.1, 111.6, 101.5, 79.5, 56.0; HRMS (ESI) *m*/*z* [M+Na]^+^ calcd for C_18_H_12_N_2_NaO^+^ 295.0842, found 295.08457; IR (neat) 2206 (C≡N) cm^−1^.

#### 3.2.46. 3-Methoxyindolo[1,2-*a*]quinoline-7-carbonitrile (**5bt**)

The reaction mixture was performed for 22 h, and the crude residue was purified by flash column chromatography on silica gel to give a greenish yellow solid (103 mg, 38%); TLC: R*_f_* = 0.10 (dichloromethane/hexane = 1:1); m.p. 183–184 °C; ^1^H NMR (400 MHz, CDCl_3_) δ 8.57 (d, *J* = 9.3 Hz, 1H), 8.44–8.41 (m, 1H), 7.98–7.96 (m, 1H), 7.64 (d, *J* = 9.3 Hz, 1H), 7.54–7.46 (m, 3H), 7.31 (dd, *J* = 9.2, 3.0 Hz, 1H), 7.22 (d, *J* = 2.9 Hz, 1H), 3.95 (s, 3H); ^13^C{^1^H} NMR (100 MHz, CDCl_3_) δ 156.2, 141.4, 132.5, 130.5, 129.4, 128.5, 125.7, 124.1, 123.7, 120.2, 118.1, 117.4, 117.3, 116.4, 114.9, 111.7, 79.4, 55.9; HRMS (ESI) *m*/*z* [M+Na]^+^ calcd for C_18_H_12_N_2_NaO^+^ 295.0842, found 295.0857; IR (neat) 2199 (C≡N) cm^−1^.

#### 3.2.47. 4-Methoxyindolo[1,2-*a*]quinoline-7-carbonitrile (**5bu**)

The reaction mixture was performed for 2.5 h, and the crude residue was purified by crystallization from hexane/dichloromethane to give a greenish yellow solid (165 mg, 61%); TLC: R*_f_* = 0.20 (dichloromethane/hexane = 1:1); m.p. 211–212 °C; ^1^H NMR (400 MHz, CDCl_3_) δ 8.37 (d, *J* = 8.3 Hz, 1H), 8.12 (d, *J* = 8.6 Hz, 1H), 7.93–7.90 (m, 2H), 7.58 (t, *J* = 8.4 Hz, 1H), 7.51–7.43 (m, 3H), 6.87 (d, *J* = 8.2 Hz, 1H), 3.99 (s, 3H); ^13^C{^1^H} NMR (100 MHz, CDCl_3_) δ 156.8, 142.4, 136.9, 132.8, 130.7, 129.7, 124.3, 123.6, 123.0, 120.1, 116.4, 115.40, 115.38, 115.1, 108.7, 105.4, 79.4, 56.2; HRMS (ESI) *m*/*z* [M+Na]^+^ calcd for C_18_H_12_N_2_NaO^+^ 295.0842, found 295.0858; IR (neat) 2204 (C≡N) cm^−1^.

#### 3.2.48. 1-Fluoro-9-methoxyindolo[1,2-*a*]quinoline-7-carbonitrile (**5cb**)

The reaction mixture was performed for 2 h, and the crude residue was purified by crystallization from hexane/dichloromethane to give a greenish yellow solid (204 mg, 70%); TLC: R*_f_* = 0.30 (dichloromethane/hexane = 1:1); m.p. 185–186 °C; ^1^H NMR (400 MHz, CDCl_3_) δ 8.10 (dd, *J* = 9.5, 6.9 Hz, 1H), 7.64 (d, *J* = 9.3 Hz, 1H), 7.58–7.56 (m, 1H), 7.48–7.42 (m, 3H), 7.28 (d, *J* = 2.6 Hz, 1H), 7.07 (dd, *J* = 9.5, 2.7 Hz, 1H), 3.95 (s, 3H); ^13^C{^1^H} NMR (100 MHz, CDCl_3_) δ 157.3 (d, *J* = 1.9 Hz), 151.5 (d, *J* = 250.5 Hz), 142.3, 130.6, 129.3, 127.5 (d, *J* = 3.0 Hz), 127.2 (d, *J* = 3.0 Hz), 125.3 (d, *J* = 8.5 Hz), 124.9 (d, *J* = 3.2 Hz), 123.2 (d, *J* = 11.7 Hz), 118.0 (d, *J* = 32.0 Hz), 117.7 (d, *J* = 1.2 Hz), 116.9 (d, *J* = 23.1 Hz), 116.1, 114.4 (d, *J* = 4.8 Hz), 99.7, 81.4, 55.9; ^19^F NMR (470 MHz, CDCl_3_) δ −110.91 (dt, *J* = 13.2, 6.9 Hz); HRMS (ESI) *m*/*z* [M+Na]^+^ calcd for C_18_H_11_FN_2_NaO^+^ 313.0748, found 313.0756; IR (neat) 2204 (C≡N) cm^−1^.

#### 3.2.49. 2-Fluoro-9-methoxyindolo[1,2-*a*]quinoline-7-carbonitrile (**5cc**)

The reaction mixture was performed for 2.5 h, and the crude residue was purified by flash column chromatography on silica gel to give a greenish yellow solid (32 mg, 11%); TLC: R*_f_* = 0.20 (dichloromethane/hexane = 1:1); m.p. 195–196 °C; ^1^H NMR (400 MHz, CDCl_3_) δ 8.28–8.25 (m, 2H), 7.80 (dd, *J* = 8.7, 6.3 Hz, 1H), 7.59 (d, *J* = 9.3 Hz, 1H), 7.51 (d, *J* = 9.3 Hz, 1H), 7.34 (d, *J* = 2.6 Hz, 1H), 7.22 (td, *J* = 8.3, 2.3 Hz, 1H), 7.14 (dd, *J* = 9.3, 2.6 Hz, 1H), 3.97 (s, 3H); ^13^C{^1^H} NMR (100 MHz, CDCl_3_) δ 163.4 (d, *J* = 250.2 Hz), 157.3, 142.1, 136.5 (d, *J* = 11.0 Hz), 131.5 (d, *J* = 10.1 Hz), 131.1, 127.7 (d, *J* = 1.5 Hz), 127.4, 120.9 (d, *J* = 2.3 Hz), 116.1, 115.9 (d, *J* = 2.7 Hz), 115.8, 114.7, 112.7 (d, *J* = 22.9 Hz), 103.2 (d, *J* = 27.8 Hz), 100.9, 80.1, 55.9; ^19^F NMR (471 MHz, CDCl_3_) δ −106.46 (dt, *J* = 11.2, 7.1 Hz). HRMS (ESI) *m*/*z* [M+Na]^+^ calcd for C_18_H_11_FN_2_NaO^+^ 313.0748, found 313.0754; IR (neat) 2203 (C≡N) cm^−1^.

#### 3.2.50. 3-Fluoro-9-methoxyindolo[1,2-*a*]quinoline-7-carbonitrile (**5cd**)

The reaction mixture was performed for 20 h, and the crude residue was purified by crystallization from hexane/dichloromethane to give a greenish yellow solid (124 mg, 43%); TLC: R*_f_* = 0.30 (dichloromethane/hexane = 1:1); m.p. 228–229 °C; ^1^H NMR (400 MHz, CDCl_3_) δ 8.55 (dd, *J* = 9.2, 4.4 Hz, 1H), 8.28 (d, *J* = 9.3 Hz, 1H), 7.66 (d, *J* = 9.3 Hz, 1H), 7.49–7.42 (m, 3H), 7.32 (d, *J* = 2.6 Hz, 1H), 7.12 (dd, *J* = 9.3, 2.6 Hz, 1H), 3.96 (s, 3H); ^13^C{^1^H} NMR (100 MHz, CDCl_3_) δ 158.9 (d, *J* = 245.8 Hz), 157.2, 141.5, 132.3 (d, *J* = 2.2 Hz), 130.9, 127.4, 127.3 (d, *J* = 3.0 Hz), 125.9 (d, *J* = 8.5 Hz), 117.9, 117.6 (d, *J* = 23.9 Hz), 117.4 (d, *J* = 8.2 Hz), 116.2, 115.8, 114.9, 114.8 (d, *J* = 22.4 Hz), 100.7, 80.0, 55.9; ^19^F NMR (470 MHz, CDCl_3_) δ −116.96 (td, *J* = 7.9, 4.3 Hz); HRMS (ESI) *m*/*z* [M+Na]^+^ calcd for C_18_H_11_FN_2_NaO^+^ 313.0748, found 313.0757; IR (neat) 2203 (C≡N) cm^−1^.

#### 3.2.51. 4-Fluoro-9-methoxyindolo[1,2-*a*]quinoline-7-carbonitrile (**5ce**)

The reaction mixture was performed for 1 h, and the crude residue was purified by crystallization from hexane/dichloromethane to give a greenish yellow solid (129 mg, 44%); TLC: R*_f_* = 0.30 (dichloromethane/hexane = 1:1); m.p. 218–219 °C; ^1^H NMR (400 MHz, CDCl_3_) δ 8.26 (d, *J* = 8.7 Hz, 1H), 8.21 (d, *J* = 9.3 Hz, 1H), 7.72 (d, *J* = 9.5 Hz, 1H), 7.64 (td, *J* = 8.4, 6.2 Hz, 1H), 7.56 (d, *J* = 9.5 Hz, 1H), 7.23 (d, *J* = 2.6 Hz, 1H), 7.17 (t, *J* = 8.7 Hz, 1H), 7.07 (dd, *J* = 9.3, 2.6 Hz, 1H), 3.93 (s, 3H); ^13^C{^1^H} NMR (100 MHz, CDCl_3_) δ 159.4 (d, *J* = 252.7 Hz), 157.2, 141.7, 136.4 (d, *J* = 5.5 Hz), 130.9, 130.4 (d, *J* = 9.8 Hz), 127.4, 120.1 (d, *J* = 7.3 Hz), 116.9 (d, *J* = 2.0 Hz), 116.04, 115.97, 114.9, 114.2 (d, *J* = 18.5 Hz), 111.6 (d, *J* = 3.7 Hz), 110.2 (d, *J* = 20.8 Hz), 100.6, 80.5, 55.9; ^19^F NMR (470 MHz, CDCl_3_) δ -116.89 (dd, *J* = 9.2, 6.4 Hz); HRMS (ESI) *m*/*z* [M+Na]^+^ calcd for C_18_H_11_FN_2_NaO^+^ 313.0748, found 313.0759; IR (neat) 2207 (C≡N) cm^−1^.

#### 3.2.52. 1-Chloro-9-methoxyindolo[1,2-*a*]quinoline-7-carbonitrile (**5cf**)

The reaction mixture was performed for 1.5 h, and the crude residue was purified by flash column chromatography on silica gel to give a greenish yellow solid (101 mg, 33%); TLC: R*_f_* = 0.30 (dichloromethane/hexane = 1:1); m.p. 180–181 °C; ^1^H NMR (400 MHz, CDCl_3_) δ 7.87 (d, *J* = 9.3 Hz, 1H), 7.69 (dd, *J* = 7.6, 1.5 Hz, 1H), 7.68 (dd, *J* = 8.0, 1.5 Hz, 1H), 7.64 (d, *J* = 9.2 Hz, 1H), 7.45 (t, *J* = 7.8 Hz, 1H), 7.43 (d, *J* = 9.2 Hz, 1H), 7.30 (d, *J* = 2.6 Hz, 1H), 7.05 (dd, *J* = 9.3, 2.6 Hz, 1H), 3.96 (s, 3H); ^13^C{^1^H} NMR (100 MHz, CDCl_3_) δ 157.2, 143.1, 132.1, 131.9, 130.4, 129.5, 128.0, 127.3, 126.9, 125.7, 122.7, 120.1, 117.5, 116.1, 113.1, 99.7, 81.4, 55.9; HRMS (ESI) *m*/*z* [M+Na]^+^ calcd for C_18_H_11_ClN_2_NaO^+^ 329.0452, found 329.0458; IR (neat) 2210 (C≡N) cm^−1^.

#### 3.2.53. 2-Chloro-9-methoxyindolo[1,2-*a*]quinoline-7-carbonitrile (**5cg**)

The reaction mixture was performed for 4 h, and the crude residue was purified by crystallization from hexane/dichloromethane to give a greenish yellow solid (186 mg, 61%); TLC: R*_f_* = 0.30 (dichloromethane/hexane = 1:1); m.p. 227–228 °C; ^1^H NMR (400 MHz, CDCl_3_) δ 8.41 (s, 1H), 8.15 (d, *J* = 9.3 Hz, 1H), 7.68 (d, *J* = 8.3 Hz, 1H), 7.53 (d, *J* = 9.3 Hz, 1H, 7.42 (d, *J* = 9.4 Hz, 1H), 7.40 (dd, *J* = 8.3, 1.8 Hz, 1H), 7.26 (d, *J* = 2.5 Hz, 1H), 7.10 (dd, *J* = 9.3, 2.6 Hz, 1H), 3.95 (s, 3H); ^13^C{^1^H} NMR (100 MHz, CDCl_3_) δ 157.3, 141.8, 136.1, 135.9, 131.0, 130.6, 127.4, 127.3, 125.0, 122.7, 116.8, 116.0, 115.9, 115.8, 114.9, 100.8, 80.3, 55.9; HRMS (ESI) *m*/*z* [M+Na]^+^ calcd for C_18_H_11_ClN_2_NaO^+^ 329.0452, found 329.0466; IR (neat) 2208 (C≡N) cm^−1^.

#### 3.2.54. 3-Chloro-9-methoxyindolo[1,2-*a*]quinoline-7-carbonitrile (**5ch**)

The reaction mixture was performed for 3 h, and the crude residue was purified by flash column chromatography on silica gel to give a greenish yellow solid (90 mg, 29%); R*_f_* = 0.30 (dichloromethane/hexane = 1:1); m.p. 243–244 °C; ^1^H NMR (400 MHz, CDCl_3_) δ 8.42 (d, *J* = 9.0 Hz, 1H), 8.20 (d, *J* = 9.3 Hz, 1H), 7.73 (d, *J* = 2.4 Hz, 1H), 7.64–7.59 (m, 2H), 7.39 (d, *J* = 9.3 Hz, 1H), 7.28 (d, *J* = 2.6 Hz, 1H), 7.10 (dd, *J* = 9.3, 2.6 Hz, 1H), 3.95 (s, 3H); ^13^C{^1^H} NMR (100 MHz, CDCl_3_) δ 157.3, 141.6, 134.2, 131.0, 130.0, 129.9, 128.8, 127.5, 127.0, 125.7, 117.9, 117.1, 116.0, 115.9, 115.0, 100.8, 80.4, 55.9; HRMS (ESI) *m*/*z* [M+Na]^+^ calcd for C_18_H_11_ClN_2_NaO^+^ 329.0452, found 329.0468; IR (neat) 2201 (C≡N) cm^−1^.

#### 3.2.55. 4-Chloro-9-methoxyindolo[1,2-*a*]quinoline-7-carbonitrile (**5ci**)

The reaction mixture was performed for 1 h, and the crude residue was purified by crystallization from hexane/dichloromethane to give a greenish yellow solid (170 mg, 55%); TLC: R*_f_* = 0.30 (dichloromethane/hexane = 1:1); m.p. 252–253 °C; ^1^H NMR (400 MHz, CDCl_3_) δ 8.51 (d, *J* = 8.6 Hz, 1H), 8.31 (d, *J* = 9.3 Hz, 1H), 8.00 (dd, *J* = 9.6, 0.8 Hz, 1H), 7.70 (d, *J* = 9.7 Hz, 1H), 7.63 (t, *J* = 8.2 Hz, 1H), 7.54 (dd, *J* = 7.9, 1.0 Hz, 1H), 7.33 (d, *J* = 2.6 Hz, 1H), 7.13 (dd, *J* = 9.3, 2.6 Hz, 1H), 3.96 (s, 3H); ^13^C{^1^H} NMR (100 MHz, CDCl_3_) δ 157.3, 141.6, 136.8, 133.9, 131.2, 129.9, 127.6, 125.4, 124.1, 122.4, 117.7, 116.2, 116.0, 115.0, 114.6, 100.8, 80.4, 55.9; HRMS (ESI) *m*/*z* [M+Na]^+^ calcd for C_18_H_11_ClN_2_NaO^+^ 329.0452, found 329.0468; IR (neat) 2205 (C≡N) cm^−1^.

#### 3.2.56. 2-Bromo-9-methoxyindolo[1,2-*a*]quinoline-7-carbonitrile (**5ck**)

The reaction mixture was performed for 7 h, and the crude residue was purified by flash column chromatography on silica gel to give a greenish yellow solid (40 mg, 11%); TLC: R*_f_* = 0.30 (dichloromethane/hexane = 1:1); m.p. 232–233 °C; ^1^H NMR (400 MHz, CDCl_3_) δ 8.67 (t, *J* = 1.1 Hz, 1H), 8.23 (d, *J* = 9.3 Hz, 1H), 7.64 (d, *J* = 8.4 Hz, 1H), 7.61 (d, *J* = 9.3 Hz, 1H), 7.57 (dd, *J* = 8.4, 1.7 Hz, 1H), 7.45 (d, *J* = 9.3 Hz, 1H), 7.32 (d, *J* = 2.6 Hz, 1H), 7.15 (dd, *J* = 9.3, 2.6 Hz, 1H), 3.96 (s, 3H); ^13^C{^1^H} NMR (100 MHz, CDCl_3_) δ 157.3, 141.9, 136.3, 131.0, 130.8, 127.8, 127.5, 127.4, 124.0, 123.1, 118.9, 117.1, 116.0, 115.9, 115.0, 100.9, 80.4, 55.9; HRMS (ESI) *m*/*z* [M+Na]^+^ calcd for C_18_H_11_BrN_2_NaO^+^ 372.9947, found 372.9958; IR (neat) 2207 (C≡N) cm^−1^.

#### 3.2.57. 4-Bromo-9-methoxyindolo[1,2-*a*]quinoline-7-carbonitrile (**5cm**)

The reaction mixture was performed for 1 h, and the crude residue was purified by crystallization from hexane/dichloromethane to give a greenish yellow solid (265 mg, 75%); TLC: R*_f_* = 0.30 (dichloromethane/hexane = 1:1); m.p. 254–255 °C; ^1^H NMR (400 MHz, CDCl_3_) δ 8.55 (d, *J* = 8.6 Hz, 1H), 8.31 (d, *J* = 9.3 Hz, 1H), 7.98 (dd, *J* = 9.7, 0.8 Hz, 1H), 7.74 (dd, *J* = 7.9, 0.9 Hz, 1H), 7.68 (d, *J* = 9.7 Hz, 1H), 7.56 (t, *J* = 8.2 Hz, 1H), 7.33 (d, *J* = 2.6 Hz, 1H), 7.13 (dd, *J* = 9.3, 2.6 Hz, 1H), 3.96 (s, 3H); ^13^C{^1^H} NMR (100 MHz, CDCl_3_) δ 157.3, 141.6, 136.8, 131.3, 130.2, 128.9, 127.6, 126.9, 124.4, 123.6, 118.0, 116.2, 116.0, 115.3, 115.0, 100.8, 80.3, 55.9; HRMS (ESI) *m*/*z* [M+Na]^+^ calcd for C_18_H_11_BrN_2_NaO^+^ 372.9947, found 372.9966; IR (neat) 2204 (C≡N) cm^−1^.

#### 3.2.58. 9-Methoxy-1-methylindolo[1,2-*a*]quinoline-7-carbonitrile (**5co**)

The reaction mixture was performed for 2.5 h, and the crude residue was purified by flash column chromatography on silica gel to give a greenish yellow solid (230 mg, 80%); TLC: R*_f_* = 0.20 (dichloromethane/hexane = 1:1); m.p. 173–174 °C; ^1^H NMR (400 MHz, CDCl_3_) δ 7.60 (dd, *J* = 7.6, 1.6 Hz, 1H), 7.572 (d, *J* = 9.2 Hz, 1H), 7.568 (d, *J* = 9.2 Hz, 1H), 7.51 (dd, *J* = 7.5, 1.6 Hz, 1H), 7.45 (d, *J* = 9.2 Hz, 1H), 7.44 (t, *J* = 7.5 Hz, 1H), 7.30 (d, *J* = 2.5 Hz, 1H), 7.02 (dd, *J* = 9.2, 2.5 Hz, 1H), 3.95 (s, 3H), 2.54 (s, 3H); ^13^C{^1^H} NMR (100 MHz, CDCl_3_) δ 157.1, 143.5, 133.6, 133.2, 130.4, 130.0, 128.4, 126.9, 126.4, 126.2, 125.1, 117.6, 116.6, 116.1, 113.1, 100.0, 79.8, 55.9, 22.2; HRMS (ESI) *m*/*z* [M+Na]^+^ calcd for C_19_H_14_N_2_NaO^+^ 309.0998, found 309.1009; IR (neat) 2213 (C≡N) cm^−1^.

#### 3.2.59. 9-Methoxy-3-methylindolo[1,2-*a*]quinoline-7-carbonitrile (**5cq**)

The reaction mixture was performed for 24 h, and the crude residue was purified by flash column chromatography on silica gel to give a greenish yellow solid (110 mg, 38%); TLC: R*_f_* = 0.20 (dichloromethane/hexane = 1:1), m.p. 189–190 °C; ^1^H NMR (400 MHz, CDCl_3_) δ 8.36 (d, *J* = 8.6 Hz, 1H), 8.23 (d, *J* = 9.3 Hz, 1H), 7.53–7.50 (m, 2H), 7.47 (dd, *J* = 8.7, 2.1 Hz, 1H), 7.39 (d, *J* = 9.3 Hz, 1H), 7.26 (m, 1H), 7.05 (dd, *J* = 9.3, 2.6 Hz, 1H), 3.94 (s, 3H), 2.49 (s, 3H); ^13^C{^1^H} NMR (100 MHz, CDCl_3_) δ 157.0, 142.0, 134.3, 133.8, 131.2, 131.0, 129.6, 128.2, 127.5, 124.3, 116.6, 116.5, 116.0, 115.6, 114.3, 100.6, 79.1, 55.9, 21.0; HRMS (ESI) *m*/*z* [M+Na]^+^ calcd for C_19_H_14_N_2_NaO^+^ 309.0998, found 309.1005; IR (neat) 2204 (C≡N) cm^−1^.

#### 3.2.60. 1,9-Dimethoxyindolo[1,2-*a*]quinoline-7-carbonitrile (**5cr**)

The reaction mixture was performed for 2.5 h, and the crude residue was purified by flash column chromatography on silica gel to give a greenish yellow solid (41 mg, 14%); TLC: R*_f_* = 0.20 (dichloromethane/hexane = 1:1); m.p. 170–171 °C; ^1^H NMR (400 MHz, CDCl_3_) δ 7.90 (d, *J* = 9.4 Hz, 1H), 7.54 (d, *J* = 9.2 Hz, 0H), 7.41 (t, *J* = 7.8 Hz, 1H), 7.37 (dd, *J* = 9.2, 1.1 Hz, 1H), 7.33 (dd, *J* = 7.8, 1.4 Hz, 1H), 7.23 (d, *J* = 2.6 Hz, 1H), 7.19 (dd, *J* = 8.0, 1.4 Hz, 1H), 6.98 (dd, *J* = 9.4, 2.6 Hz, 1H), 3.95 (s, 3H), 3.94 (s, 3H); ^13^C{^1^H} NMR (100 MHz, CDCl_3_) δ 156.8, 149.4, 143.0, 130.5, 130.4, 127.7, 127.2, 125.5, 125.2, 121.2, 120.0, 117.1, 116.7, 113.0, 112.3, 99.2, 80.2, 55.94, 55.87; HRMS (ESI) *m*/*z* [M+Na]^+^ calcd for C_19_H_14_N_2_NaO_2_^+^ 325.0947, found 325.0956; IR (neat) 2198 (C≡N) cm^−1^.

#### 3.2.61. 2,9-Dimethoxyindolo[1,2-*a*]quinoline-7-carbonitrile (**5cs**)

The reaction mixture was performed for 3.5 h, and the crude residue was purified by flash column chromatography on silica gel to give a greenish yellow solid (264 mg, 87%); TLC: R*_f_* = 0.20 (dichloromethane/hexane = 1:1); m.p. 182–183 °C; ^1^H NMR (400 MHz, CDCl_3_) δ 8.24 (d, *J* = 9.3 Hz, 1H), 7.98 (d, *J* = 2.3 Hz, 1H), 7.70 (d, *J* = 8.7 Hz, 1H), 7.45 (app s, 2H), 7.29 (d, *J* = 2.6 Hz, 1H), 7.09–7.04 (m, 2H), 4.02 (s, 3H), 3.95 (s, 3H); ^13^C{^1^H} NMR (100 MHz, CDCl_3_) δ 161.3, 157.1, 142.7, 137.0, 131.2, 131.0, 128.2, 127.4, 118.3, 116.6, 115.9, 114.0, 113.9, 111.7, 100.9, 100.7, 79.0, 56.0, 55.9; HRMS (ESI) *m*/*z* [M+Na]^+^ calcd for C_19_H_14_N_2_NaO_2_^+^ 325.0947, found 325.0956; IR (neat) 2199 (C≡N) cm^−1^.

#### 3.2.62. 3,9-Dimethoxyindolo[1,2-*a*]quinoline-7-carbonitrile (**5ct**)

The reaction mixture was performed for 4 h, and the crude residue was purified by flash column chromatography on silica gel to give a greenish yellow solid (132 mg, 44%); TLC: R*_f_* = 0.30 (dichloromethane/hexane = 1:1); m.p. 235–236 °C; ^1^H NMR (400 MHz, CDCl_3_) δ 8.50 (d, *J* = 9.3 Hz, 1H), 8.29 (d, *J* = 9.3 Hz, 1H), 7.62 (d, *J* = 9.3 Hz, 1H), 7.47 (d, *J* = 9.3 Hz, 1H), 7.32–7.29 (m, 2H), 7.22 (d, *J* = 2.9 Hz, 1H), 7.09 (dd, *J* = 9.3, 2.6 Hz, 1H), 3.96 (s, 3H), 3.95 (s, 3H); ^13^C{^1^H} NMR (100 MHz, CDCl_3_) δ 157.0, 156.1, 141.5, 131.0, 130.3, 128.0, 127.4, 125.6, 118.3, 117.13, 117.09, 116.7, 115.9, 114.4, 111.5, 100.6, 78.9, 55.88, 55.87; HRMS (ESI) *m*/*z* [M+Na]^+^ calcd for C_19_H_14_N_2_NaO_2_^+^ 325.0947, found 325.0954; IR (neat) 2199 (C≡N) cm^−1^.

#### 3.2.63. 4,9-Dimethoxyindolo[1,2-*a*]quinoline-7-carbonitrile (**5cu**)

The reaction mixture was performed for 4 h, and the crude residue was purified by crystallization from hexane/dichloromethane to give a greenish yellow solid (237 mg, 78%); TLC: R*_f_* = 0.20 (dichloromethane/hexane = 1:1); m.p. 240–241 °C; ^1^H NMR (400 MHz, CDCl_3_) δ 8.28 (d, *J* = 9.3 Hz, 1H), 8.11 (d, *J* = 8.6 Hz, 1H), 7.96 (d, *J* = 9.6 Hz, 1H), 7.61 (t, *J* = 8.4 Hz, 1H), 7.52 (d, *J* = 9.6 Hz, 1H), 7.28 (d, *J* = 2.6 Hz, 1H), 7.06 (dd, *J* = 9.3, 2.6 Hz, 1H), 6.89 (d, *J* = 8.2 Hz, 1H), 4.01 (s, 3H), 3.95 (s, 3H); ^13^C{^1^H} NMR (100 MHz, CDCl_3_) δ 157.1, 156.8, 142.5, 136.6, 131.2, 130.6, 127.6, 122.5, 116.6, 116.3, 115.2, 115.1, 114.1, 108.4, 105.1, 100.5, 79.0, 56.2, 55.9; HRMS (ESI) *m*/*z* [M+Na]^+^ calcd for C_19_H_14_N_2_NaO_2_^+^ 325.0947, found 325.0956; IR (neat) 2204 (C≡N) cm^−1^.

### 3.3. Biological Assays and Anticancer Evaluation Protocols

#### 3.3.1. Primary Screening

All test compounds were dissolved in DMSO (Merck, Darmstadt, Germany) to prepare 50 mM stock solutions. Human cancer cells (HCT-116 cells, HT-29, or MCF-7; ATCC, Manassas, VA, USA) were seeded in 96-well plates at a density of 2 × 10^3^ cells/well. HCT 116 and MCF-7 cells were cultured in DMEM high-glucose medium (Cytiva, Marlborough, MA, USA) supplemented with 10% FBS (Merck, Darmstadt, Germany), 1X HEPES (Cytiva, Marlborough, MA, USA), and 1% penicillin/streptomycin (Thermo Fisher Scientific, Wilmington, DE, USA), whereas HT-29 cells were cultured in DMEM/F-12 medium (Cytiva, Marlborough, MA, USA) supplemented with 10% FBS and 1% penicillin/streptomycin. The cells were incubated at 37 °C with 5% CO_2_ for 24 h. After incubation, the test compounds were added at a final concentration of 10 μM and further incubated for 72 h under the same conditions. Subsequently, the culture media were removed and replaced with serum-free media containing MTT (Abcam, Cambridge, UK), followed by an additional incubation at 37 °C with 5% CO_2_ for 3 h. The MTT-containing media were then removed, and DMSO was added to dissolve the resulting formazan crystals. The absorbance was measured at 570 nm using an EnVision Multi-Mode Microplate Reader (PerkinElmer, Waltham, MA, USA) [49,50,51].

##### Statistical Analysis

The percentage of cell viability was used to select compounds that inhibit cell proliferation by 80% or more (cell viability ≤ 20%) compared with the DMSO control. It was calculated using the following equation:

$$\%\;\mathrm{Cell}\;\mathrm{viability}=\frac{{\mathrm{OD}}_{\mathrm{Compound}}}{{\mathrm{OD}}_{\mathrm{DMSO}}}\times \;100$$

where *OD_compound_* and *OD_DMSO_* represent the optical density (absorbance) values of the test compound and the DMSO control, respectively.

The *Z′*-factor is a measure of statistical effect size used to quantify the suitability and quality of an assay for high-throughput screening. The *Z′*-factor is calculated using the following equation:

$$Z^{\prime }-\mathrm{factor}=1-\frac{3\left(\sigma_{P}+\sigma_{N}\right)}{\left|\mu_{P}-\mu_{N}\right|}$$

where *σ_P_* and *σ_N_* represent the standard deviations of the positive and negative controls, respectively, while *μ_P_* and *μ_N_* represent the mean values of the positive and negative controls, respectively.

#### 3.3.2. Evaluation of IC_50_ Values

The selected active compounds from the primary screening were evaluated for their half-maximal inhibitory concentration (IC_50_) values against HCT-116, HT-29, and MCF-7 cancer cell lines. Cells were seeded in 96-well plates at a density of 2 × 10^3^ cells/well. HCT 116 and MCF-7 cells were cultured in DMEM high-glucose medium supplemented with 10% FBS, 1X HEPES, and 1% penicillin/streptomycin, whereas HT-29 cells were cultured in DMEM/F-12 medium supplemented with 10% FBS and 1% penicillin/streptomycin. The cells were incubated at 37 °C with 5% CO_2_ for 24 h. After incubation, the test compounds were added at various concentrations (100, 10, 1, 0.1, 0.01, and 0.001 μM) and further incubated for 72 h under the same conditions. Subsequently, the culture media were removed and replaced with serum-free media containing MTT, followed by an additional incubation at 37 °C with 5% CO_2_ for 3 h. The MTT-containing media were then removed, and DMSO was added to dissolve the resulting formazan crystals. The absorbance was measured at 570 nm using an EnVision Multi-Mode Microplate Reader [49,50,51]. All assays were performed in two independent experiments (*N* = 2), each in triplicate (*n* = 3). Data are presented as mean ± SD. IC_50_ values were calculated using non-linear regression analysis (four-parameter logarithmic curve fitting) via GraphPad Prism software (Version 10). The complete dataset of IC_50_ values from individual independent runs with full statistical analyses, as well as representative concentration–response curves, are provided in the Supplementary Materials.

##### Statistical Analysis

The half-maximal inhibitory concentration (IC_50_) represents the concentration of a compound required to achieve 50% inhibition of cell viability. The IC_50_ values were determined using non-linear regression analysis according to the following equation:

$$Y=\mathrm{Bottom}+\;\frac{\mathrm{Top}-\mathrm{Bottom}}{1+10^{\left(\log{\mathrm{IC}}_{50}-X)(\mathrm{Hillslope}\right)}}$$

where *Y* is the percentage of cell viability, *X* is the logarithm of compound concentration, *Top* and *Bottom* are the maximal and minimal response plateaus, and *HillSlope* represents the curve steepness factor.

### 3.4. Computational Method

#### 3.4.1. Preparation of Ligands and Proteins

The 3D structures of the target proteins were obtained from the Protein Data Bank website. The downloaded proteins were prepared for molecular docking using ChimeraX software (Version 1.8), which was used to delete any water molecules and ligands present in the protein. The ligands were prepared for molecular docking by optimizing their structures to the mol2 file format using Avogadro software (Version 1.2.0) [52].

#### 3.4.2. Molecular Docking

Molecular docking calculations were performed using AutoDock 4.2.6. Protein PDB files were converted to PDBQT format by adding polar hydrogens and assigning Kollman charges. The grid box was set to cover the entire protein with 40 × 40 × 40 grid points and a grid spacing of 0.375 Å. The specific grid center coordinates (*x*, *y*, *z*) for each target protein were set to (−8.615, −49.545, 12.689) for CDK2 (PDB ID: 1DI8), (13.654, 27.991, 9.706) for CDK6 (PDB ID: 5L2I), and (22.000, 0.300, 52.800) for EGFR (PDB ID: 1M17). Conformational searching was executed using the Lamarckian Genetic Algorithm (LGA) with 40 independent GA runs. The resulting docking poses were subsequently analyzed using BIOVIA Discovery Studio Visualizer 2024 to evaluate predicted binding energies and interaction profiles [53,54,55].

## 4. Conclusions

A series of fifty-four novel indolo[1,2-*a*]quinoline-7-carbonitrile derivatives was successfully synthesized via a cascade reaction involving nucleophilic aromatic substitution and Knoevenagel condensation, affording the desired products in moderate to good yields. Biological evaluation against human cancer cell lines identified several potent antiproliferative agents. Specifically, compounds **5at**, **5bc**, **5bk**, **5cd**, and **5ch** exhibited significant cytotoxicity against colorectal cancer HCT-116 cells, with **5ch** emerging as the most potent derivative (IC_50_ = 2.16 ± 1.37 µM). Against colorectal cancer HT-29 cells, eight compounds (**5bc**, **5bd**, **5bk**, **5ca**, **5cd**, **5ch**, **5ck**, and **5cs**) displayed potent activity, with **5cs** showing the highest efficacy (IC_50_ = 2.27 ± 1.21 µM). Furthermore, compounds **5bh**, **5bk**, and **5bl** were active against breast cancer MCF-7 cells, with **5bl** being the most effective (IC_50_ = 3.84 µM, SD < 0.01 µM). Notably, compound **5bk** demonstrated broad-spectrum cytotoxicity across all three tested cell lines (IC_50_ = 3.64–6.06 µM), highlighting its potential as a versatile lead candidate for anticancer drug discovery.

Molecular docking simulations provided supportive in silico insights, revealing favorable predicted binding affinities of active derivatives within the catalytic pockets of target enzymes (CDK2 for HCT-116, CDK6 for HT-29, and EGFR for MCF-7). The binding interactions were primarily stabilized by hydrogen bonding via the C7-cyano substituent, hydrophobic π-π stacking of the rigid indolo[1,2-*a*]quinoline core, and key halogen contacts mediated by the bromine atom in compound **5bk**. Overall, these combined in vitro antiproliferative data and complementary in silico observations suggest that the indolo[1,2-*a*]quinoline framework represents a promising pharmacophore for further medicinal chemistry optimization and targeted biochemical target-validation studies.

## Figures and Tables

**Table 1:** Preparation of indolo[1,2-*a*]quinoline-7-carbonitrile derivatives ^a^.

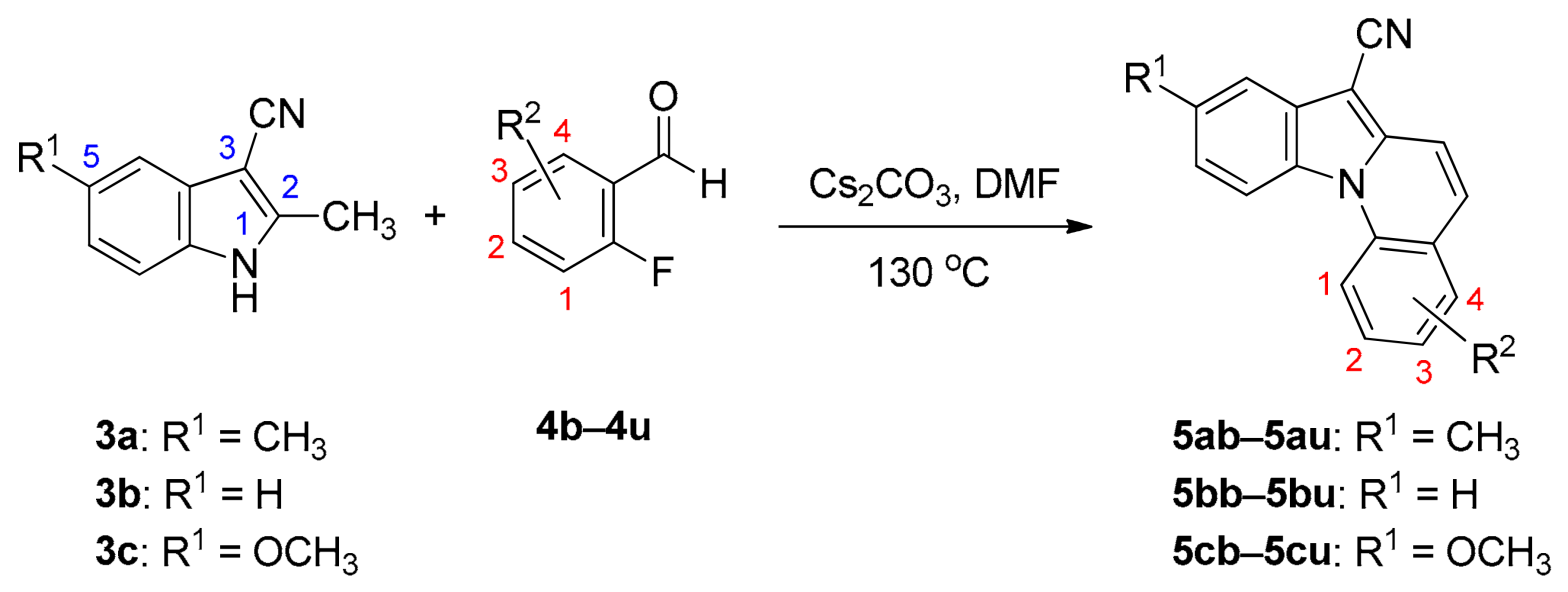						
**Entry**	**Indole**	**Benzaldehyde**	**R^2^**	**Time (h) ^b^**	**Product**	**Yield (%)**
1	**3a**	**4b**	1-F	0.5	**5ab**	40 ^d^
2	**3a**	**4c**	2-F	3.5	**5ac**	25 ^e^
3	**3a**	**4d**	3-F	18	**5ad**	ND
4	**3a**	**4e**	4-F	0.5	**5ae**	65 ^d^
5	**3a**	**4f**	1-Cl	1	**5af**	27 ^d^
6	**3a**	**4g**	2-Cl	24	**5ag**	17 ^e^
7	**3a**	**4h**	3-Cl	24	**5ah**	10 ^e^
8	**3a**	**4i**	4-Cl	0.5	**5ai**	74 ^d^
9	**3a**	**4j**	1-Br	3	**5aj**	13 ^d^
10	**3a**	**4k**	2-Br	2	**5ak**	26 ^d^
11	**3a**	**4l**	3-Br	1	**5al**	52 ^d^
12	**3a**	**4m**	4-Br	1.5	**5am**	77 ^d^
13	**3a**	**4n**	3-I	3	**5an**	38 ^e^
14	**3a**	**4o**	1-CH_3_	3.5	**5ao**	22 ^e^
15	**3a**	**4p**	2-CH_3_	24	**5ap**	14 ^e^
16	**3a**	**4q**	3-CH_3_	5.5	**5aq**	18 ^d^
17	**3a**	**4r**	1-OCH_3_	24 ^c^	**5ar**	34 ^d^
18	**3a**	**4s**	2-OCH_3_	2 ^c^	**5as**	64 ^d^
19	**3a**	**4t**	3-OCH_3_	24 ^c^	**5at**	66 ^d^
20	**3a**	**4u**	4-OCH_3_	0.5	**5au**	83 ^d^
21	**3b**	**4b**	1-F	2.5	**5bb**	50 ^e^
22	**3b**	**4c**	2-F	3.5	**5bc**	18 ^e^
23	**3b**	**4d**	3-F	4	**5bd**	37 ^d^
24	**3b**	**4e**	4-F	3	**5be**	38 ^d^
25	**3b**	**4f**	1-Cl	3	**5bf**	45 ^e^
26	**3b**	**4g**	2-Cl	4	**5bg**	16 ^d^
27	**3b**	**4h**	3-Cl	5	**5bh**	43 ^e^
28	**3b**	**4i**	4-Cl	3	**5bi**	76 ^d^
29	**3b**	**4j**	1-Br	3.5	**5bj**	ND
30	**3b**	**4k**	2-Br	4	**5bk**	19 ^e^
31	**3b**	**4l**	3-Br	4	**5bl**	38 ^d^
32	**3b**	**4m**	4-Br	1	**5bm**	60 ^d^
33	**3b**	**4n**	3-I	3	**5bn**	36 ^e^
34	**3b**	**4o**	1-CH_3_	2.5	**5bo**	41 ^e^
35	**3b**	**4p**	2-CH_3_	22	**5bp**	16 ^e^
36	**3b**	**4q**	3-CH_3_	24 ^c^	**5bq**	43 ^e^
37	**3b**	**4r**	1-OCH_3_	2.5	**5br**	27 ^e^
38	**3b**	**4s**	2-OCH_3_	3	**5bs**	40 ^d^
39	**3b**	**4t**	3-OCH_3_	22	**5bt**	38 ^e^
40	**3b**	**4u**	4-OCH_3_	2.5	**5bu**	61 ^d^
41	**3c**	**4b**	1-F	2	**5cb**	70 ^d^
42	**3c**	**4c**	2-F	2.5	**5cc**	11 ^e^
43	**3c**	**4d**	3-F	20	**5cd**	43 ^d^
44	**3c**	**4e**	4-F	1	**5ce**	44 ^d^
45	**3c**	**4f**	1-Cl	1.5	**5cf**	33 ^e^
46	**3c**	**4g**	2-Cl	4	**5cg**	61 ^d^
47	**3c**	**4h**	3-Cl	3	**5ch**	29 ^e^
48	**3c**	**4i**	4-Cl	1	**5ci**	55 ^d^
49	**3c**	**4j**	1-Br	1.5	**5cj**	ND
50	**3c**	**4k**	2-Br	7	**5ck**	11 ^e^
51	**3c**	**4l**	3-Br	3	**5cl**	ND
52	**3c**	**4m**	4-Br	1	**5cm**	75 ^d^
53	**3c**	**4n**	3-I	2.5	**5cn**	ND
54	**3c**	**4o**	1-CH_3_	2.5	**5co**	80 ^e^
55	**3c**	**4p**	2-CH_3_	24	**5cp**	ND
56	**3c**	**4q**	3-CH_3_	24	**5cq**	38 ^e^
57	**3c**	**4r**	1-OCH_3_	2.5	**5cr**	14 ^e^
58	**3c**	**4s**	2-OCH_3_	3.5	**5cs**	87 ^e^
59	**3c**	**4t**	3-OCH_3_	4	**5ct**	44 ^e^
60	**3c**	**4u**	4-OCH_3_	4	**5cu**	78 ^d^

^a^ Reaction conditions: **3a**, **3b** or **3c** (1.0 mmol), **4b**–**4u** (1.6 mmol), Cs_2_CO_3_ (3.0 mmol), DMF (3 mL). ^b^ The reaction was terminated after the indole substrate was completely consumed. ^c^ The indole substrate was not completely consumed. ^d^ Isolated by recrystallization. ^e^ Isolated by flash column chromatography (ND = % yield not determined due to purification issues).

**Table 2:** Cytotoxic activity against human colorectal (HCT-116 and HT-29) and breast (MCF-7) cancer cells (Data are presented as mean ± SD; *N* = 2).

Entry	Compound	IC_50_ (µM)		
		HCT-116	HT-29	MCF-7
1	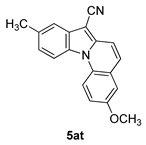	4.62 ± 2.12	-	-
2	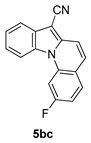	4.95 ± 0.27	3.93 ± 1.00	-
3	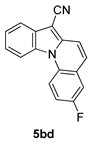	-	4.39 ± 1.79	-
4	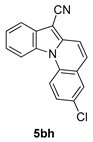	-	-	5.68 ± 1.62
5	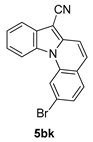	3.64 ± 0.72	5.29 ± 1.83	6.06 ± 0.16
6	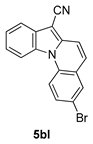	-	-	3.84, SD < 0.01
7	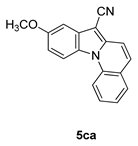	-	8.18 ± 1.59	-
8	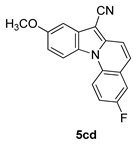	3.51 ± 1.55	5.44 ± 2.96	-
9	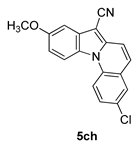	2.16 ± 1.37	2.40 ± 1.03	-
10	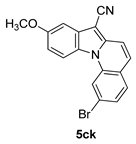	-	4.13 ± 0.91	-
11	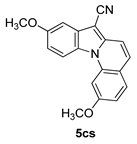	-	2.27 ± 1.21	-

**Table 3:** Molecular docking results of compounds **5a**, **5at**, **5bc**, **5bk**, **5cd**, **5ch**, and doxorubicin within the active site of cyclin-dependent kinase 2 (CDK2) (PDB ID: 1DI8) ^a^ [43].

Compound	Binding Energy (kcal/mol)	Interaction			
		H-Bond		Hydrophobic	Others
		Number	Residue		
**5a**	−8.67	1	ASP:145	VAL:18, ALA:144, ILE:10, ALA:31, VAL:64, LEU:134	-
**5at**	−8.58	2	ASP:145, LEU:83	ALA:144, LEU:134, ALA:31, PHE:82, VAL:18, VAL:64	-
**5bc**	−8.06	1	ASP:145	ALA:144, LEU:134, VAL:64, ALA:31, VAL:18	-
**5bk**	−8.79	1	ASP:145	VAL:64, LEU:134, ALA:31, PHE:82, ILE:10, ALA:144	-
**5cd**	−8.33	4	LYS:33, ASP:145, LEU:83, ASN:132	VAL:18, ILE:10. PHE:82, ALA:31, PHE:80, LEU:134, VAL:64, ALA:144	Halogen (fluorine) interaction (LEU:83)
**5ch**	−8.39	1	LEU:83	ILE:10, VAL:18, PHE:80, ALA:144, LEU:148, LYS:33, ALA:31, VAL:64, LEU:134	-
doxorubicin	−8.83	4	LEU:83, ASP:145, ASN:132, GLU:12	LYS:89, LEU:134, PHE:82, ILE:10, VAL:18, ALA:31	-

^a^ RMSD of the native ligand (4-(3-hydroxyanilino)-6,7-dimethoxyquinazoline, DTQ) = 1.43 Å.

**Table 4:** Molecular docking results of compounds **5bc**, **5bd**, **5bk**, **5ca**, **5cd**, **5ch**, **5ck**, **5cs**, and doxorubicin within the active site of cyclin-dependent kinase 6 (CDK6) (PDB ID: 5L2I) ^a^ [46].

Compound	Binding Energy (kcal/mol)	Interaction			
		H-Bond		Hydrophobic	Others
		Number	Residue		
**5bc**	−7.77	2	VAL:101, HIS:100	LEU:152, ILE:19, VAL:27	Halogen (fluorine) interaction (GLN:149)
**5bd**	−7.85	2	VAL:101, HIS:101	ILE:19, LEU:152, ALA:41, PHE:98, VAL:77, ALA:162	Halogen (fluorine) interaction (GLN:149)
**5bk**	−9.49	1	LYS:43	VAL:27, ILE:19, LEU:152, ALA:41, VAL:77, PHE:98	-
**5ca**	−7.81	1	ASP:163, VAL:101, HIS:100	VAL:77, PHE:98, ALA:162, ILE:19, LEU:152	-
**5cd**	−7.73	2	VAL:101, ASP:163	ILE:19, LEU:152, VAL:77, PHE:98 ALA:162	Electrostatic interaction (ASP:104), Halogen (fluorine) interaction (ASP:104)
**5ch**	−8.04	2	VAL:101, ASP:163	ILE:19, LEU:152, VAL:77, PHE:98, ALA:162	Electrostatic interaction (ASP:104)
**5ck**	−8.56	-	-	ALA:162, HIS:100, ILE:19, VAL:77, ALA:41, VAL:101, LEU:152	-
**5cs**	−8.48	2	ASP:163, ASP:104	ALA:162, PHE:98, VAL:77, VAL:101, ALA:41, LEU:152, VAL:27, ILE:19	-
doxorubicin	−10.96	6	ASP:163, GLN:149, ASP:104, THR:107, VAL:101, HIS:100	ALA:162, VAL:77, ALA:41, LEU:152, ILE:19	-

^a^ RMSD of the native ligand (palbociclib, LQQ) = 0.83 Å.

**Table 5:** Molecular docking results of compounds **5bh**, **5bk**, **5bl**, and doxorubicin within the active site of epidermal growth factor receptor (EGFR) kinase (PDB: 1M17) ^a^ [48].

Compound	Binding Energy (kcal/mol)	Interaction			
		H-Bond		Hydrophobic	Others
		Number	Residue		
**5bh**	−8.40	1	ASP:831	LYS:721, ALA:719, VAL:702, LEU:694, LEU:820	Miscellaneous interaction (sulfur) (MET:742)
**5bk**	−8.02	3	ASP:831, LYS:721 THR:766	LEU:820, VAL:702, ALA:719, LEU:768, LEU:694, LYS:721	-
**5bl**	−8.61	1	ASP:831	LEU:820, LYS:721, ALA:719, VAL:702, LEU:694	Miscellaneous interaction (sulfur) (MET:742)
doxorubicin	−11.69	3	ASP:831, LYS:721, MET:769	LEU:820, VAL:702, ALA:719, LEU:694, LEU:768, ARG:817	-

^a^ RMSD of the native ligand (erlotinib, AQ4) = 1.64 Å.

**Table 6:** In silico drug-likeness parameters and predicted ADME properties of the synthesized candidates (**5at**, **5bc**, **5bd**, **5bh**, **5bk**, **5bl**, **5ca**, **5cd**, **5ch**, **5ck**, and **5cs**).

Compound	MW (g/mol)	Rotatable Bonds	H-Bond Acceptors	H-Bond Donors	TPSA (Å^2^)	Consensus Log *P*	GI Absorption	BBB Permeant	P-gp Substrate	Lipinski Violations	Bioavailability Score	PAINS Alerts
**5at**	286.33	1	2	0	37.43	3.65	High	Yes	No	0	0.55	0
**5bc**	260.27	0	2	0	28.20	3.74	High	Yes	No	0	0.55	0
**5bd**	260.27	0	2	0	28.20	3.73	High	Yes	No	0	0.55	0
**5bh**	276.72	0	1	0	28.20	3.95	High	Yes	No	0	0.55	0
**5bk**	321.17	0	1	0	28.20	4.04	High	Yes	No	0	0.55	0
**5bl**	321.17	0	1	0	28.20	4.03	High	Yes	No	0	0.55	0
**5ca**	272.30	1	2	0	37.43	3.40	High	Yes	No	0	0.55	0
**5cd**	290.29	1	3	0	37.43	3.64	High	Yes	No	0	0.55	0
**5ch**	306.75	1	2	0	37.43	3.84	High	Yes	No	0	0.55	0
**5ck**	351.20	1	2	0	37.43	3.93	High	Yes	No	0	0.55	0
**5cs**	302.33	2	3	0	46.66	3.31	High	Yes	No	0	0.55	0

## Data Availability

The data supporting this work are in the article and Supplementary Materials.

## Supplementary Materials

The following supporting information can be downloaded at https://www.mdpi.com/article/10.3390/molecules31203731/s1, ^1^H, ^13^C NMR and IR spectra of compounds **7a**–**7c**, **3a**–**3c**, **5ab**−**5au**, **5bb**−**5bu**, and **5cb**−**5cu**; ^19^F NMR spectra of **5ab**, **5ac**, **5ae**, **5bb**–**5be**, and **5cb**–**5ce**; SAR analysis and primary screening data of indolo[1,2-*a*]quinoline derivatives for cancer activity; evaluation of IC50 values; and molecular docking results and 2D/3D interaction poses.
