# Supplementary material for: One-Pot Synthesis and Anticancer Activity Evaluation of Indolo[1,2-a]quinoline-7-carbonitrile Derivatives
Source: Molecules. 2026 Oct 17;31(20):3731. doi: 10.3390/molecules31203731 (PMC13707563; doi:10.3390/molecules31203731)
Supplement: Supplementary file 1 [file molecules-31-03731-s001.zip › molecules-4598344-supplementary.pdf]

Supplementary Materials

One-pot synthesis and anticancer activity evaluation of indolo[1,2-*a*]quinoline-7-carbonitrile derivatives

Kredmanee Yimnoi [a], Norasian Boosaman [a], Nutthawat Chuanopparat [a], Kedchin Jearawuttanakul [b], Sawinee Seemakhan [b], Tanawadee Khumpanied [b], Warawuth Wannalo [b], Suparerk Borwornpinyo [b, c,] * and Paiboon Ngernmeesri [a,] *

[a] Department of Chemistry and Center of Excellence for Innovation in Chemistry, Faculty of Science, Kasetsart University, Bangkok 10900, Thailand. E-mail: suparerk.bor@mahidol.ac.th; fscipbn@ku.ac.th

[b] Excellent Center for Drug Discovery, Faculty of Science, Mahidol University, Bangkok, 10400, Thailand

[c] Department of Biotechnology, Faculty of Science, Mahidol University, Bangkok, 10400, Thailand

Table of Contents

1. ^{1}H and ^{13}C{1H} NMR spectra of compound **7a** S1
2. IR spectrum of **7a** S2
3. ^{1}H and ^{13}C{1H} NMR spectra of compound **7b** S3
4. IR spectrum of **7b** S4
5. ^{1}H and ^{13}C{1H} NMR spectra of compound **7c** S5
6. IR spectrum of **7c** S6
7. ^{1}H and ^{13}C{1H} NMR spectra of compound **3a** S7
8. IR spectrum of **3a** S8
9. ^{1}H and ^{13}C{1H} NMR spectra of compound **3b** S9
10. IR spectrum of **3b** S10
11. ^{1}H and ^{13}C{1H} NMR spectra of compound **3c** S11
12. IR spectrum of **3c** S12
13. ^{1}H, ^{13}C{^{1}H} and ^{19}F NMR spectra of compound **5ab** S13
14. IR spectrum of **5ab** S16
15. ^{1}H, ^{13}C{^{1}H} and ^{19}F NMR spectra of compound **5ac** S17
16. IR spectrum of **5ac** S19
17. ^{1}H, ^{13}C{^{1}H} and ^{19}F NMR spectra of compound **5ae** S20
18. IR spectrum of **5ae** S22
19. ^{1}H and ^{13}C{^{1}H} NMR spectra of compound **5af** S23
20. IR spectrum of **5af** S24
21. ^{1}H and ^{13}C{^{1}H} NMR spectra of compound **5ag** S25
22. IR spectrum of **5ag** S26
23. ^{1}H and ^{13}C{^{1}H} NMR spectra of compound **5ah** S27
24. IR spectrum of **5ah** S28
25. ^{1}H and ^{13}C{^{1}H} NMR spectra of compound **5ai** S29
26. IR spectrum of **5ai** S30

Table of Contents

27. ^{1}H and $^{13}C\{^{1}H\}$ NMR spectra of compound **5aj** S31

28. IR spectrum of **5aj** S32

29. ^{1}H and $^{13}C\{^{1}H\}$ NMR spectra of compound **5ak** S33

30. IR spectrum of **5ak** S34

31. ^{1}H and $^{13}C\{^{1}H\}$ NMR spectra of compound **5al** S35

32. IR spectrum of **5al** S36

33. ^{1}H and $^{13}C\{^{1}H\}$ NMR spectra of compound **5am** S37

34. IR spectrum of **5am** S38

35. ^{1}H and $^{13}C\{^{1}H\}$ NMR spectra of compound **5an** S39

36. IR spectrum of **5an** S40

37. ^{1}H and $^{13}C\{^{1}H\}$ NMR spectra of compound **5ao** S41

38. IR spectrum of **5ao** S42

39. ^{1}H and $^{13}C\{^{1}H\}$ NMR spectra of compound **5ap** S43

40. IR spectrum of **5ap** S44

41. ^{1}H and $^{13}C\{^{1}H\}$ NMR spectra of compound **5aq** S45

42. IR spectrum of **5aq** S46

43. ^{1}H and $^{13}C\{^{1}H\}$ NMR spectra of compound **5ar** S47

44. IR spectrum of **5ar** S48

45. ^{1}H and $^{13}C\{^{1}H\}$ NMR spectra of compound **5as** S49

46. IR spectrum of **5as** S50

47. ^{1}H and $^{13}C\{^{1}H\}$ NMR spectra of compound **5at** S51

48. IR spectrum of **5at** S52

49. ^{1}H and $^{13}C\{^{1}H\}$ NMR spectra of compound **5au** S53

50. IR spectrum of **5au** S54

51. ^{1}H, $^{13}C\{^{1}H\}$ and ^{19}F NMR spectra of compound **5bb** S55

52. IR spectrum of **5bb** S57

Table of Contents

53. ^{1}H, $^{13}C\{^{1}H\}$ and ^{19}F NMR spectra of compound **5bc** S58

54. IR spectrum of **5bc** S60

55. ^{1}H, $^{13}C\{^{1}H\}$ and ^{19}F NMR spectra of compound **5bd** S61

56. IR spectrum of **5bd** S63

57. ^{1}H, $^{13}C\{^{1}H\}$ and ^{19}F NMR spectra of compound **5be** S64

58. IR spectrum of **5be** S66

59. ^{1}H and $^{13}C\{^{1}H\}$ NMR spectra of compound **5bf** S67

60. IR spectrum of **5bf** S68

61. ^{1}H and $^{13}C\{^{1}H\}$ NMR spectra of compound **5bg** S69

62. IR spectrum of **5bg** S70

63. ^{1}H and $^{13}C\{^{1}H\}$ NMR spectra of compound **5bh** S71

64. IR spectrum of **5bh** S72

65. ^{1}H and $^{13}C\{^{1}H\}$ NMR spectra of compound **5bi** S73

66. IR spectrum of **5bi** S74

67. ^{1}H and $^{13}C\{^{1}H\}$ NMR spectra of compound **5bk** S75

68. IR spectrum of **5bk** S76

69. ^{1}H and $^{13}C\{^{1}H\}$ NMR spectra of compound **5bl** S77

70. IR spectrum of **5bl** S78

71. ^{1}H and $^{13}C\{^{1}H\}$ NMR spectra of compound **5bm** S79

72. IR spectrum of **5bm** S80

73. ^{1}H and $^{13}C\{^{1}H\}$ NMR spectra of compound **5bn** S81

74. IR spectrum of **5bn** S82

75. ^{1}H and $^{13}C\{^{1}H\}$ NMR spectra of compound **5bo** S83

76. IR spectrum of **5bo** S84

77. ^{1}H and $^{13}C\{^{1}H\}$ NMR spectra of compound **5bp** S85

78. IR spectrum of **5bp** S86

Table of Contents

79. ^{1}H and $^{13}C\{^{1}H\}$ NMR spectra of compound **5bq** S87

80. IR spectrum of **5bq** S88

81. ^{1}H and $^{13}C\{^{1}H\}$ NMR spectra of compound **5br** S89

82. IR spectrum of **5br** S90

83. ^{1}H and $^{13}C\{^{1}H\}$ NMR spectra of compound **5bs** S91

84. IR spectrum of **5bs** S92

85. ^{1}H and $^{13}C\{^{1}H\}$ NMR spectra of compound **5bt** S93

86. IR spectrum of **5bt** S94

87. ^{1}H and $^{13}C\{^{1}H\}$ NMR spectra of compound **5bu** S95

88. IR spectrum of **5bu** S96

89. ^{1}H, $^{13}C\{^{1}H\}$ and ^{19}F NMR spectra of compound **5cb** S97

90. IR spectrum of **5cb** S99

91. ^{1}H, $^{13}C\{^{1}H\}$ and ^{19}F NMR spectra of compound **5cc** S100

92. IR spectrum of **5cc** S102

93. ^{1}H, $^{13}C\{^{1}H\}$ and ^{19}F NMR spectra of compound **5cd** S103

94. IR spectrum of **5cd** S105

95. ^{1}H, $^{13}C\{^{1}H\}$ and ^{19}F NMR spectra of compound **5ce** S106

96. IR spectrum of **5ce** S108

97. ^{1}H and $^{13}C\{^{1}H\}$ NMR spectra of compound **5cf** S109

98. IR spectrum of **5cf** S110

99. ^{1}H and $^{13}C\{^{1}H\}$ NMR spectra of compound **5cg** S111

100. IR spectrum of **5cg** S112

101. ^{1}H and $^{13}C\{^{1}H\}$ NMR spectra of compound **5ch** S113

102. IR spectrum of **5ch** S114

103. ^{1}H and $^{13}C\{^{1}H\}$ NMR spectra of compound **5ci** S115

104. IR spectrum of **5ci** S116

Table of Contents

105. ^{1}H and $^{13}C\{^{1}H\}$ NMR spectra of compound **5ck** S117

106. IR spectrum of **5ck** S118

107. ^{1}H and $^{13}C\{^{1}H\}$ NMR spectra of compound **5cm** S119

108. IR spectrum of **5cm** S120

109. ^{1}H and $^{13}C\{^{1}H\}$ NMR spectra of compound **5co** S121

110. IR spectrum of **5co** S122

111. ^{1}H and $^{13}C\{^{1}H\}$ NMR spectra of compound **5cq** S123

112. IR spectrum of **5cq** S124

113. ^{1}H and $^{13}C\{^{1}H\}$ NMR spectra of compound **5cr** S125

114. IR spectrum of **5cr** S126

115. ^{1}H and $^{13}C\{^{1}H\}$ NMR spectra of compound **5cs** S127

116. IR spectrum of **5cs** S128

117. ^{1}H and $^{13}C\{^{1}H\}$ NMR spectra of compound **5ct** S129

118. IR spectrum of **5ct** S130

119. ^{1}H and $^{13}C\{^{1}H\}$ NMR spectra of compound **5cu** S131

120. IR spectrum of **5cu** S132

121. SAR analysis and primary screening data of indolo[1,2-*a*]quinoline derivatives for cancer activity S133t

122. Evaluation of IC_{50} values S137

123. Molecular docking results and 2D/3D interaction poses S144

^{1}H NMR spectrum (400 MHz, DMSO-d_6) of compound **7a**

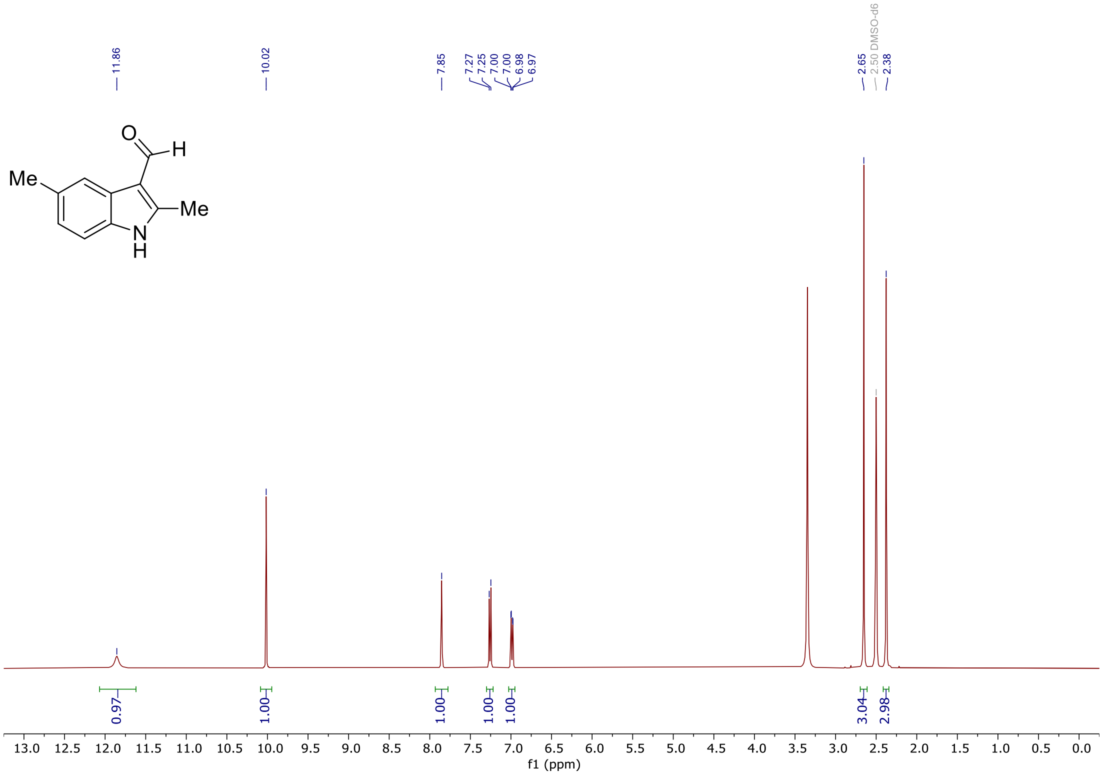

^{13}C{^{1}H} NMR spectrum (100 MHz, DMSO-d_6) of compound **7a**

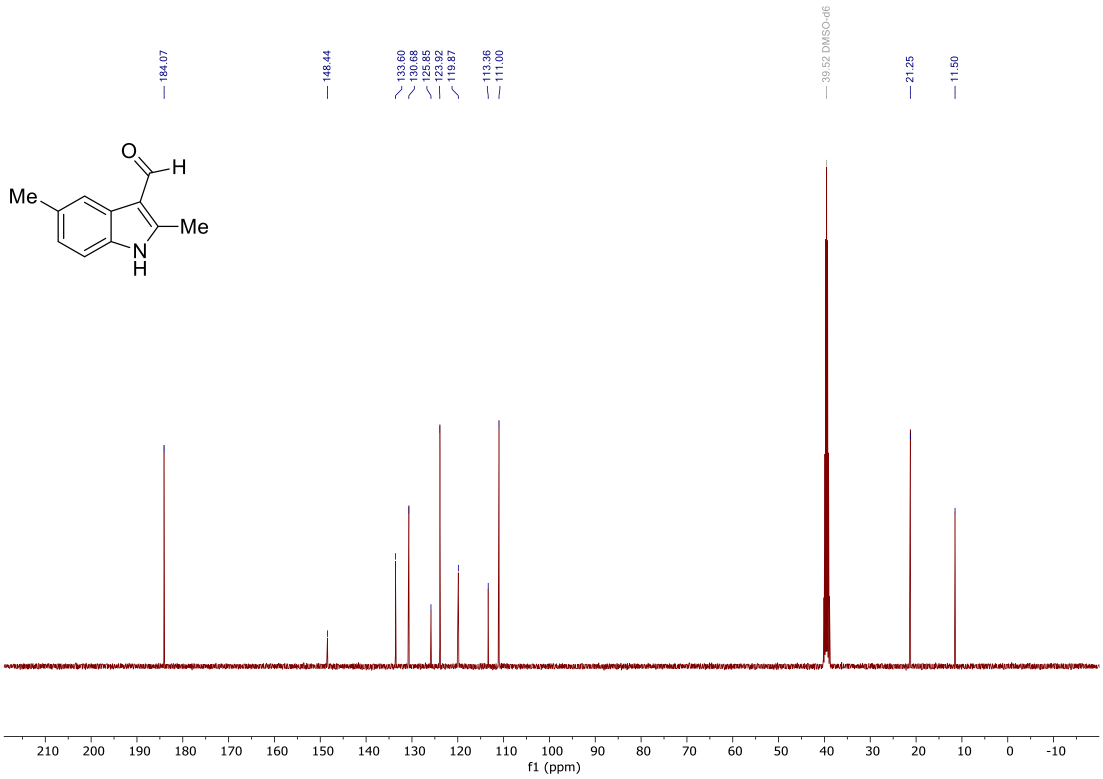

IR spectrum (neat) of compound **7a**

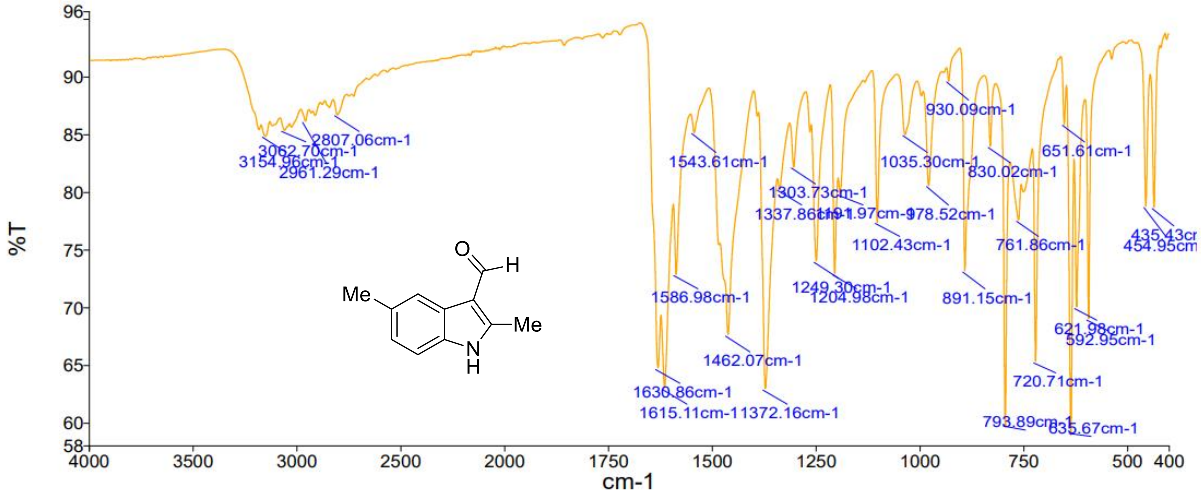

^{1}H NMR spectrum (400 MHz, DMSO-d_6) of compound **7b**

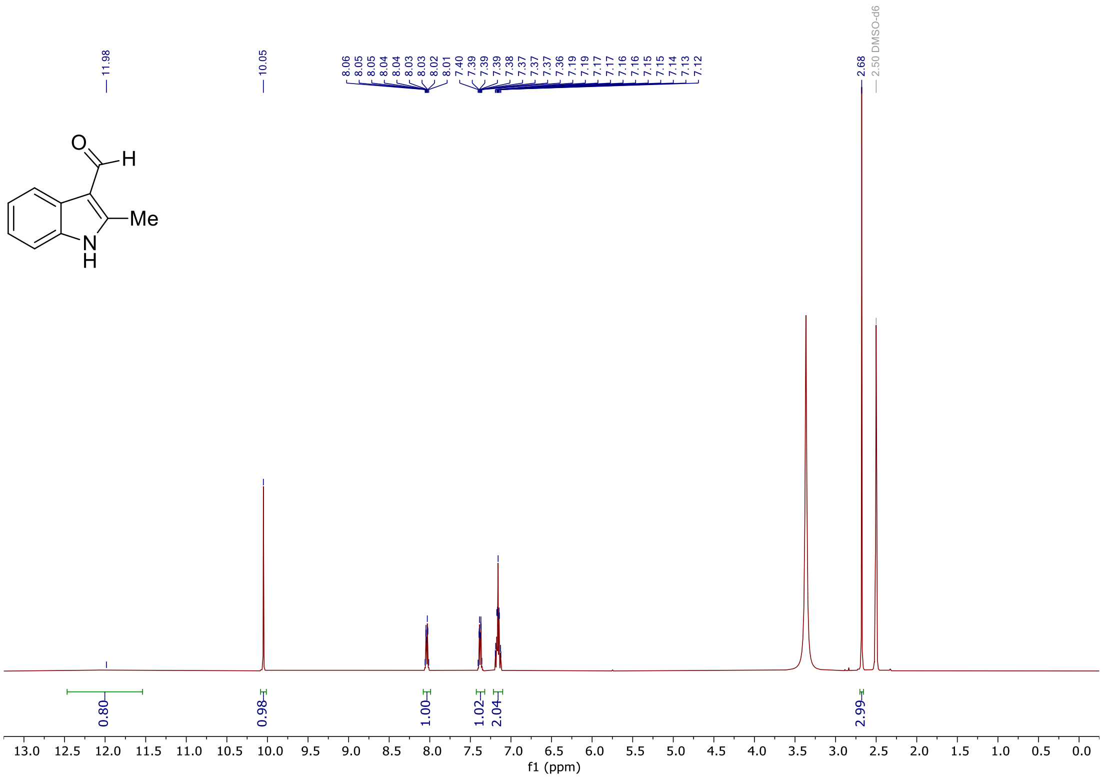

^{13}C{^{1}H} NMR spectrum (100 MHz, DMSO-d_6) of compound **7b**

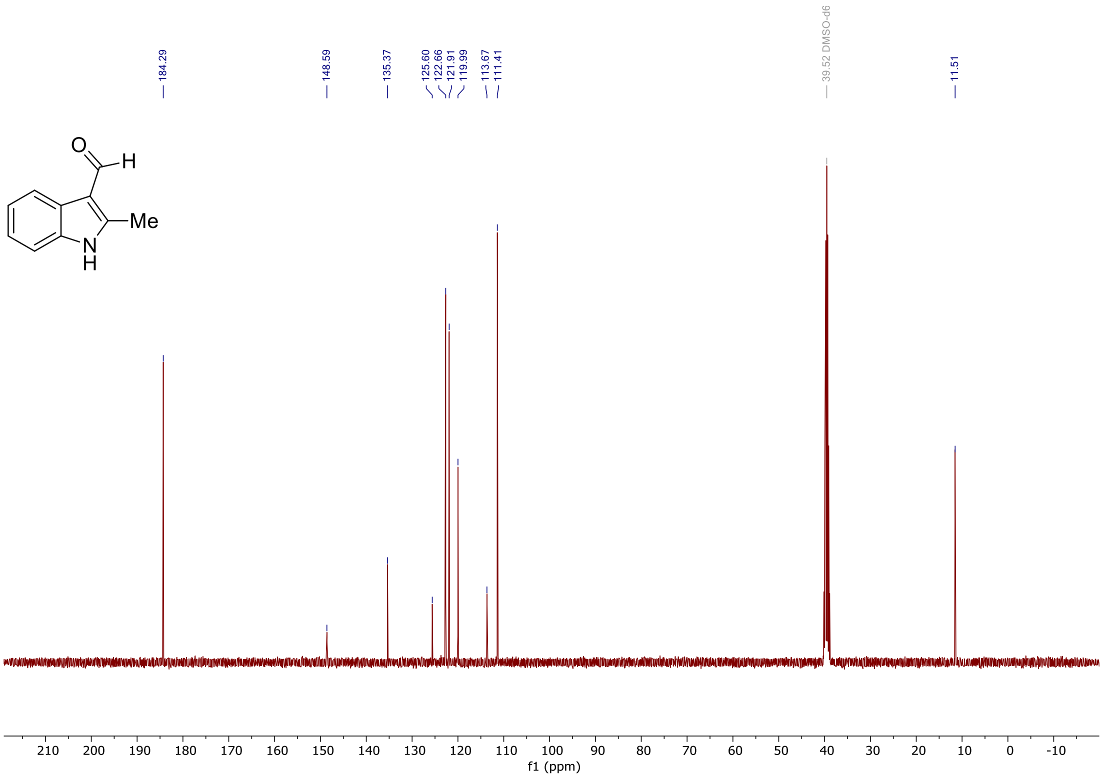

IR spectrum (neat) of compound **7b**

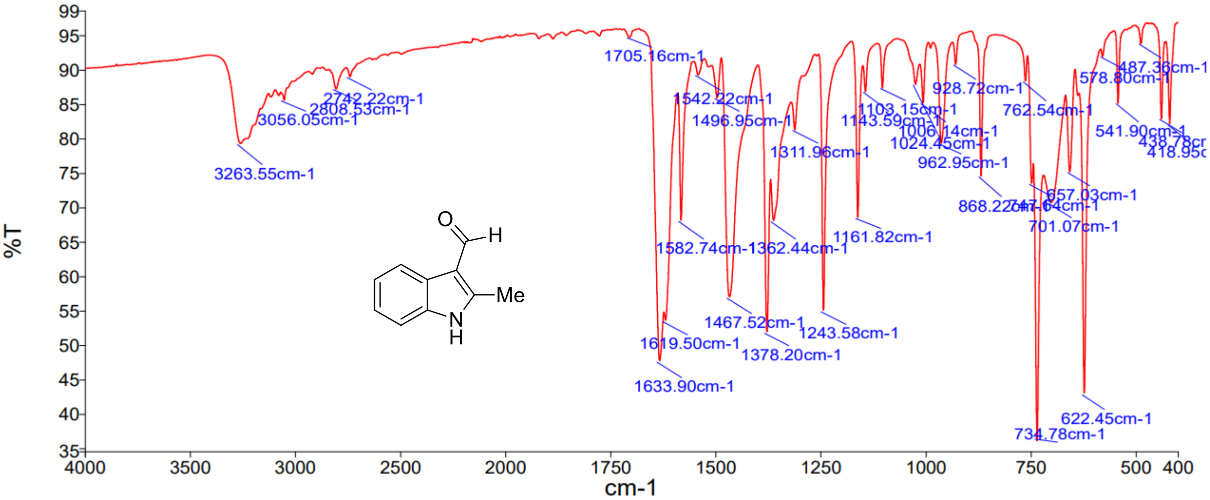

^{1}H NMR spectrum (400 MHz, DMSO-d_6) of compound **7c**

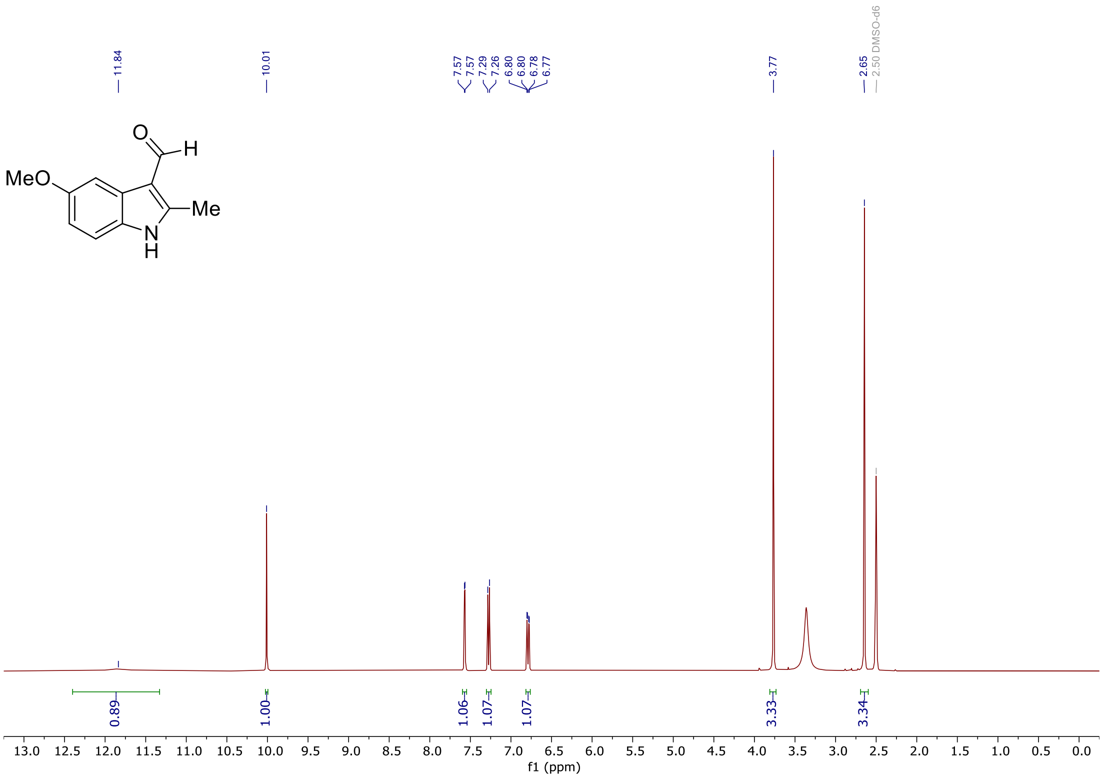

^{13}C{^{1}H} NMR spectrum (100 MHz, DMSO-d_6) of compound **7c**

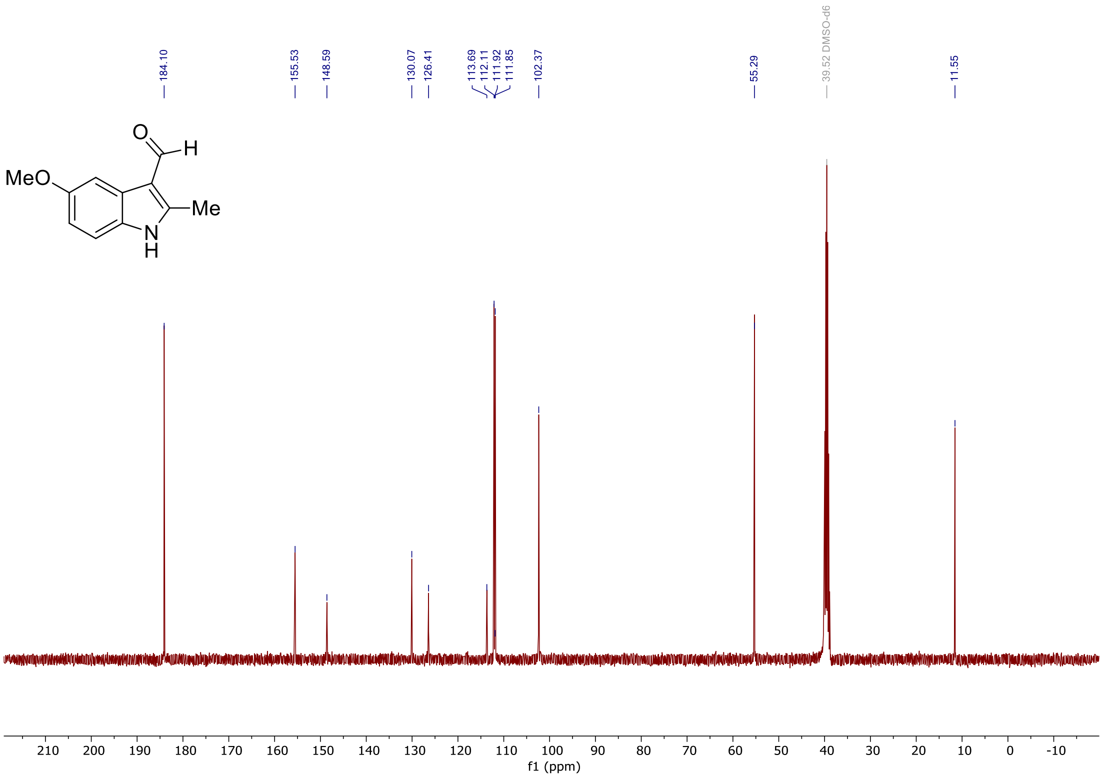

IR spectrum (neat) of compound **7c**

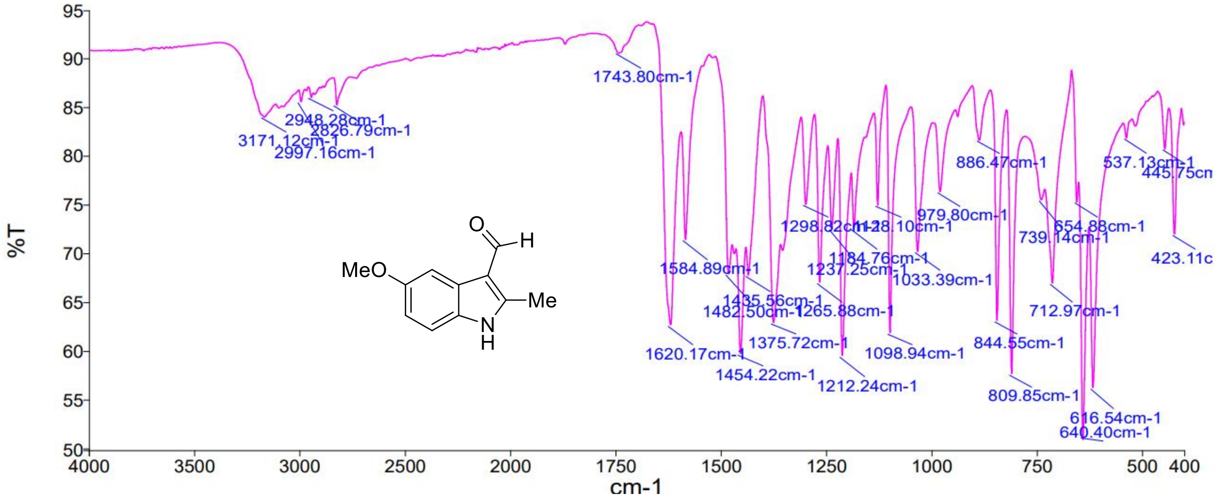

^{1}H NMR spectrum (400 MHz, DMSO-d_6) of compound **3a**

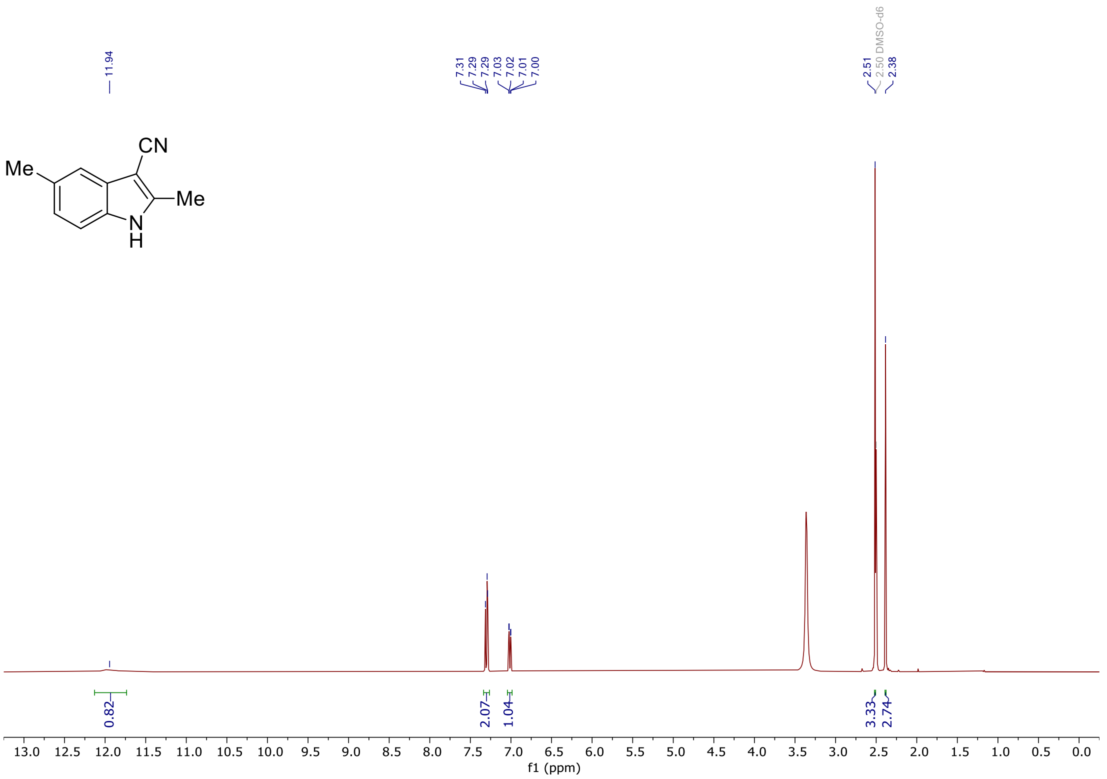

$^{13}C\{^{1}H\}$ NMR spectrum (100 MHz, DMSO-d_6) of compound **3a**

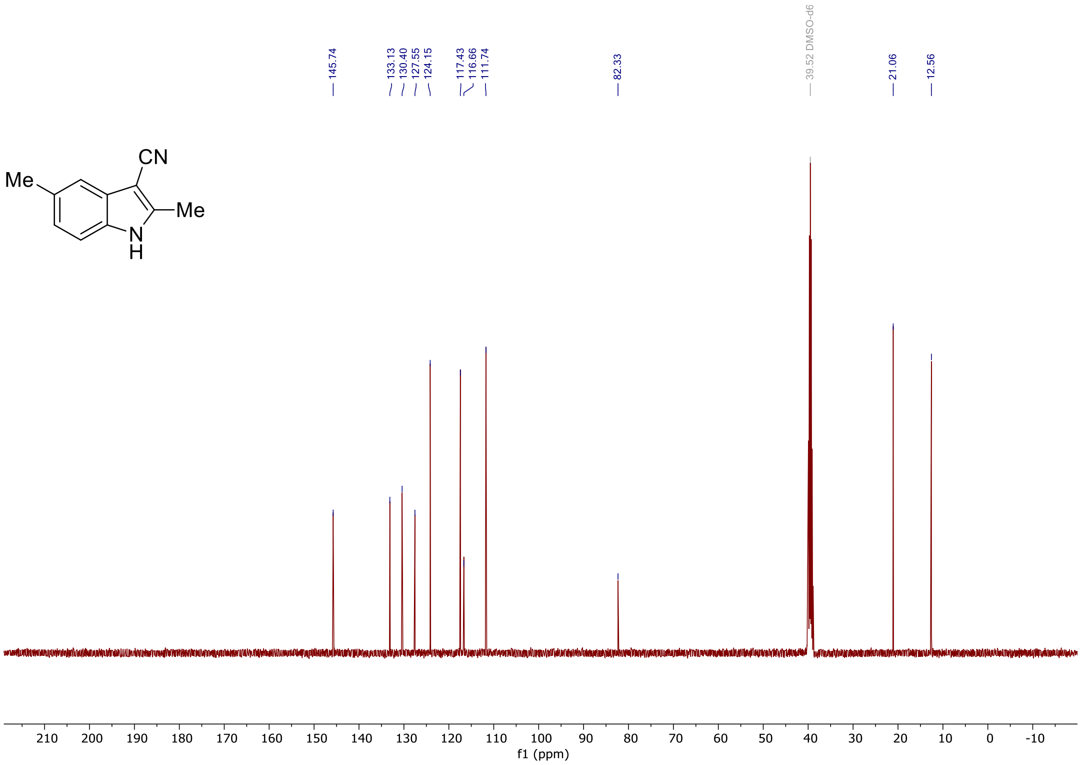

IR spectrum (neat) of compound **3a**

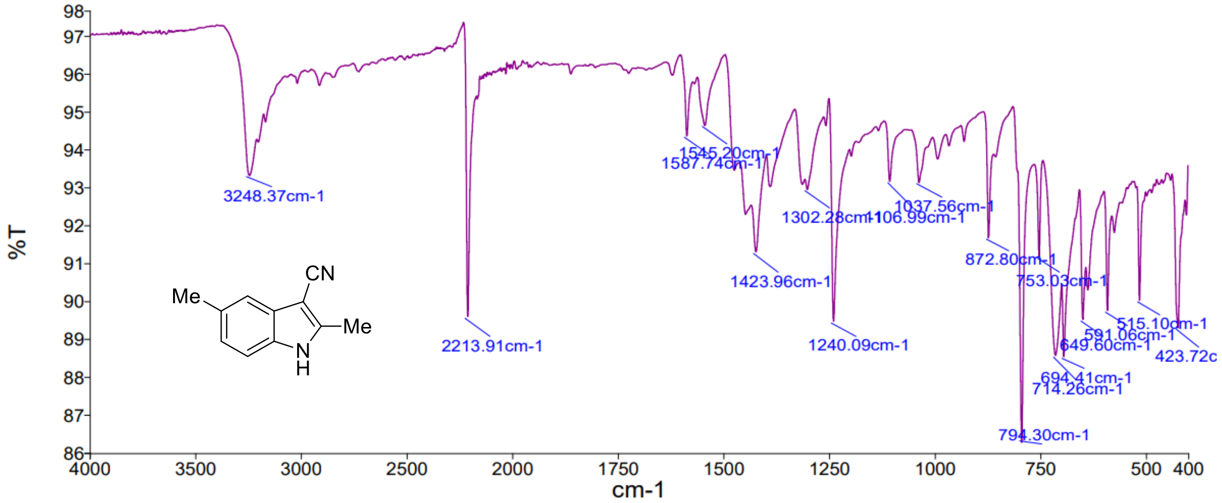

^{1}H NMR spectrum (400 MHz, DMSO-d_6) of compound **3b**

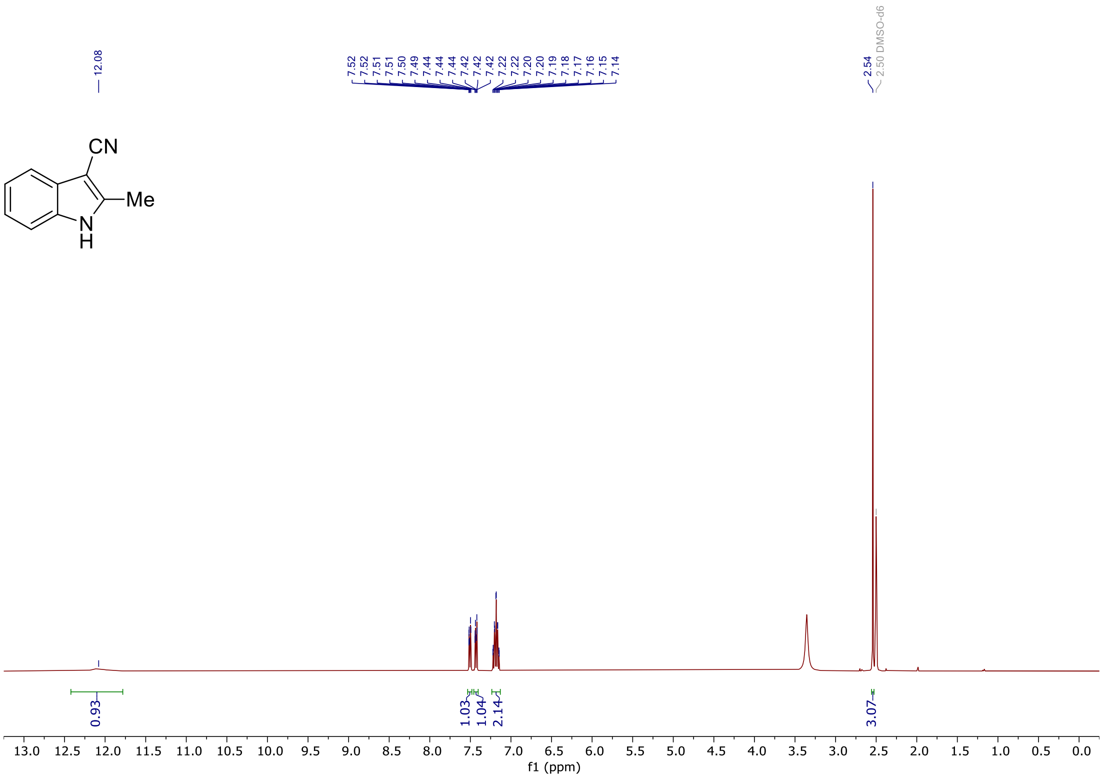

$^{13}C\{^{1}H\}$ NMR spectrum (100 MHz, DMSO-d_6) of compound **3b**

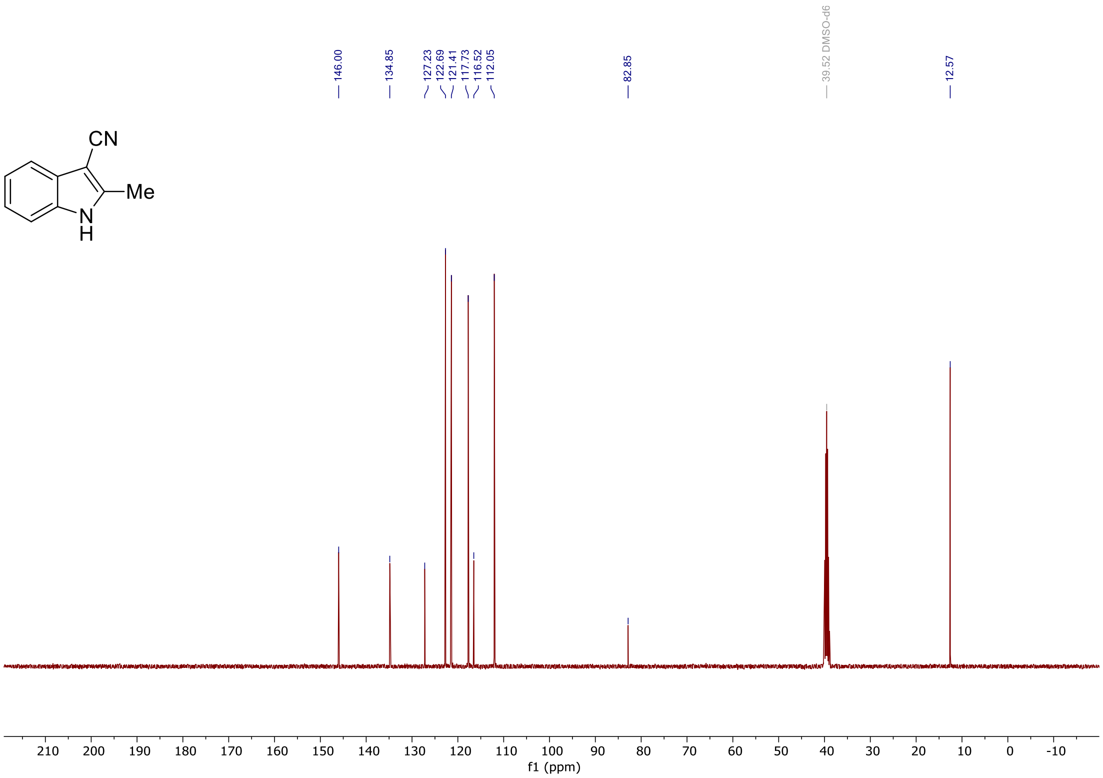

IR spectrum (neat) of compound **3b**

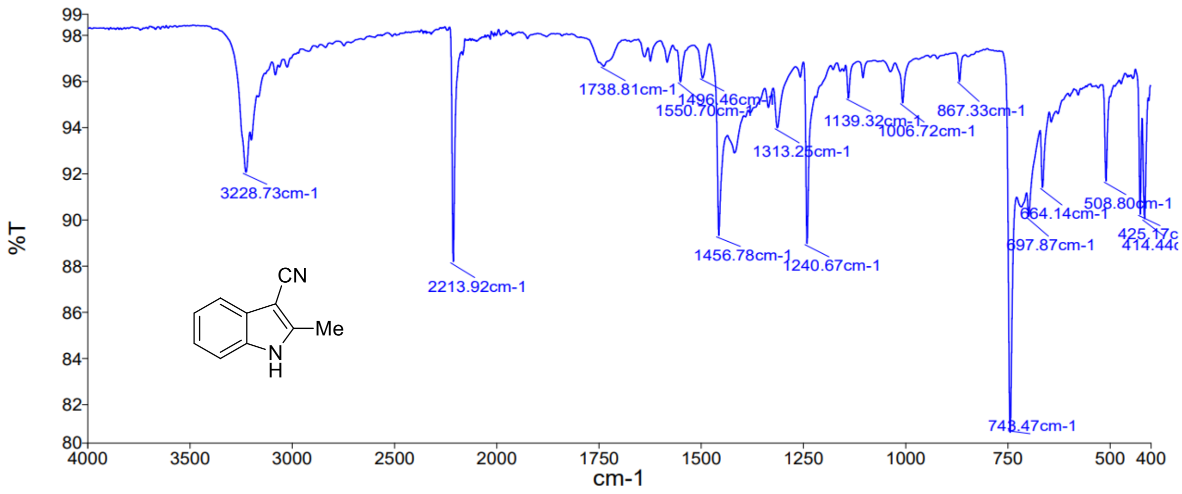

^{1}H NMR spectrum (400 MHz, DMSO-d_6) of compound **3c**

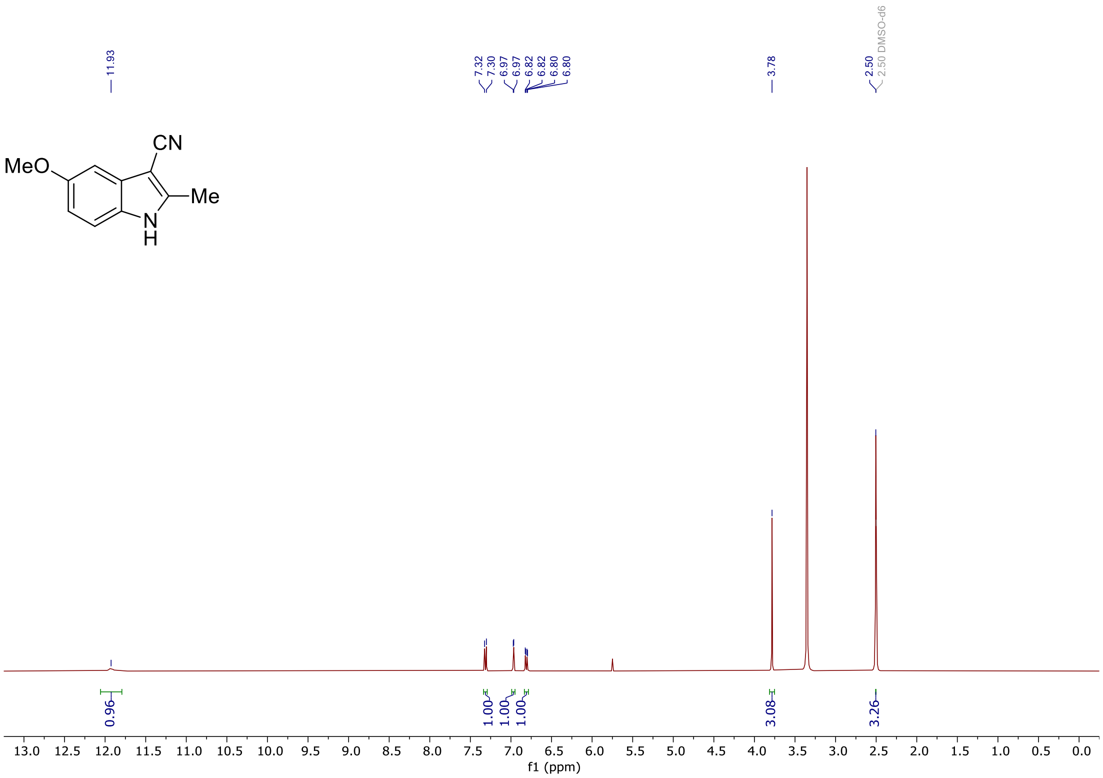

^{13}C{^{1}H} NMR spectrum (100 MHz, DMSO-d_6) of compound **3c**

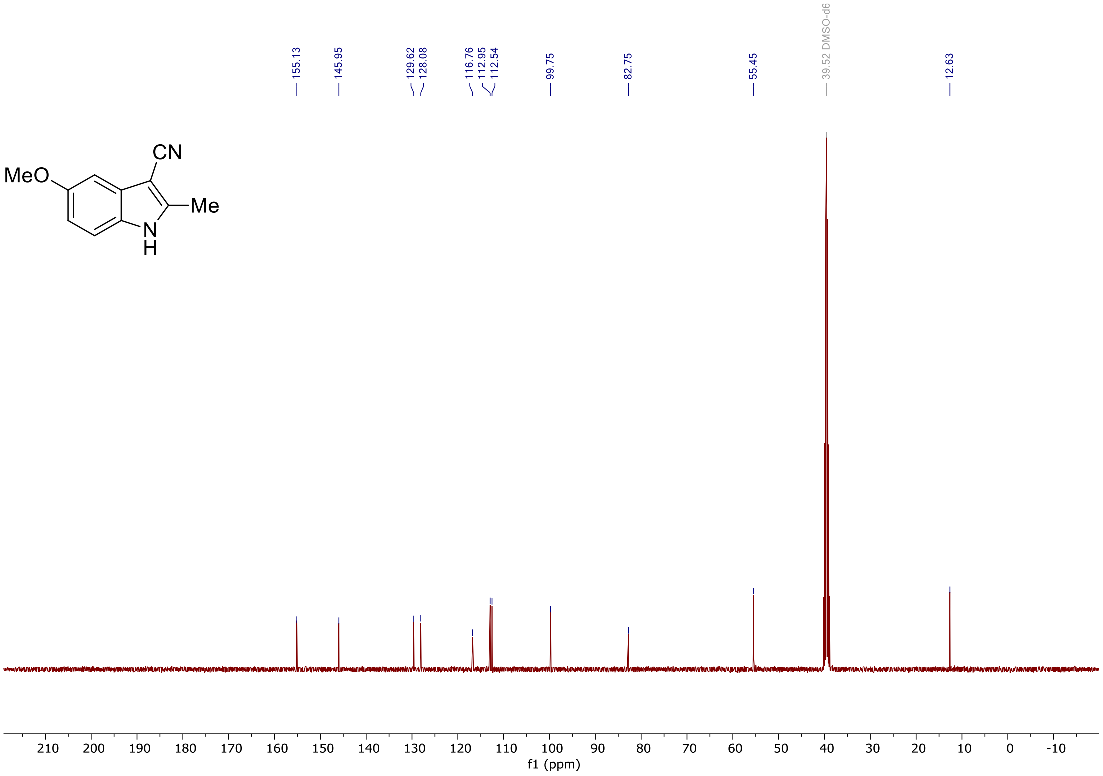

IR spectrum (neat) of compound **3c**

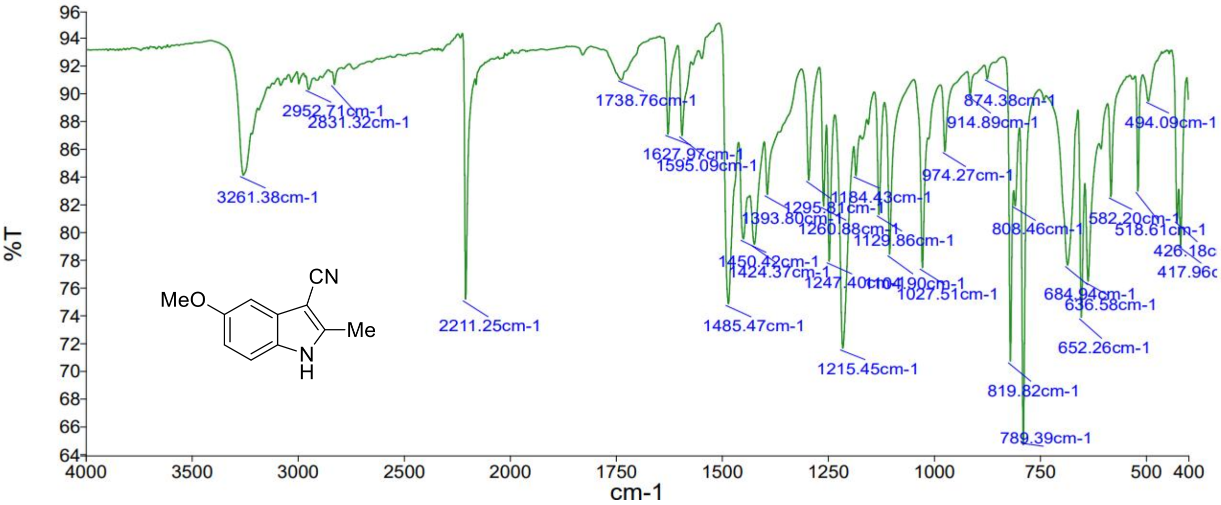

^{1}H NMR spectrum (400 MHz, DMSO-d_6) of compound **5ab**

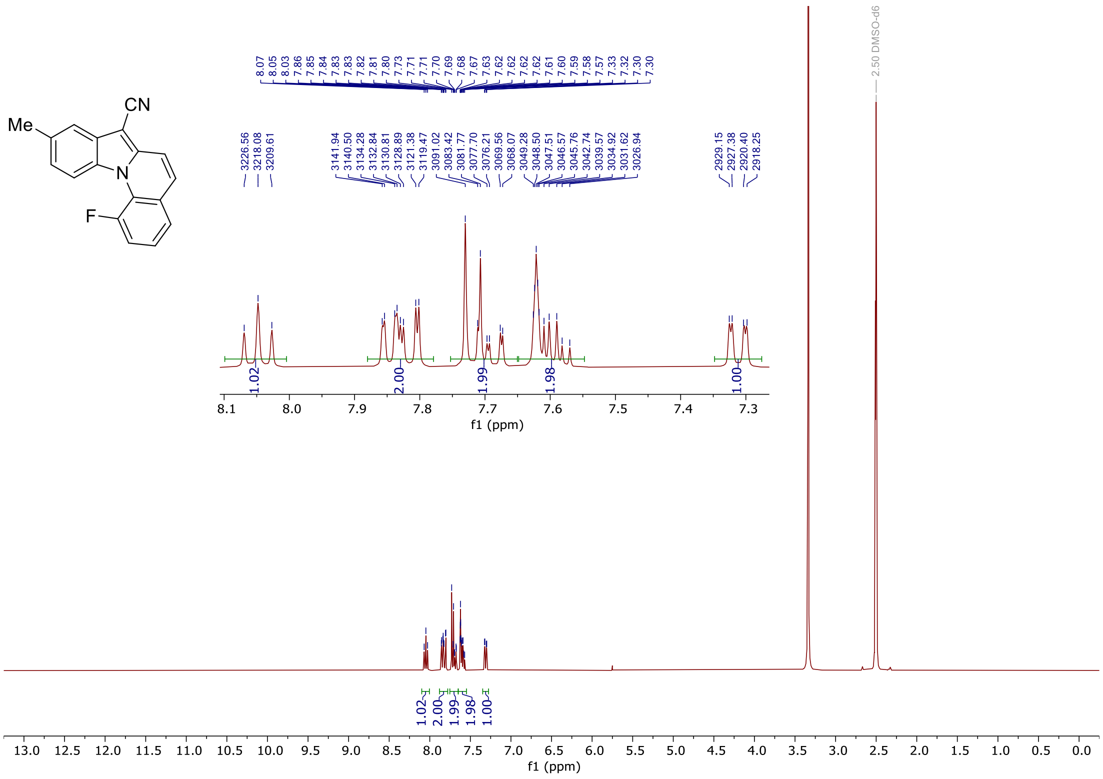

^{1}H NMR spectrum (400 MHz, $CDCl_3$) of compound of compound **5ab**

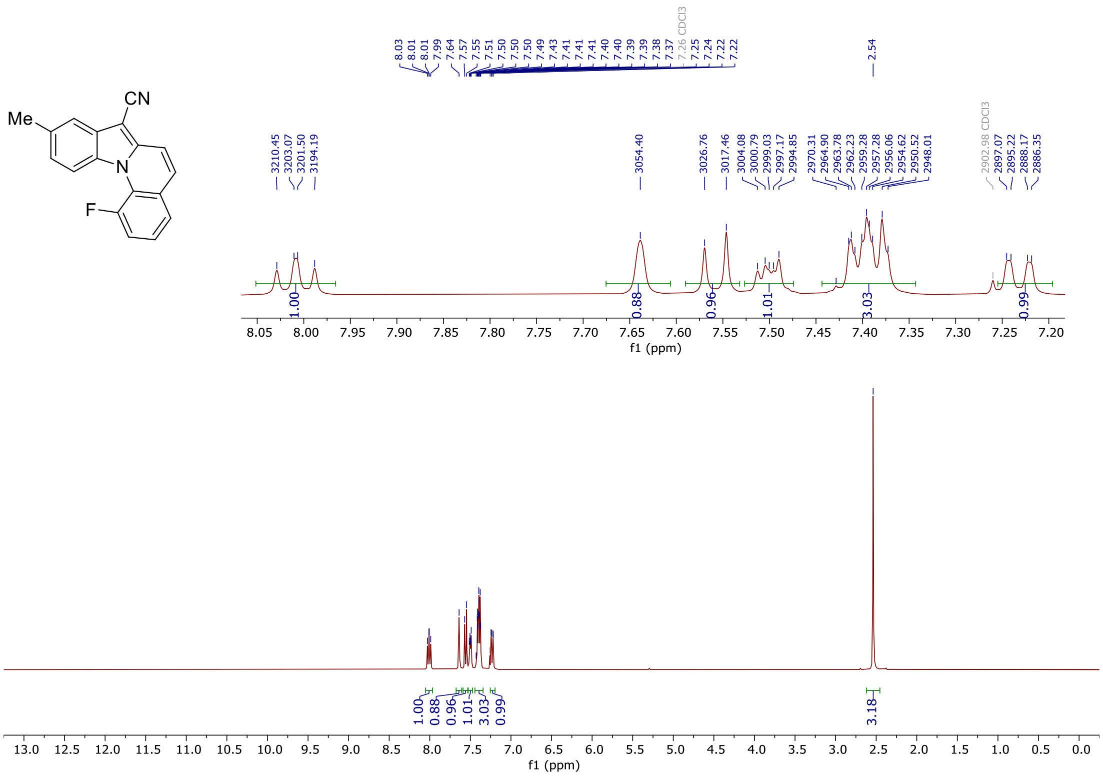

$^{13}C\{^{1}H\}$ NMR spectrum (100 MHz, DMSO-d_6) of compound **5ab**

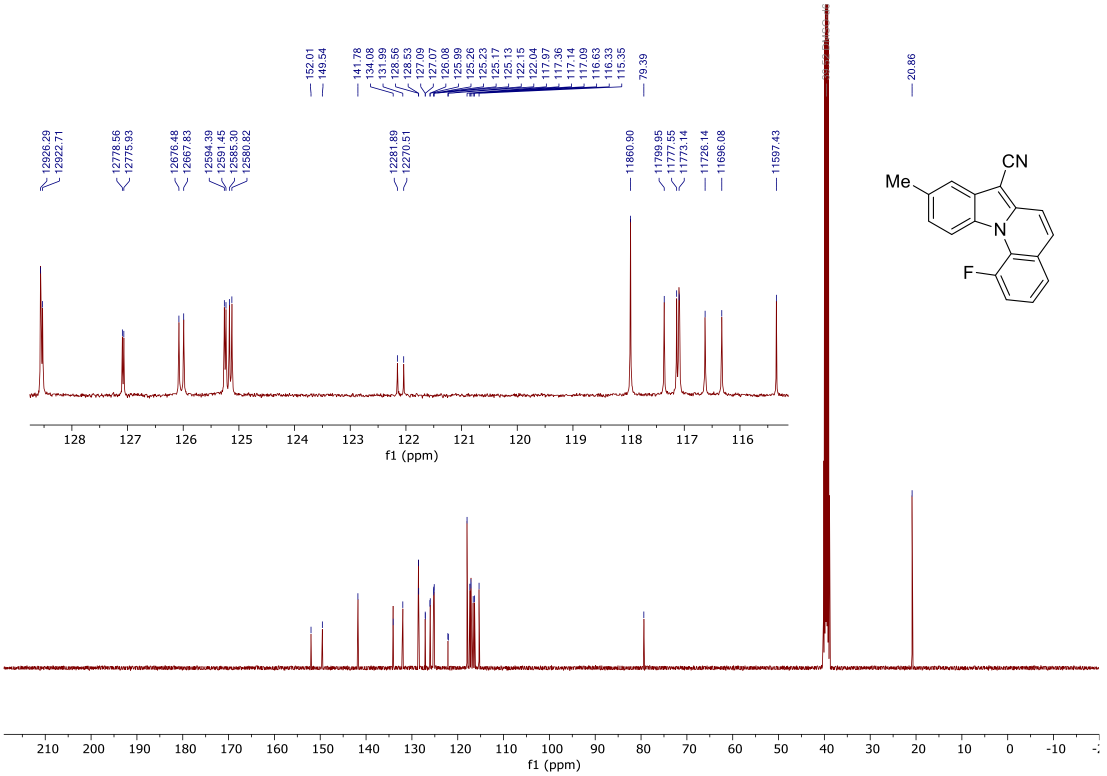

^{19}F NMR spectrum (470 MHz, $CDCl_3$) of compound **5ab**

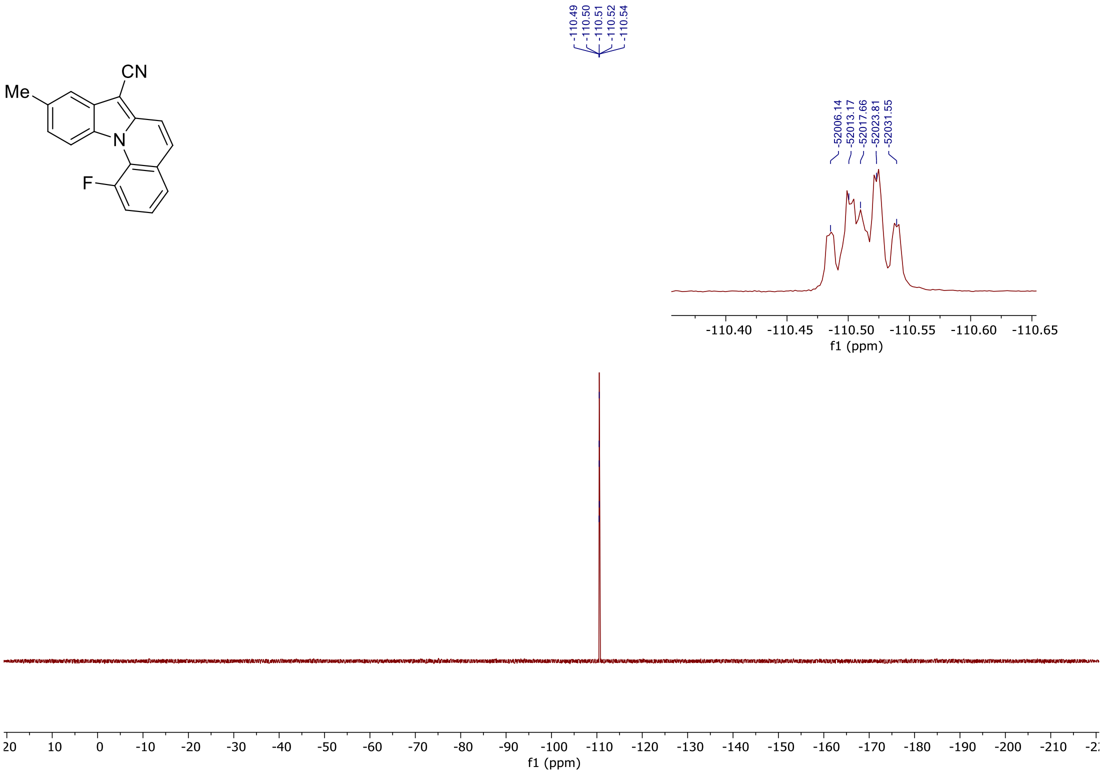

^{19}F NMR spectrum (470 MHz, $CDCl_3$) of compound **5ab** with an internal standard (fluorobenzene)

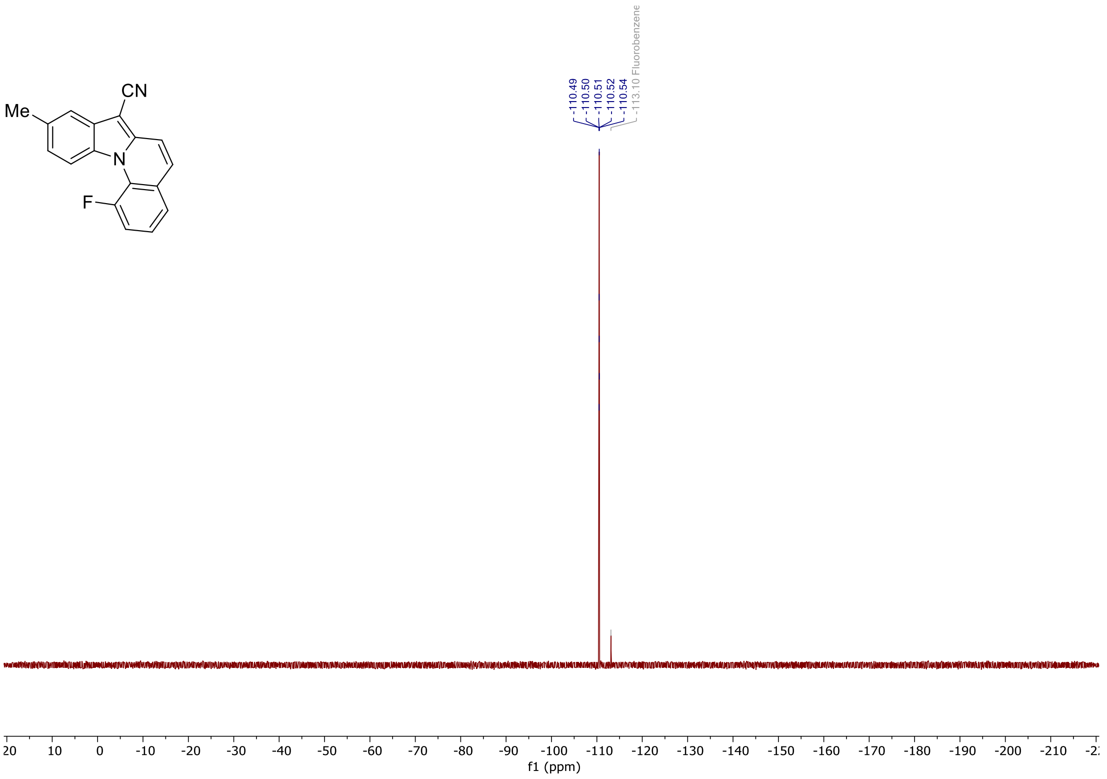

IR spectrum (neat) of compound **5ab**

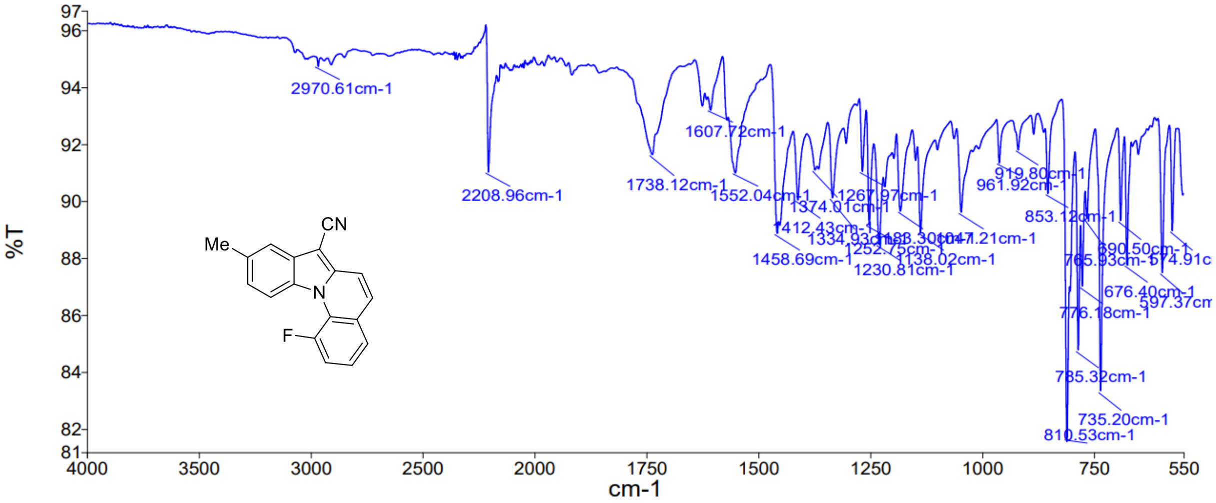

^{1}H NMR spectrum (400 MHz, DMSO-d_6) of compound **5ac**

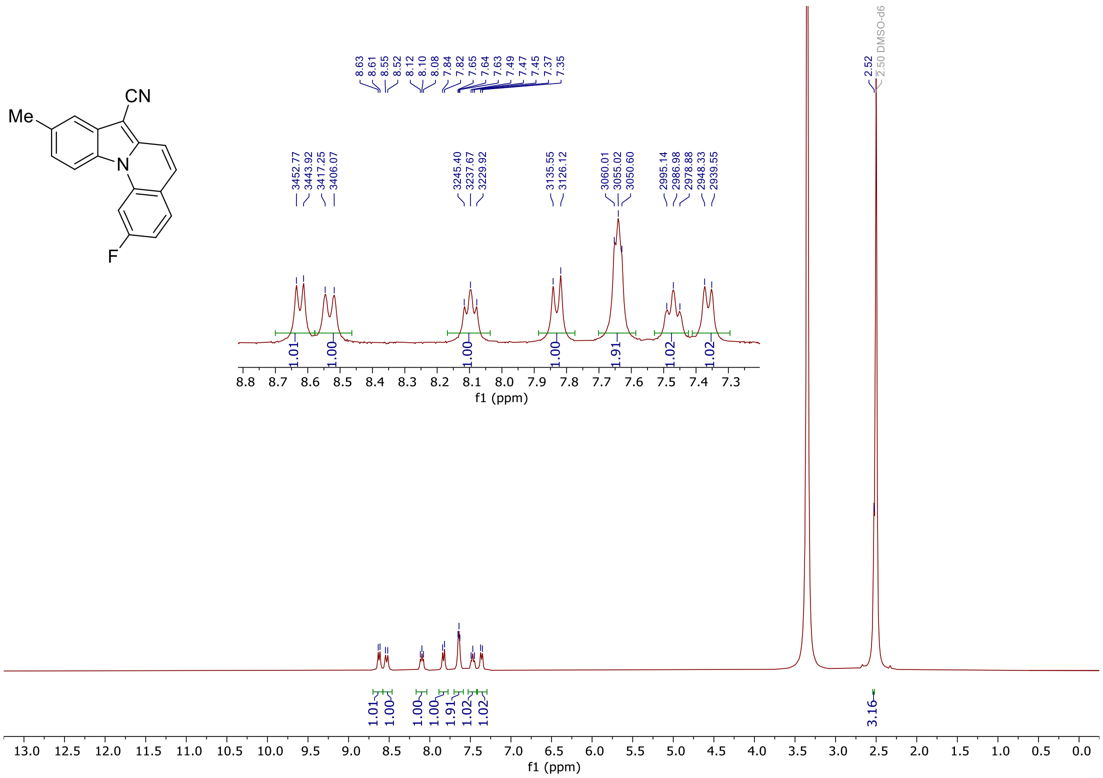

^{13}C{^{1}H} NMR spectrum (100 MHz, DMSO-d_6) of compound **5ac**

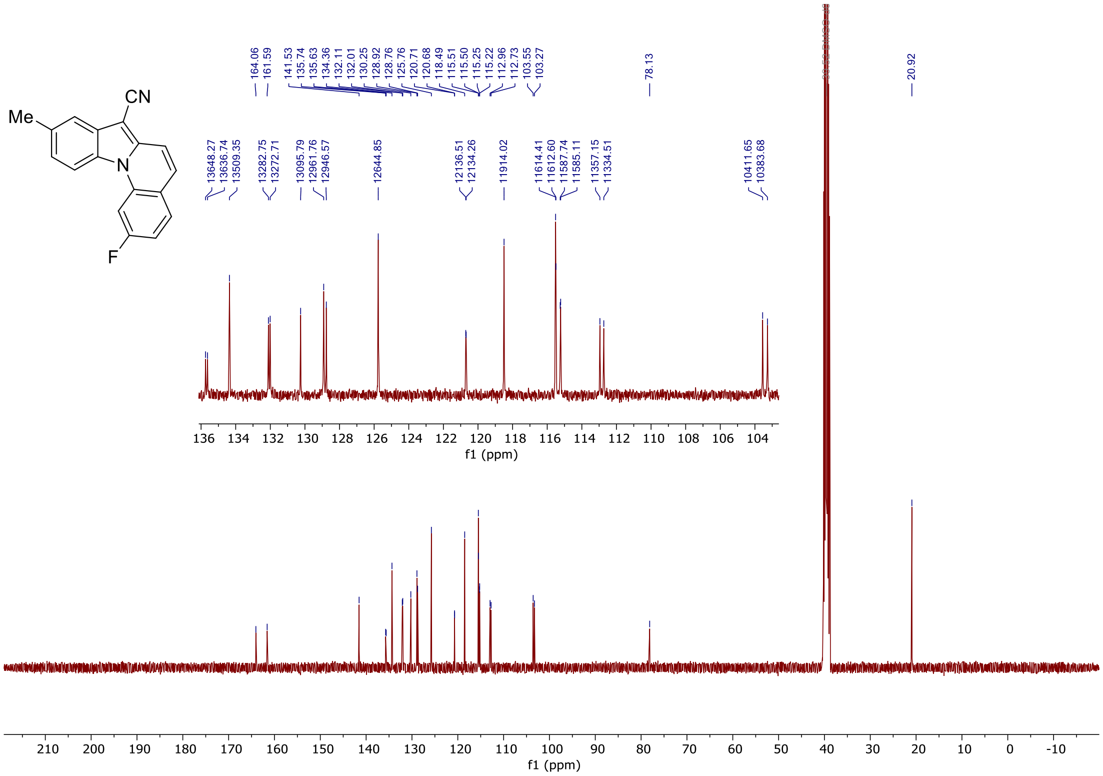

^{19}F NMR spectrum (470 MHz, $CDCl_3$) of compound **5ac**

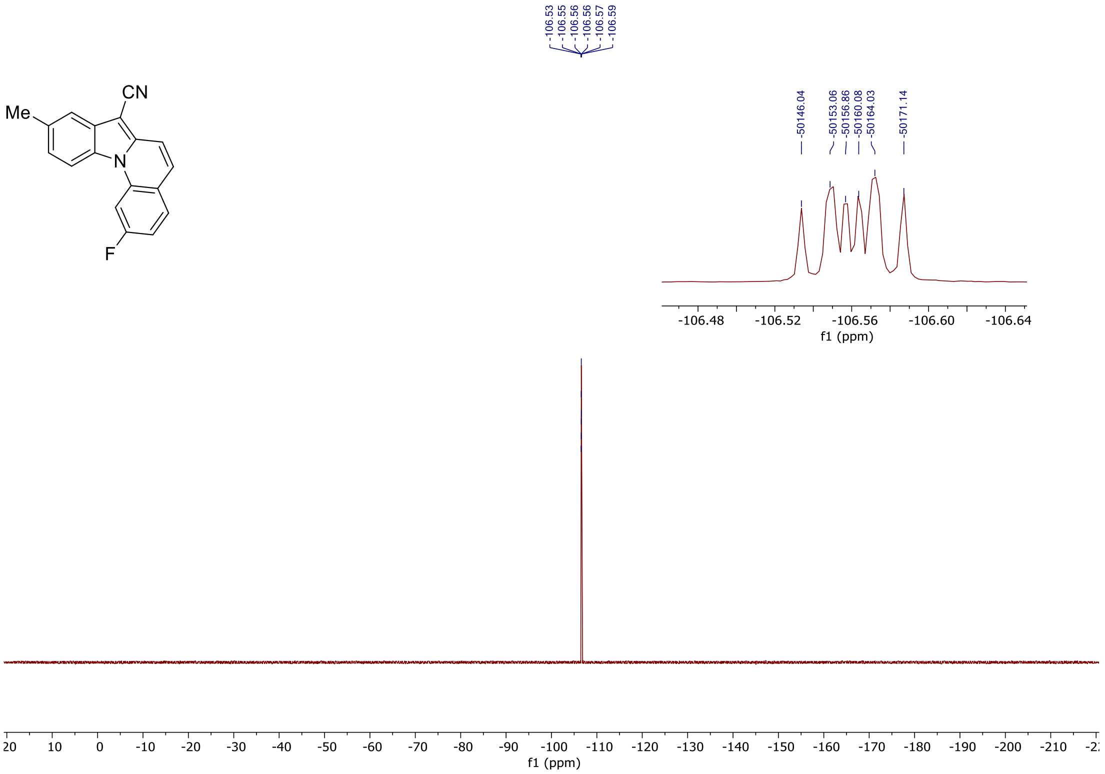

^{19}F NMR spectrum (470 MHz, $CDCl_3$) of compound **5ac** with an internal standard (fluorobenzene)

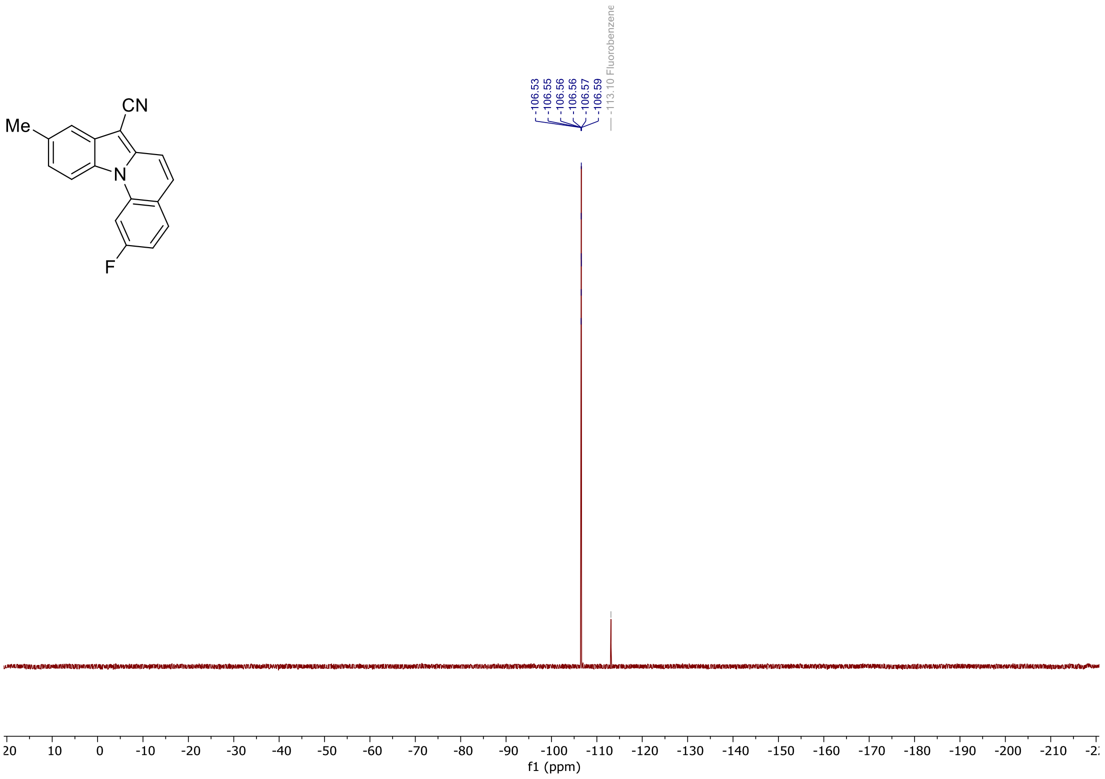

IR spectrum (neat) of compound **5ac**

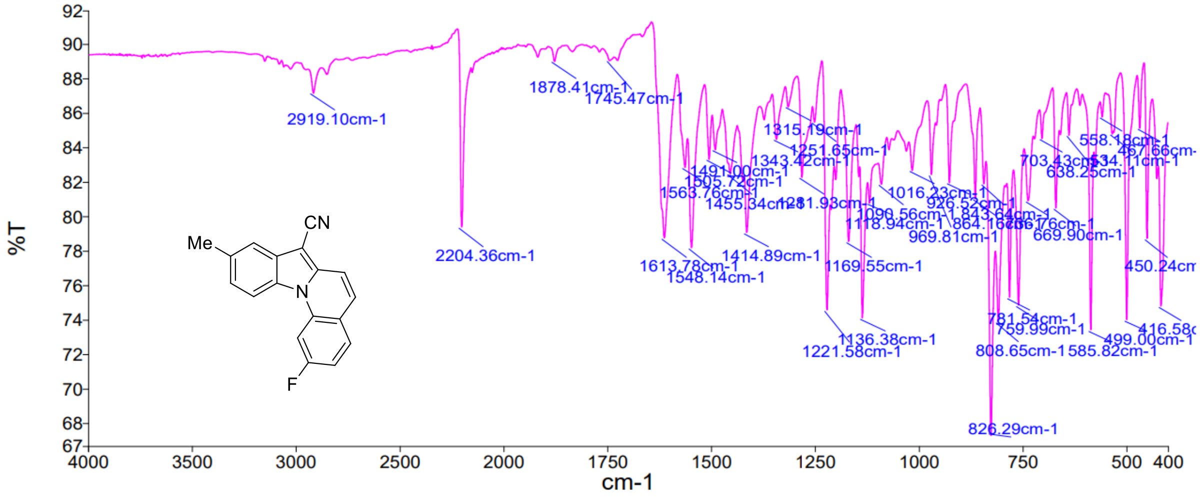

^{1}H NMR spectrum (400 MHz, $CDCl_3$) of compound **5ae**

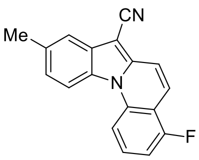

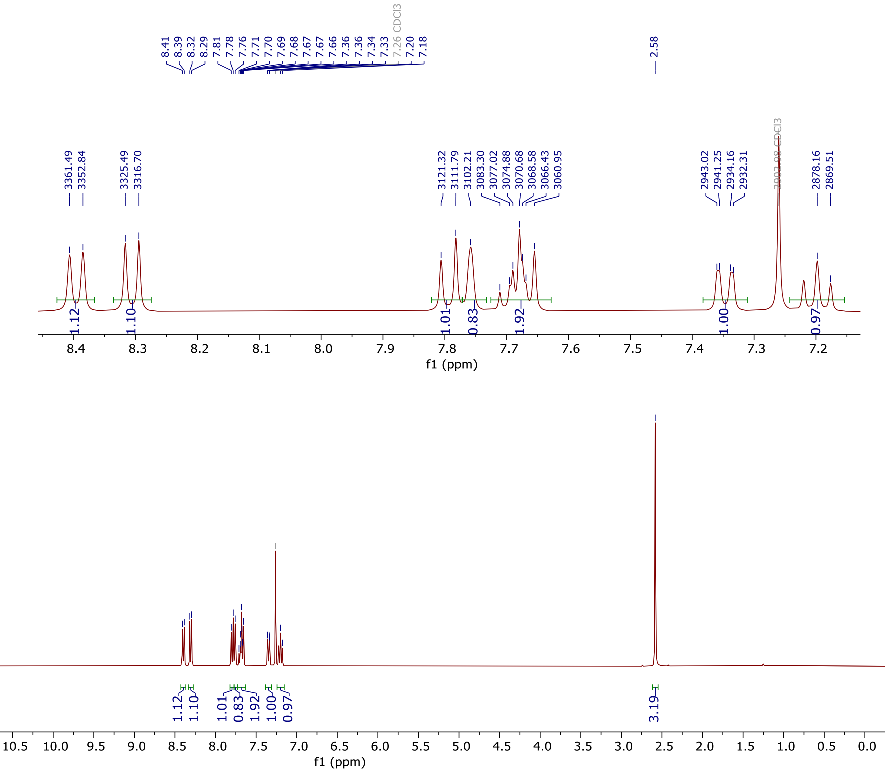

^{13}C{^{1}H} NMR spectrum (100 MHz, DMSO-d_6) of compound **5ae**

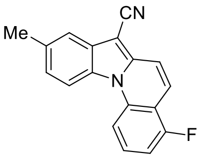

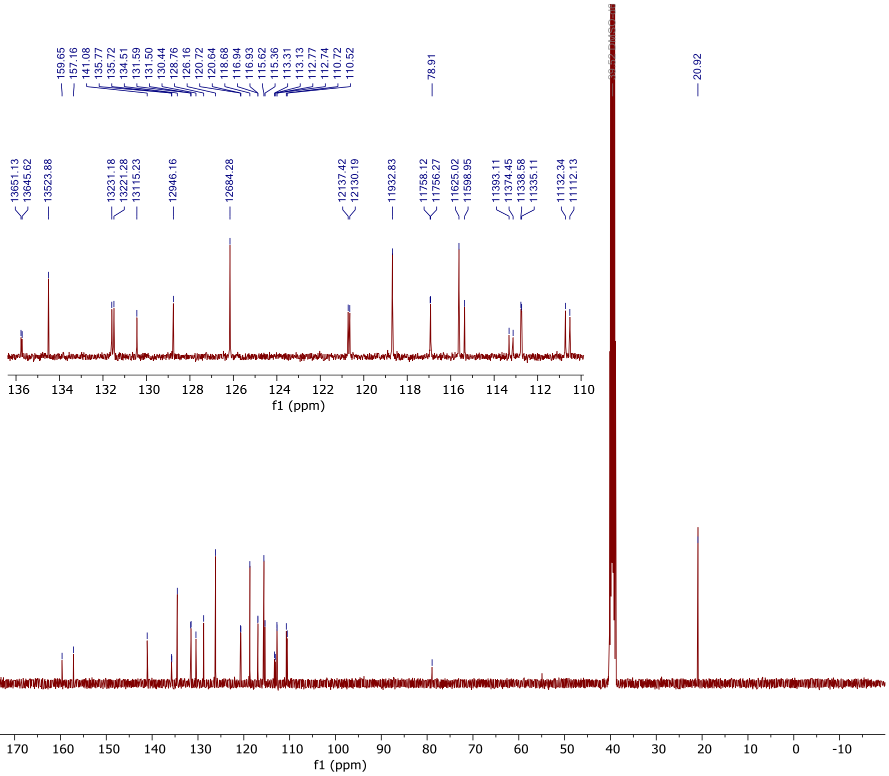

^{19}F NMR spectrum (470 MHz, CDCl$_3$) of compound **5ae**

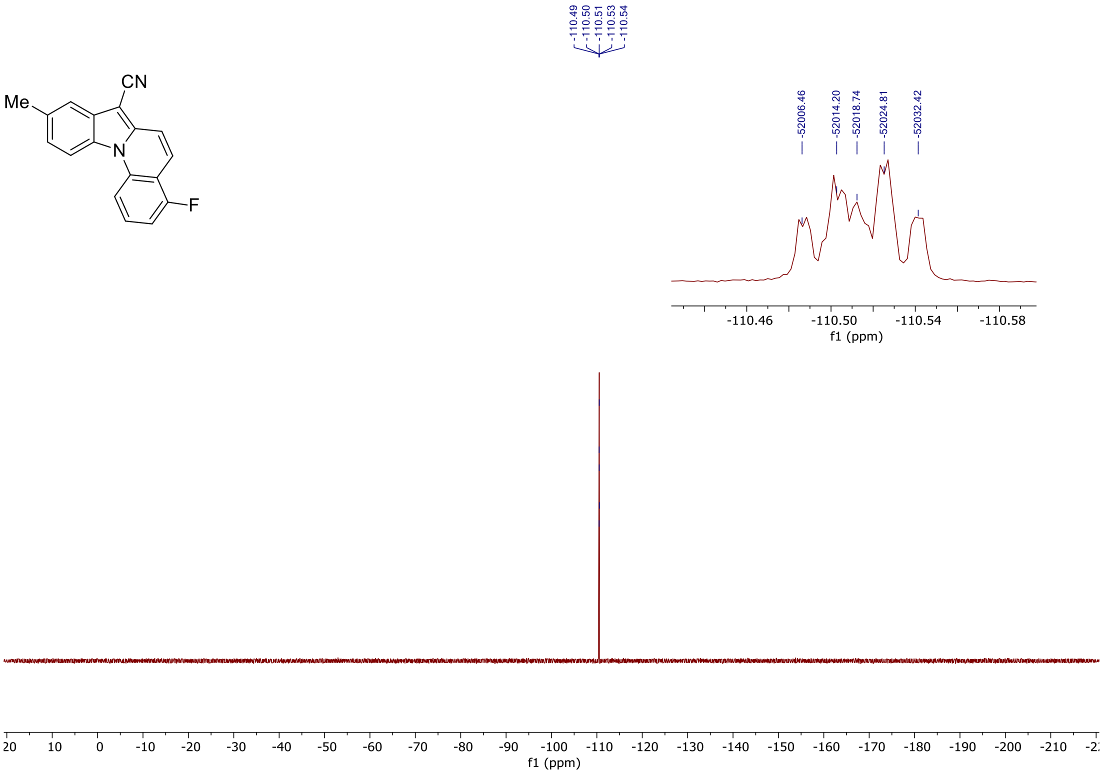

^{19}F NMR spectrum (470 MHz, CDCl$_3$) of compound **5ae** with an internal standard (fluorobenzene)

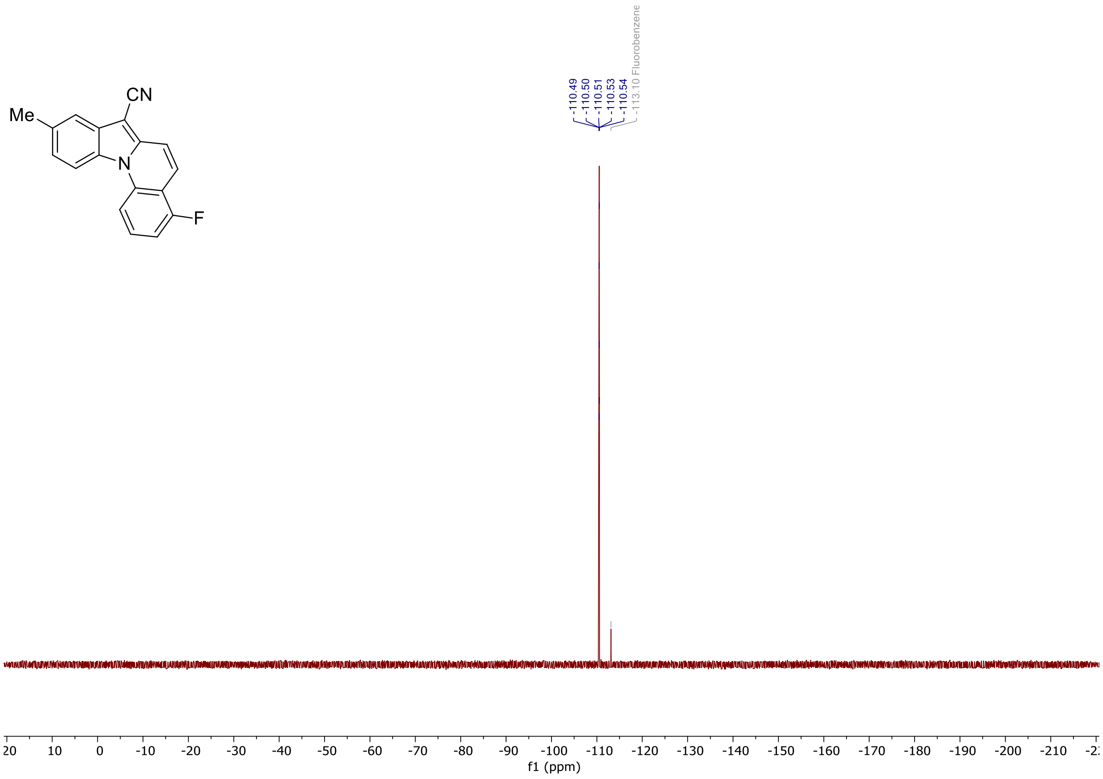

IR spectrum (neat) of compound **5ae**

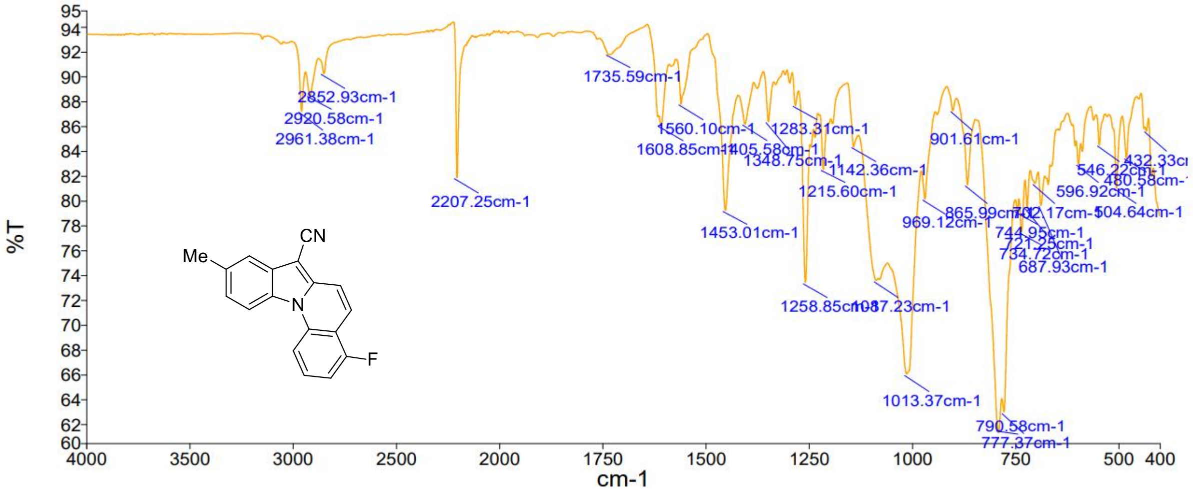

^{1}H NMR spectrum (400 MHz, DMSO-d_6) of compound of **5af**

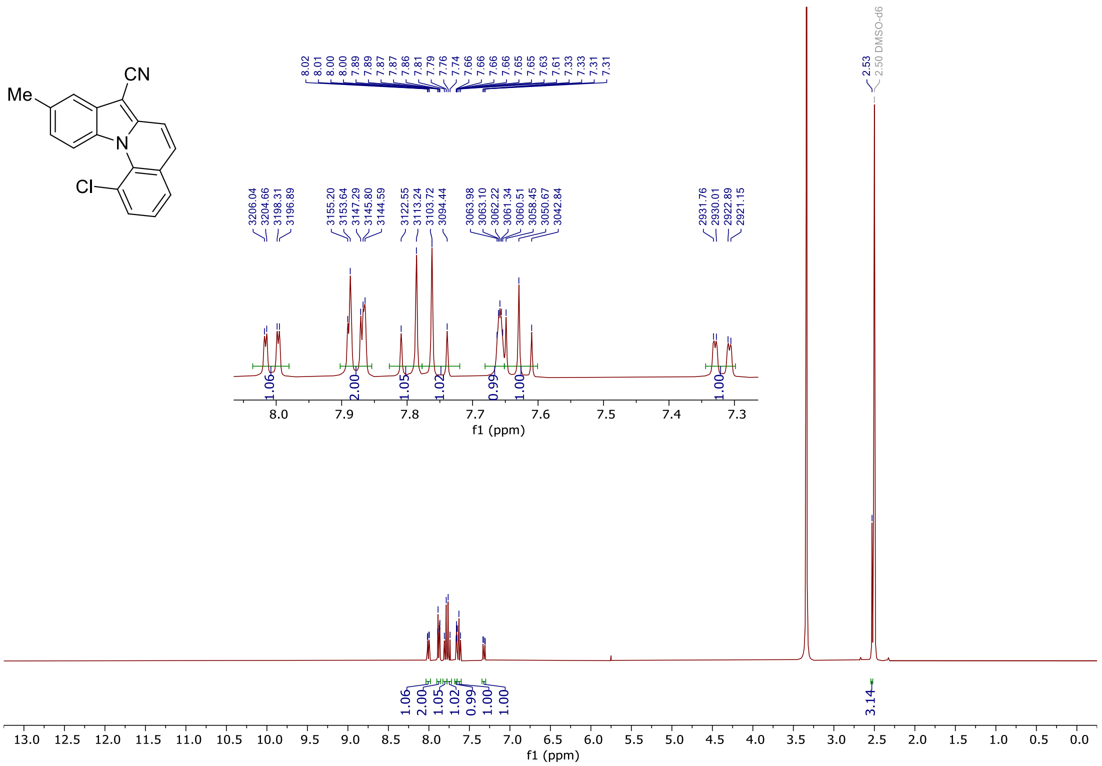

^{13}C{^{1}H} NMR spectrum (100 MHz, DMSO-d_6) of compound **5af**

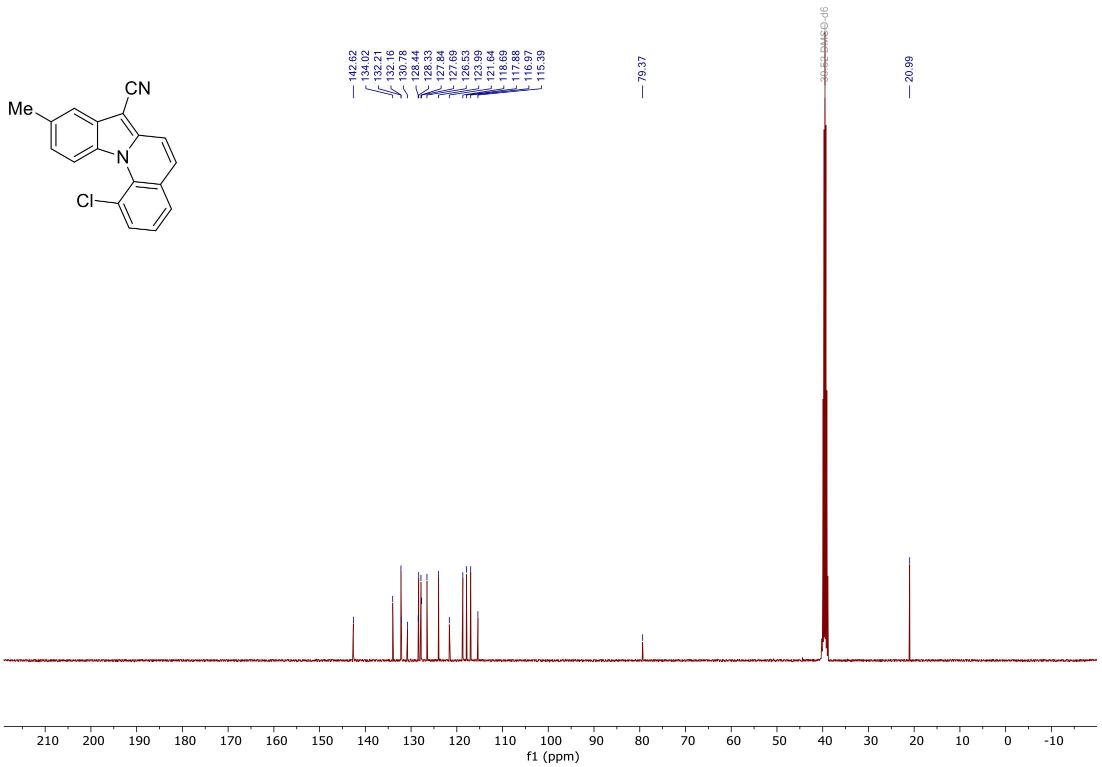

IR spectrum (neat) of compound **5af**

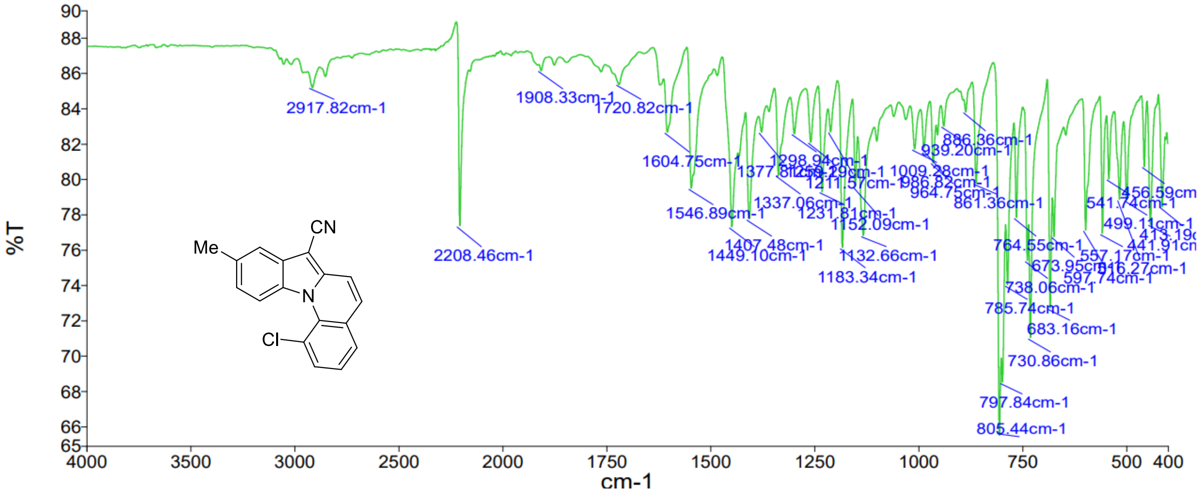

^{1}H NMR spectrum (400 MHz, $CDCl_3$) of compound **5ag**

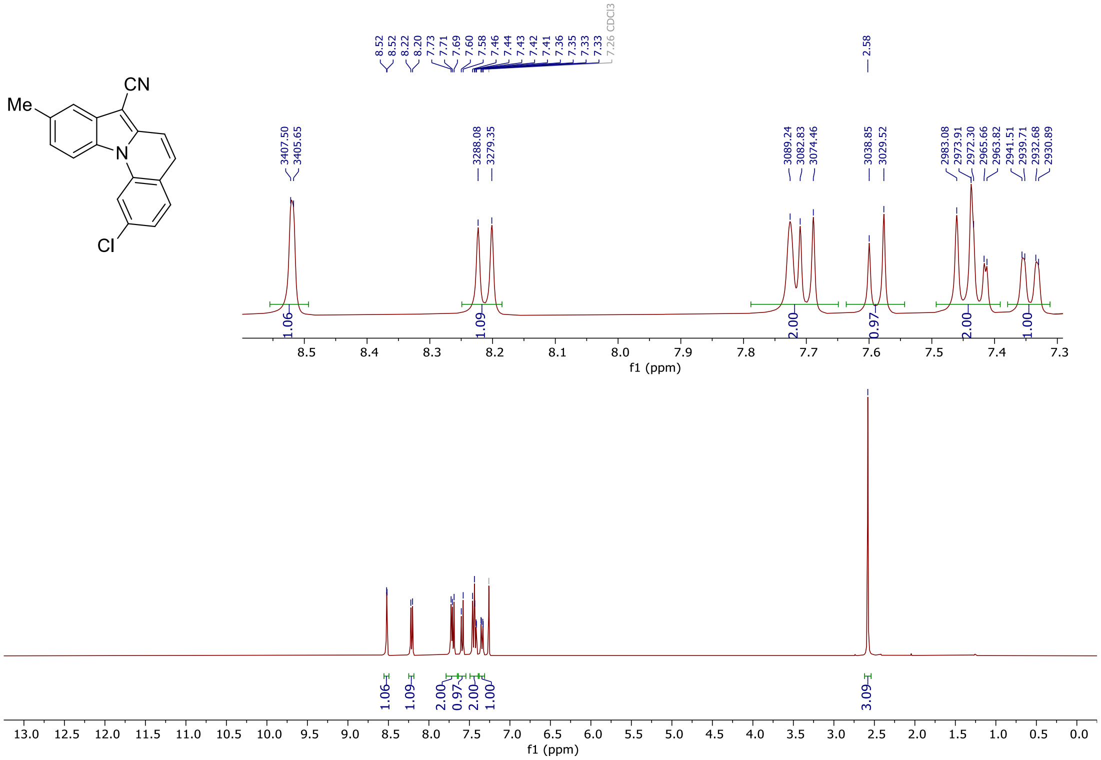

^{13}C{^{1}H} NMR spectrum (100 MHz, $CDCl_3$) of compound **5ag**

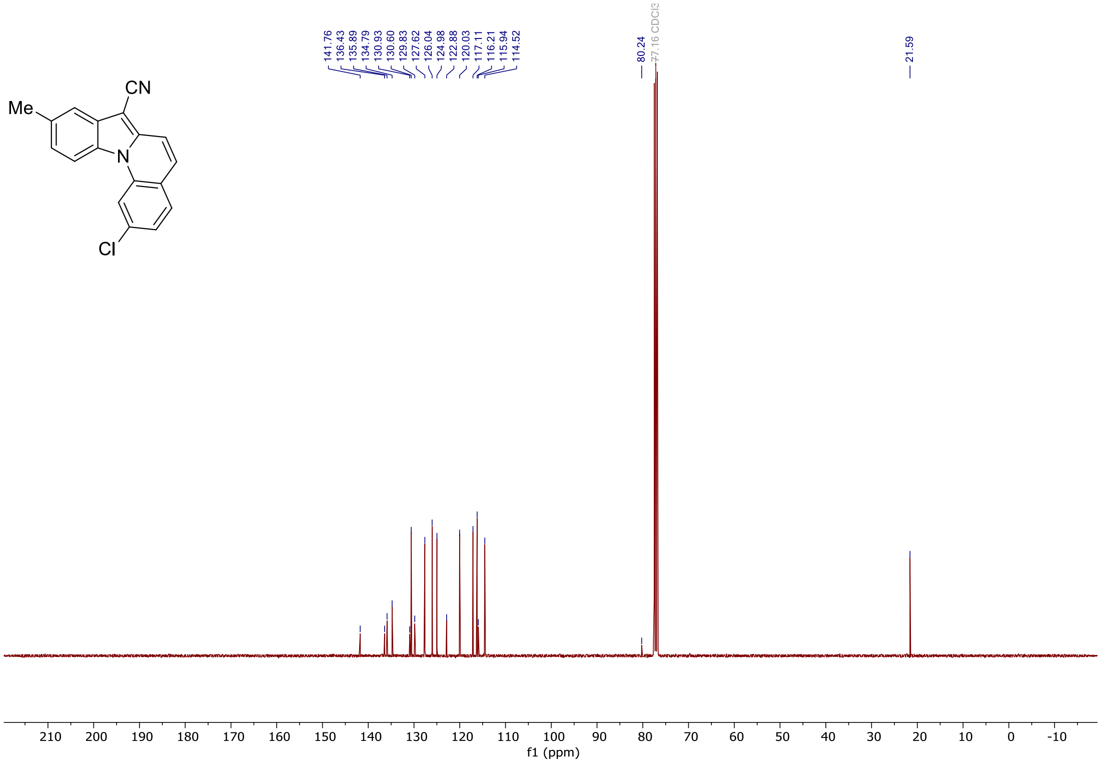

IR spectrum (neat) of compound **5ag**

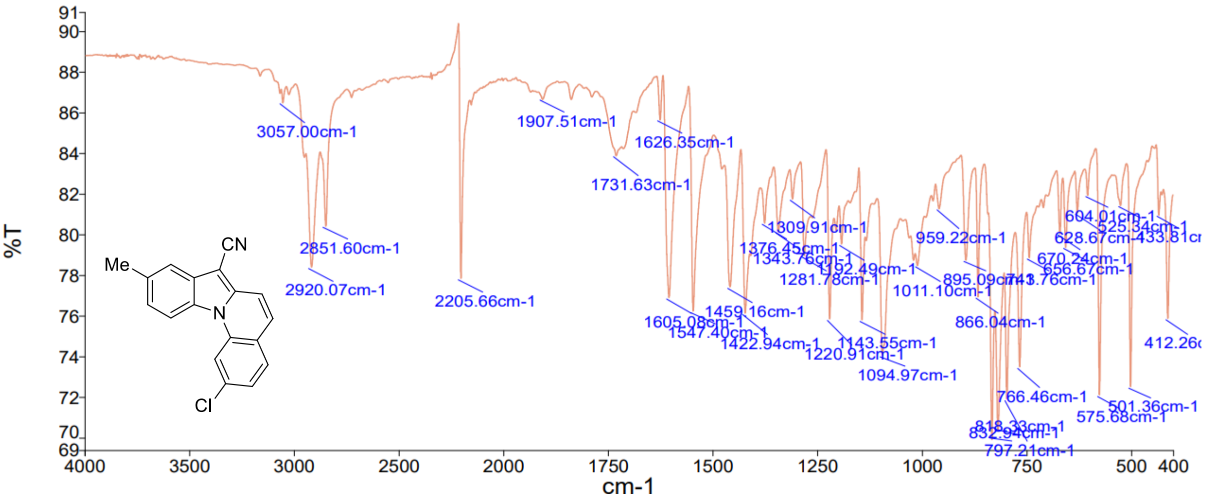

^{1}H NMR spectrum (400 MHz, $CDCl_3$) of compound **5ah**

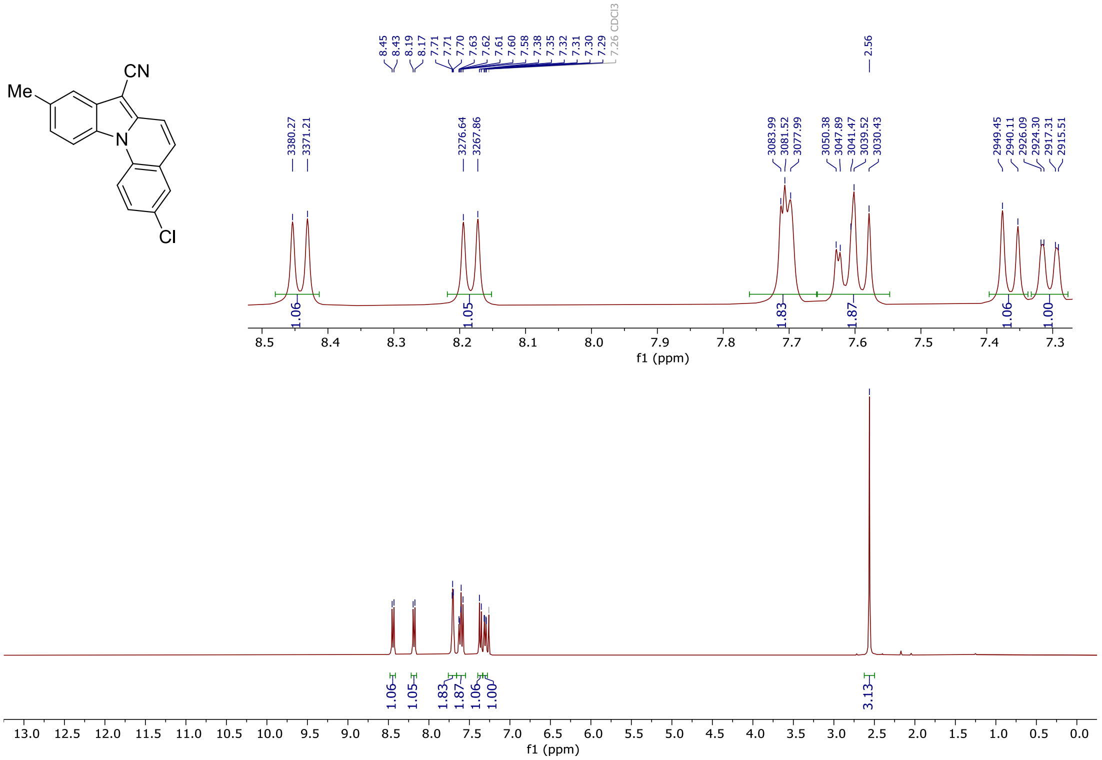

^{13}C{^{1}H} NMR spectrum (100 MHz, DMSO-d_6) of compound **5ah**

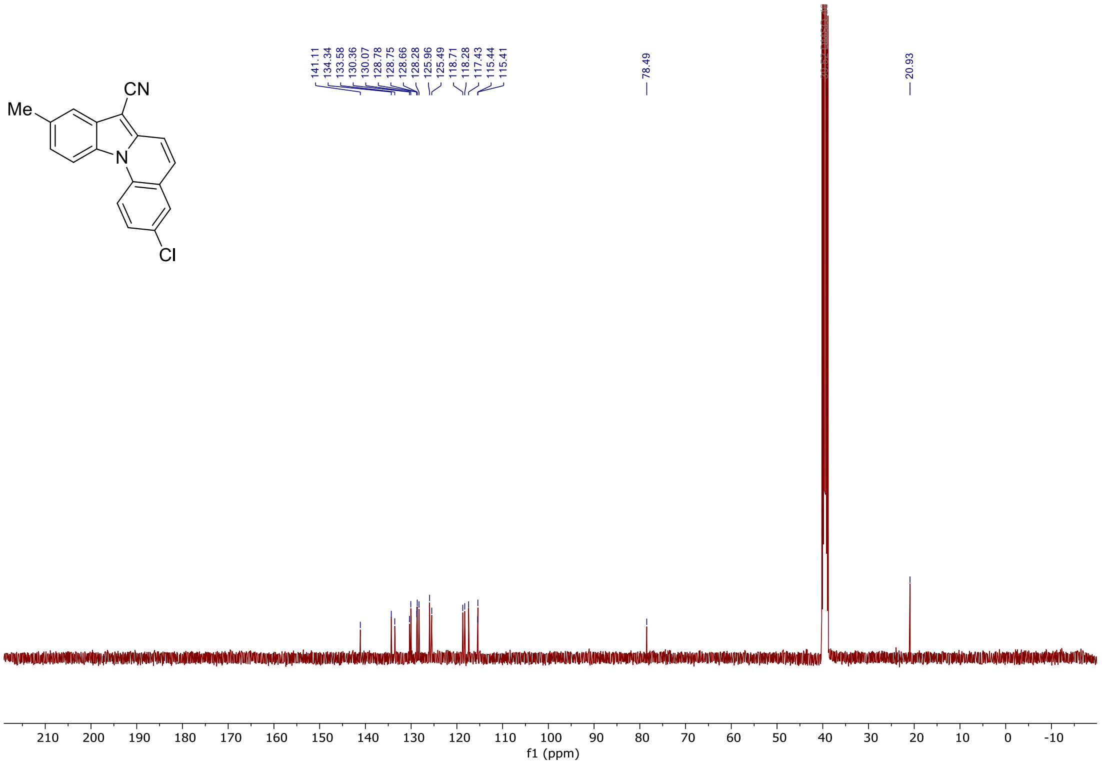

IR spectrum (neat) of compound **5ah**

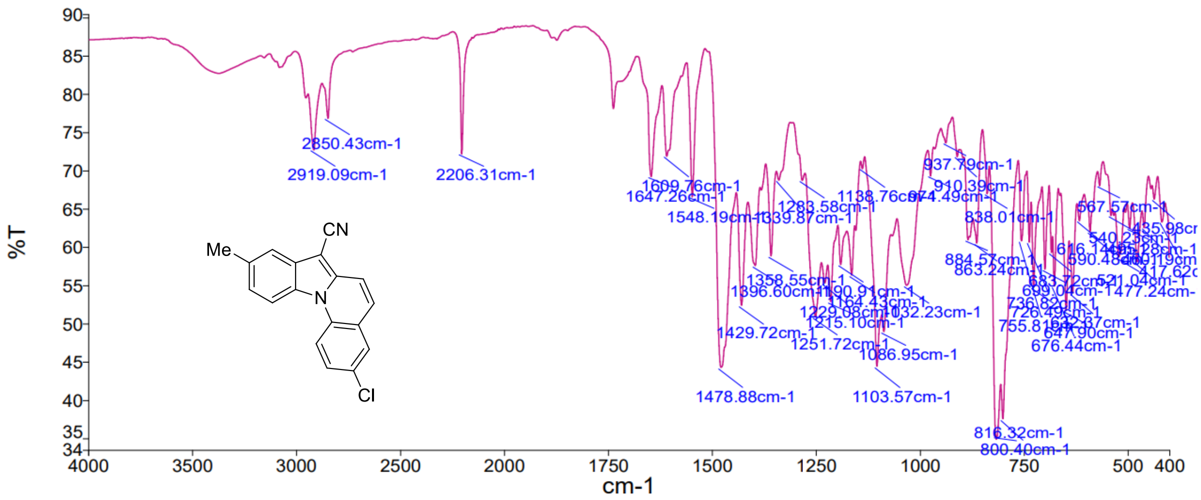

^{1}H NMR spectrum (400 MHz, $CDCl_3$) of compound **5ai**

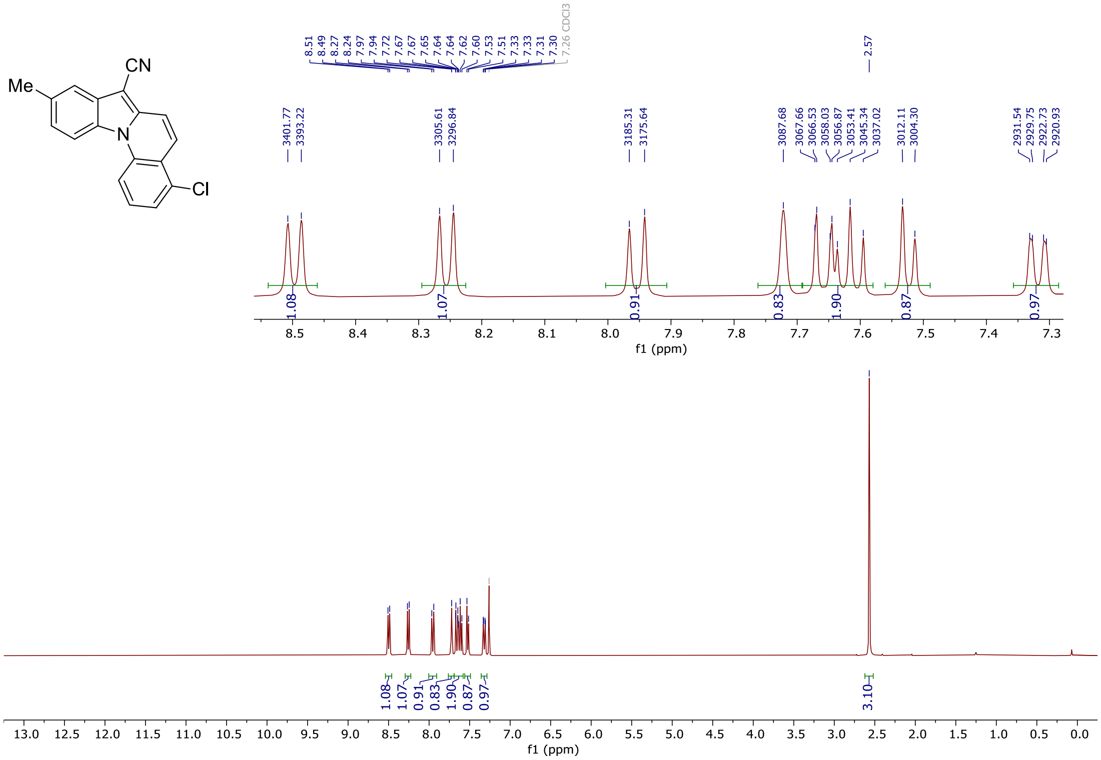

$^{13}C\{^{1}H\}$ NMR spectrum (100 MHz, DMSO-d_6) of compound **5ai**

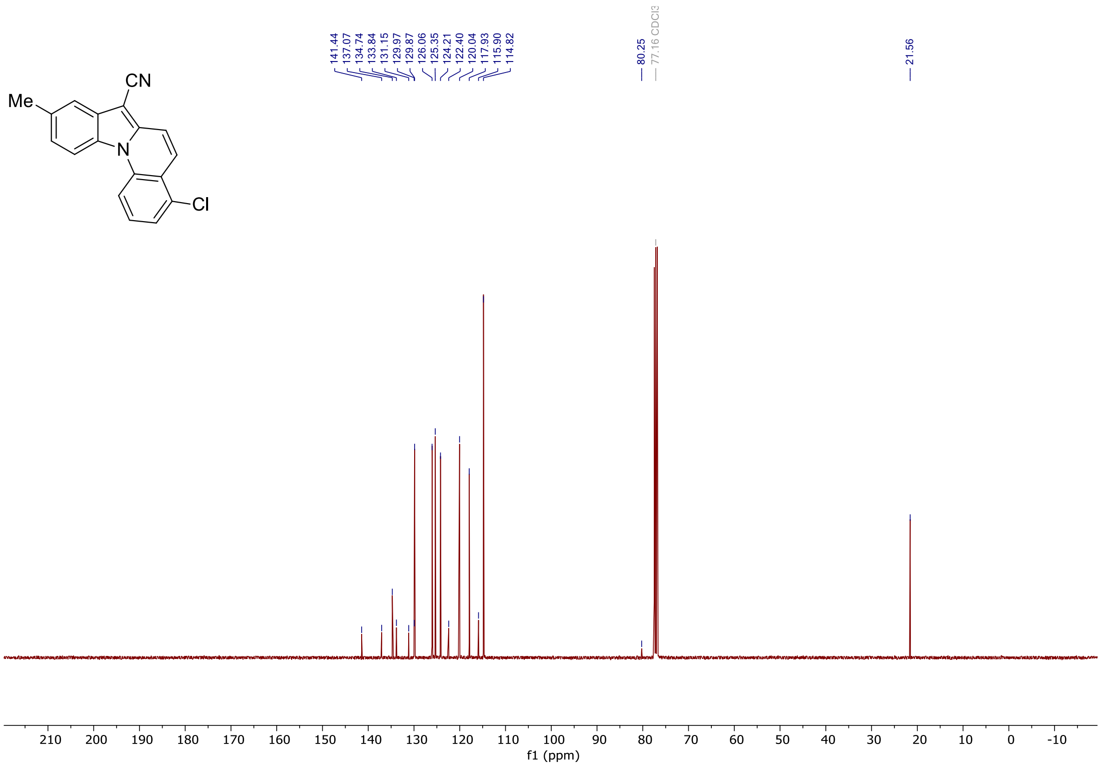

IR spectrum (neat) of compound **5ai**

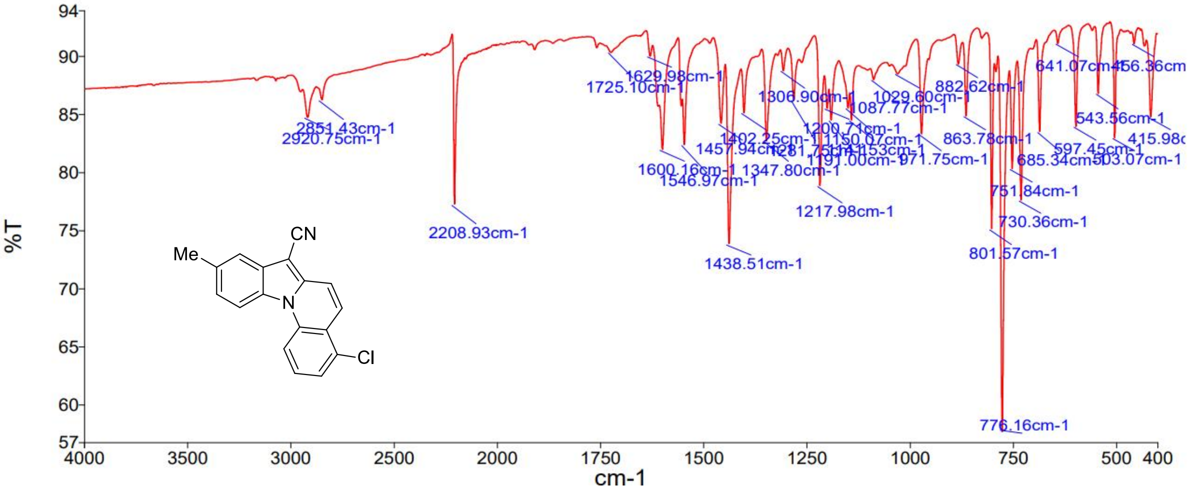

^{1}H NMR spectrum (400 MHz, DMSO-d_6) of compound **5aj**

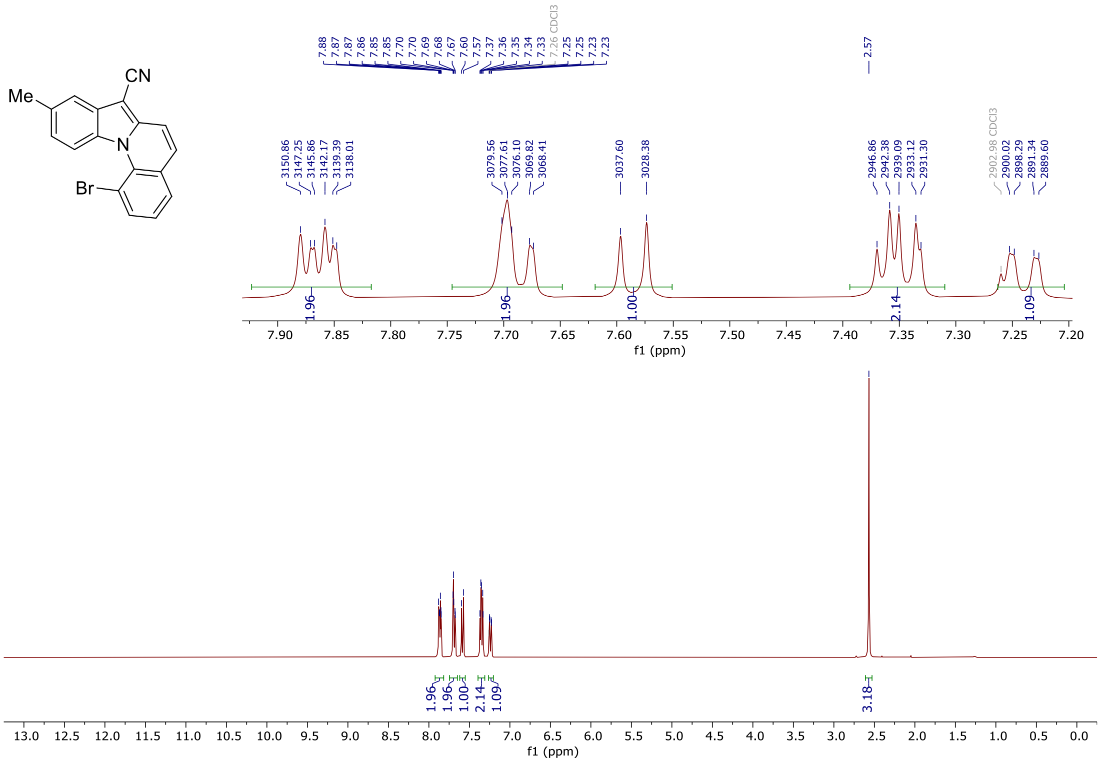

^{13}C{^{1}H} NMR spectrum (100 MHz, DMSO-d_6) of compound **5aj**

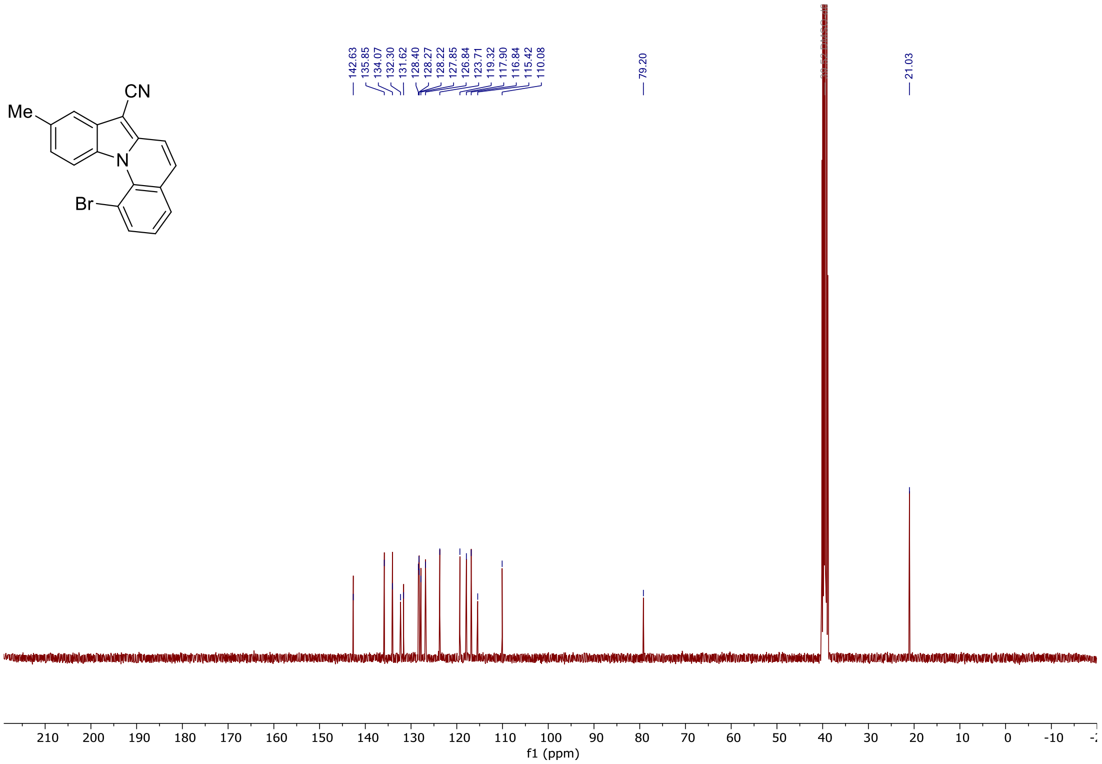

IR spectrum (neat) of compound **5aj**

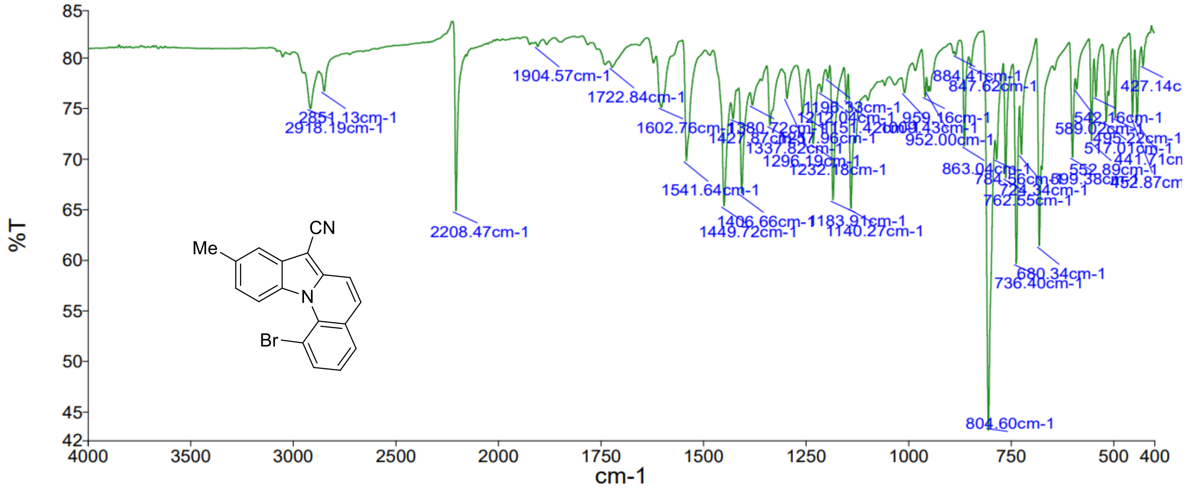

^{1}H NMR spectrum (400 MHz, $CDCl_3$) of compound **5ak**

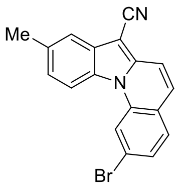

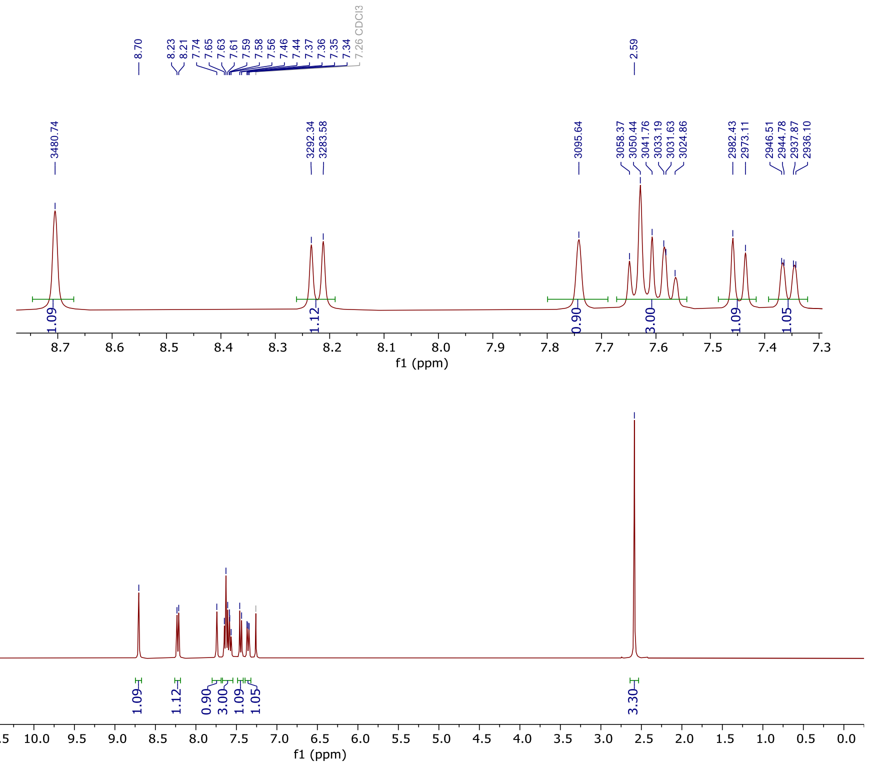

^{13}C{^{1}H} NMR spectrum (100 MHz, $CDCl_3$) od compound **5ak**

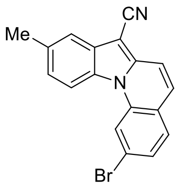

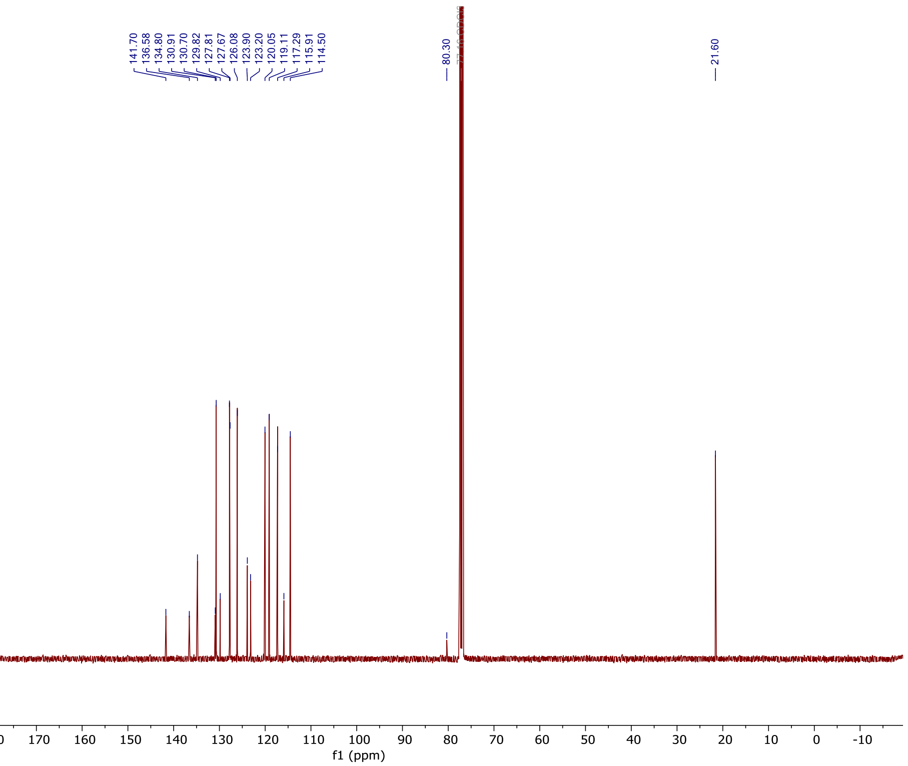

IR spectrum (neat) of compound **5ak**

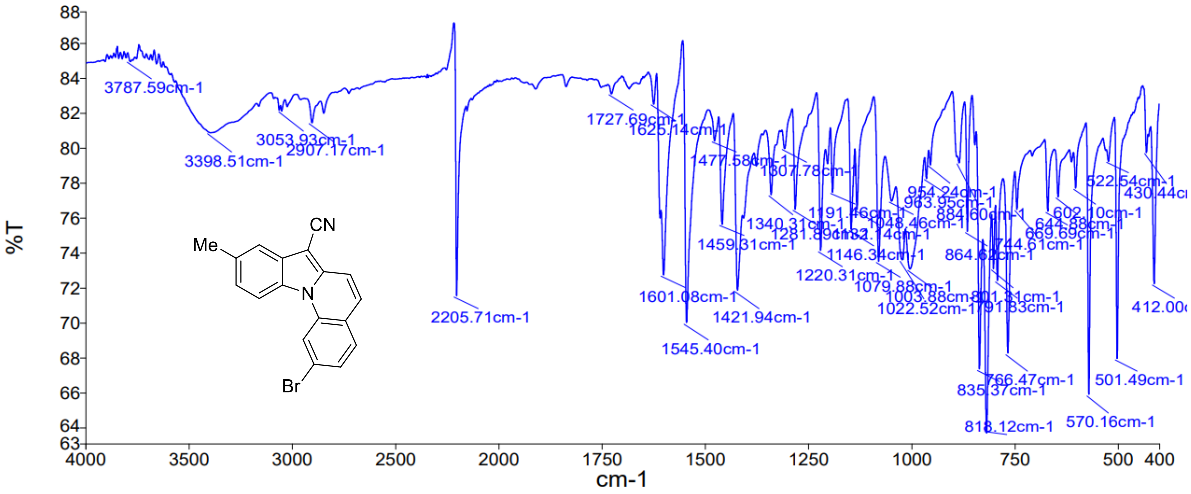

^1H NMR spectrum (400 MHz, DMSO-d_6) of compound **5al**

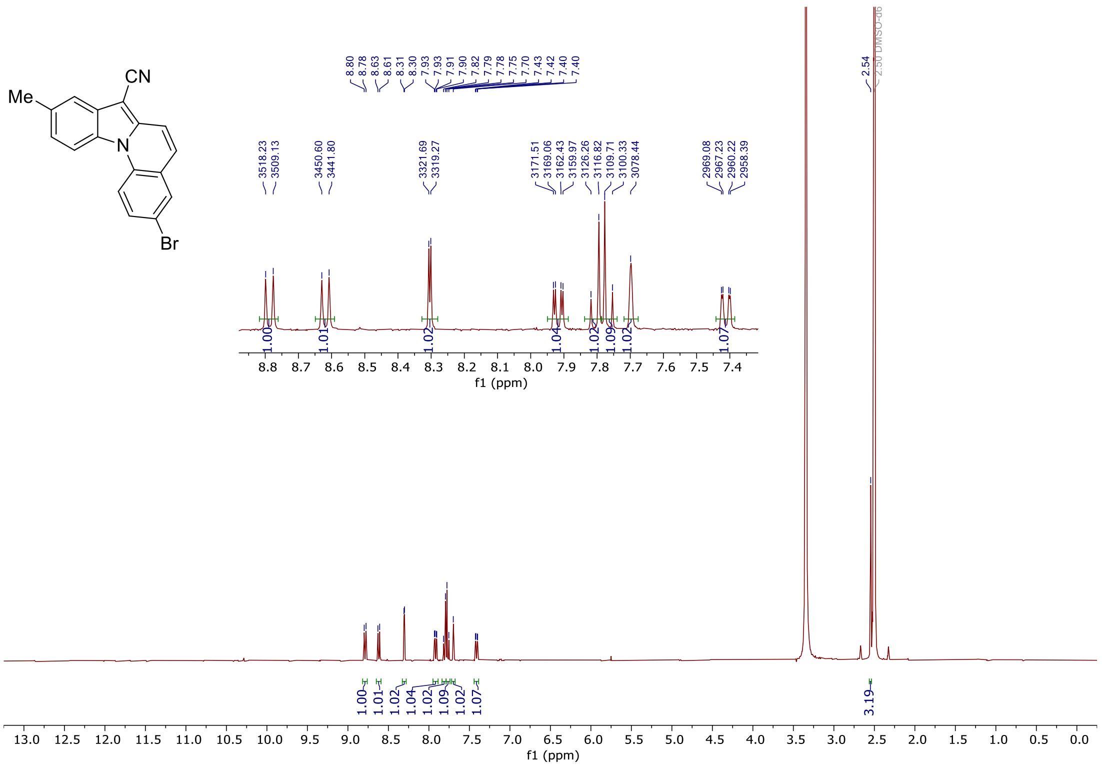

$^{13}C\{^1H\}$ NMR spectrum (100 MHz, DMSO-d_6) of compound **5al**

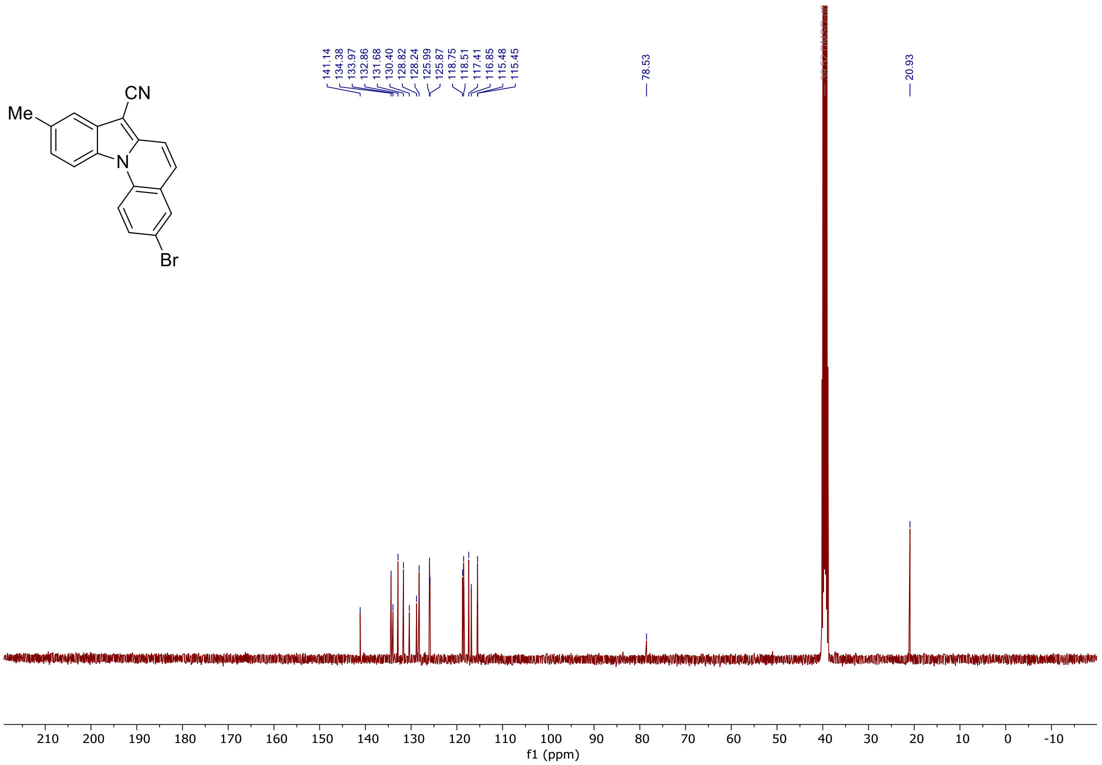

IR spectrum (neat) of compound **5al**

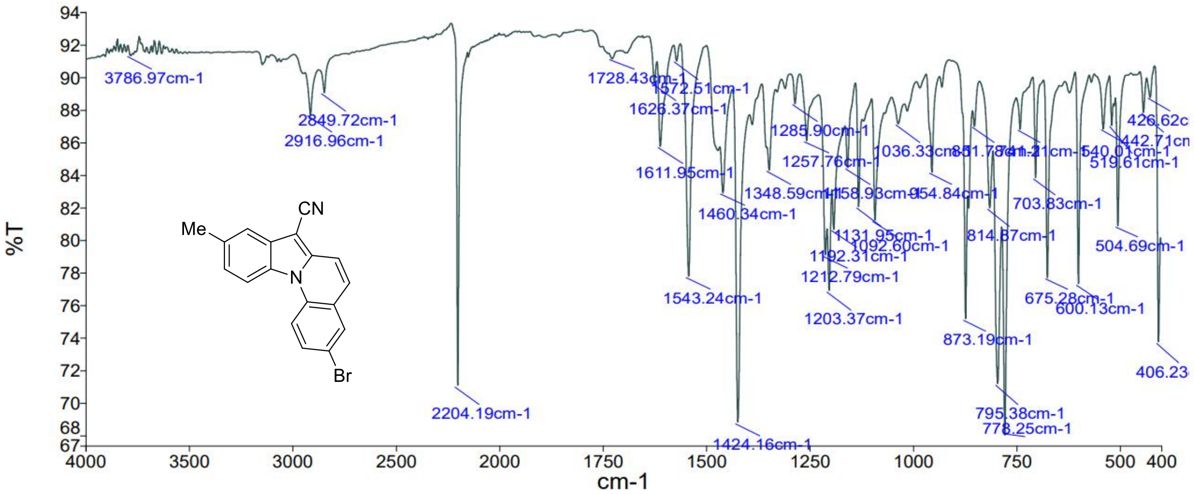

^{1}H NMR spectrum (400 MHz, $CDCl_3$) of compound **5am**

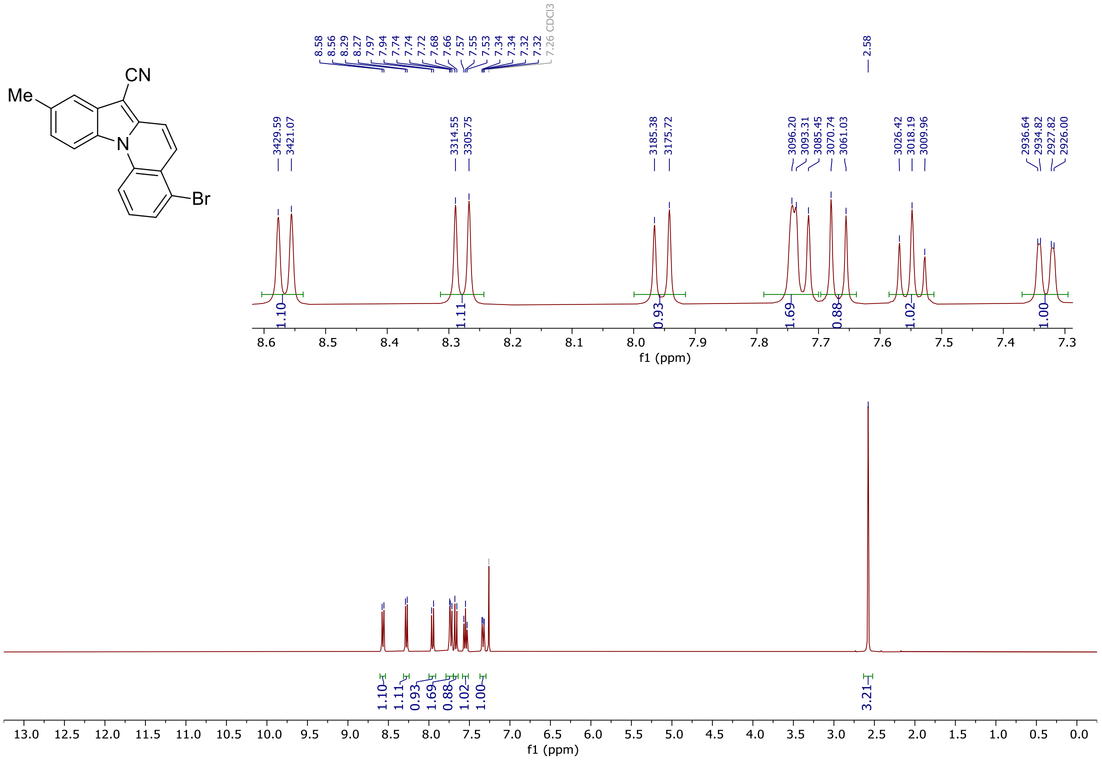

^{13}C{^{1}H} NMR spectrum (100 MHz, $CDCl_3$) of **5am**

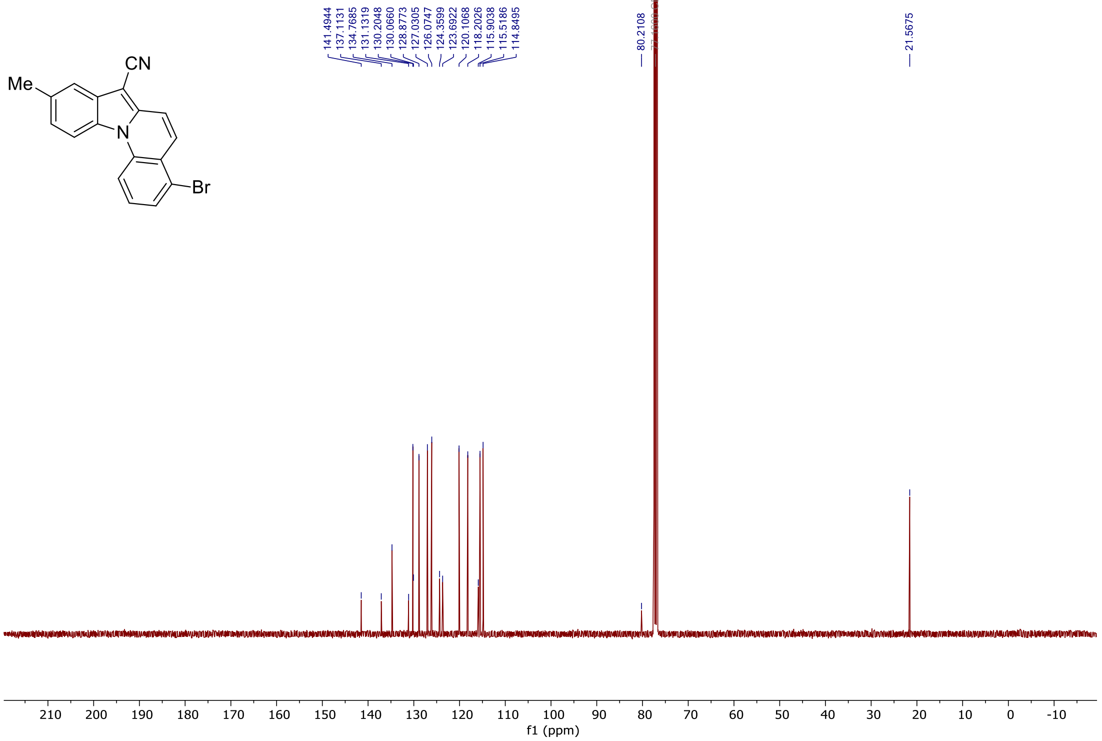

IR spectrum (neat) of compound **5am**

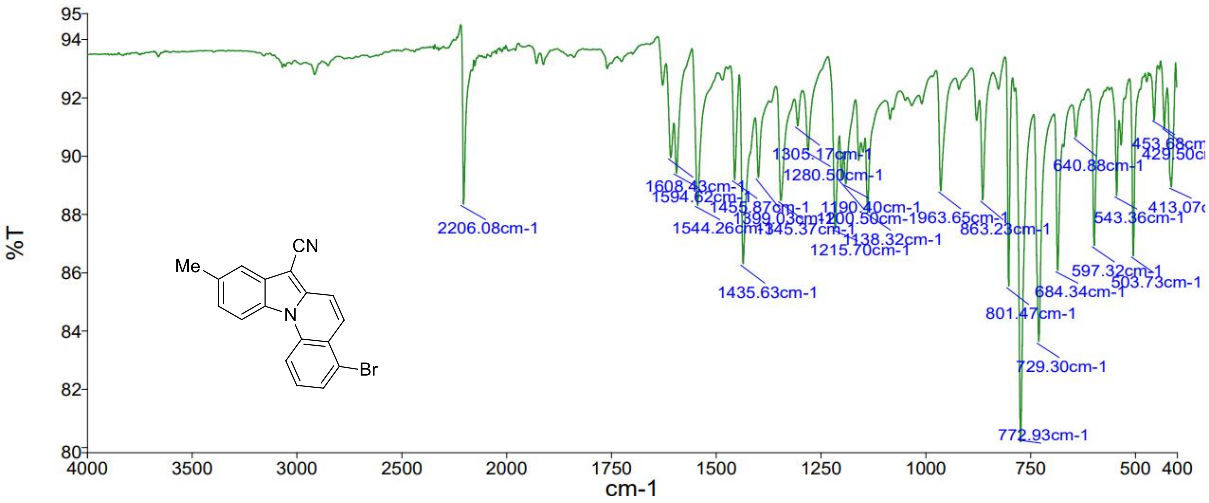

^{1}H NMR spectrum (400 MHz, $CDCl_3$) of compound **5an**

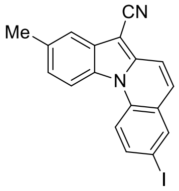

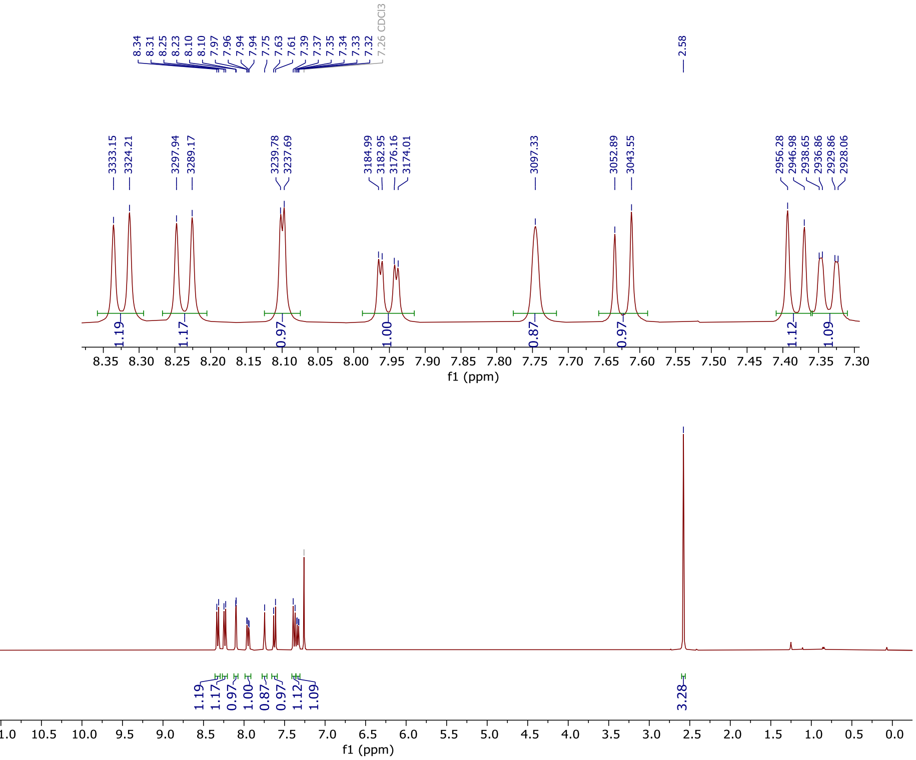

^{13}C{^{1}H} NMR spectrum (100 MHz, $CDCl_3$) of compound **5an**

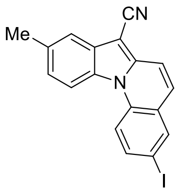

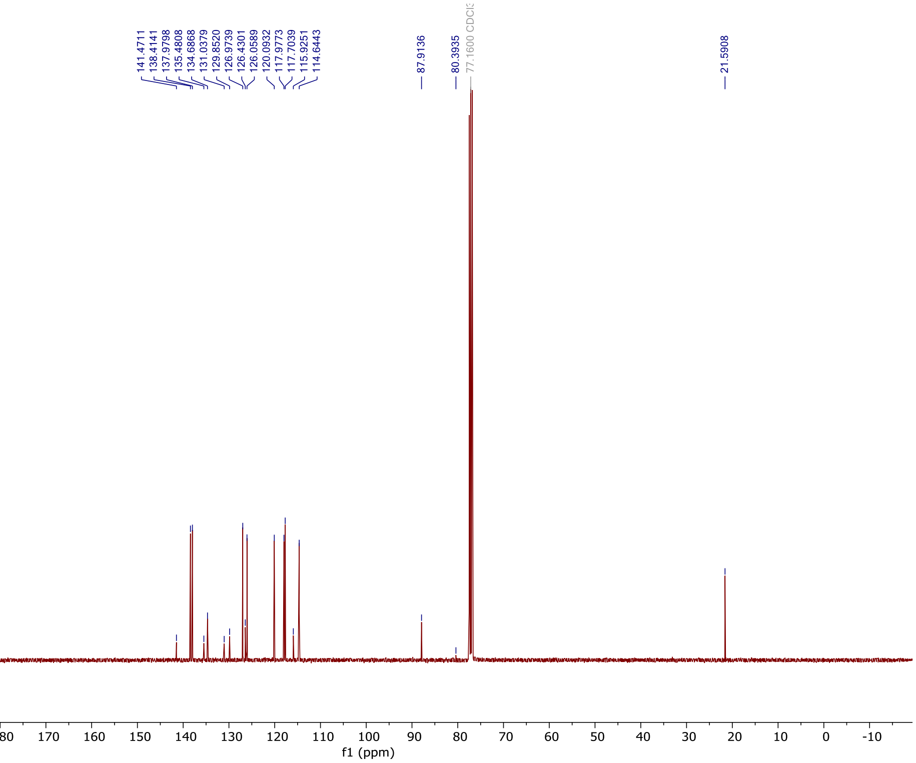

IR spectrum (neat) of compound **5an**

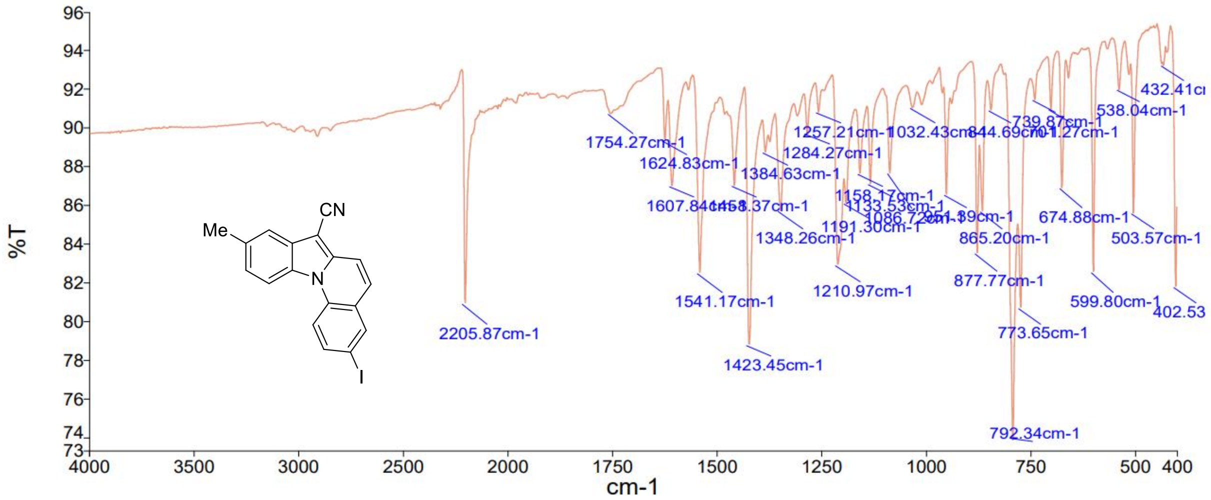

^{1}H NMR spectrum (400 MHz, $CDCl_3$) of compound **5ao**

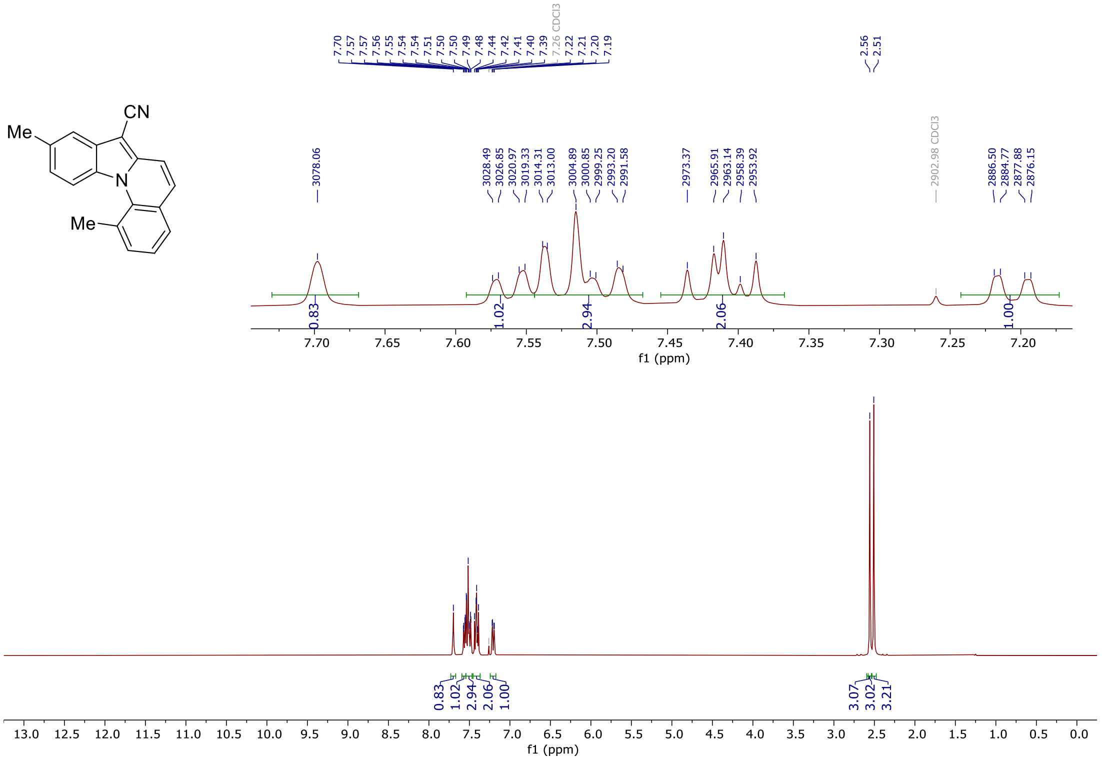

^{13}C{^{1}H} NMR spectrum (100 MHz, $CDCl_3$) of compound **5ao**

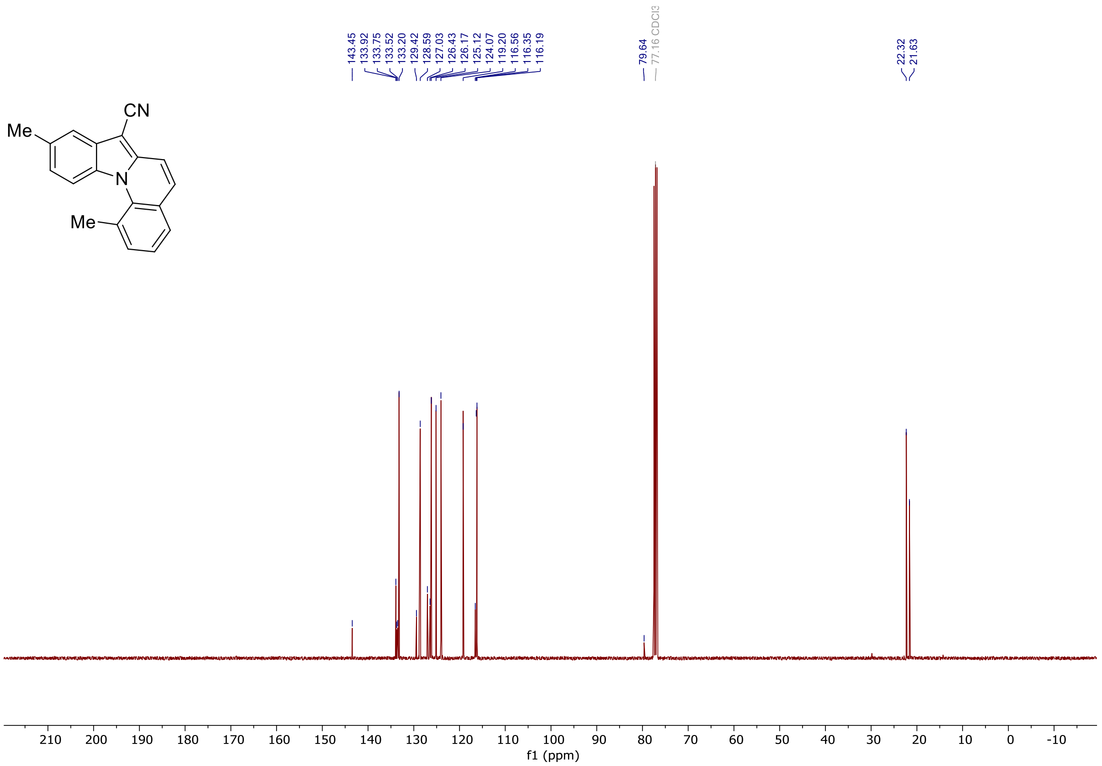

IR spectrum (neat) of compound **5ao**

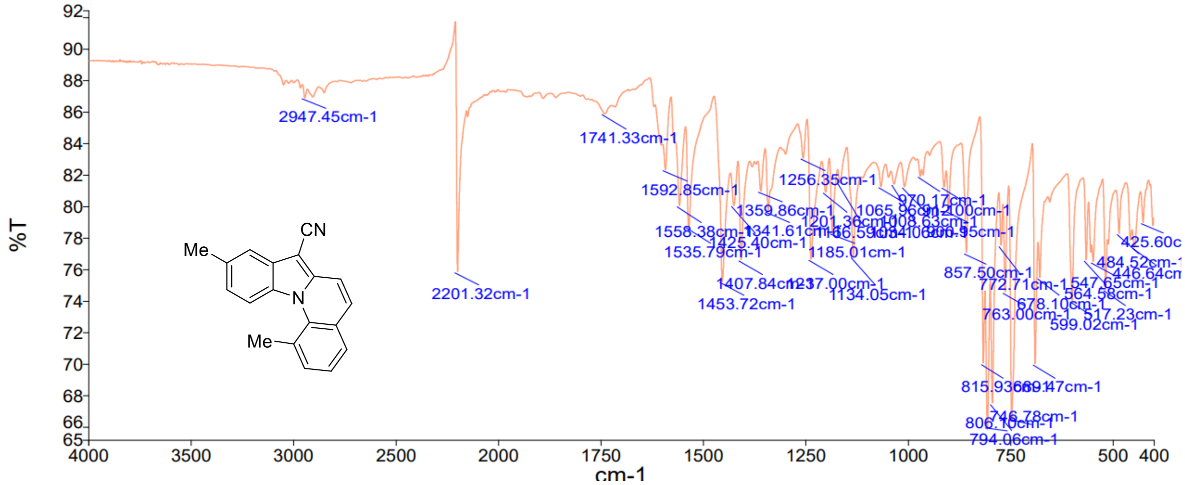

^{1}H NMR spectrum (400 MHz, $CDCl_3$) of compound **5ap**

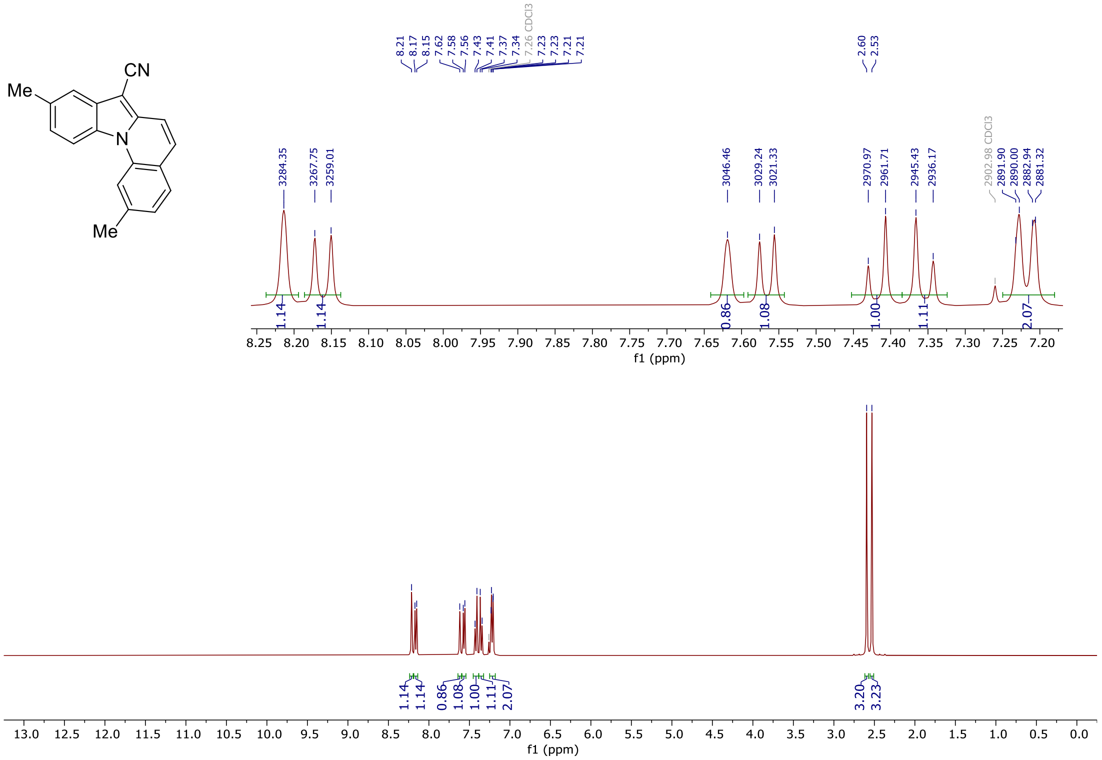

$^{13}C\{^{1}H\}$ NMR spectrum (100 MHz, $CDCl_3$) of compound **5ap**

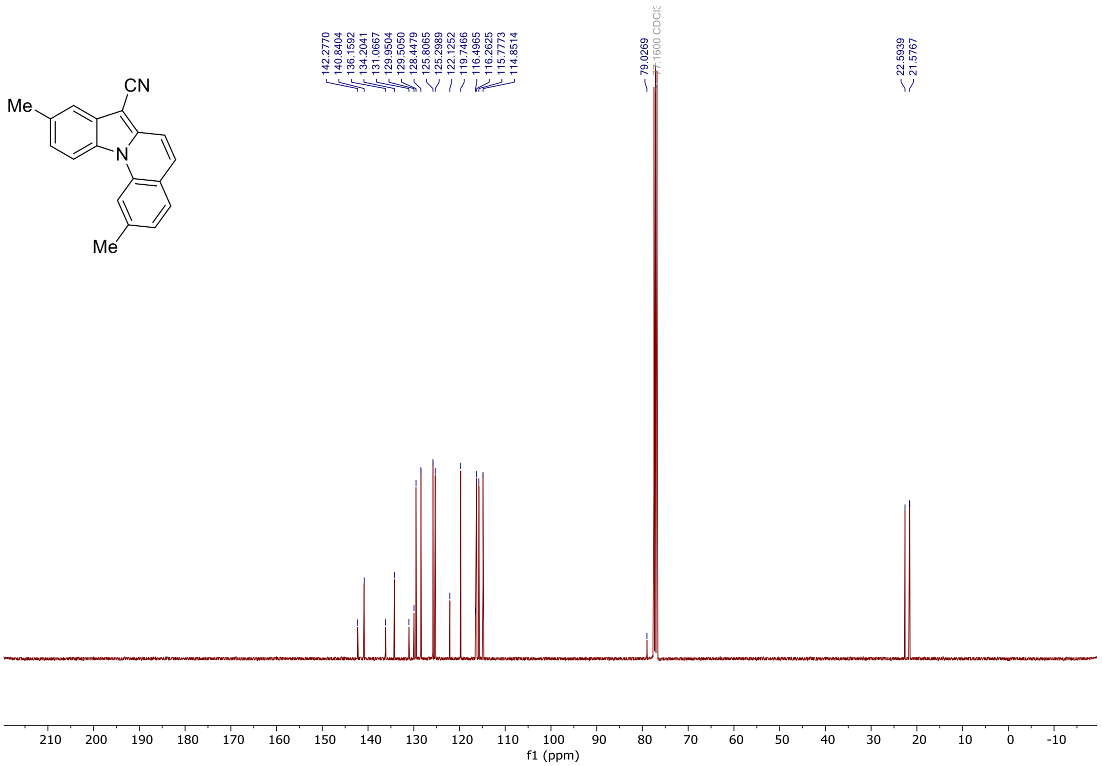

IR spectrum (neat) of compound **5ap**

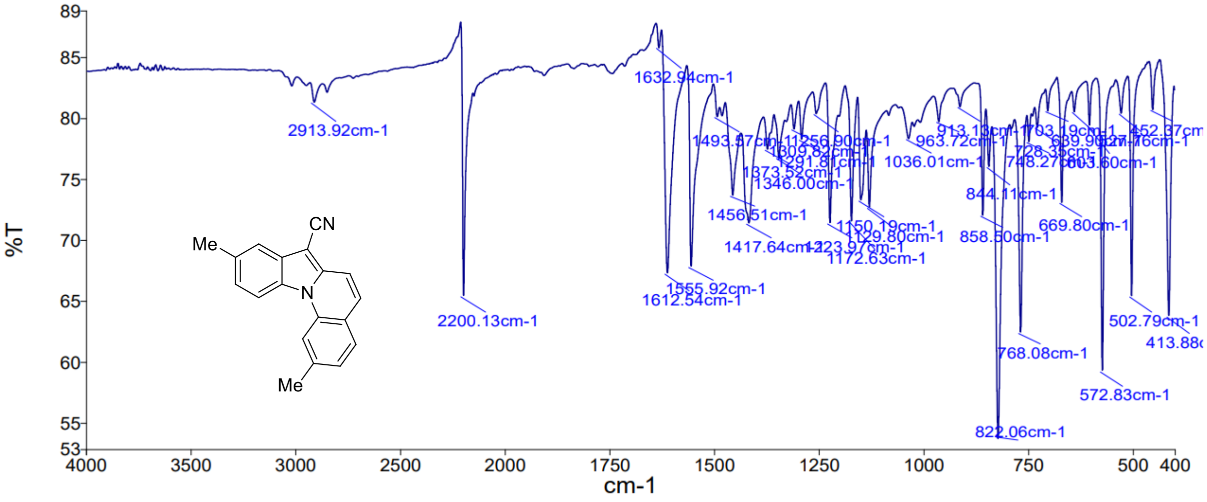

^{1}H NMR spectrum (400 MHz, DMSO-d_6) of compound **5aq**

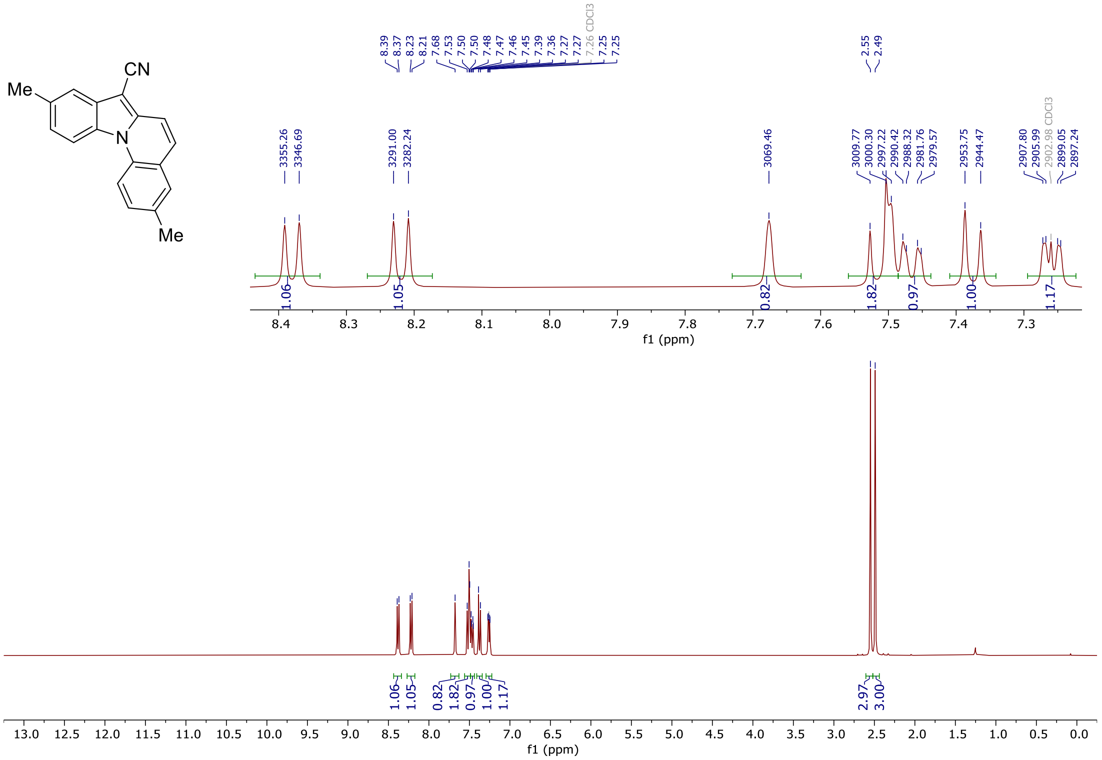

^{13}C{^{1}H} NMR spectrum (100 MHz, $CDCl_3$) of compound **5aq**

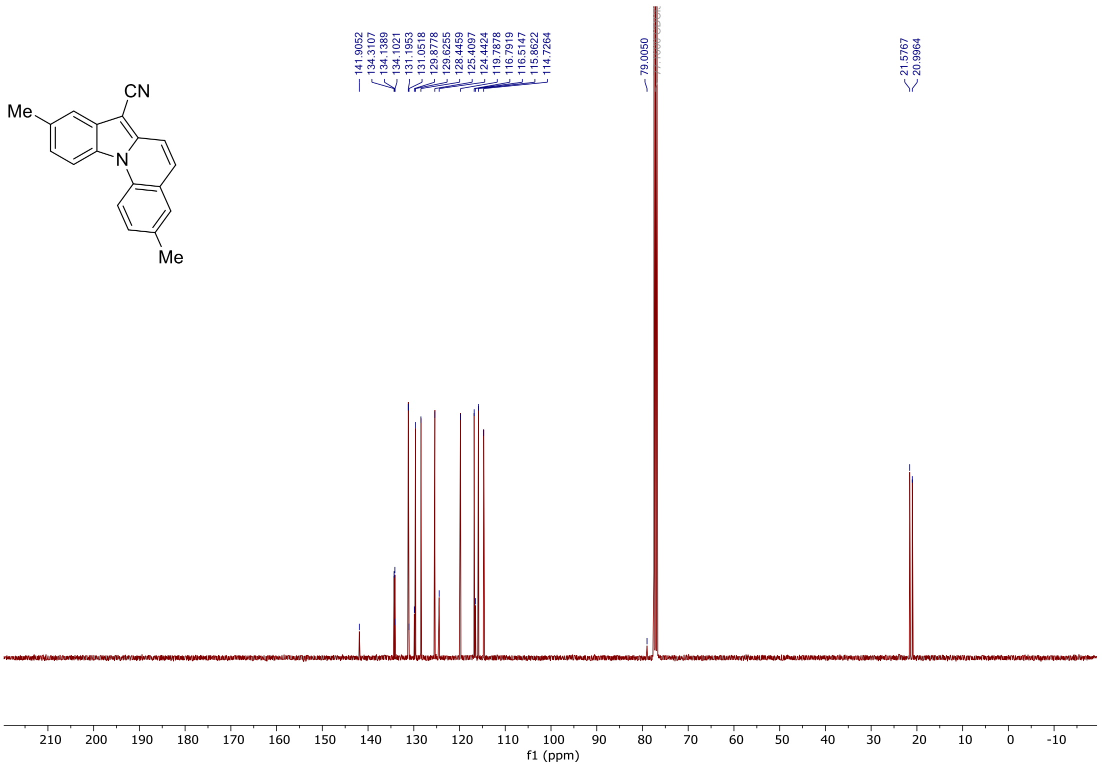

IR spectrum (neat) of compound **5aq**

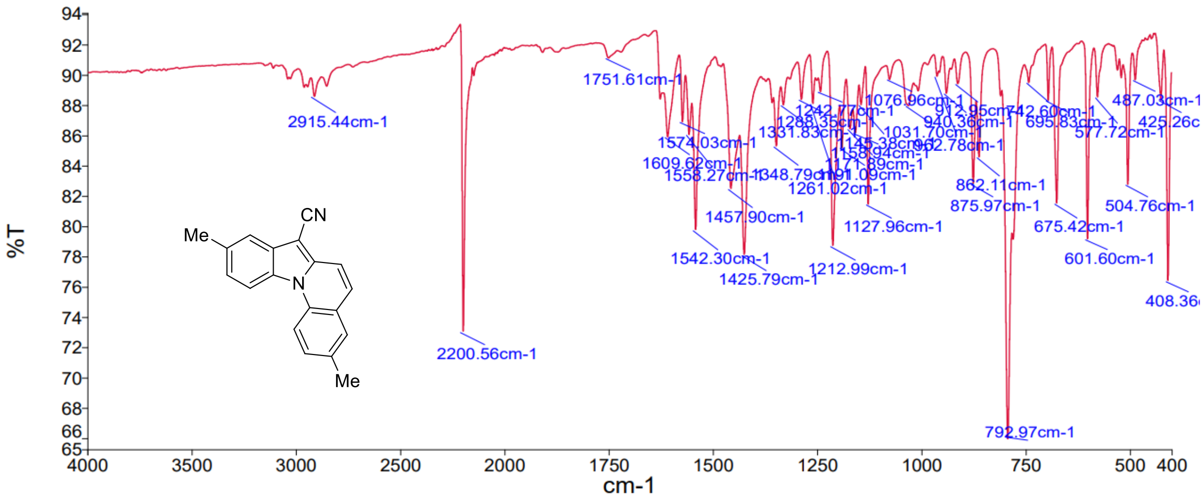

^{1}H NMR spectrum (400 MHz, $CDCl_3$) of compound **5ar**

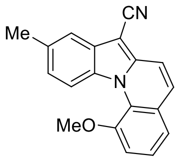

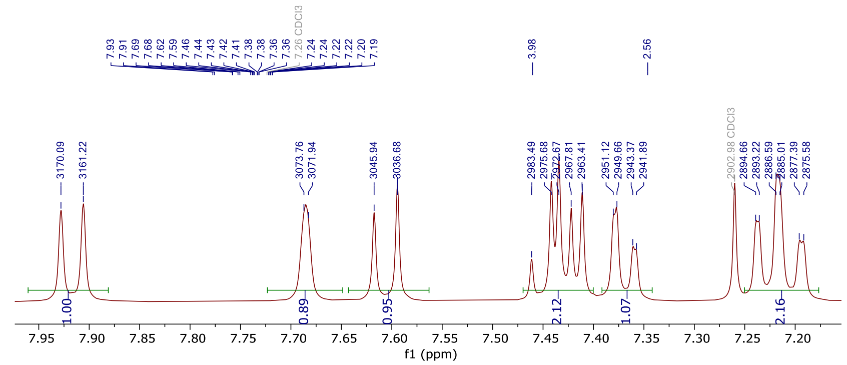

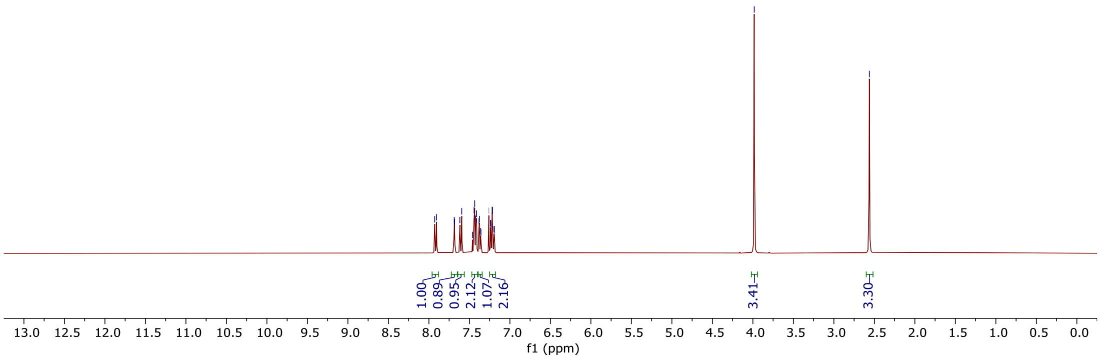

$^{13}C\{^{1}H\}$ NMR spectrum (100 MHz, $CDCl_3$) of compound **5ar**

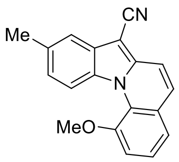

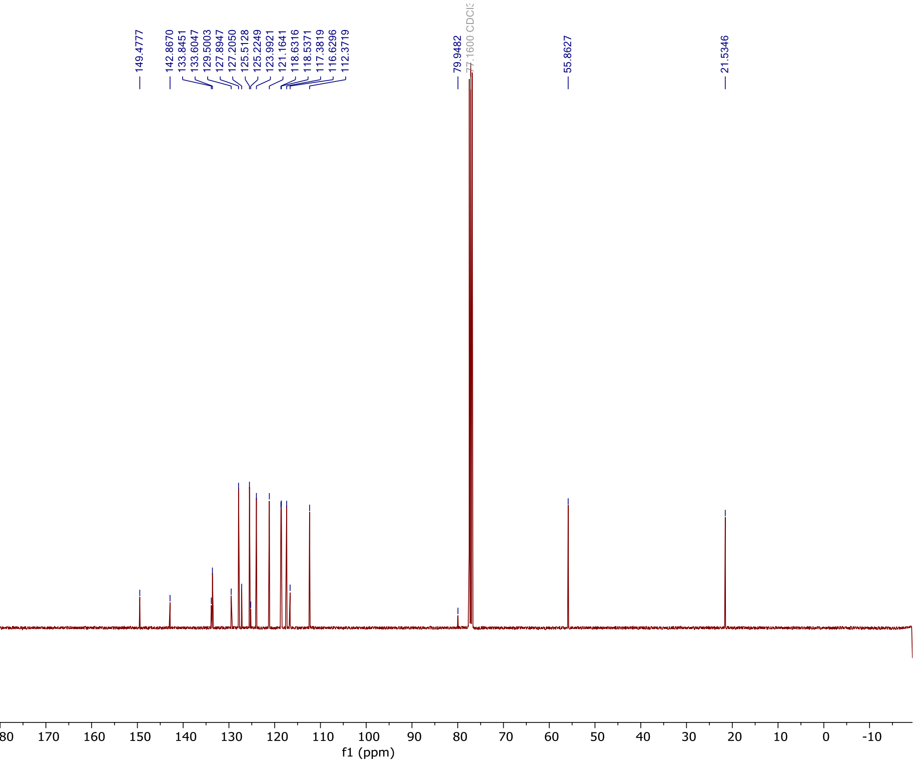

IR spectrum (neat) of compound **5ar**

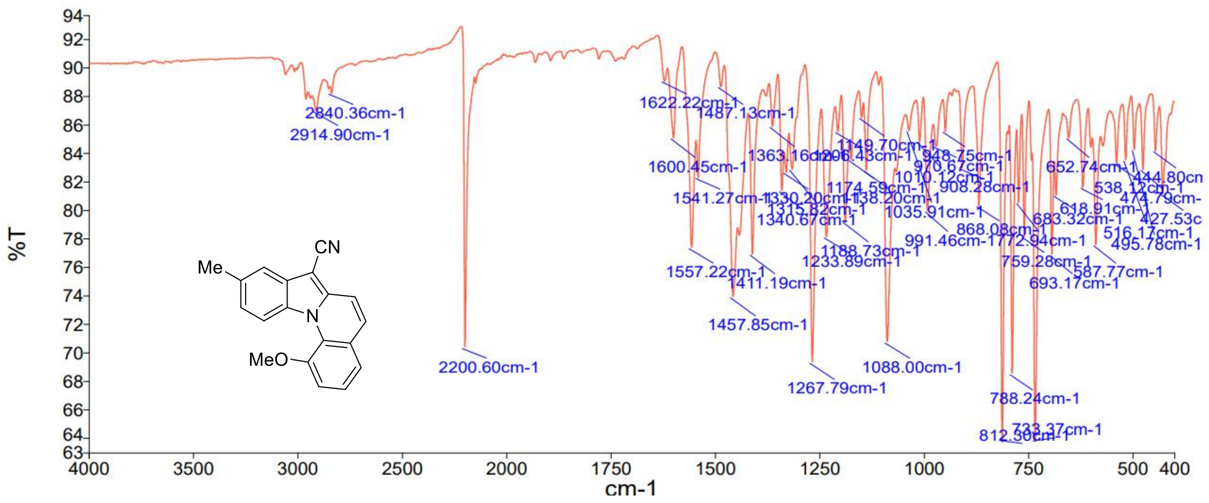

^{1}H NMR spectrum (400 MHz, $CDCl_3$) of compound **5as**

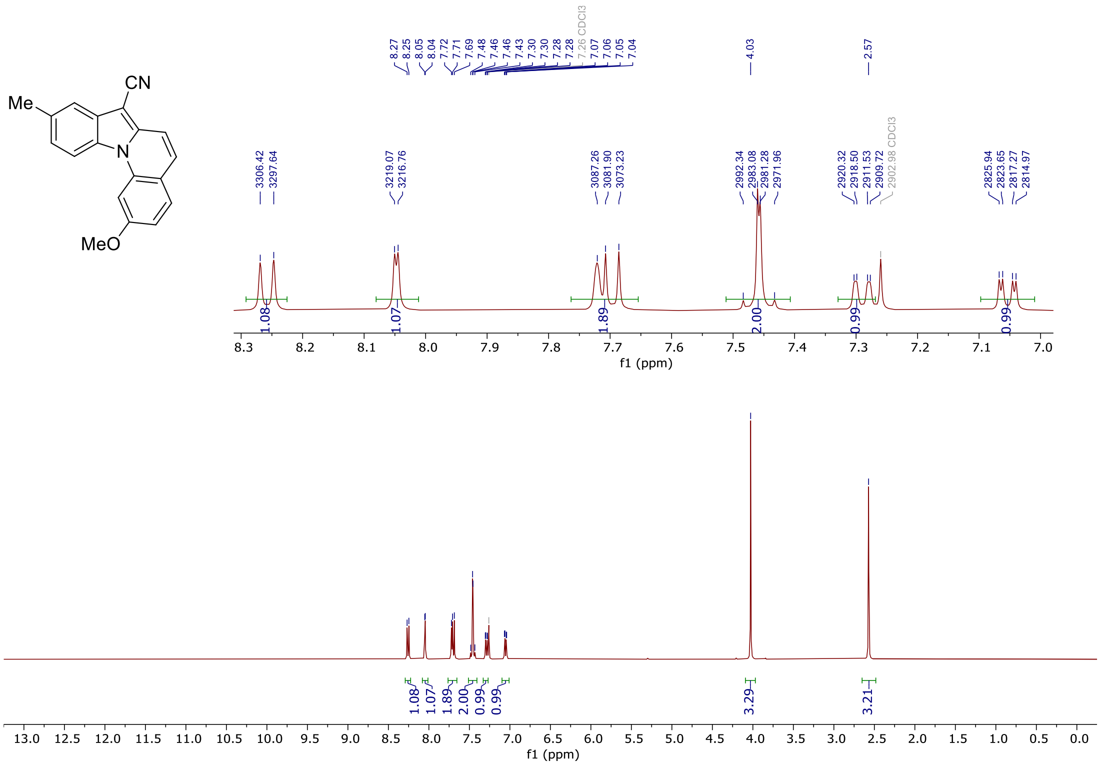

^{13}C{^{1}H} NMR spectrum (100 MHz, $CDCl_3$) of compound **5as**

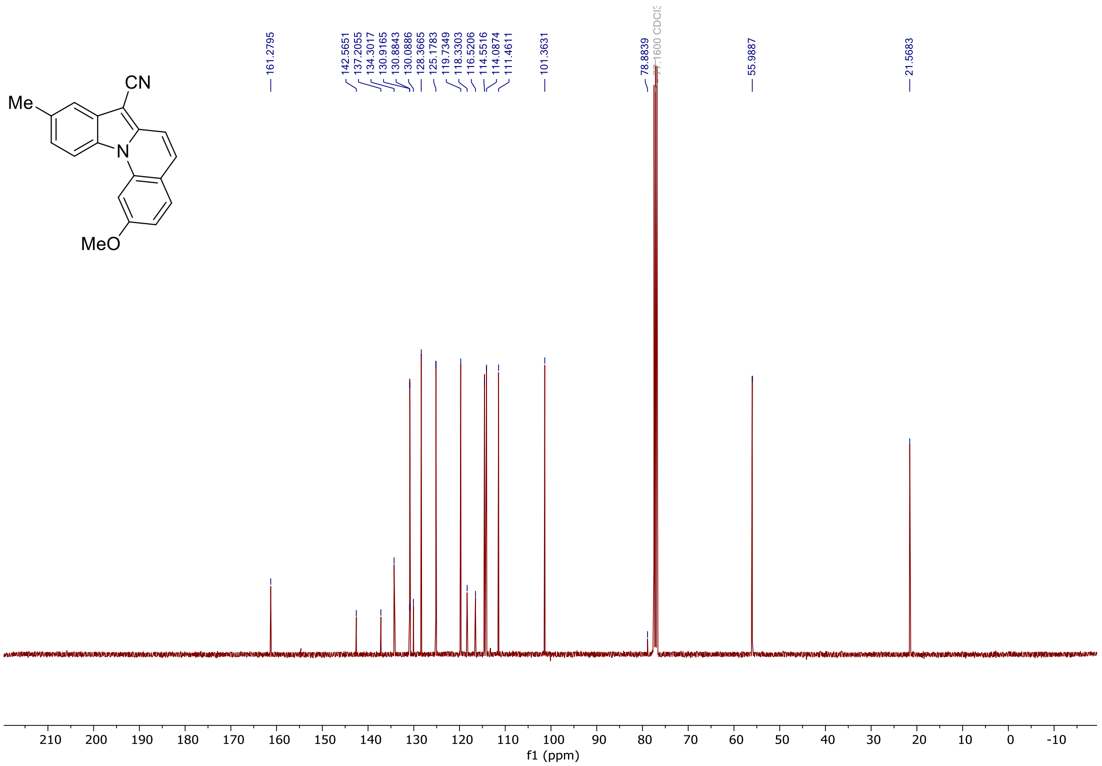

IR spectrum (neat) of compound **5as**

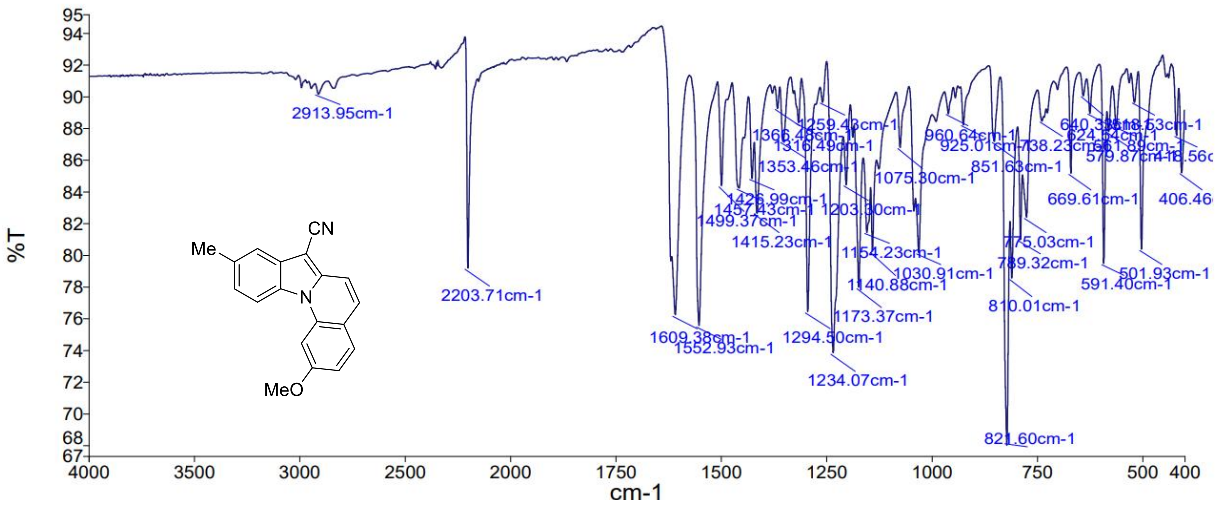

^{1}H NMR spectrum (400 MHz, $CDCl_3$) of compound **5at**

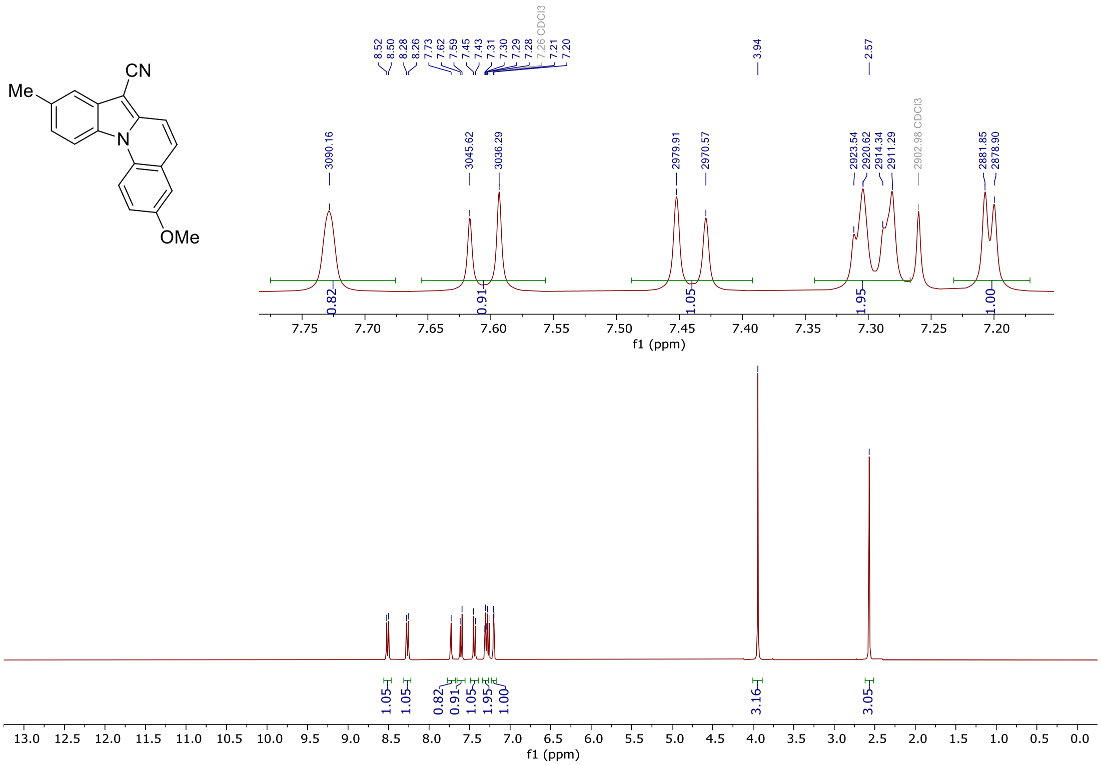

$^{13}C\{^{1}H\}$ NMR spectrum (100 MHz, $CDCl_3$) of compound **5at**

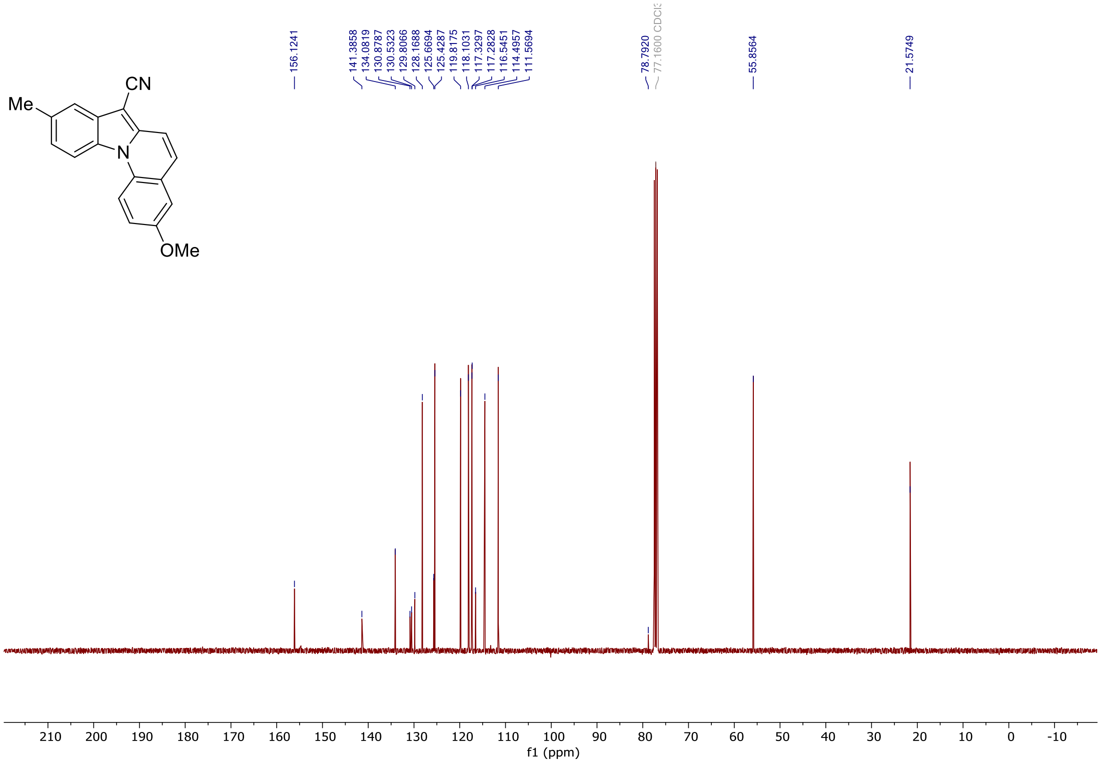

IR spectrum (neat) of compound **5at**

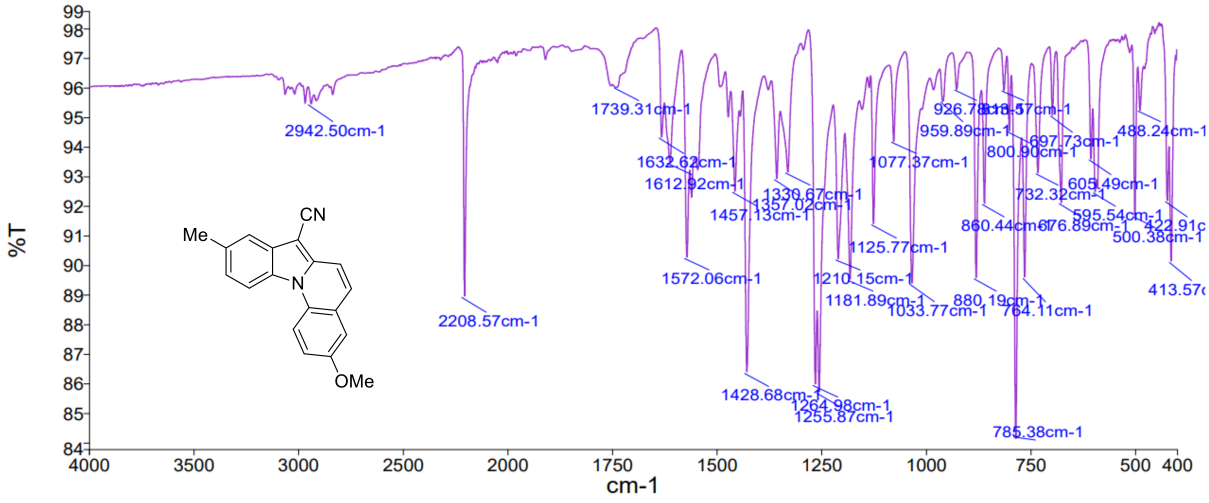

^{1}H NMR spectrum (400 MHz, $CDCl_3$) of compound **5au**

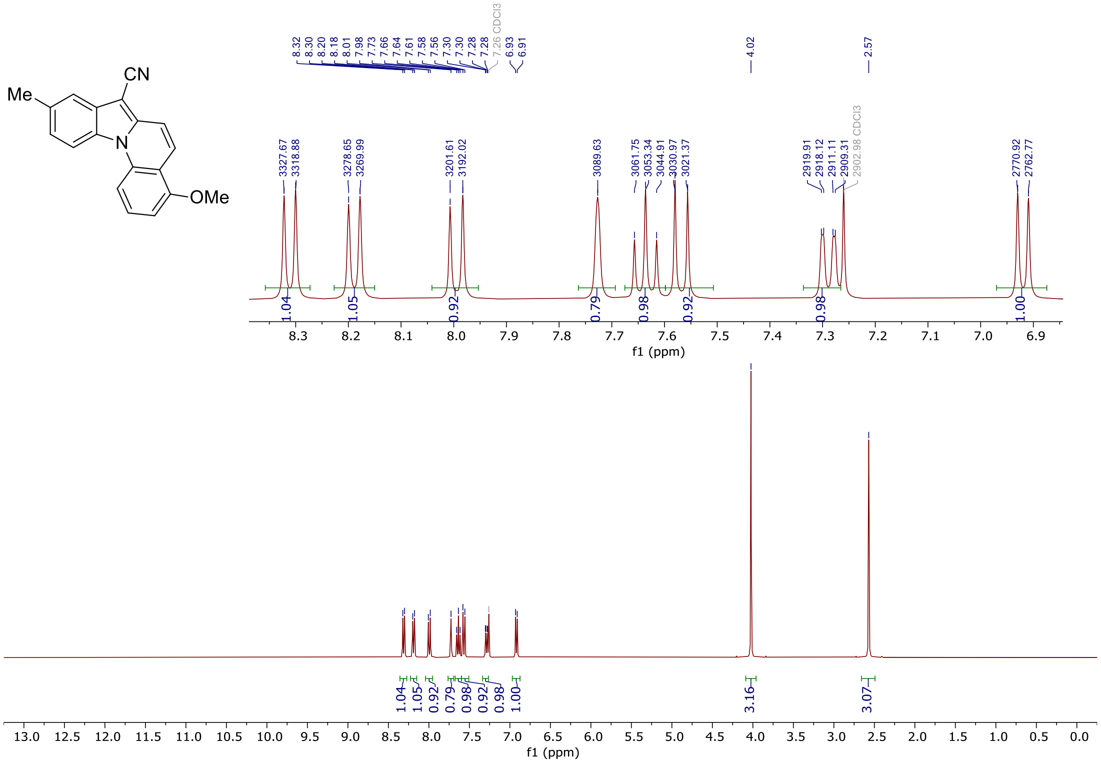

$^{13}C\{^{1}H\}$ NMR spectrum (100 MHz, $CDCl_3$) of compound **5au**

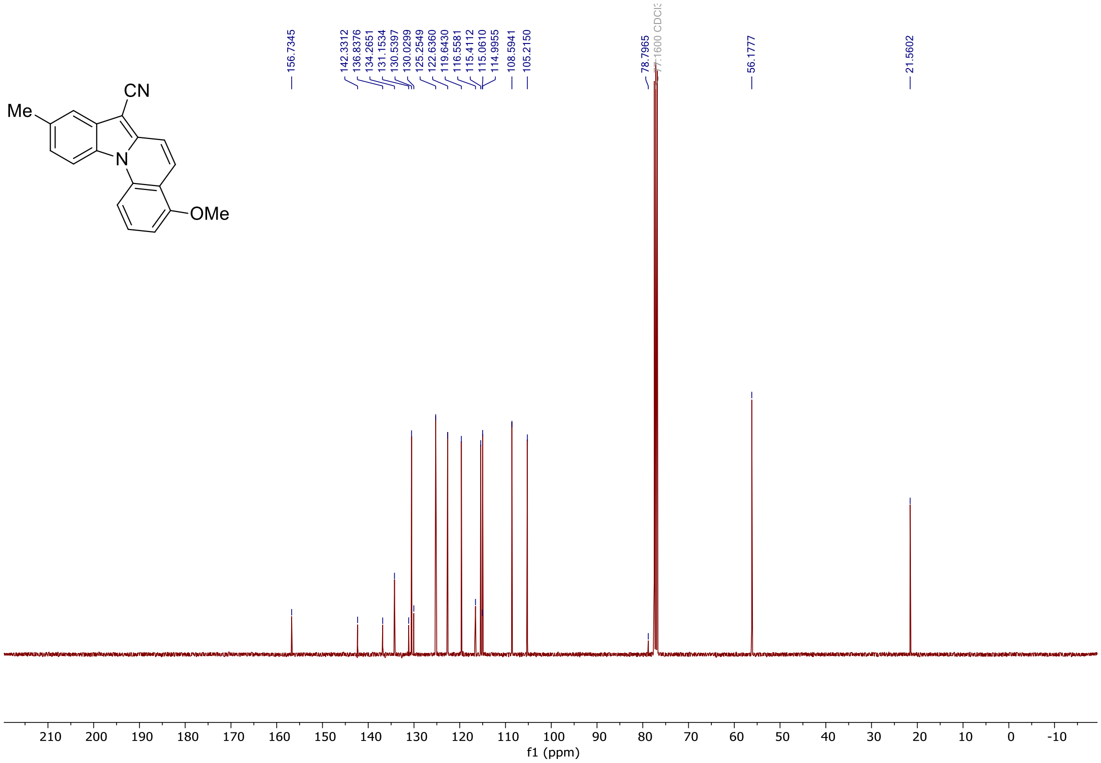

IR spectrum (neat) of compound **5au**

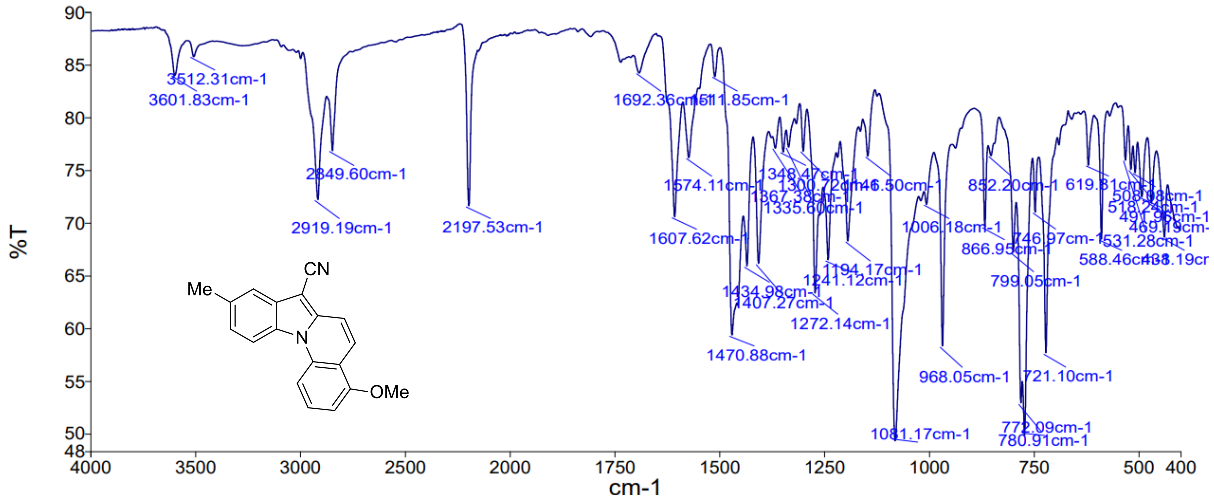

^{1}H NMR spectrum (400 MHz, $CDCl_3$) of compound **5bb**

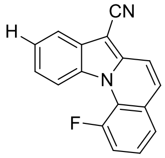

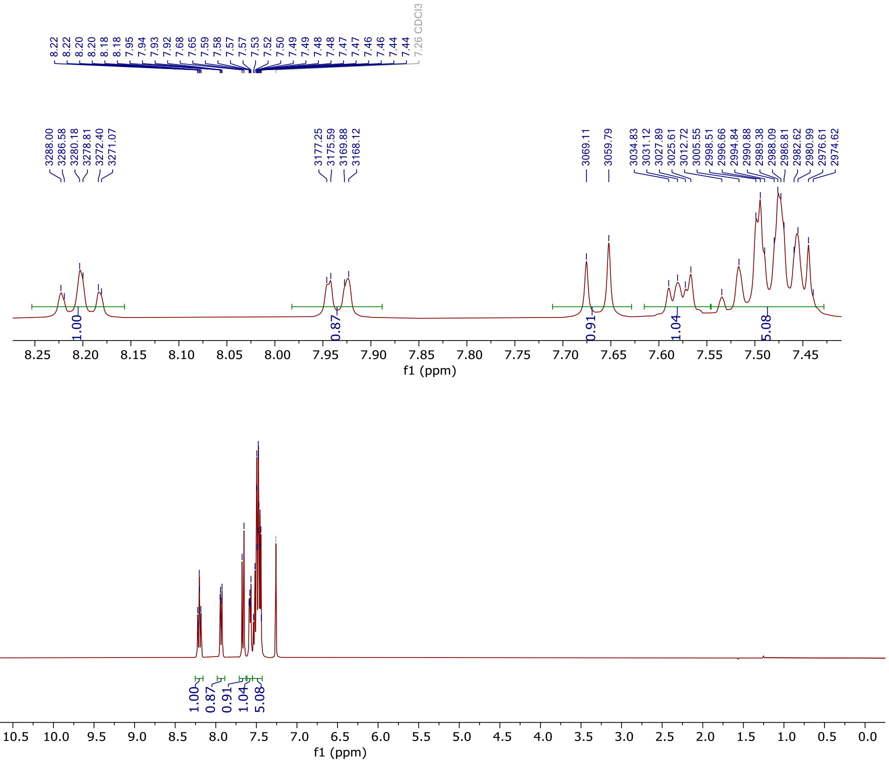

^{13}C{^{1}H} NMR spectrum (100 MHz, $CDCl_3$) of compound **5bb**

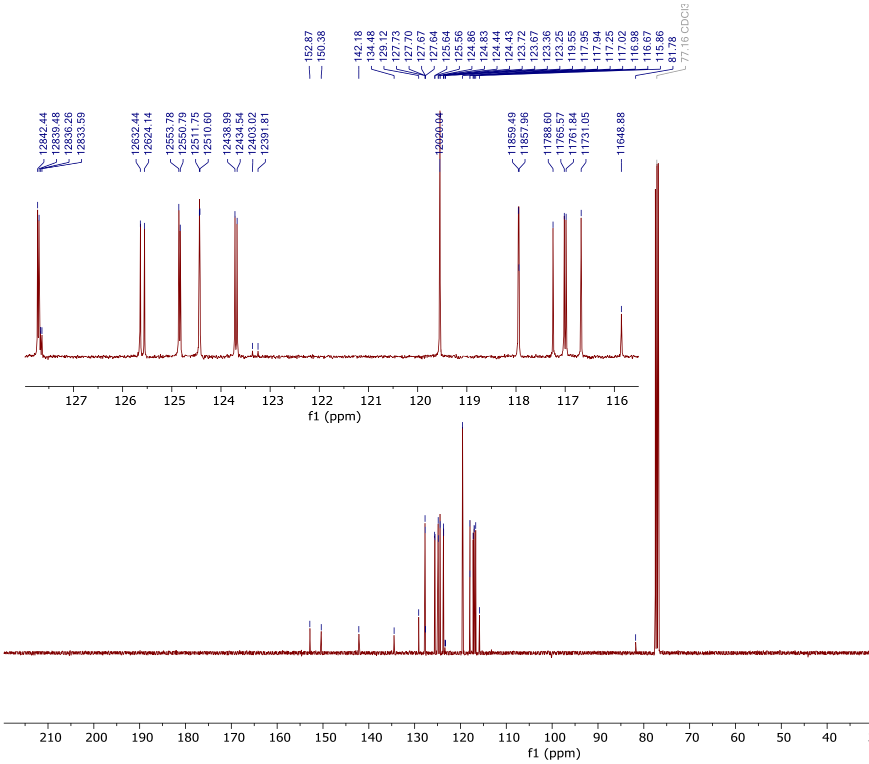

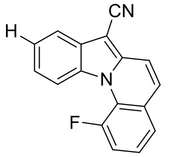

^{19}F NMR spectrum (470 MHz, $CDCl_3$) of compound **5bb**

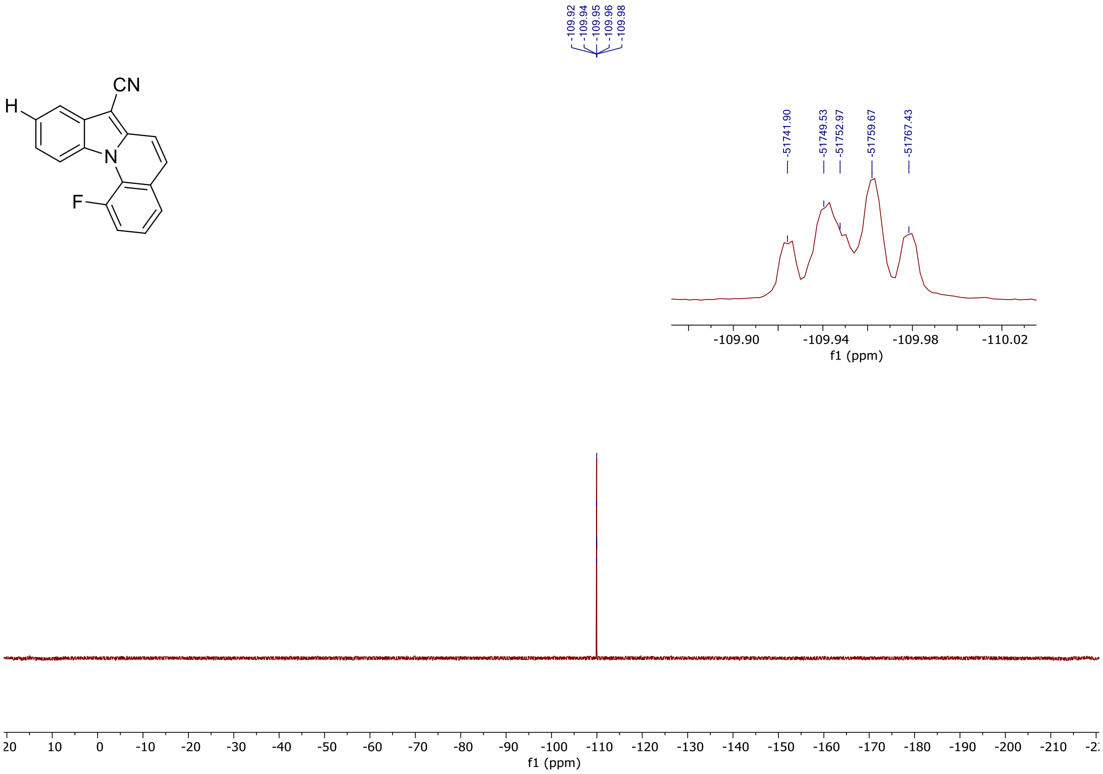

^{19}F NMR spectrum (470 MHz, $CDCl_3$) of compound **5bb** with an internal standard (fluorobenzene)

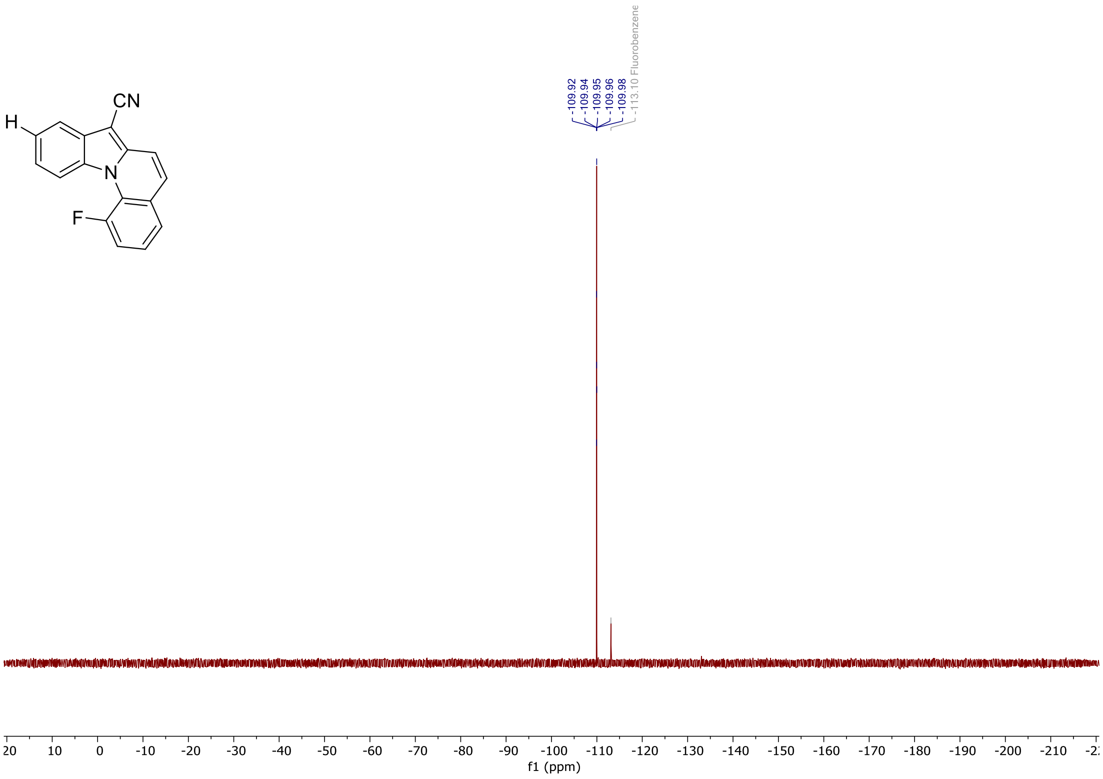

IR spectrum (neat) of compound **5bb**

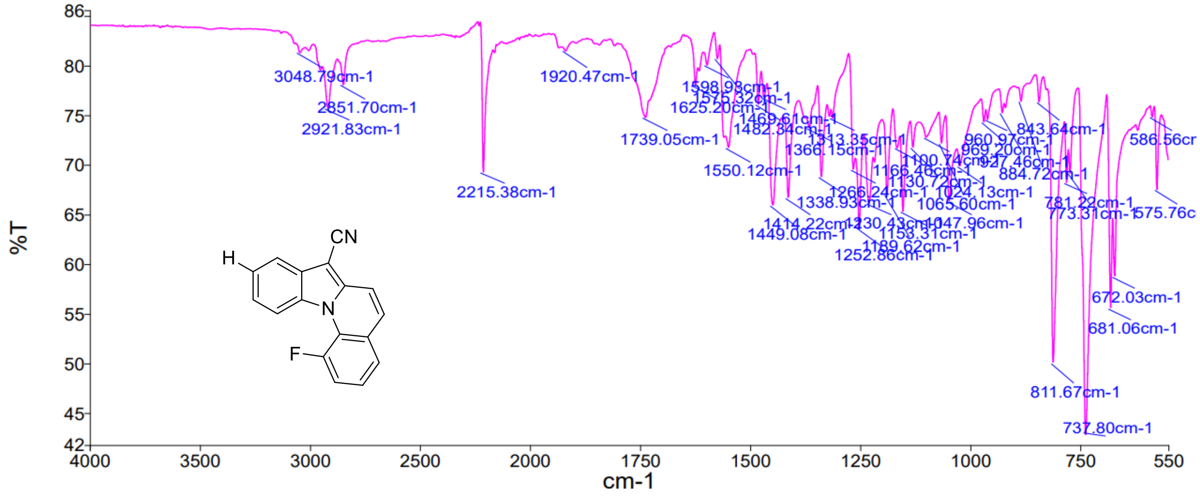

^{1}H NMR spectrum (400 MHz, $CDCl_3$) of compound **5bc**

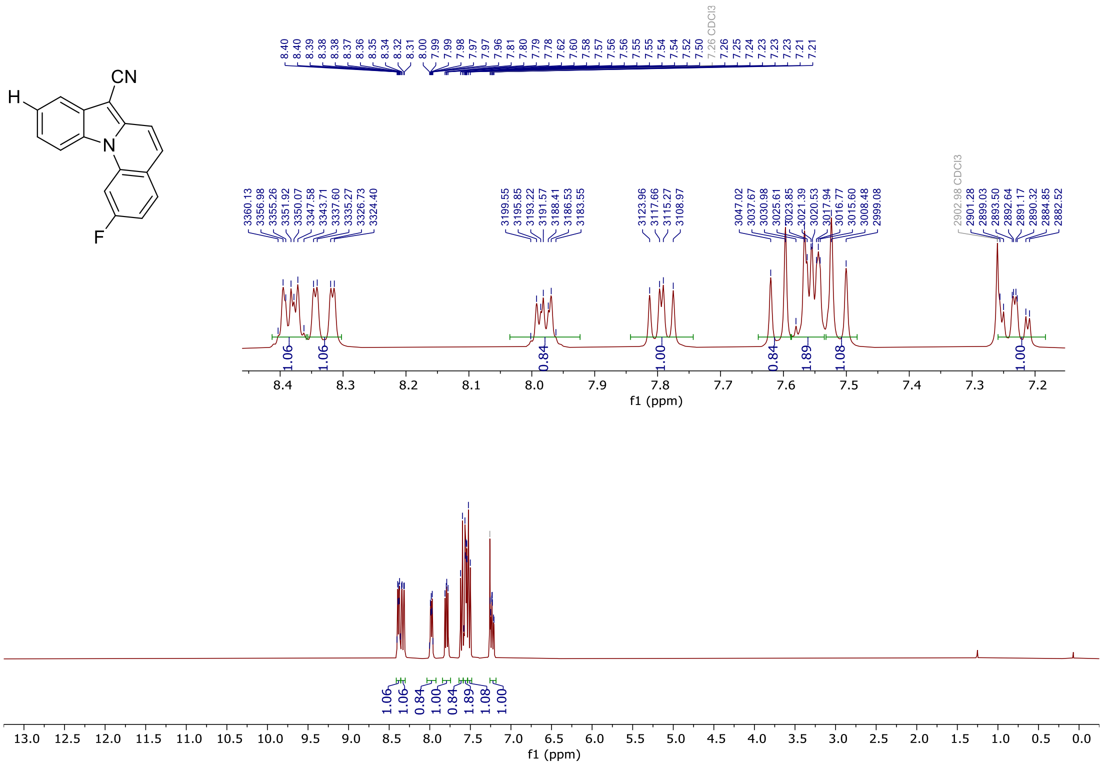

^{13}C{^{1}H} NMR spectrum (100 MHz, $CDCl_3$) of compound **5bc**

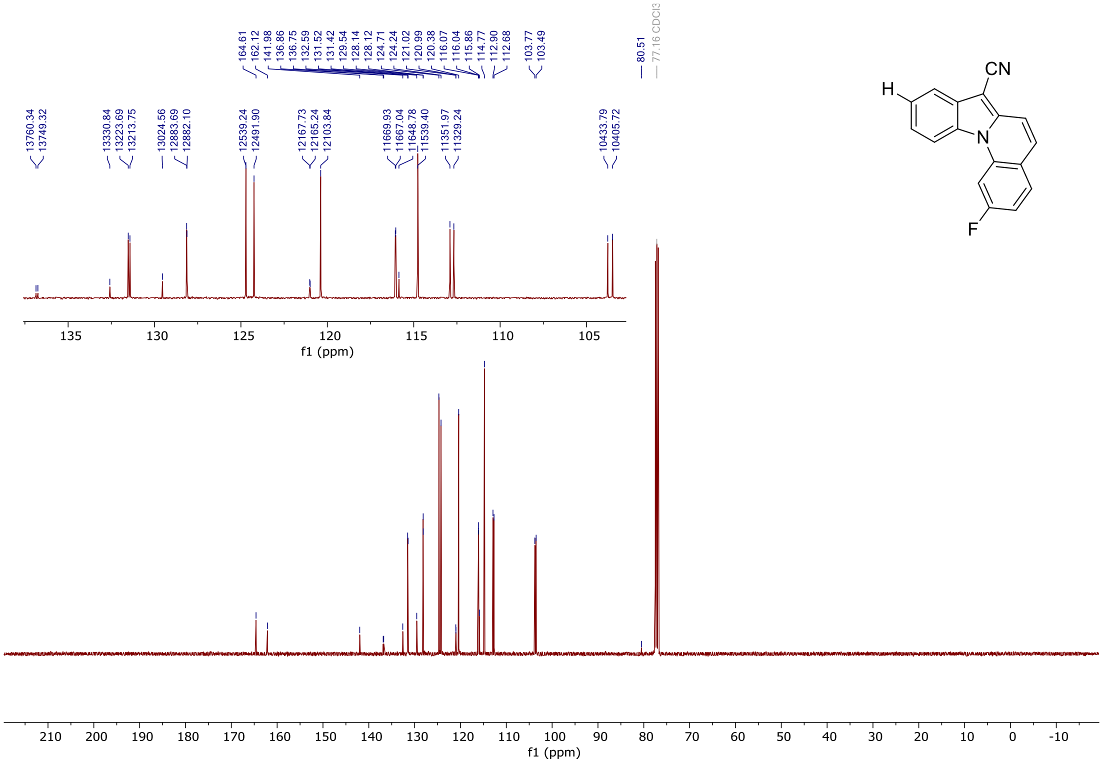

^{19}F NMR spectrum (470 MHz, $CDCl_3$) of compound **5bc**

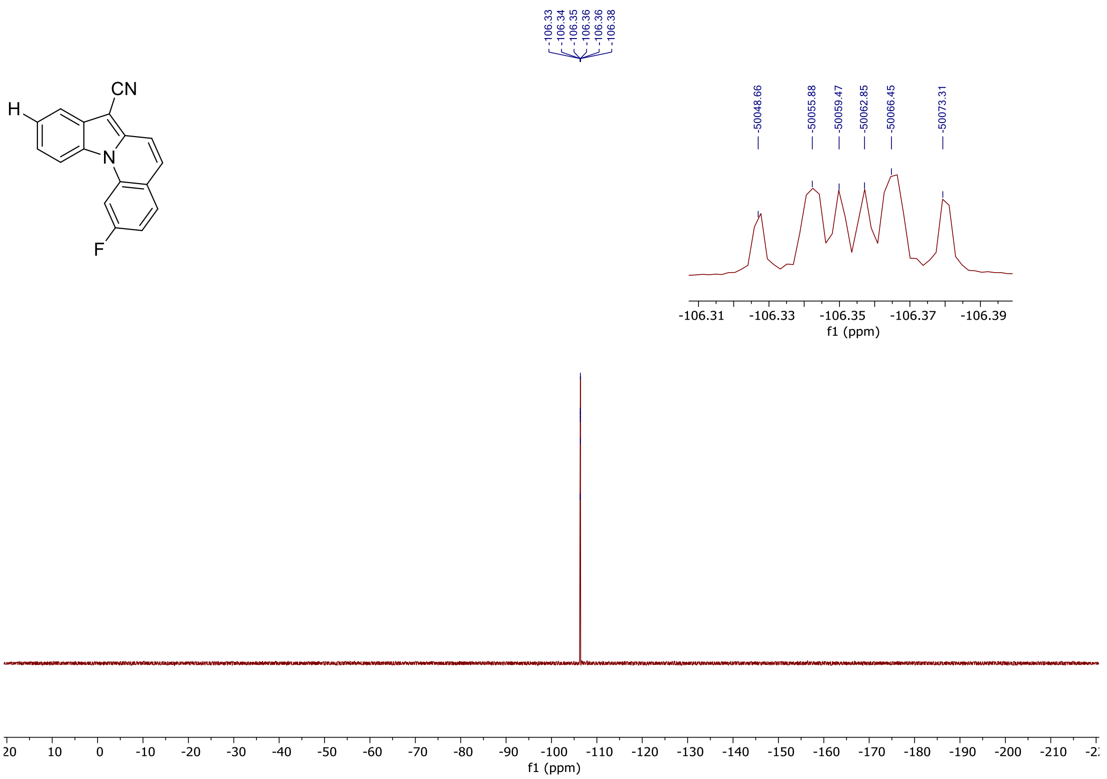

^{19}F NMR spectrum (500 MHz, $CDCl_3$) of compound **5bc** with an internal standard (fluorobenzene)

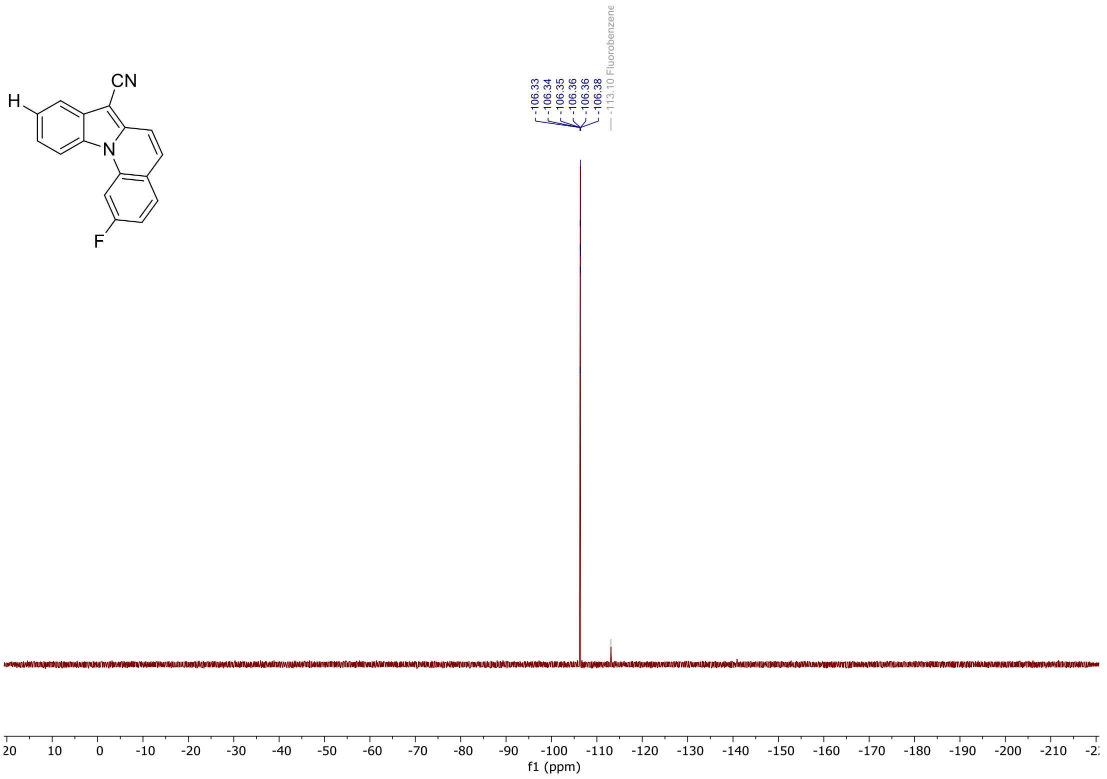

IR spectrum (neat) of compound **5bc**

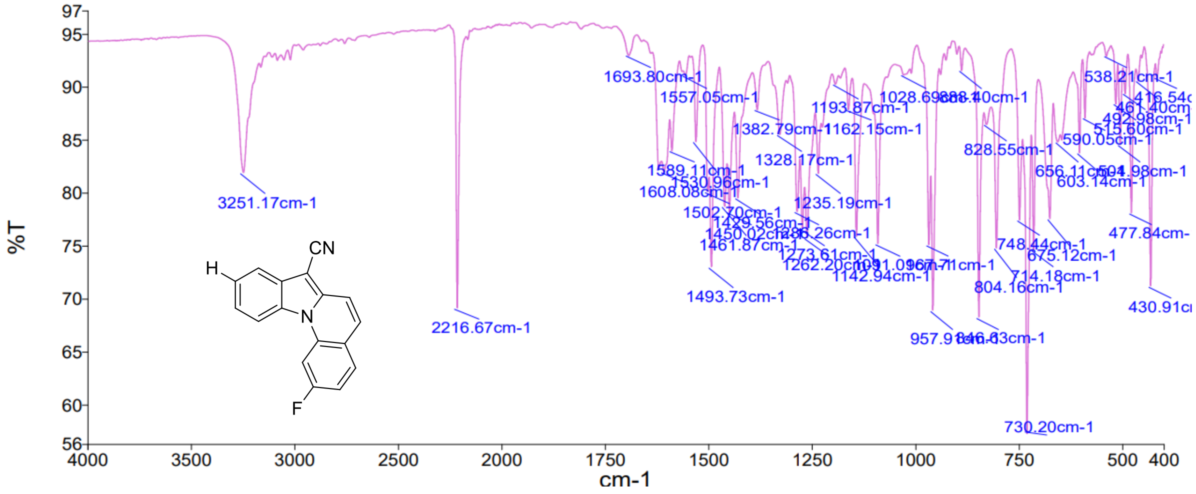

^{1}H NMR spectrum (400 MHz, $CDCl_3$) of compound **5bd**

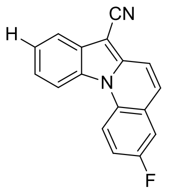

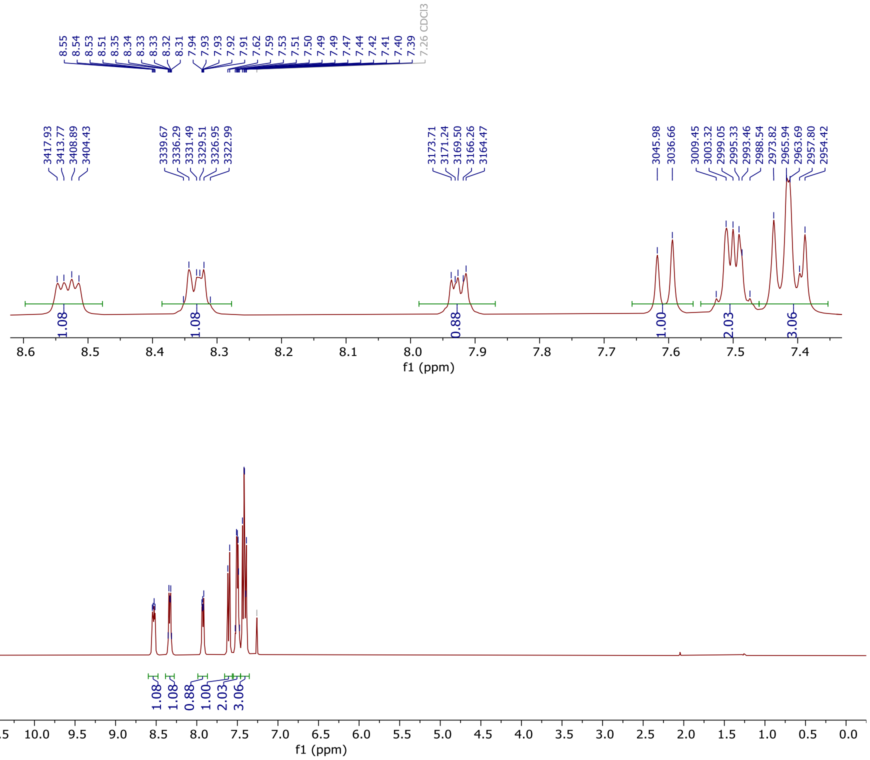

^{13}C{^{1}H} NMR spectrum (100 MHz, $CDCl_3$) of compound **5bd**

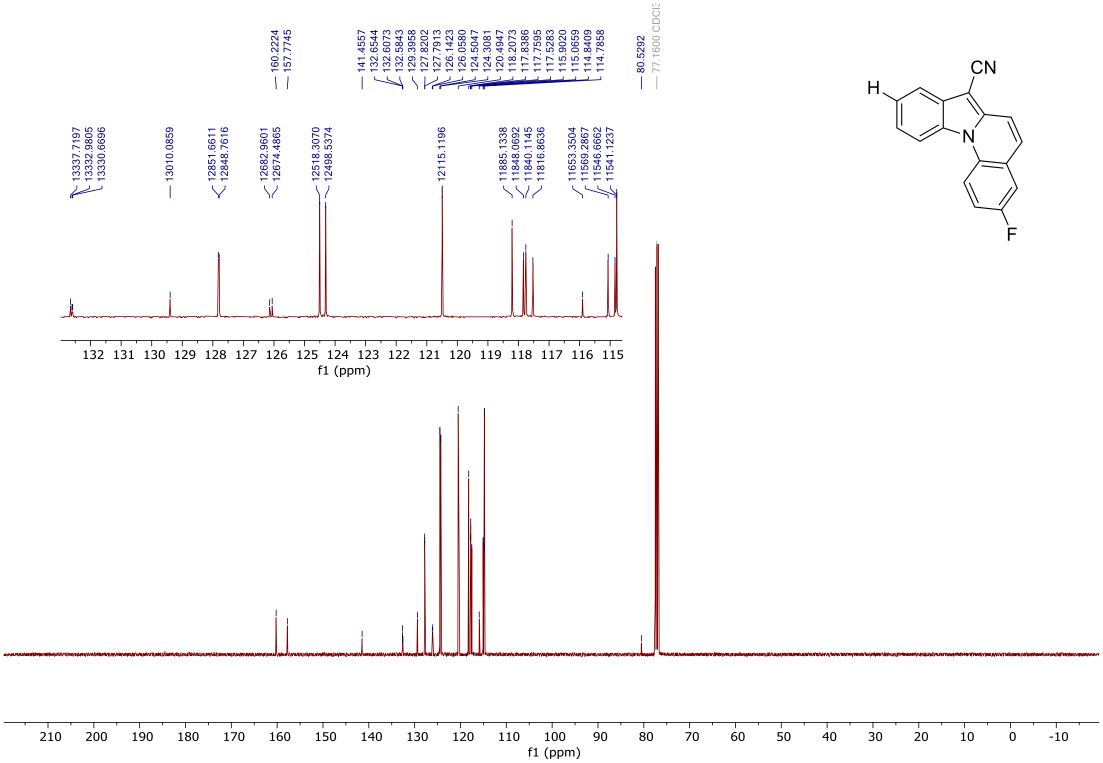

^{19}F NMR spectrum (500 MHz, $CDCl_3$) of compound **5bd**

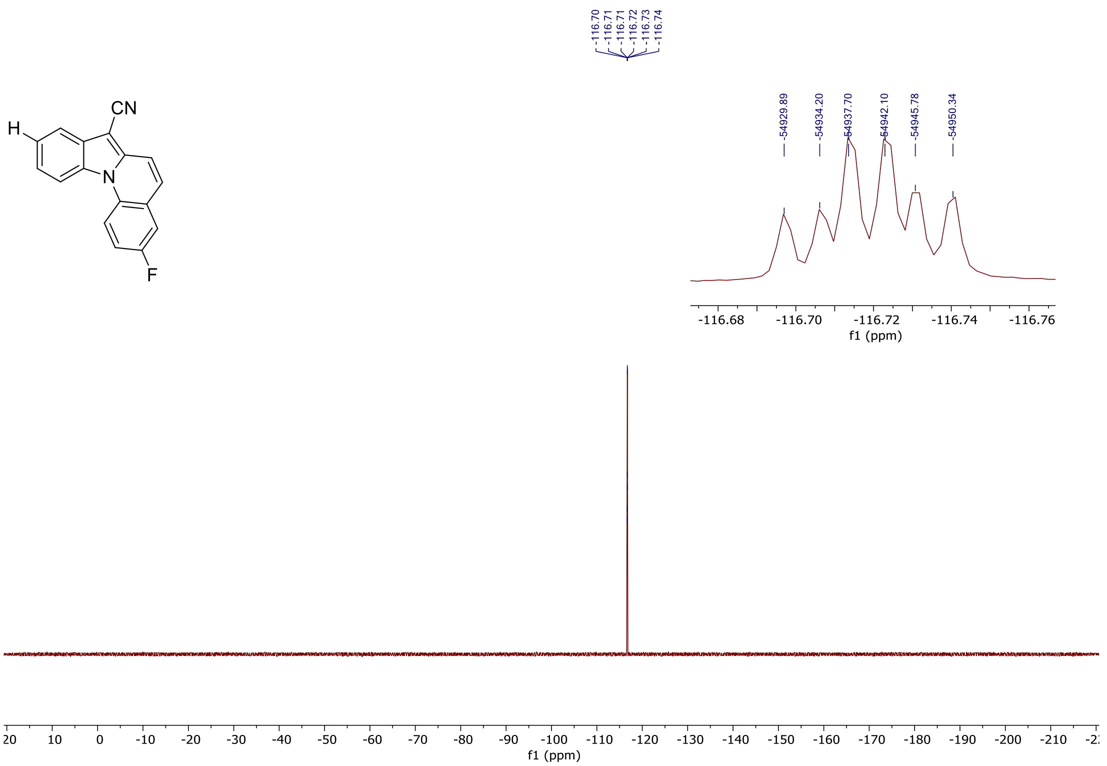

^{19}F NMR spectrum (500 MHz, $CDCl_3$) of compound **5bd** with an internal standard (fluorobenzene)

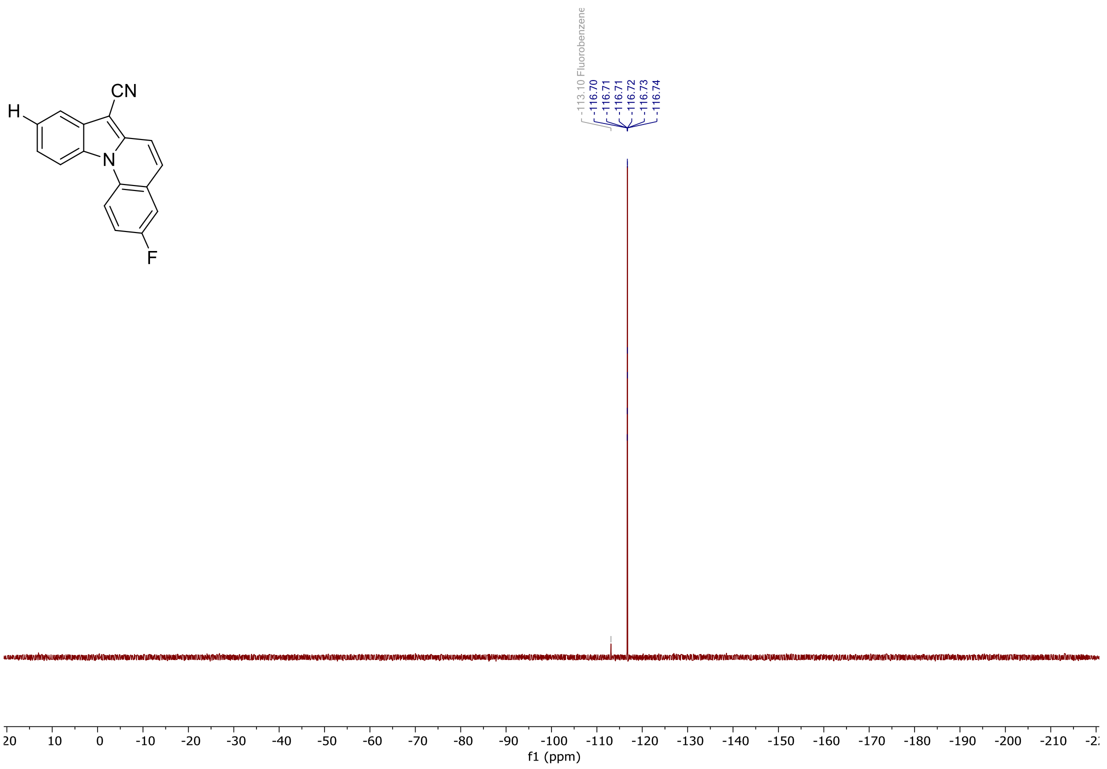

IR spectrum (neat) of compound **5bd**

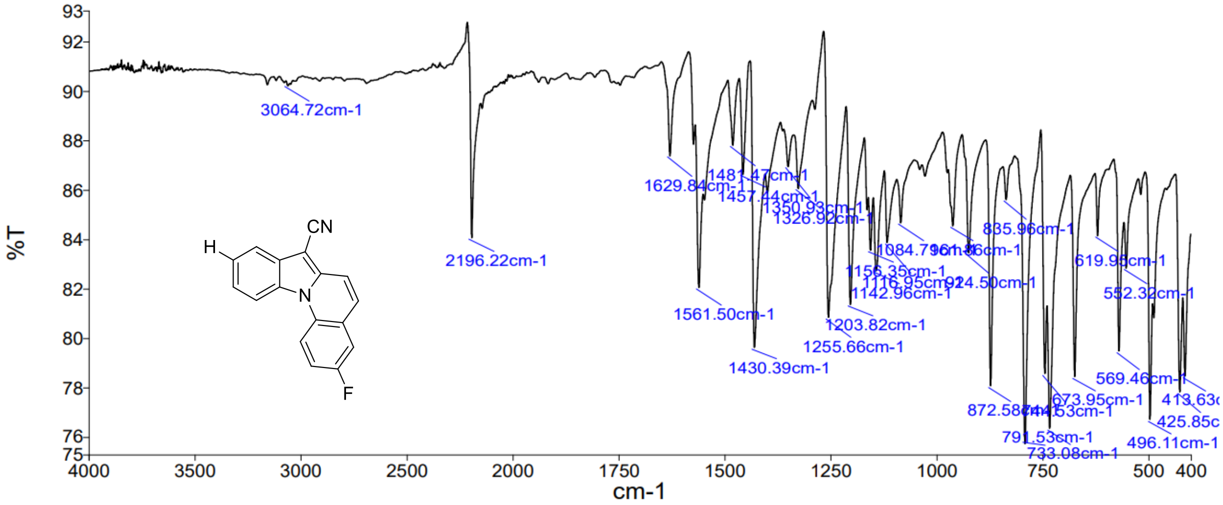

^{1}H NMR spectrum (400 MHz, $CDCl_3$) of compound **5be**

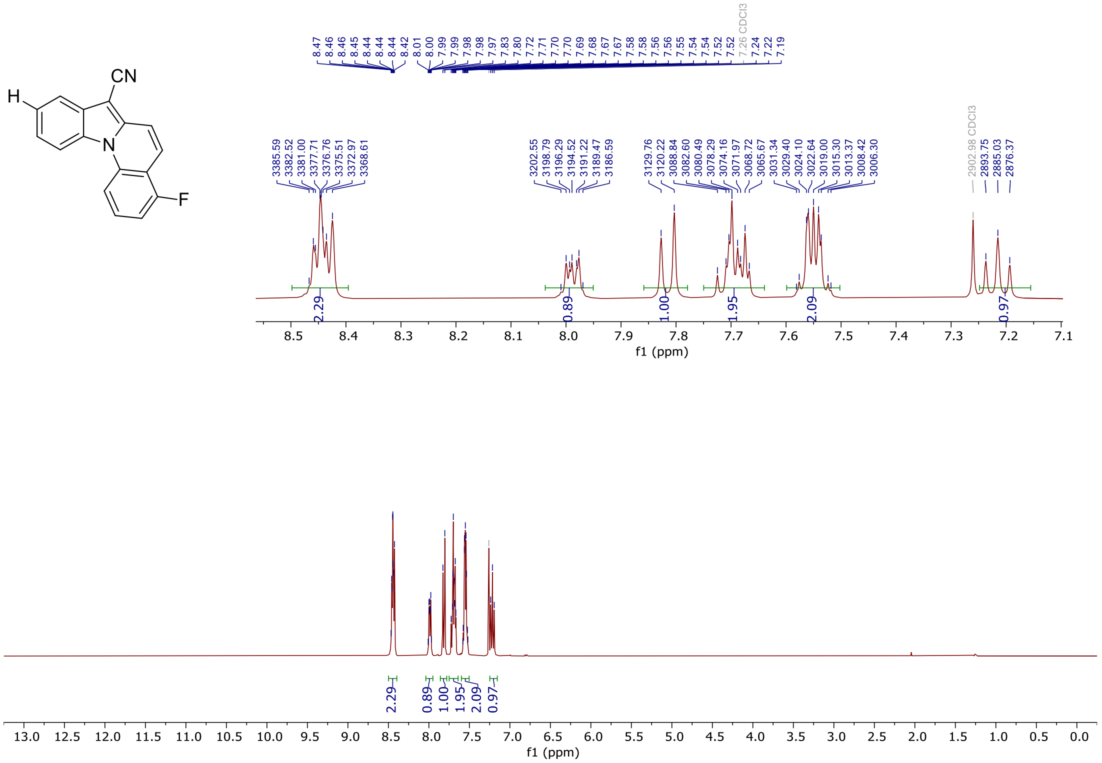

$^{13}C\{^{1}H\}$ NMR spectrum (100 MHz, $CDCl_3$) of compound **5be**

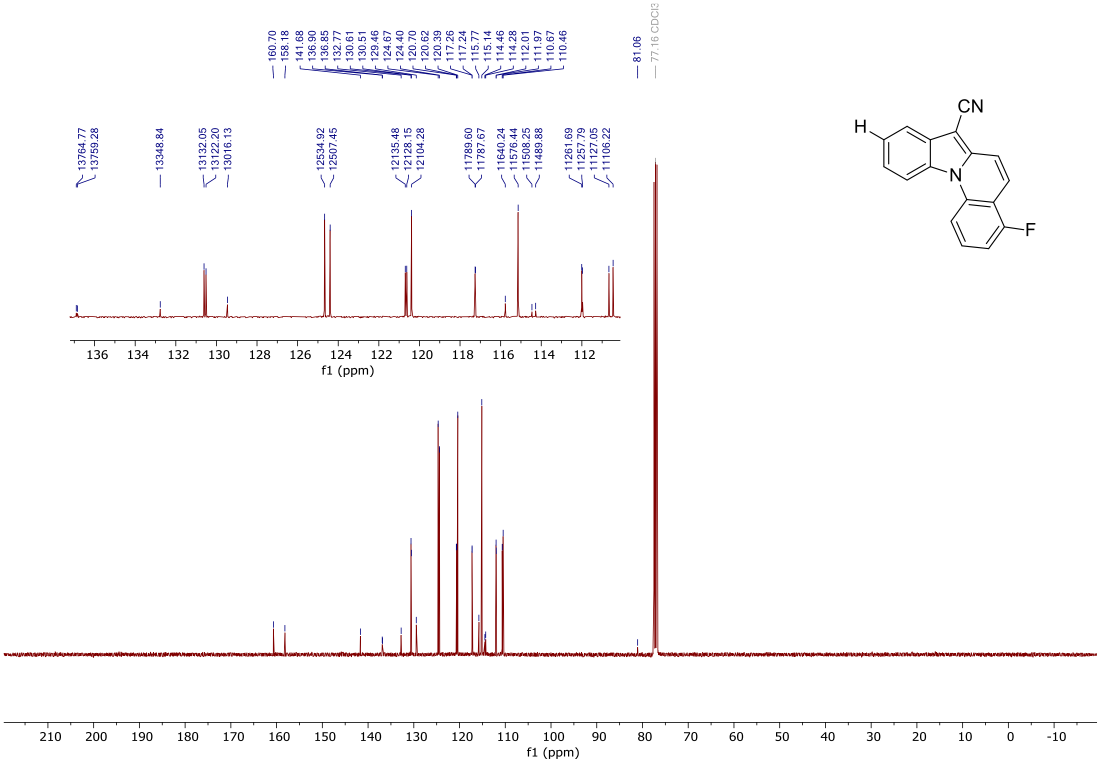

^{19}F NMR spectrum (500 MHz, CDCl$_3$) of compound **5be**

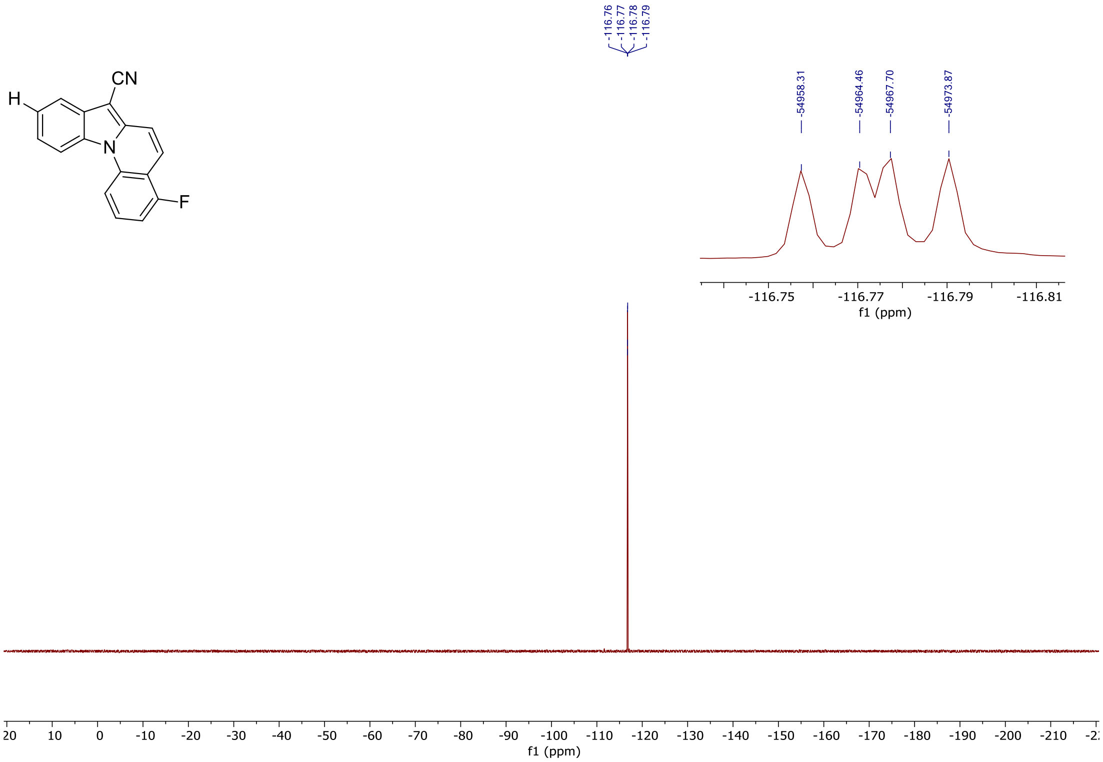

^{19}F NMR spectrum (500 MHz, CDCl$_3$) of compound **5be** with an internal standard (fluorobenzene)

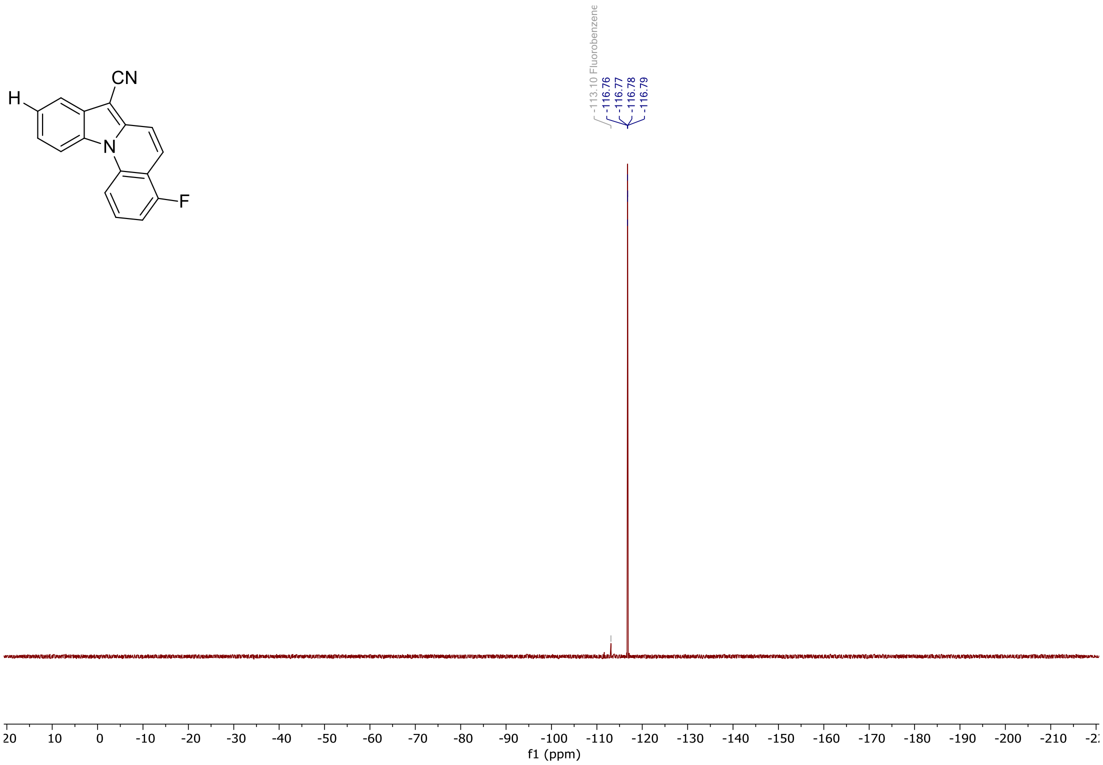

IR spectrum (neat) of compound **5be**

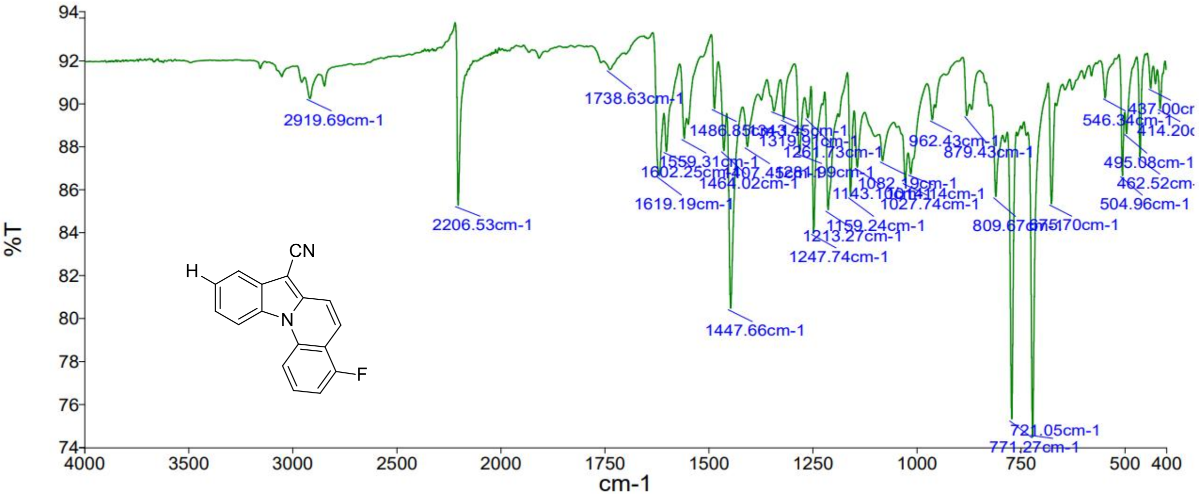

^{1}H NMR spectrum (400 MHz, $CDCl_3$) of compound **5bf**

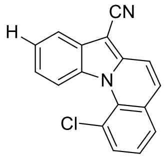

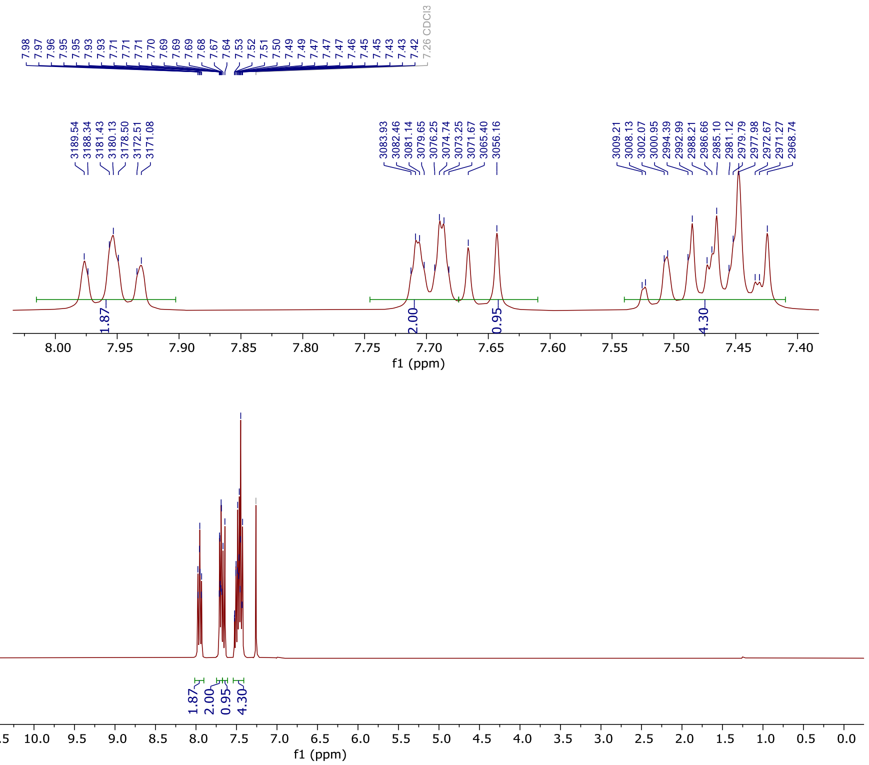

$^{13}C\{^{1}H\}$ NMR spectrum (100 MHz, $CDCl_3$) of compound **5bf**

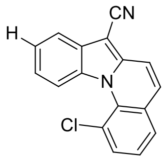

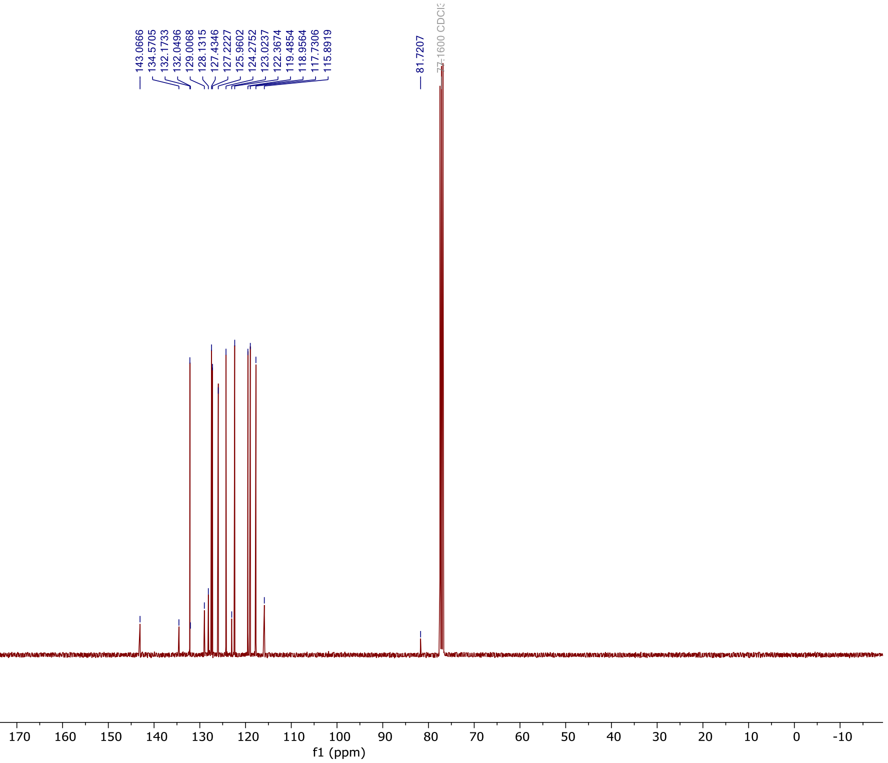

IR spectrum (neat) of compound **5bf**

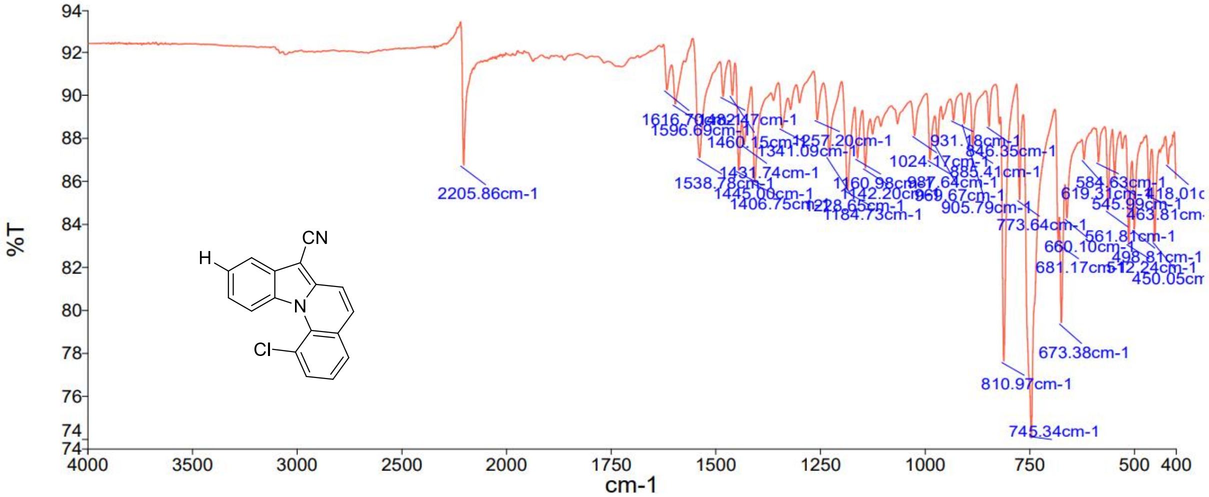

^{1}H NMR spectrum (400 MHz, $CDCl_3$) of compound **5bg**

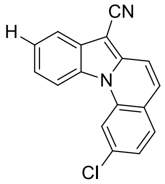

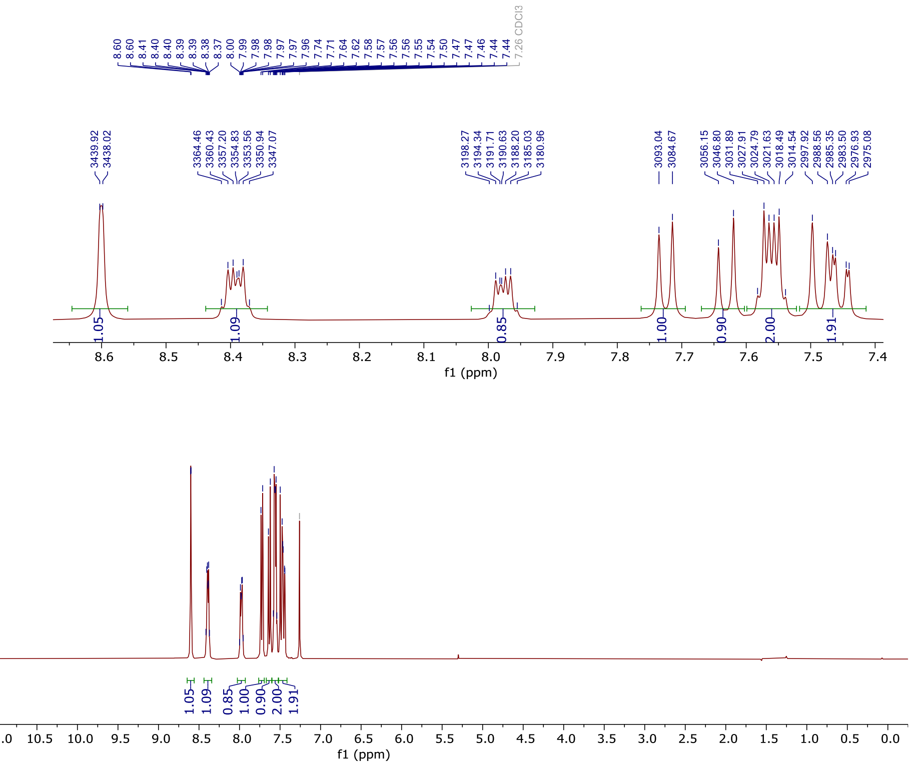

$^{13}C\{^{1}H\}$ NMR spectrum (100 MHz, $CDCl_3$) of compound **5bg**

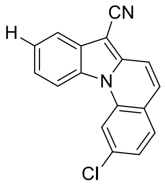

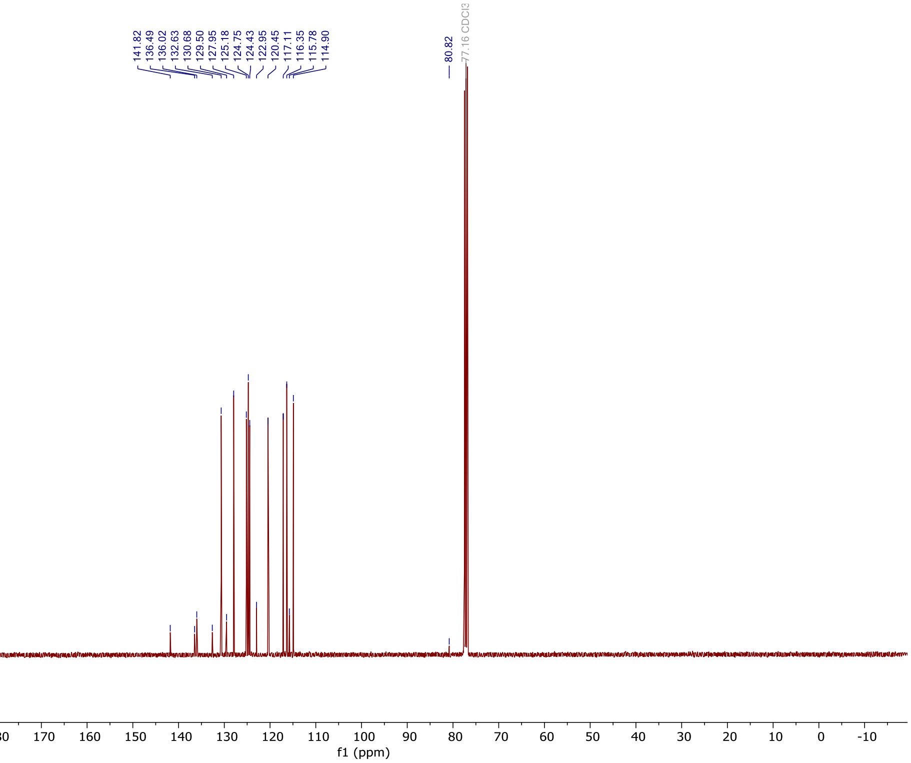

IR spectrum (neat) of compound **5bg**

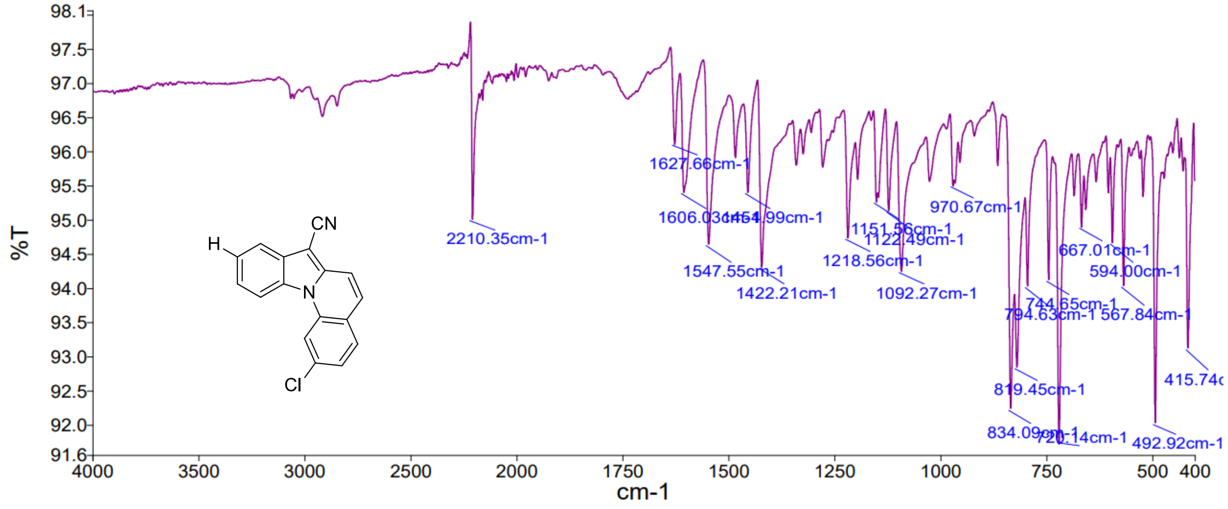

^{1}H NMR spectrum (400 MHz, $CDCl_3$) of compound **5bh**

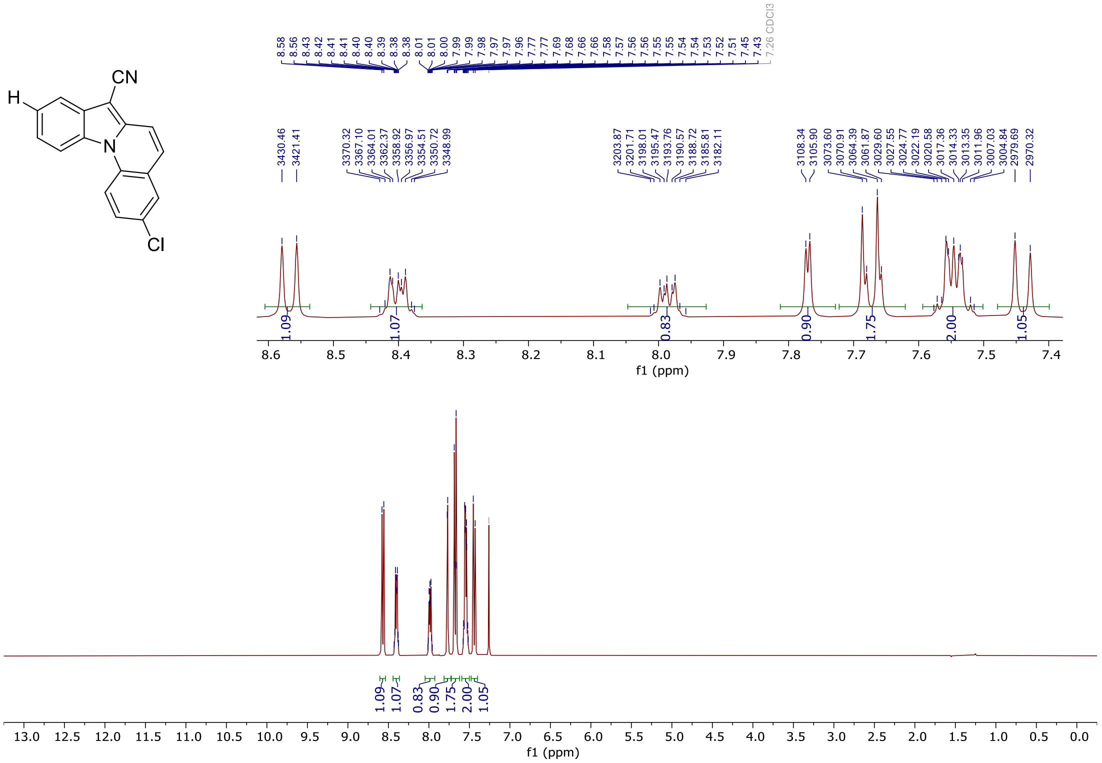

$^{13}C\{^{1}H\}$ NMR spectrum (100 MHz, $CDCl_3$) of compound **5bh**

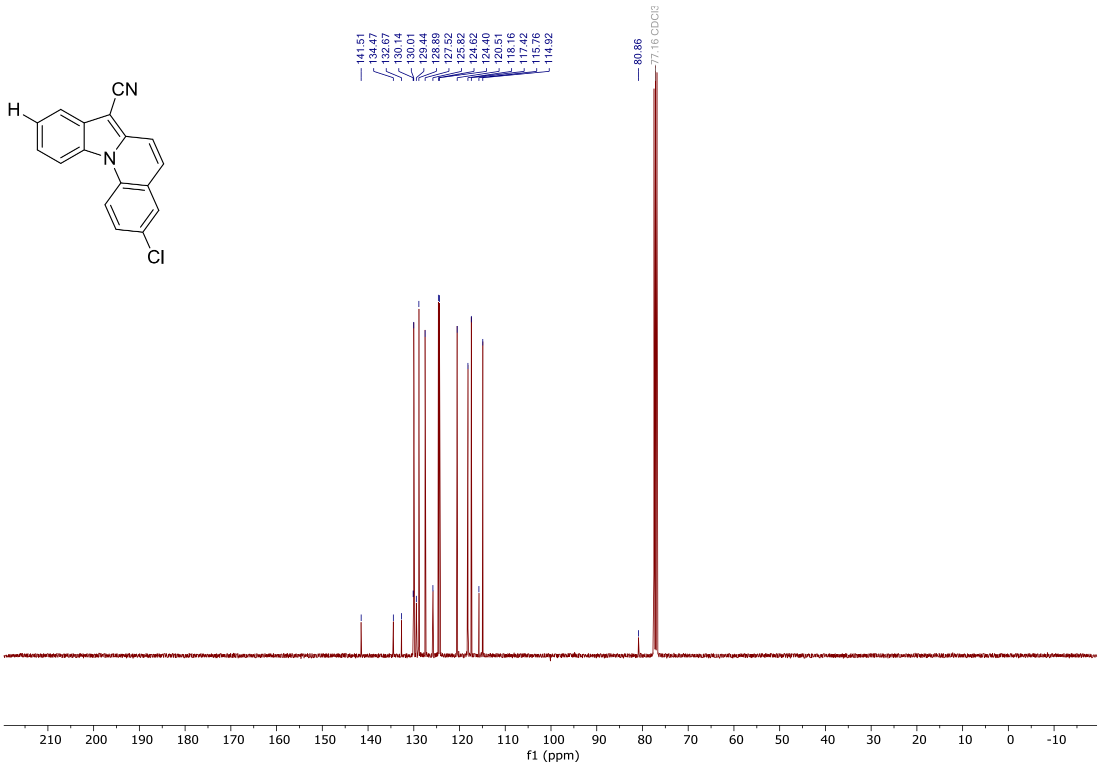

IR spectrum (neat) of compound **5bh**

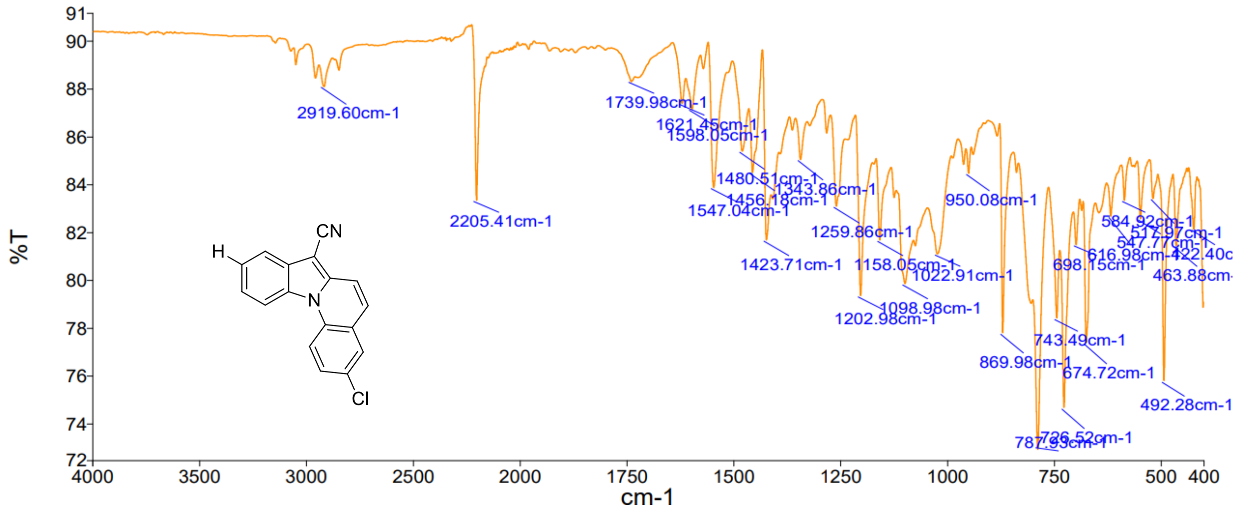

^{1}H NMR spectrum (400 MHz, $CDCl_3$) of compound **5bi**

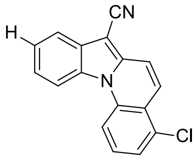

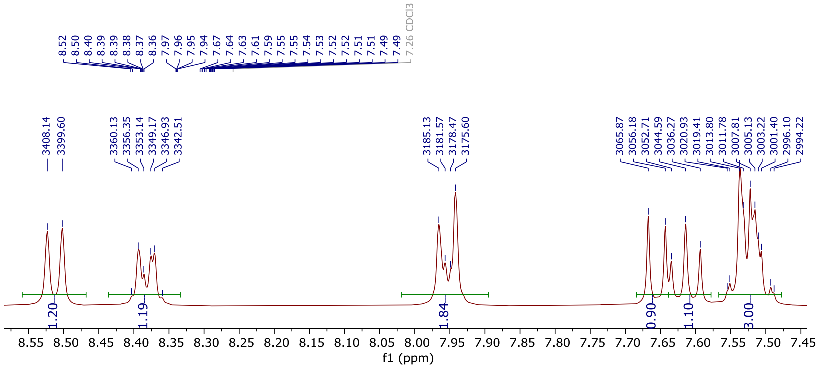

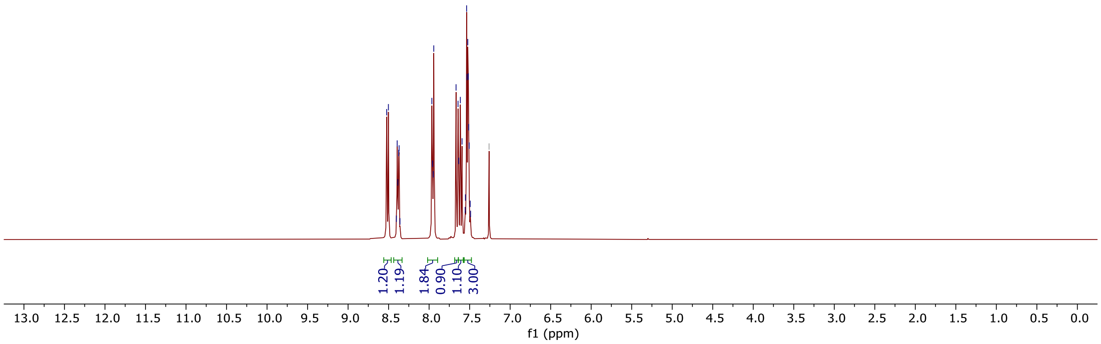

^{13}C{^{1}H} NMR spectrum (100 MHz, $CDCl_3$) of compound **5bi**

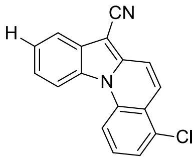

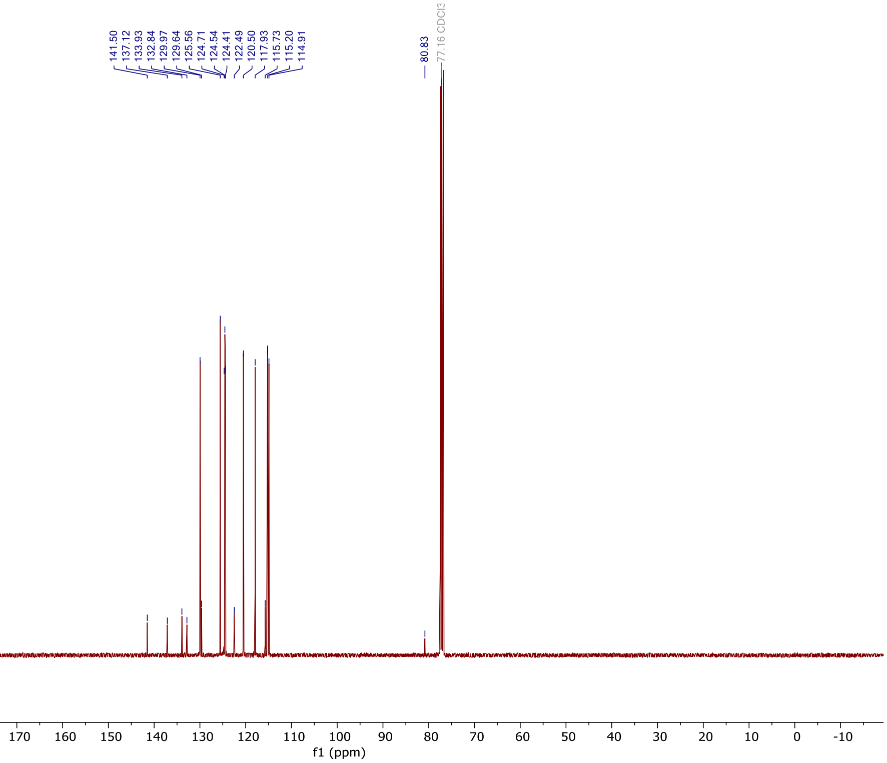

IR spectrum (neat) of compound **5bi**

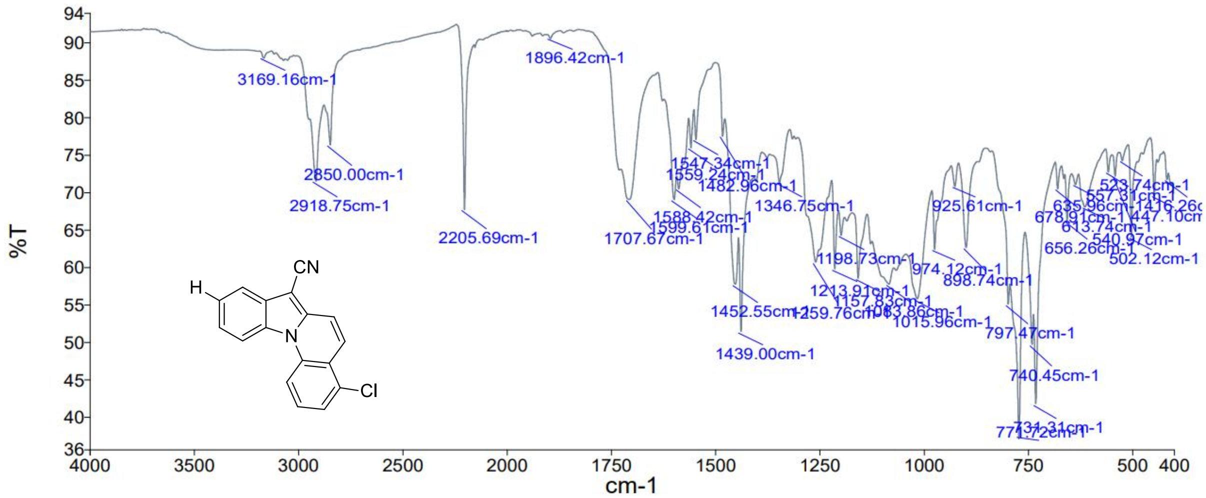

^{1}H NMR spectrum (400 MHz, $CDCl_3$) of compound **5bk**

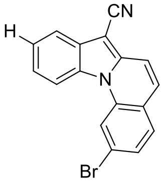

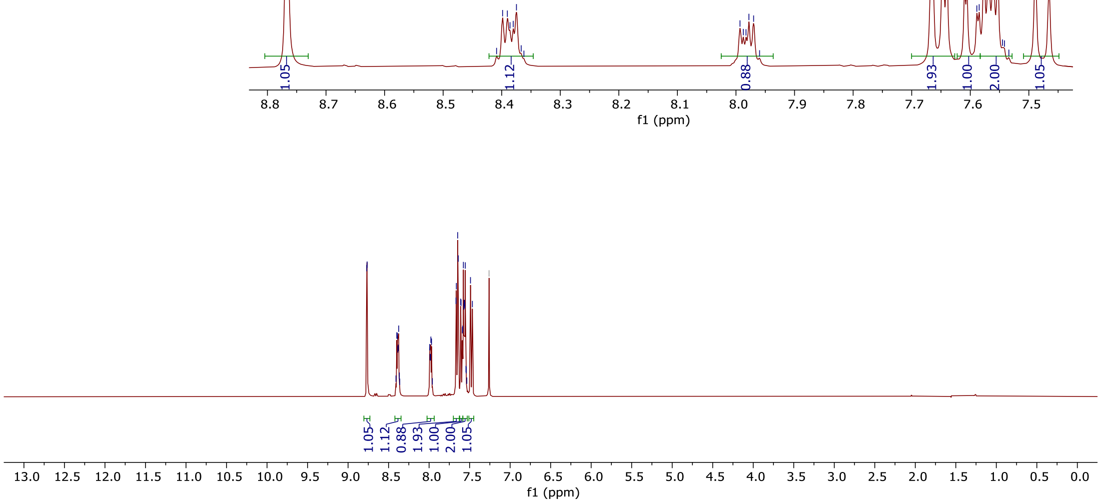

^{13}C{^{1}H} NMR spectrum (100 MHz, $CDCl_3$) of compound **5bk**

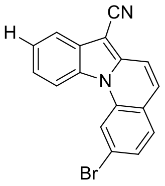

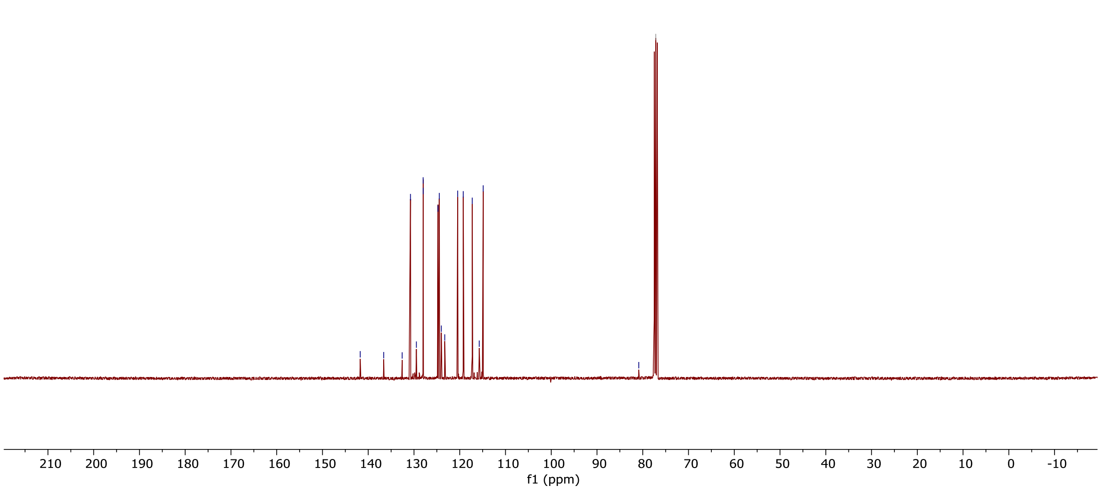

IR spectrum (neat) of compound **5bk**

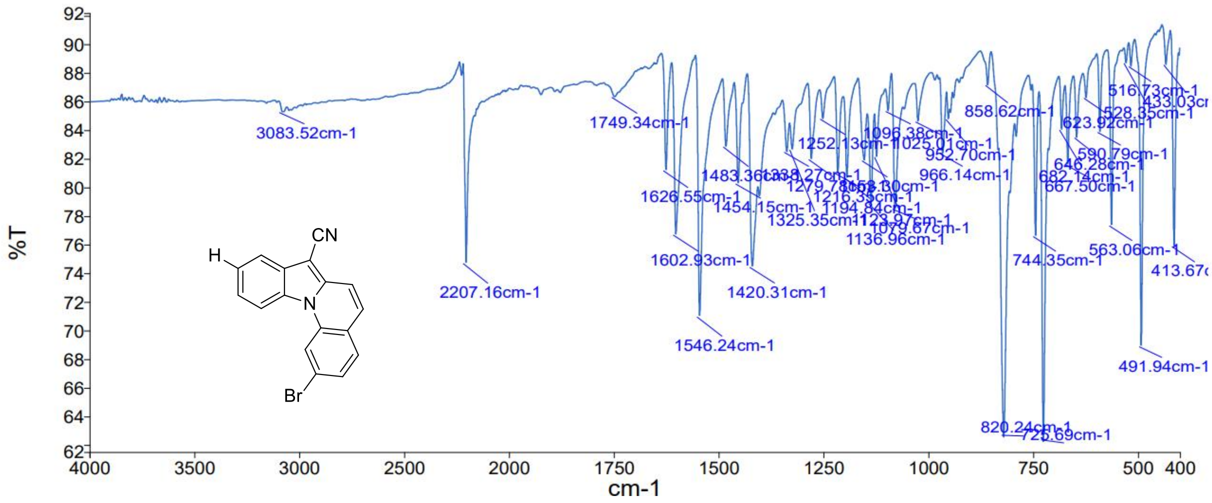

^{1}H NMR spectrum (400 MHz, $CDCl_3$) of compound **5bl**

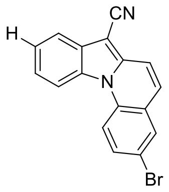

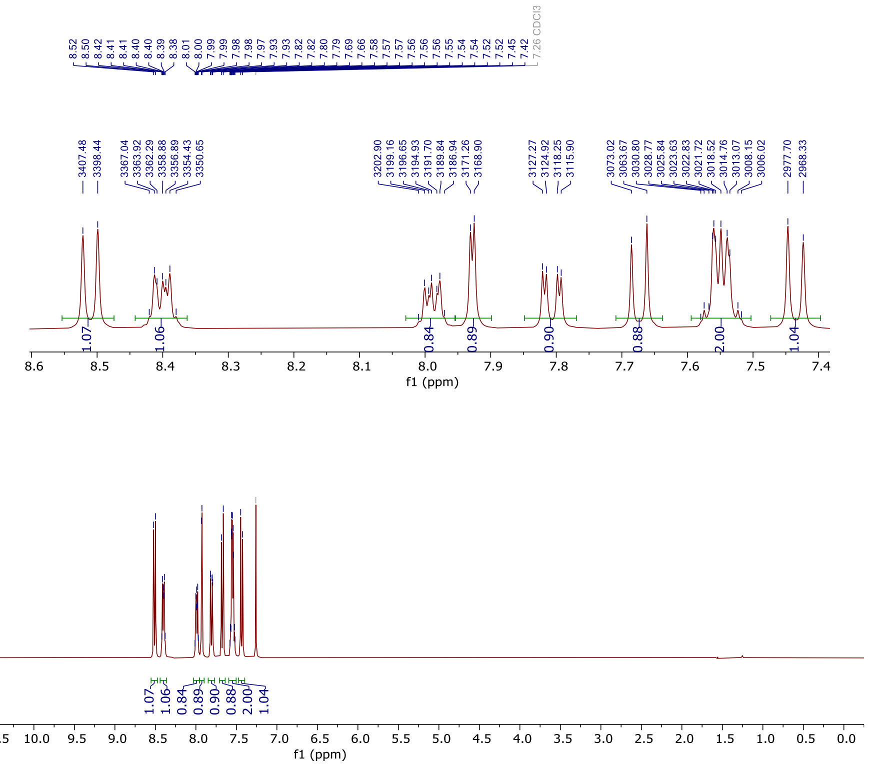

$^{13}C\{^{1}H\}$ NMR spectrum (100 MHz, $CDCl_3$) of compound **5bl**

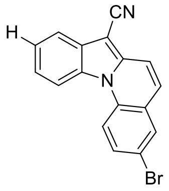

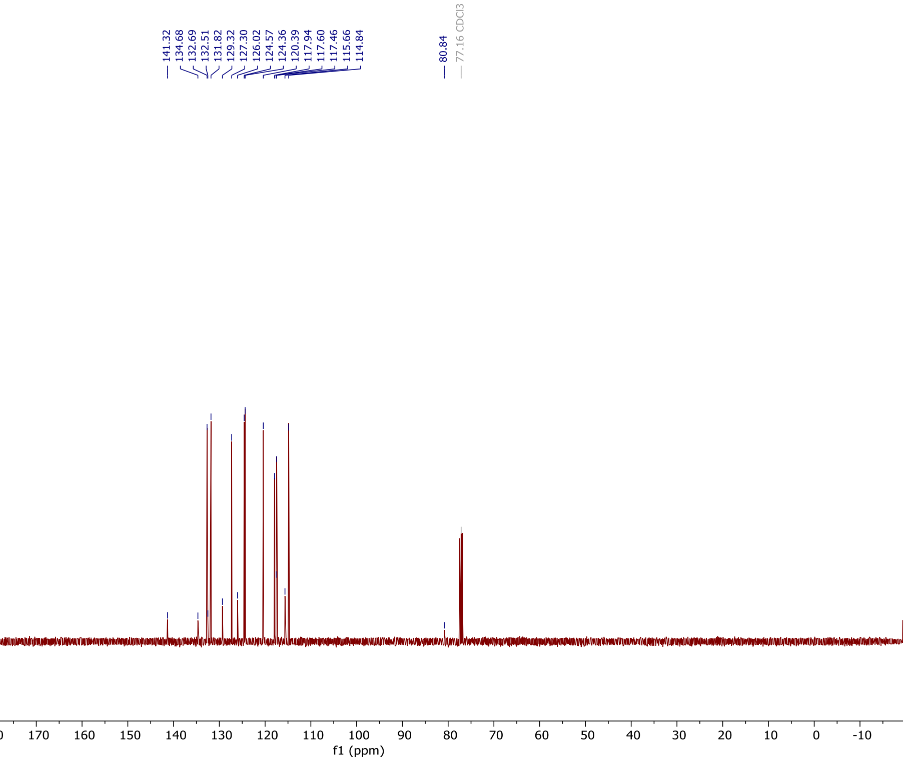

IR spectrum (neat) of compound **5bl**

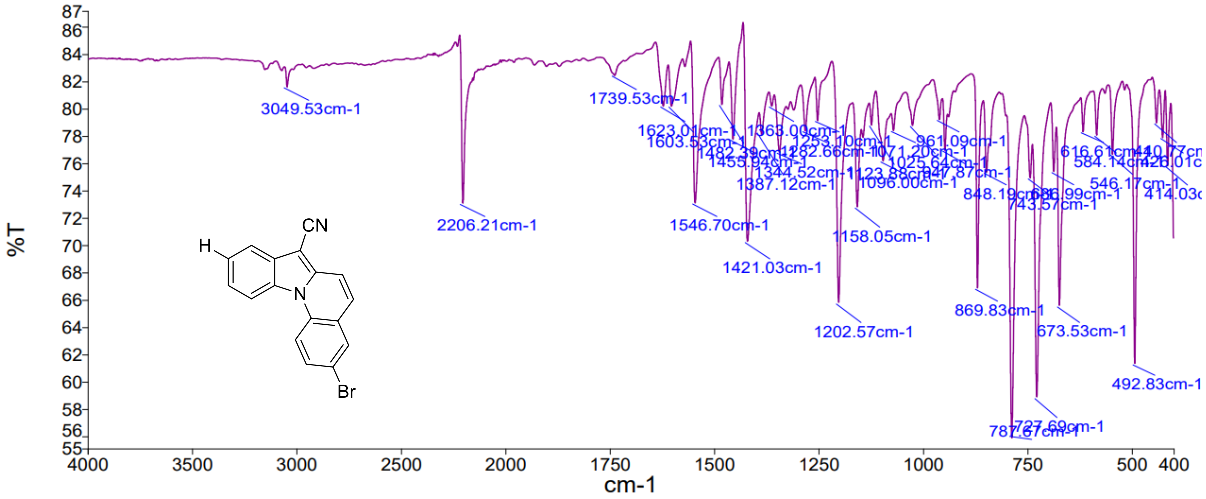

^{1}H NMR spectrum (400 MHz, $CDCl_3$) of compound **5bm**

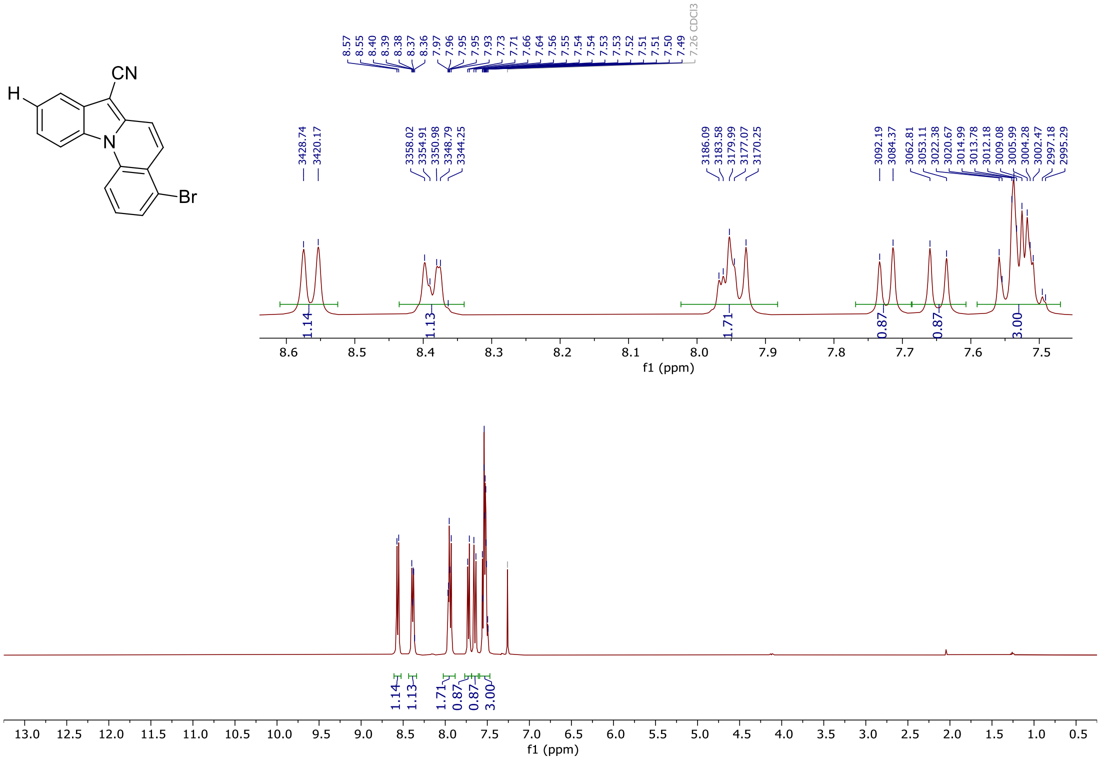

^{13}C{^{1}H} NMR spectrum (100 MHz, $CDCl_3$) of compound **5bm**

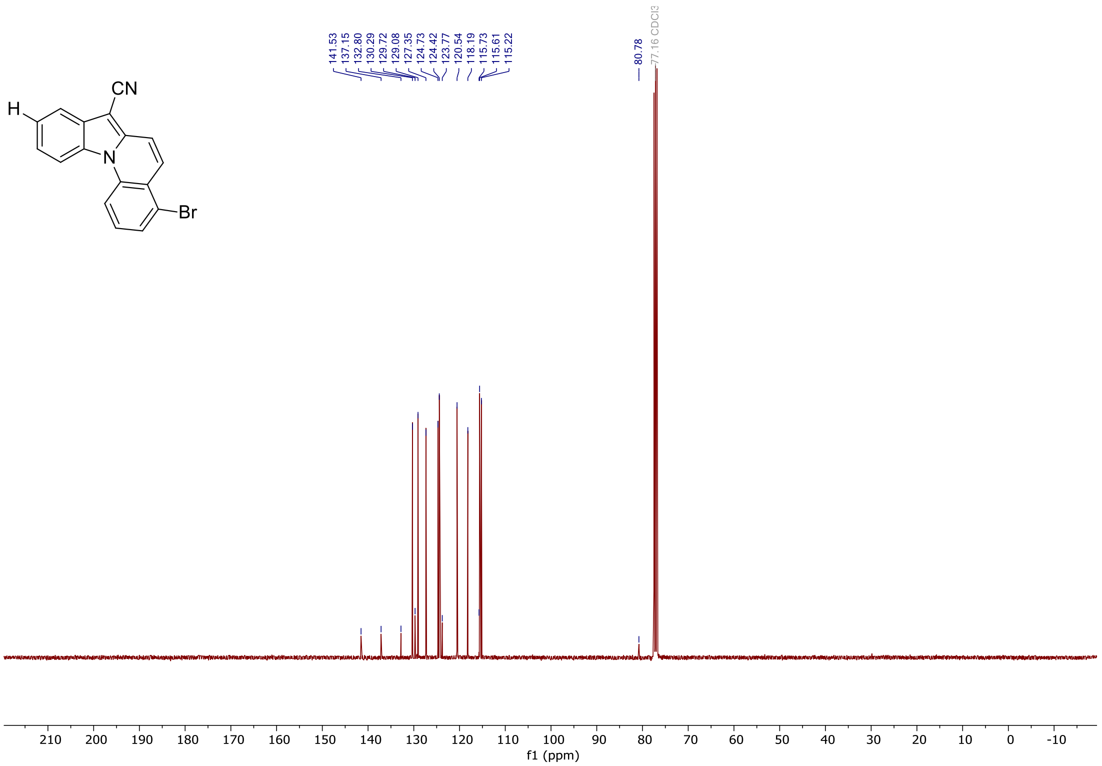

IR spectrum (neat) of compound **5bm**

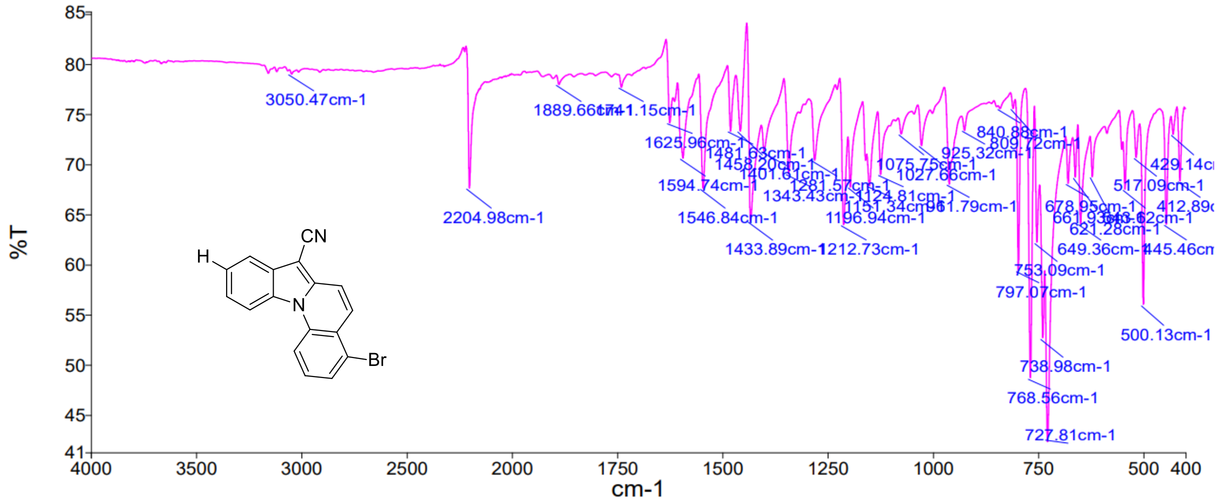

^{1}H NMR spectrum (400 MHz, $CDCl_3$) of compound **5bn**

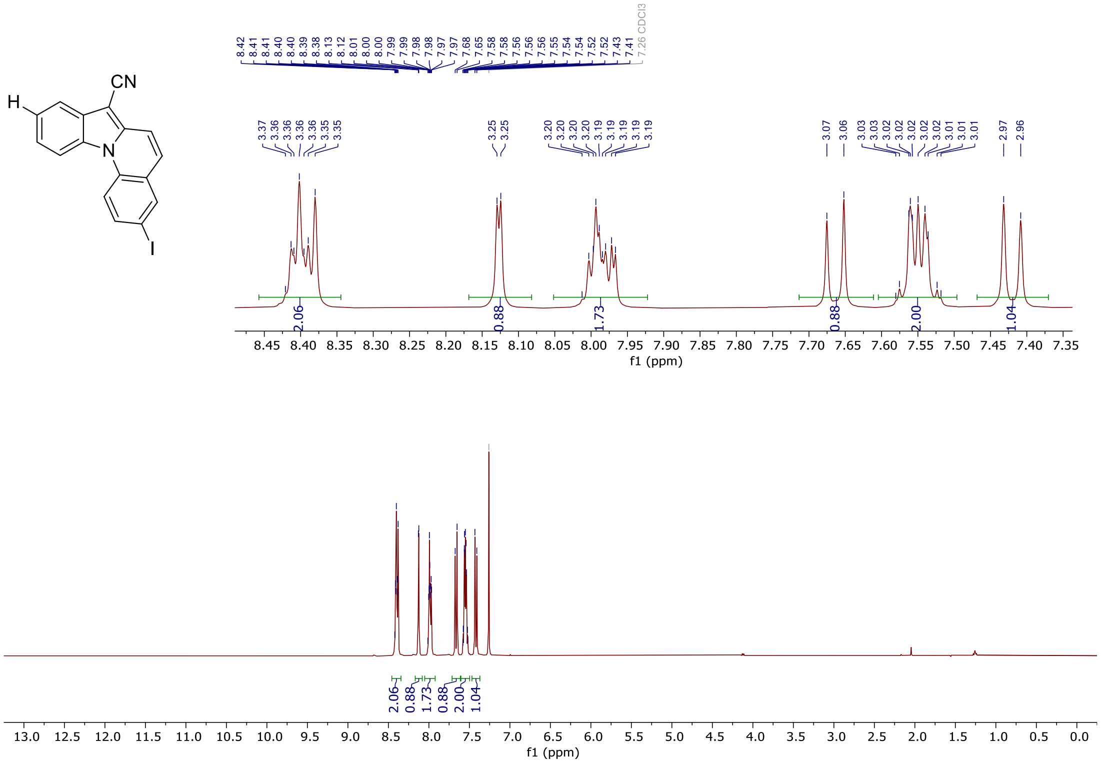

$^{13}C\{^{1}H\}$ NMR spectrum (100 MHz, $CDCl_3$) of compound **5bn**

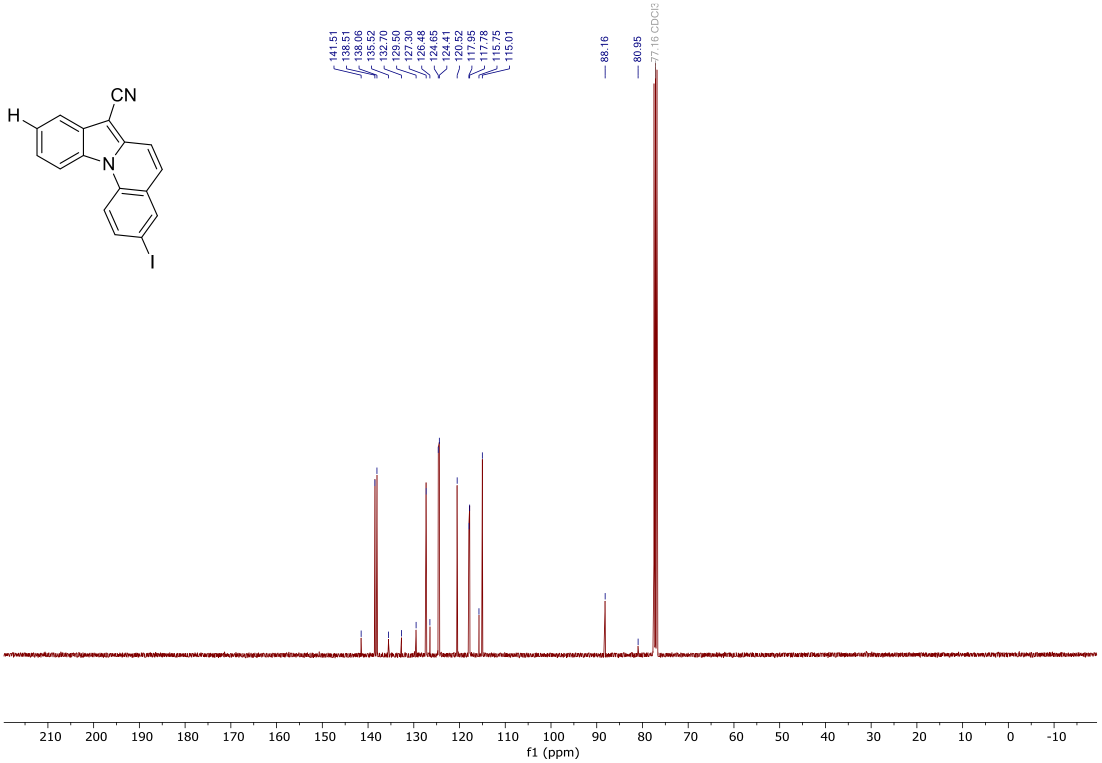

IR spectrum (neat) of compound **5bn**

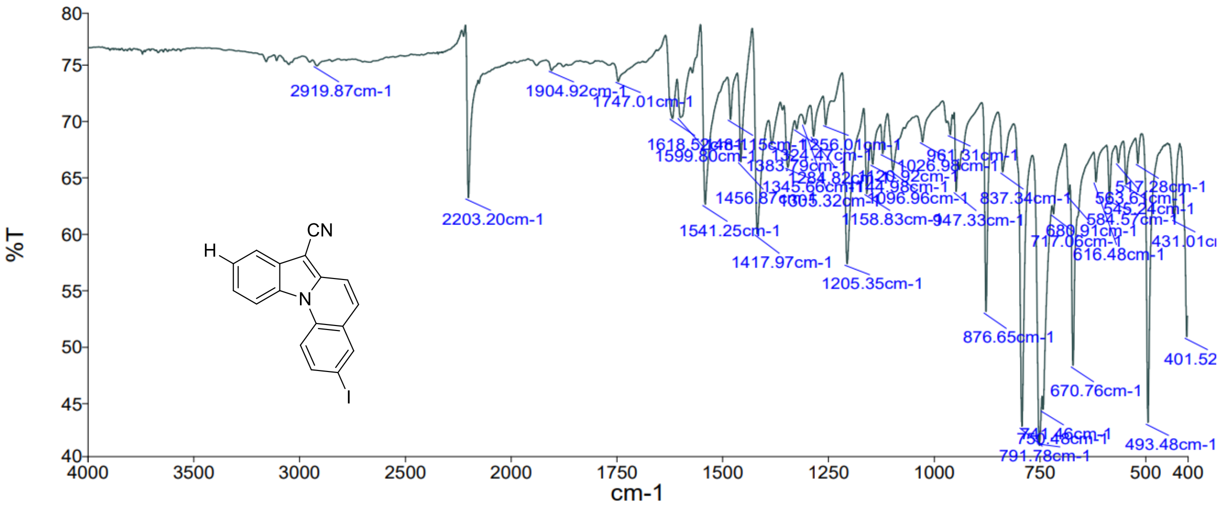

^{1}H NMR spectrum (400 MHz, DMSO-d_6) of compound **5bo**

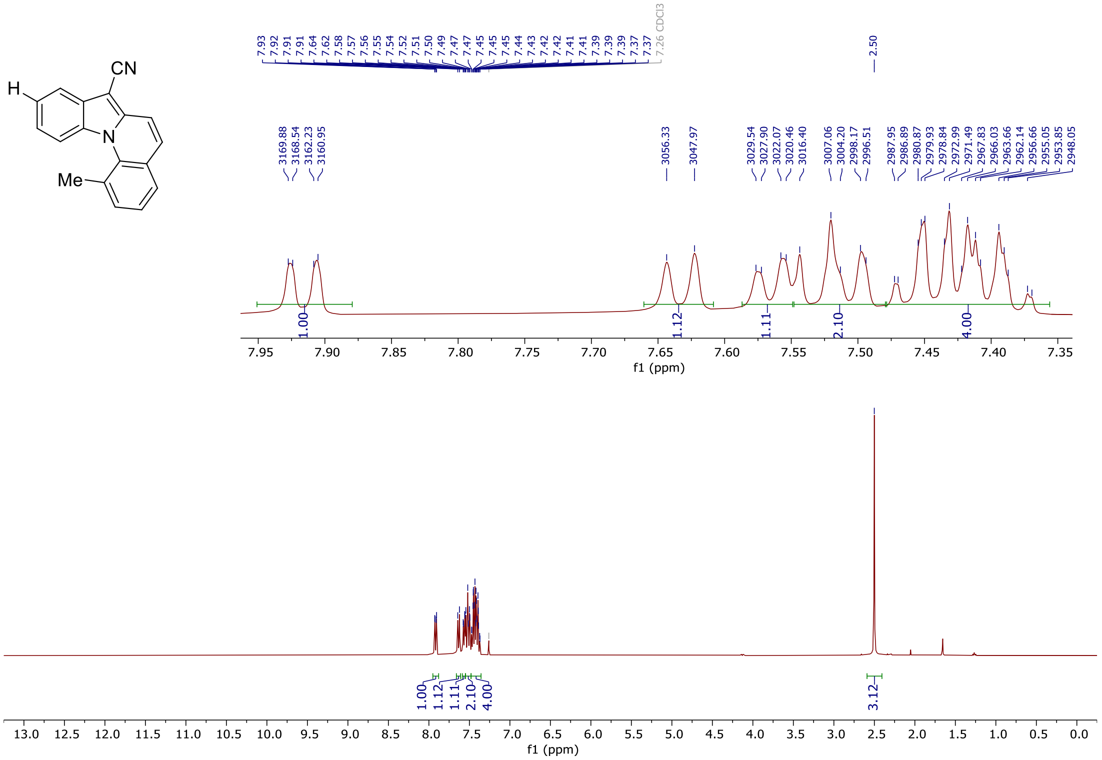

^{13}C{^{1}H} NMR spectrum (100 MHz, $CDCl_3$) of compound **5bo**

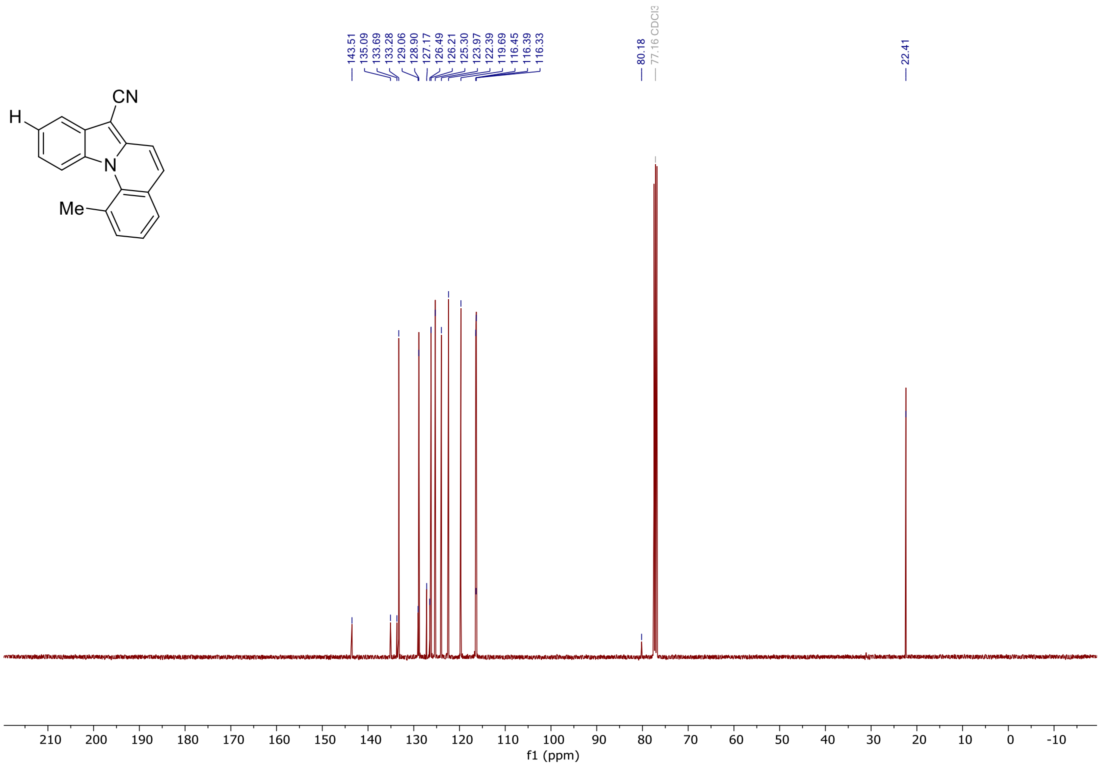

IR spectrum (neat) of compound **5bo**

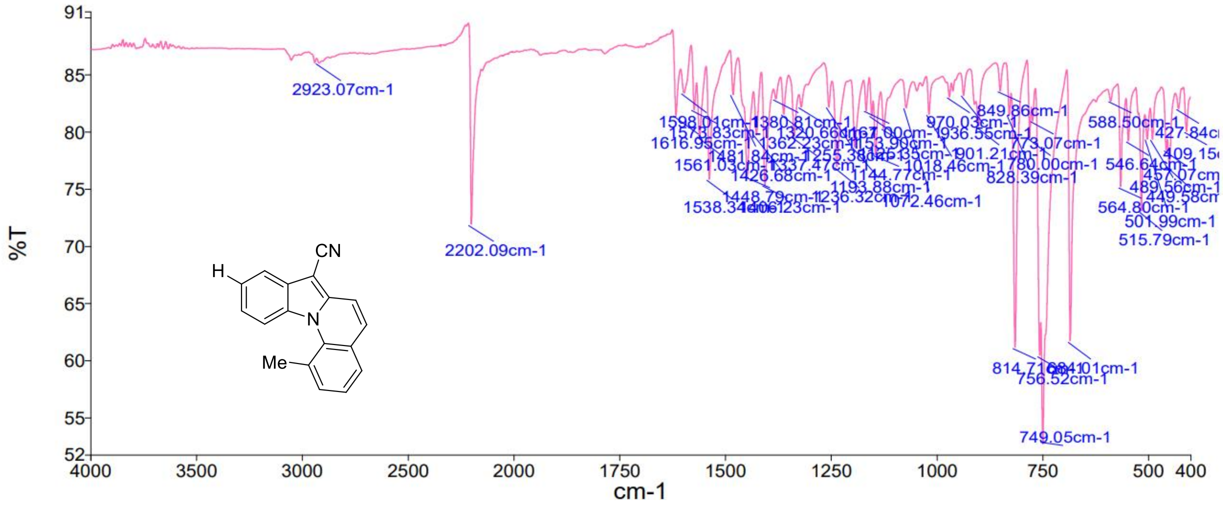

^{1}H NMR spectrum (400 MHz, $CDCl_3$) of compound **5bp**

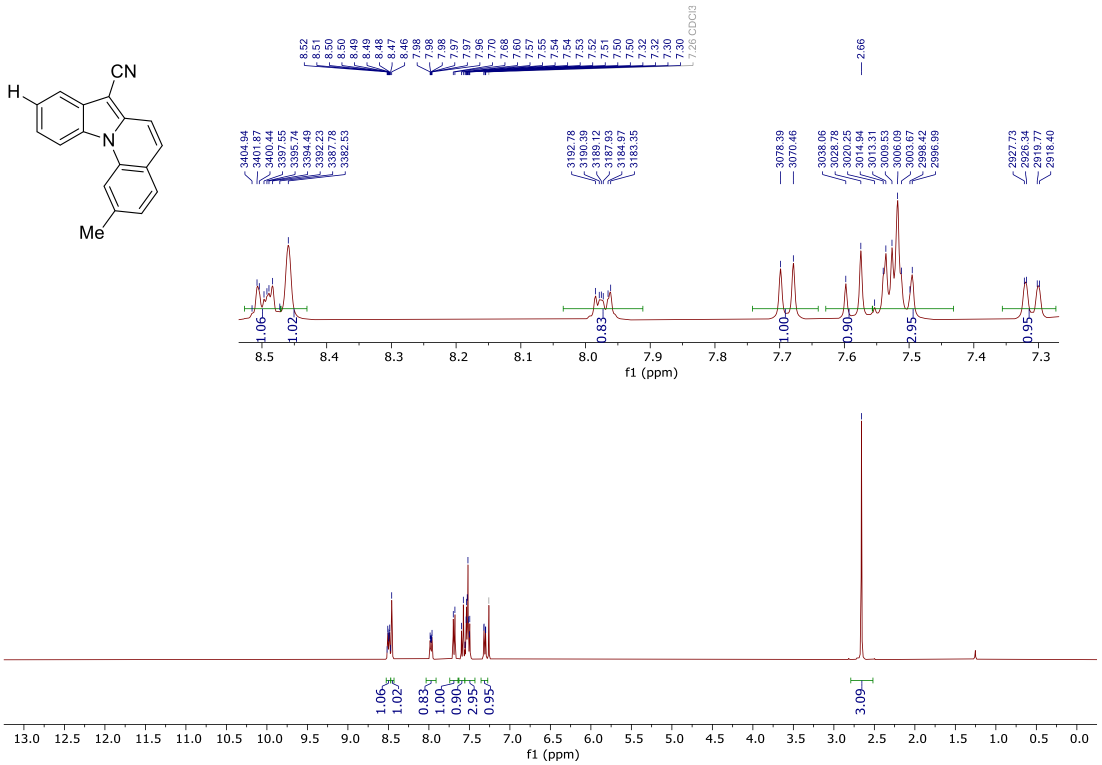

$^{13}C\{^{1}H\}$ NMR spectrum (100 MHz, $CDCl_3$) of compound **5bp**

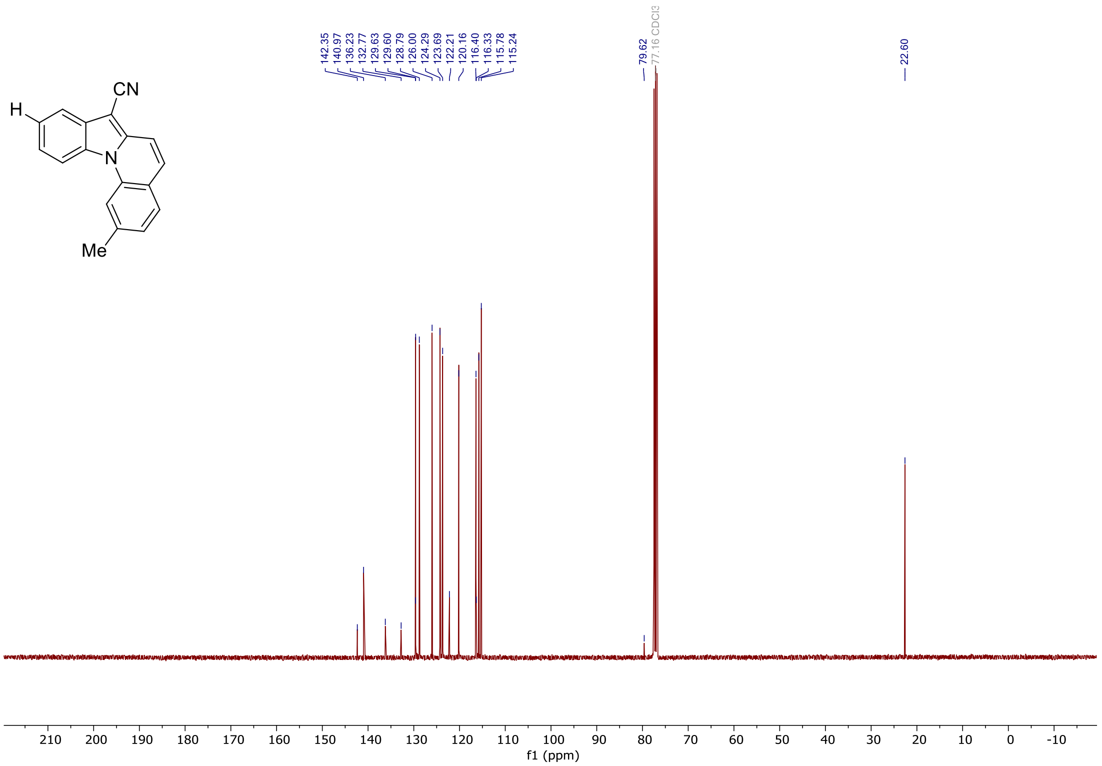

IR spectrum (neat) of compound **5bp**

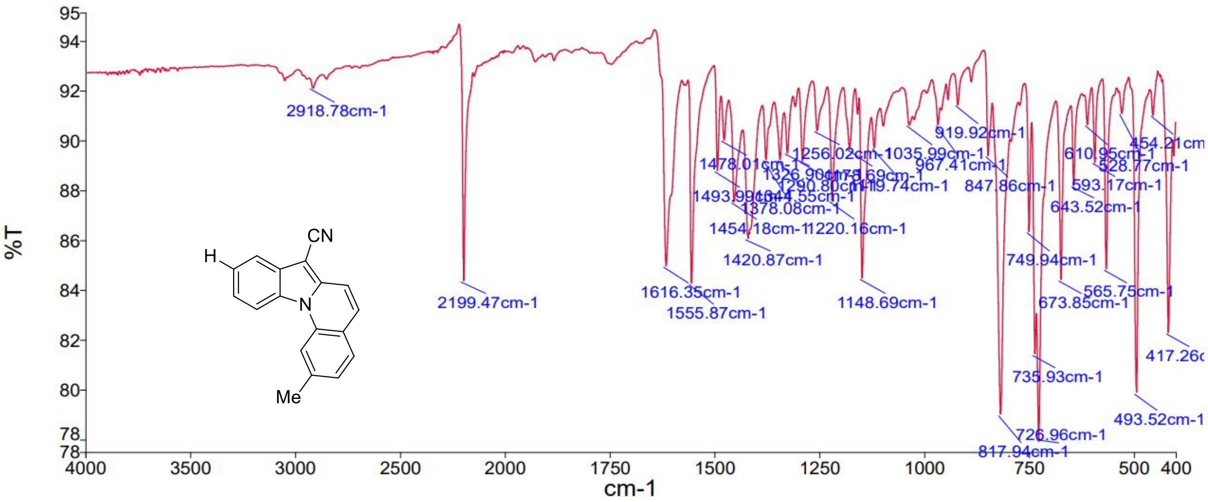

^{1}H NMR spectrum (400 MHz, $CDCl_3$) of compound **5bq**

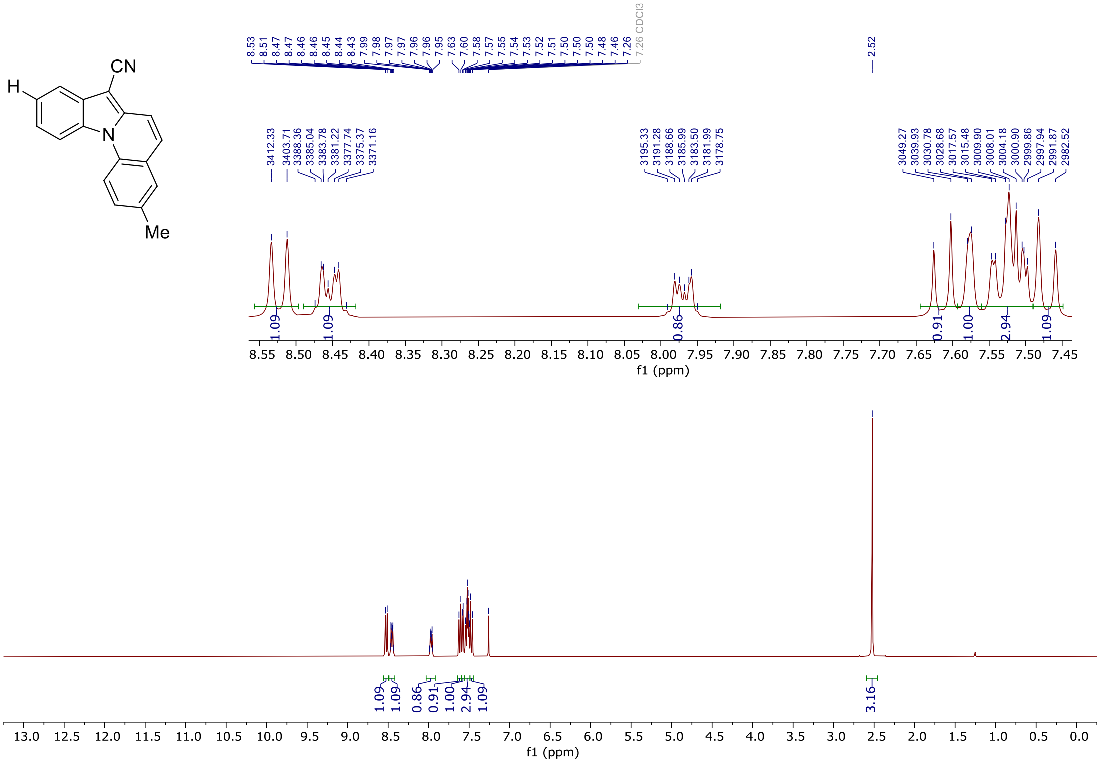

$^{13}C\{^{1}H\}$ NMR spectrum (100 MHz, $CDCl_3$) of compound **5bq**

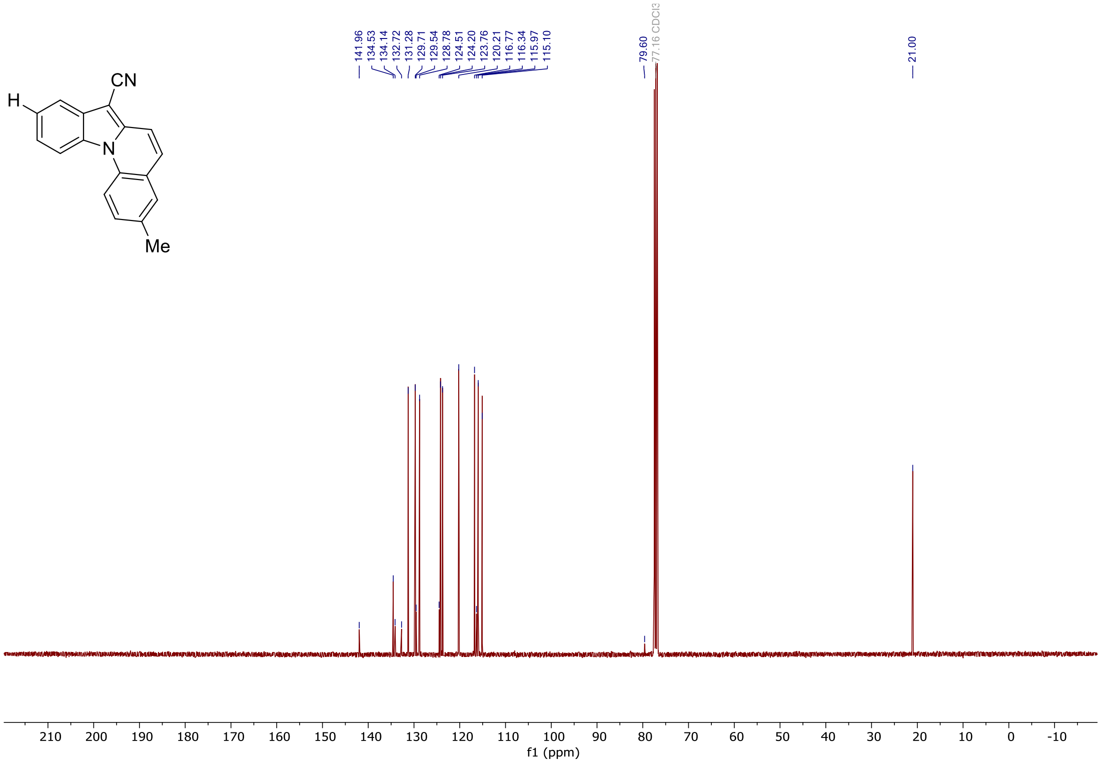

IR spectrum (neat) of compound **5bq**

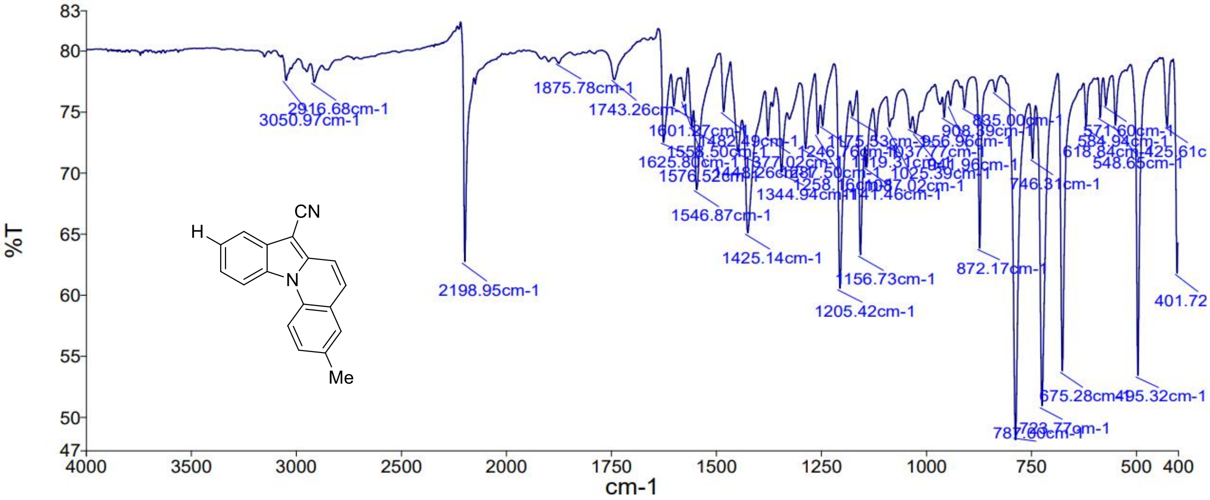

^{1}H NMR spectrum (400 MHz, $CDCl_3$) of compound **5br**

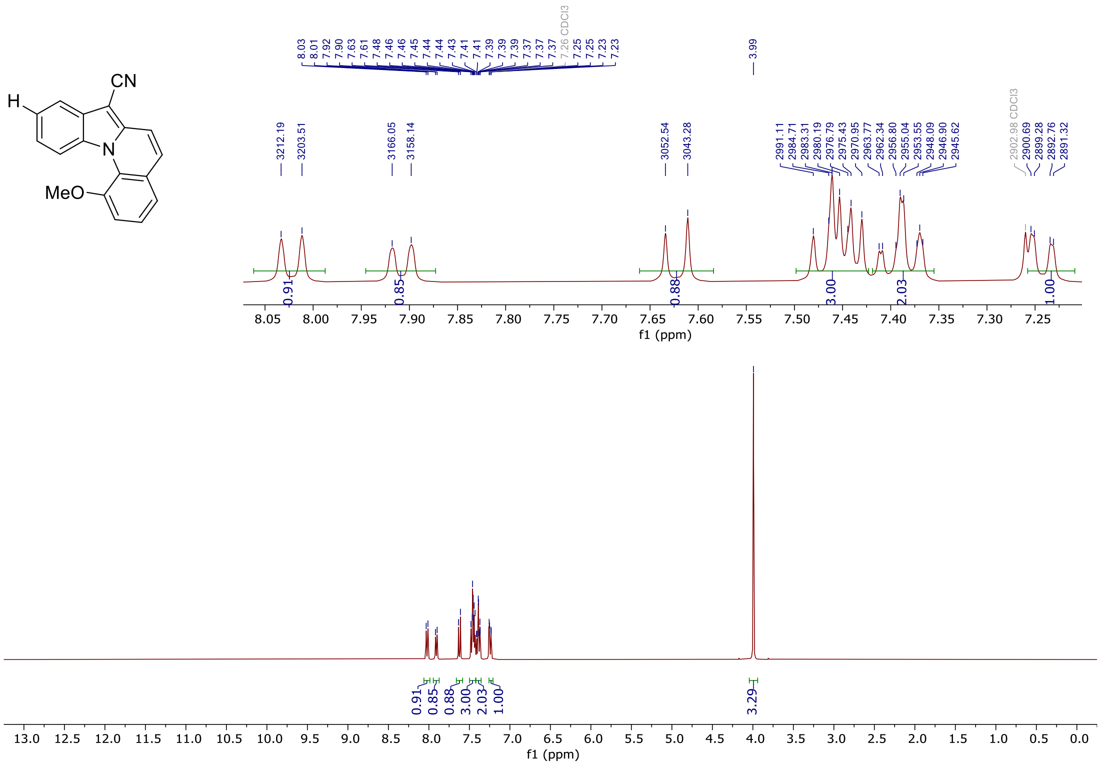

$^{13}C\{^{1}H\}$ NMR spectrum (100 MHz, $CDCl_3$) of compound **5br**

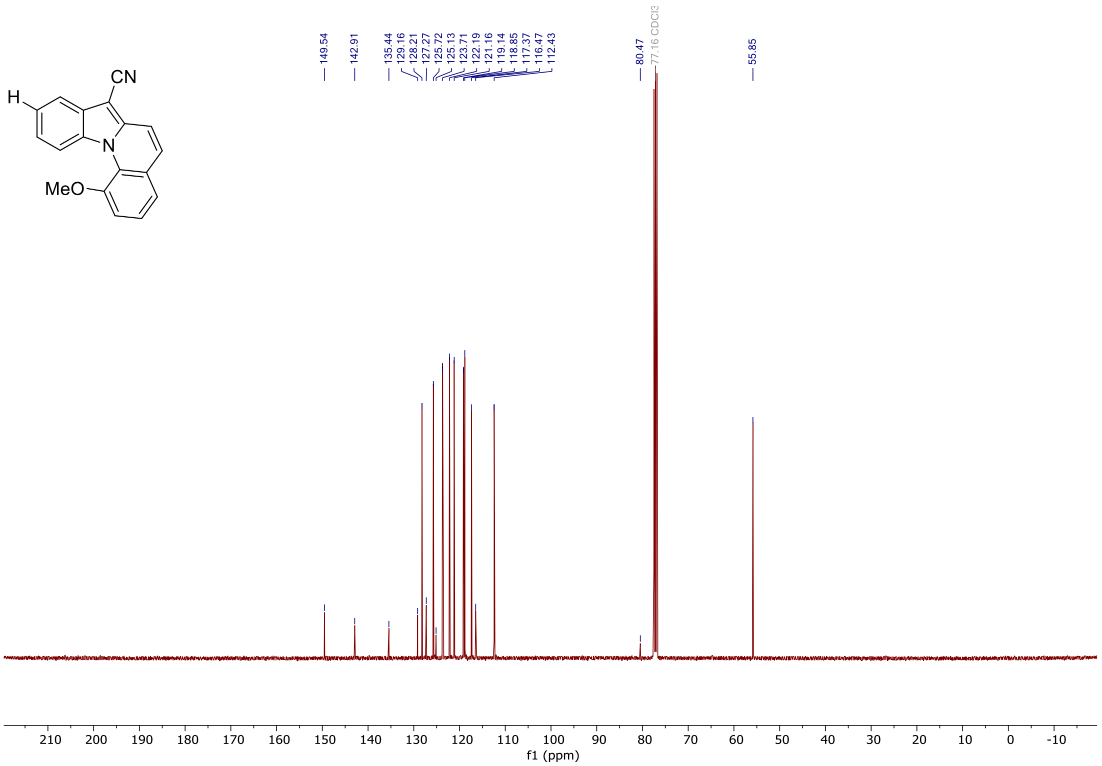

IR spectrum (neat) of compound **5br**

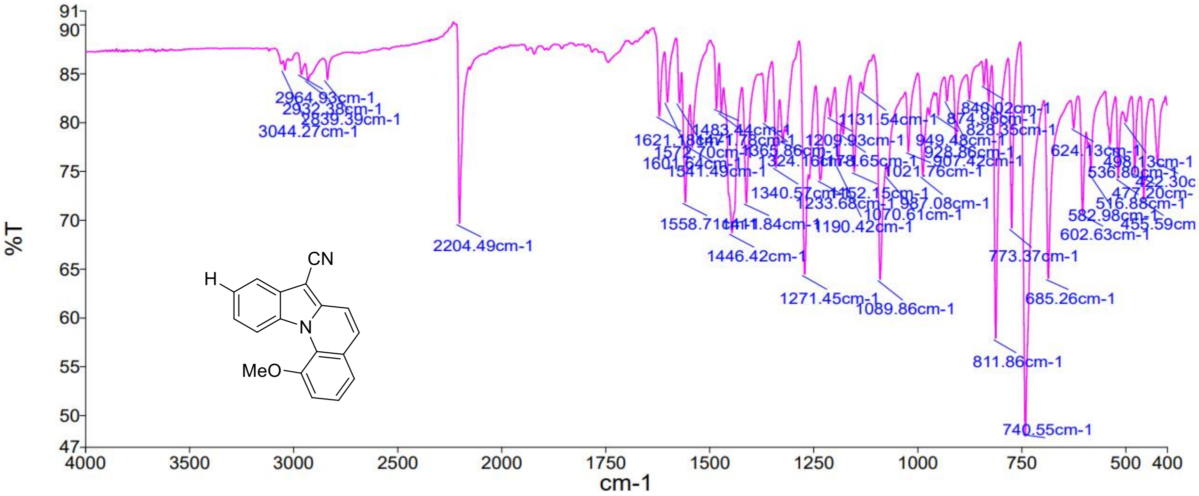

^{1}H NMR spectrum (400 MHz, $CDCl_3$) of compound **5bs**

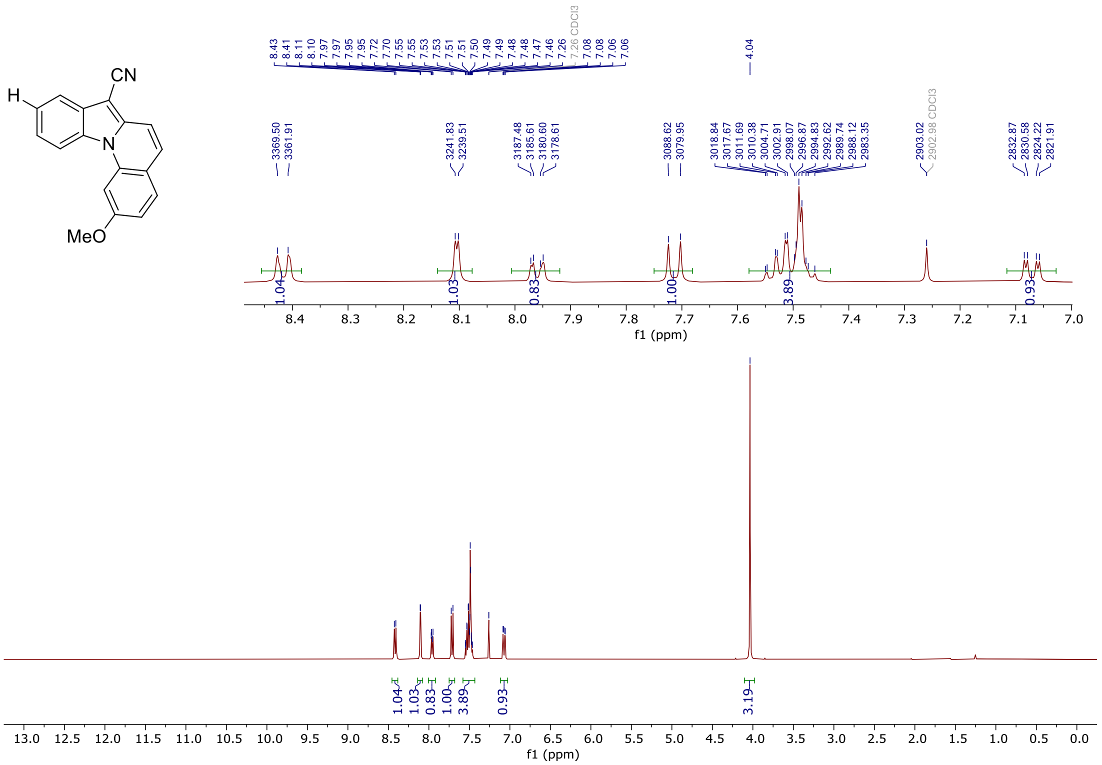

^{13}C{^{1}H} NMR spectrum (100 MHz, $CDCl_3$) of compound **5bs**

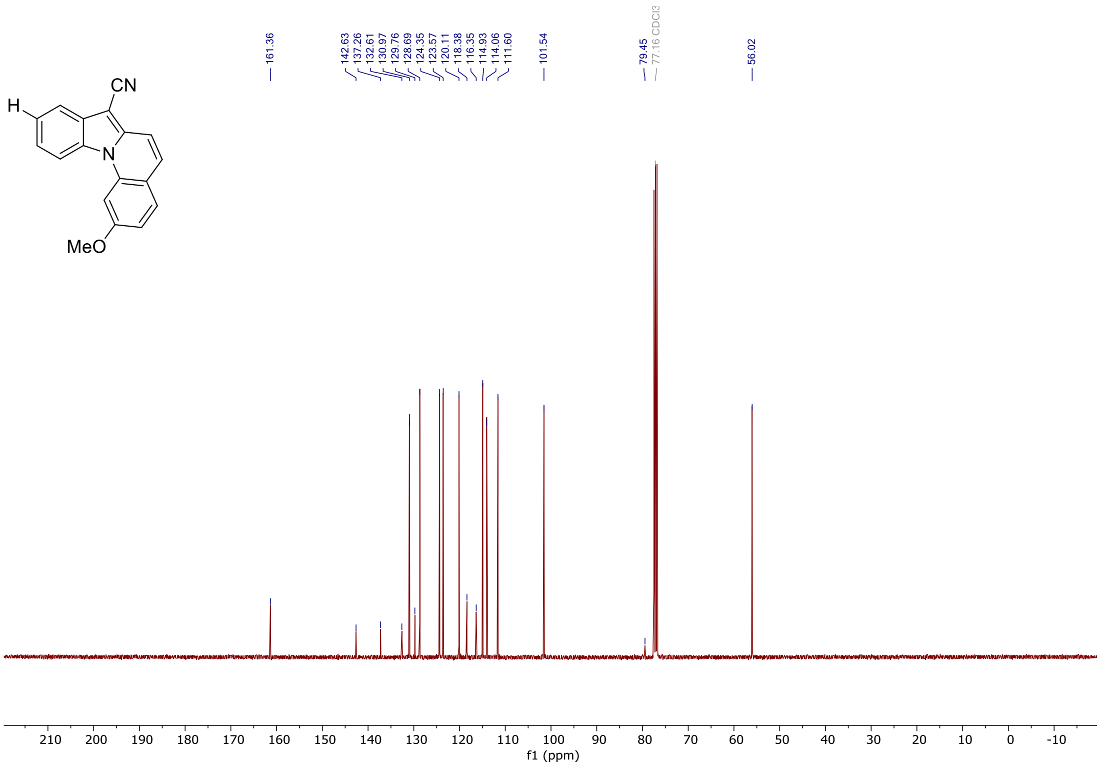

IR spectrum (neat) of compound **5bs**

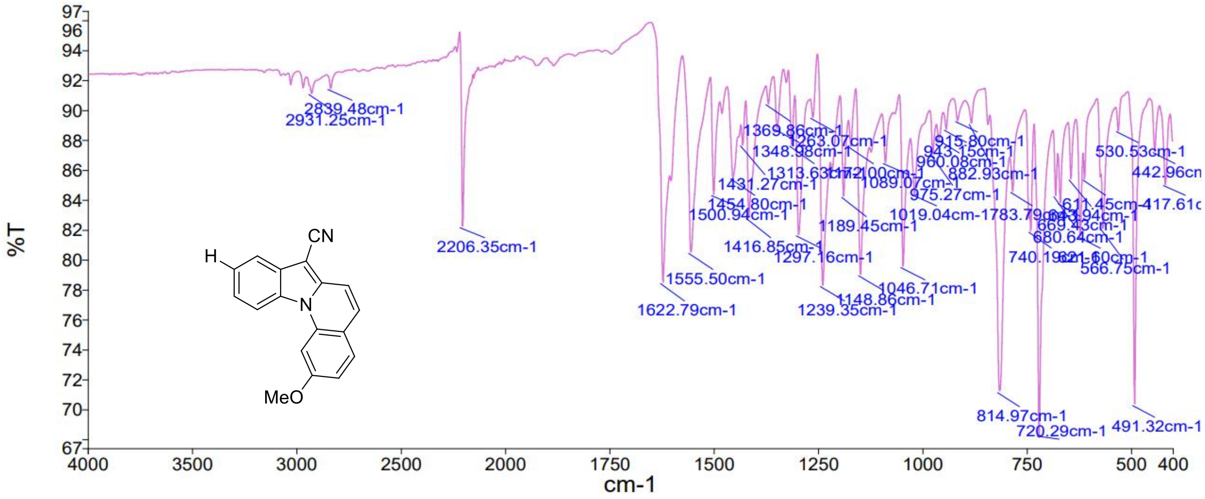

^{1}H NMR spectrum (400 MHz, $CDCl_3$) of compound **5bt**

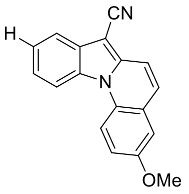

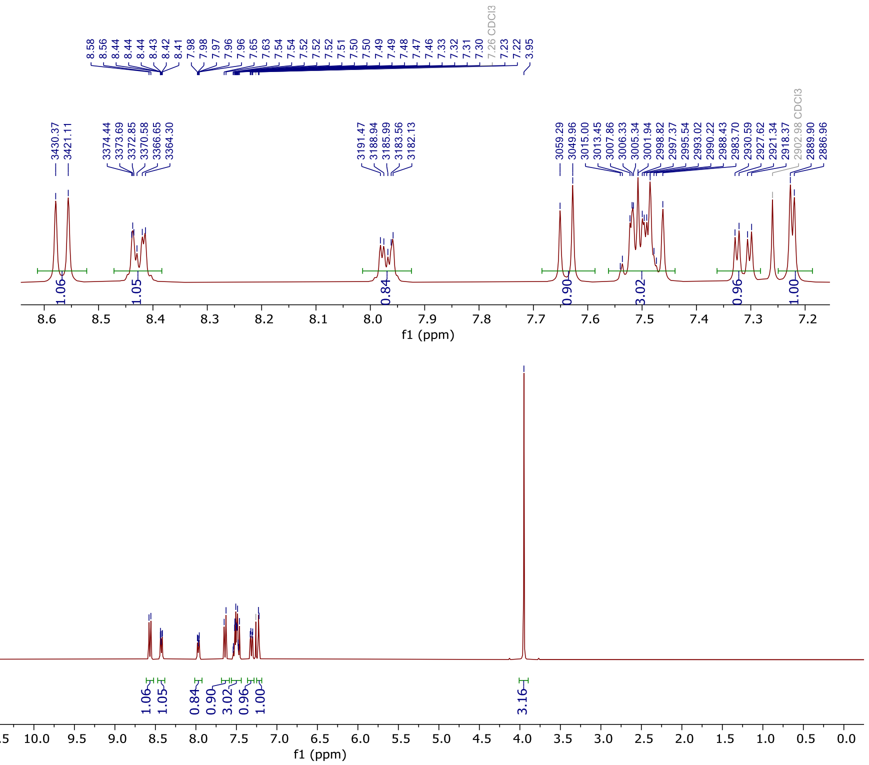

^{13}C{^{1}H} NMR spectrum (100 MHz, $CDCl_3$) of compound **5bt**

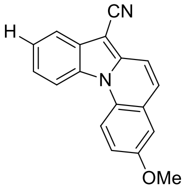

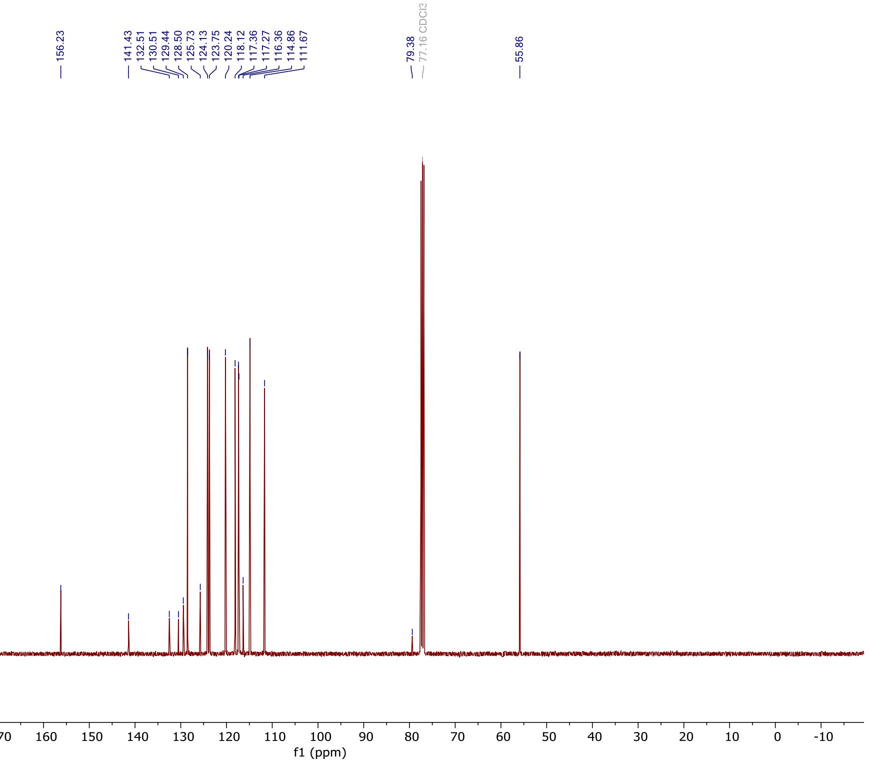

IR spectrum (neat) of compound **5bt**

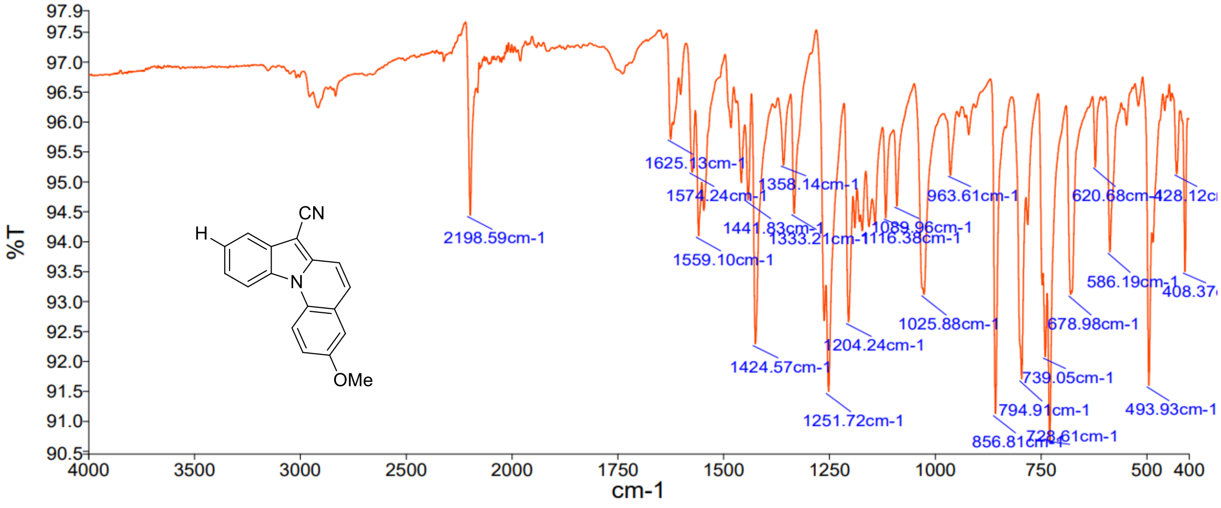

^{1}H NMR spectrum (400 MHz, $CDCl_3$) of compound **5bu**

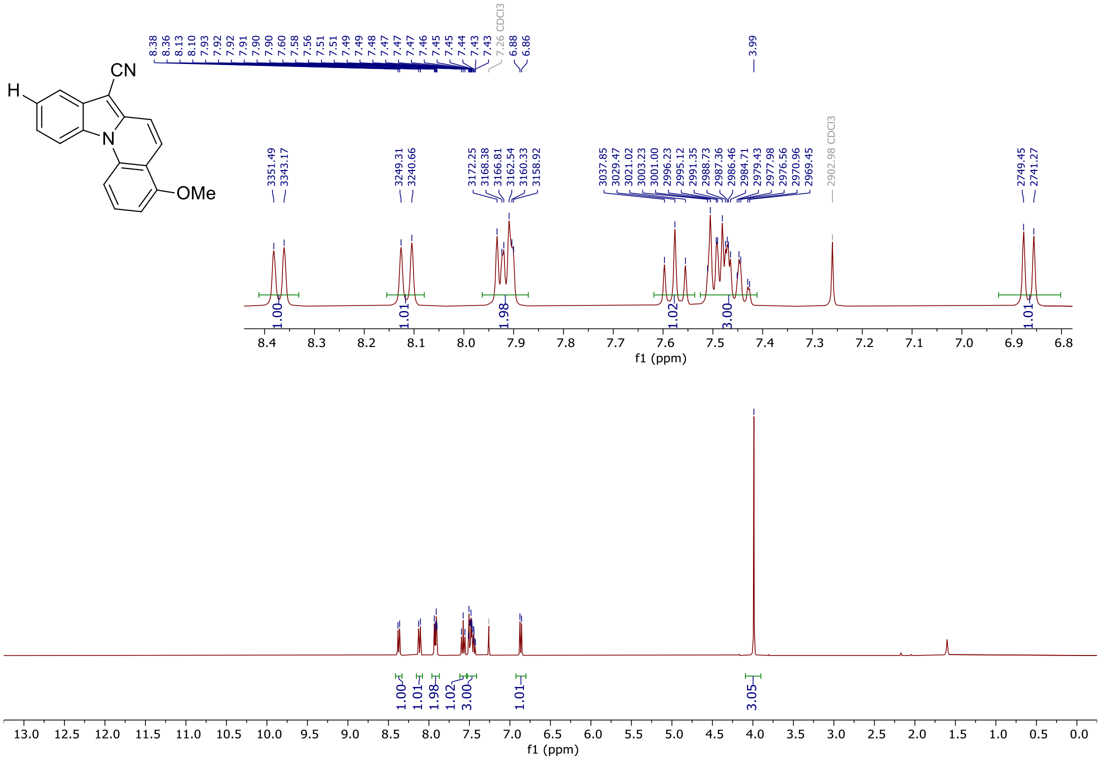

$^{13}C\{^1H\}$ NMR spectrum (100 MHz, $CDCl_3$) of compound **5bu**

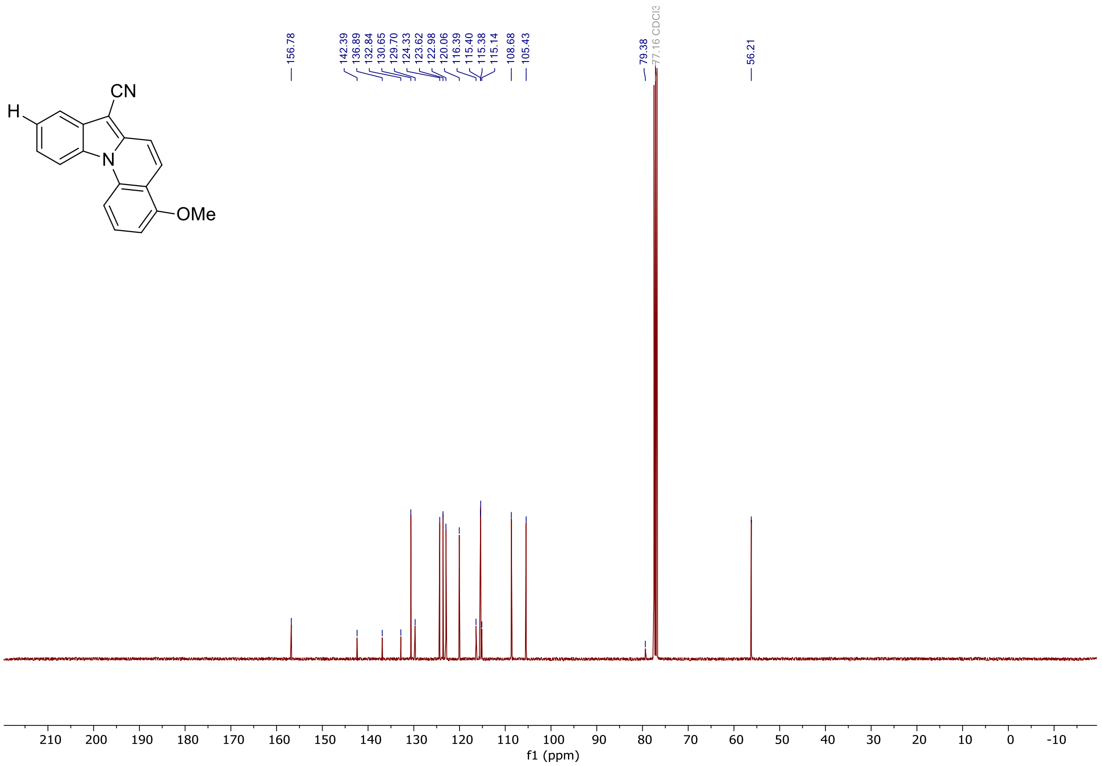

IR spectrum (neat) of compound **5bu**

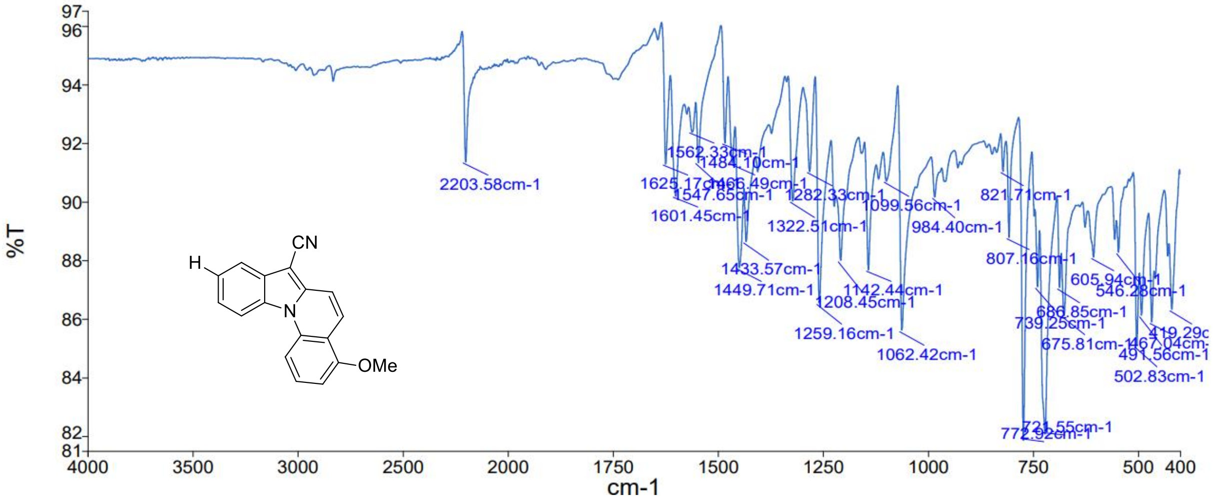

^{1}H NMR spectrum (400 MHz, $CDCl_3$) of compound **5cb**

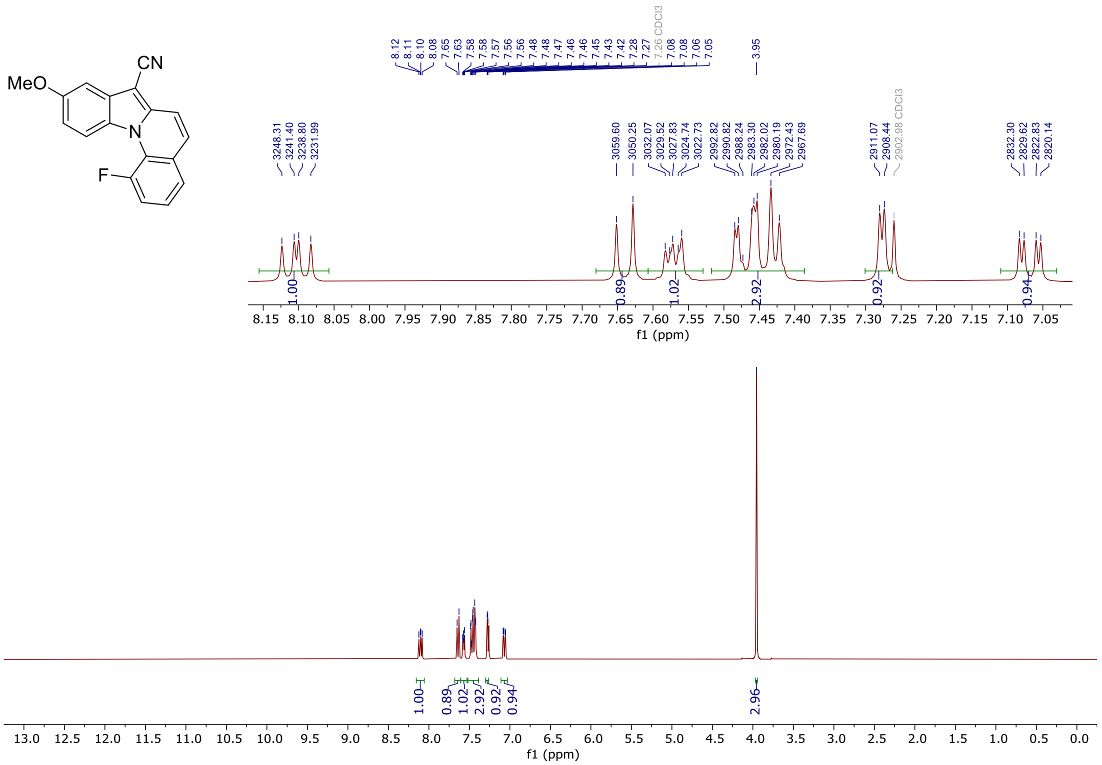

$^{13}C\{^1H\}$ NMR spectrum (100 MHz, $CDCl_3$) of compound **5cb**

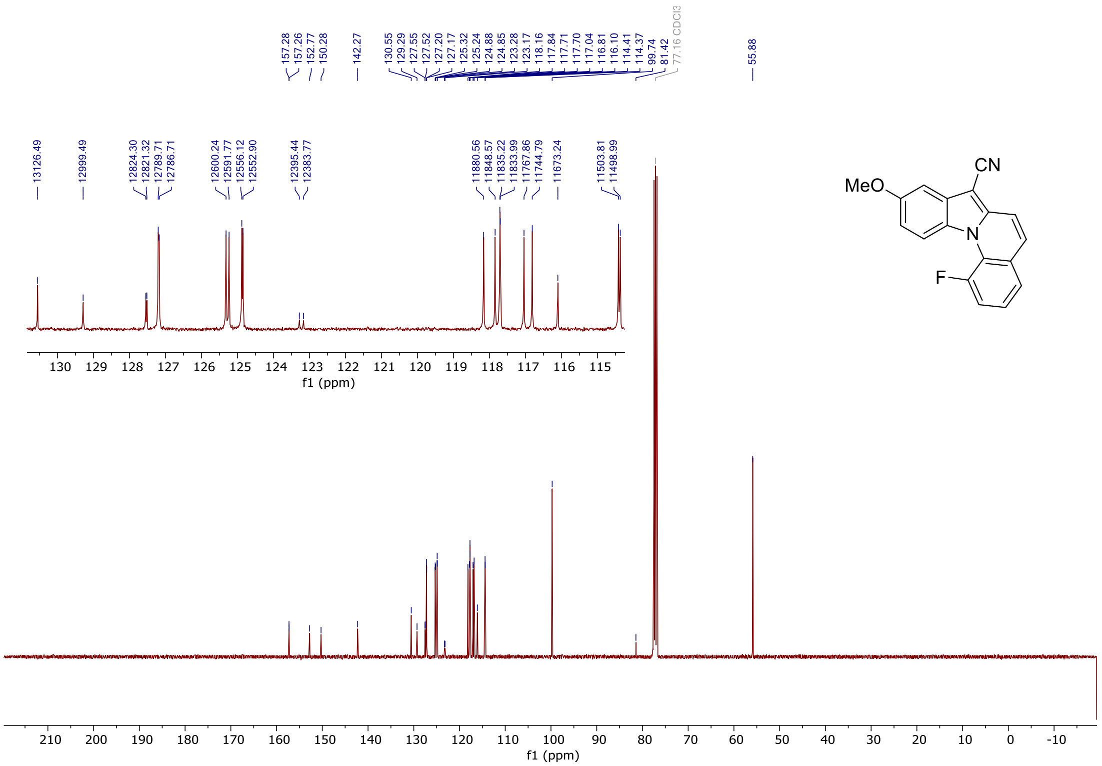

^{19}F NMR spectrum (500 MHz, $CDCl_3$) of compound **5cb**

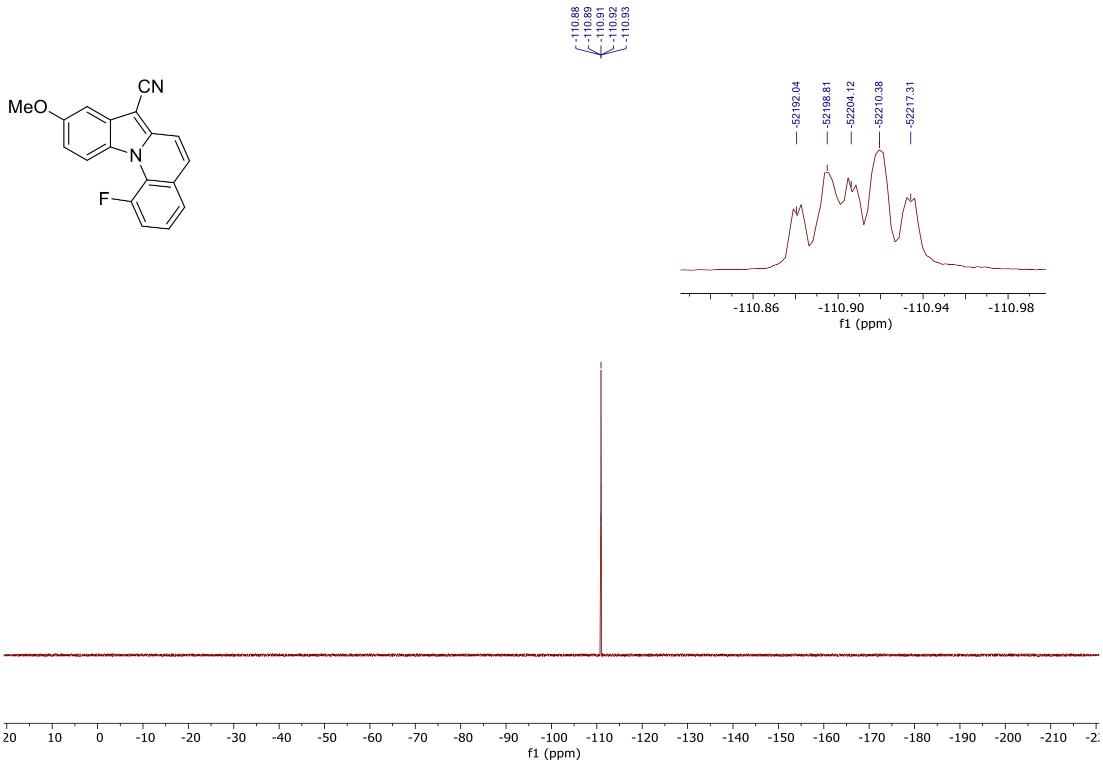

^{19}F NMR spectrum (500 MHz, $CDCl_3$) of compound **5cb**

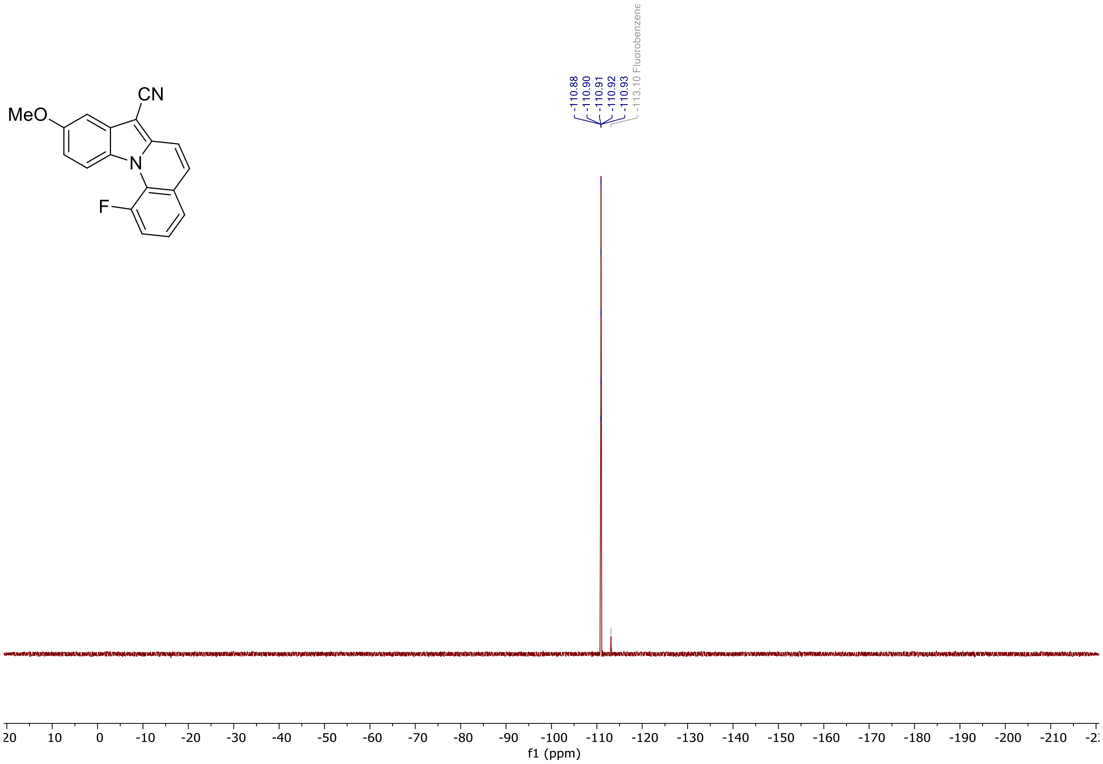

IR spectrum (neat) of compound **5cb**

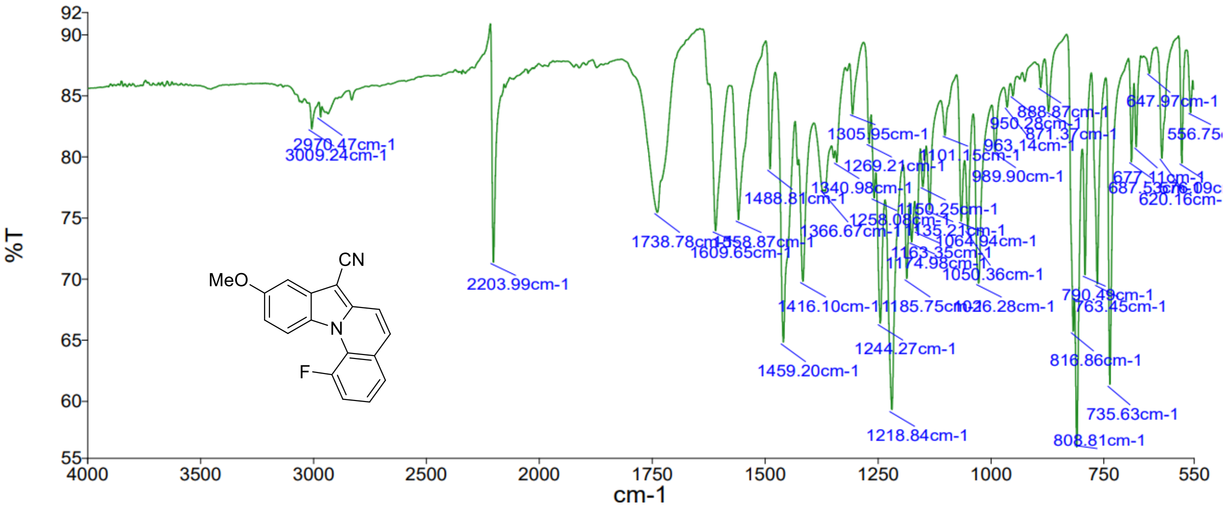

^{1}H NMR spectrum (400 MHz, $CDCl_3$) of compound **5cc**

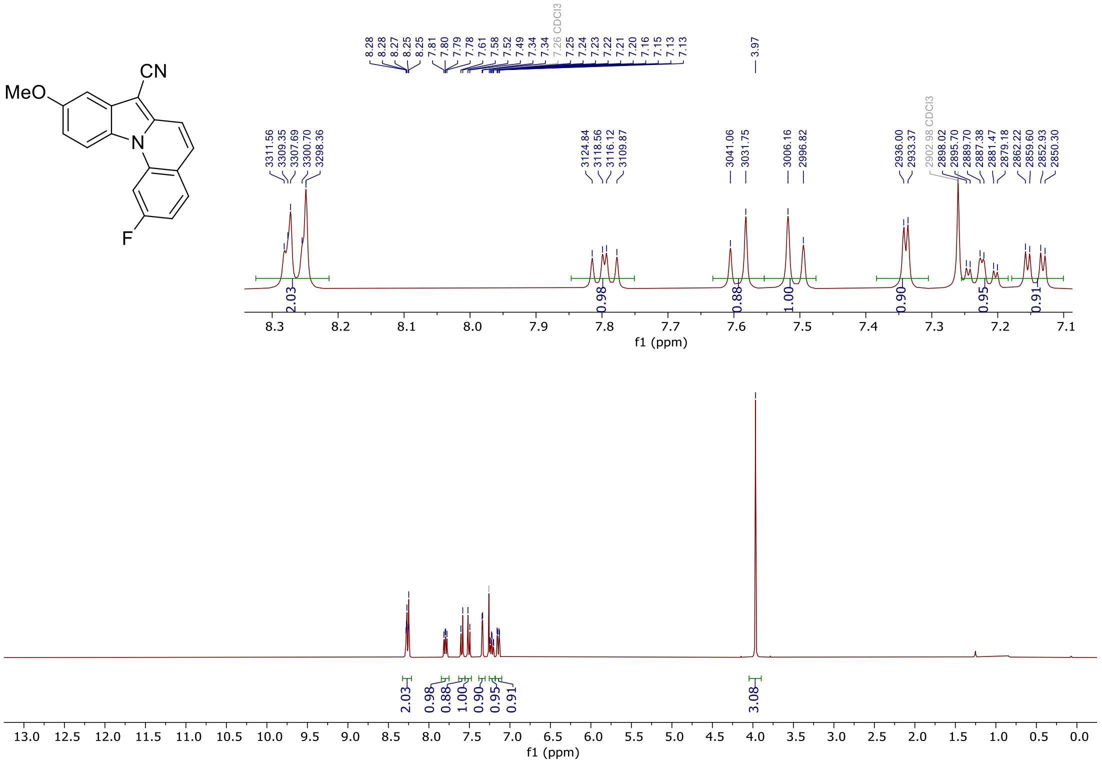

$^{13}C\{^{1}H\}$ NMR spectrum (100 MHz, $CDCl_3$) of compound **5cc**

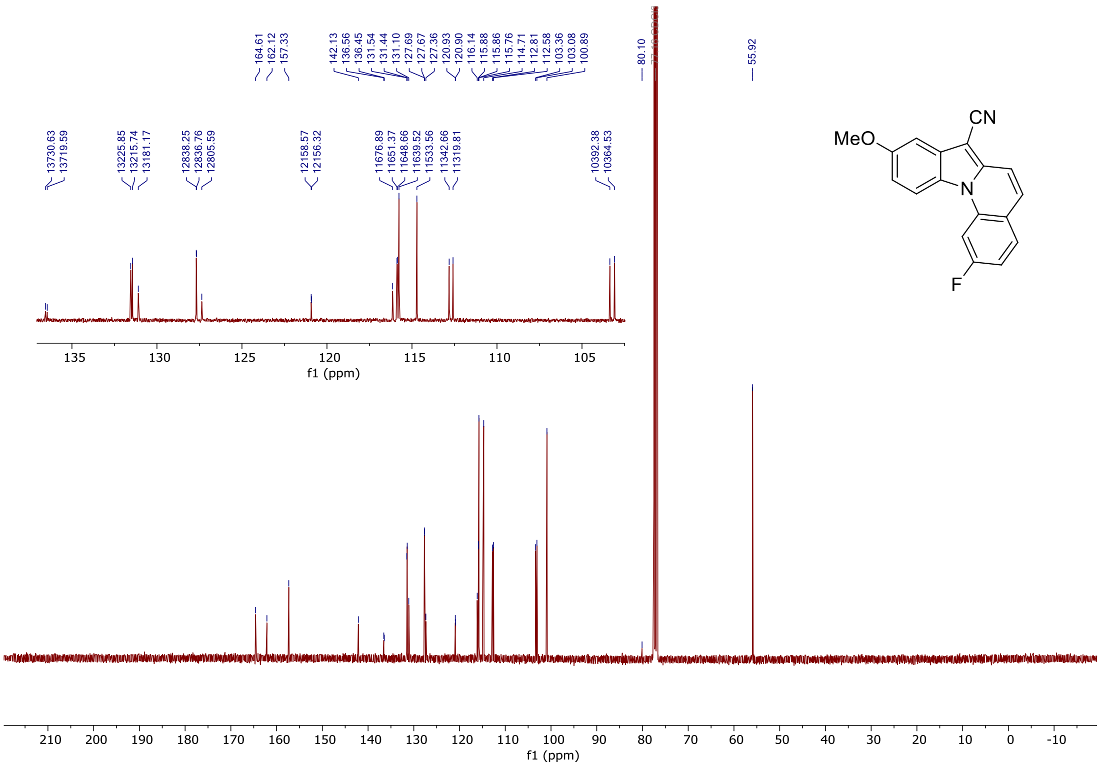

^{19}F NMR spectrum (470 MHz, $CDCl_3$) of compound **5cc**

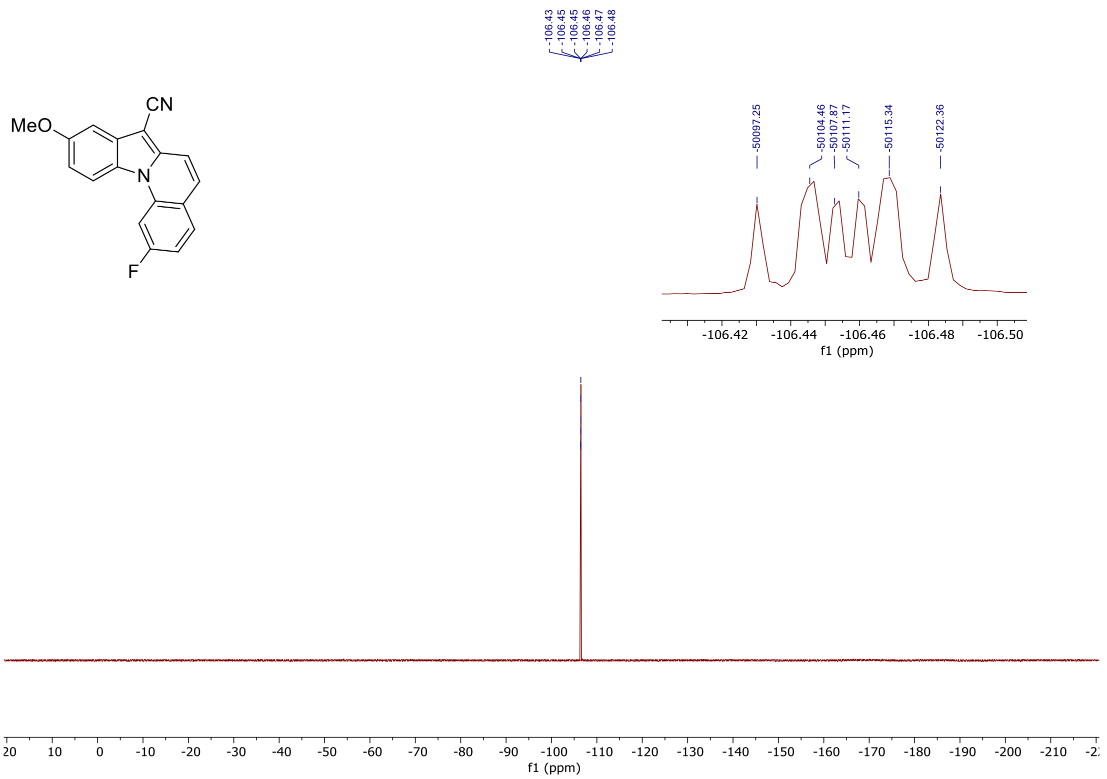

^{19}F NMR spectrum (470 MHz, $CDCl_3$) of compound **5cc** with an internal standard (fluorobenzene)

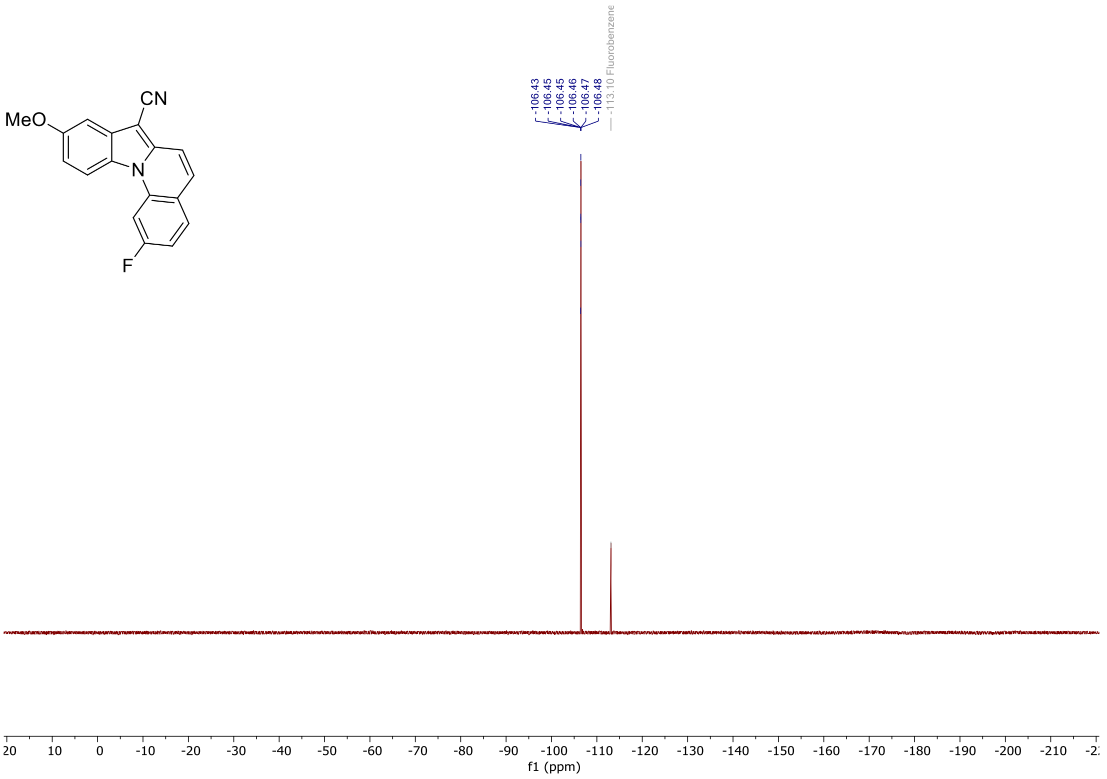

IR spectrum (neat) of compound **5cc**

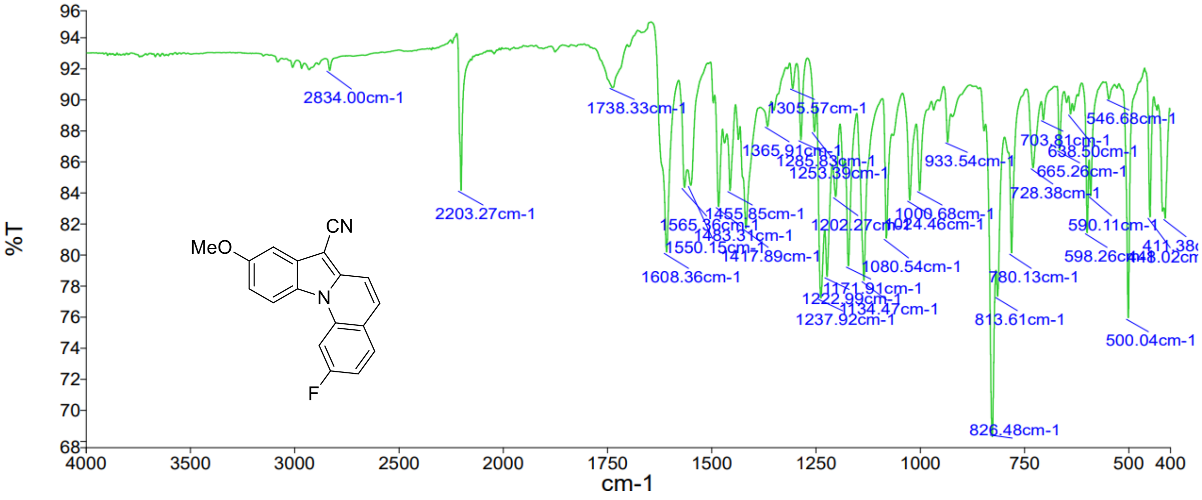

^{1}H NMR spectrum (400 MHz, $CDCl_3$) of compound **5cd**

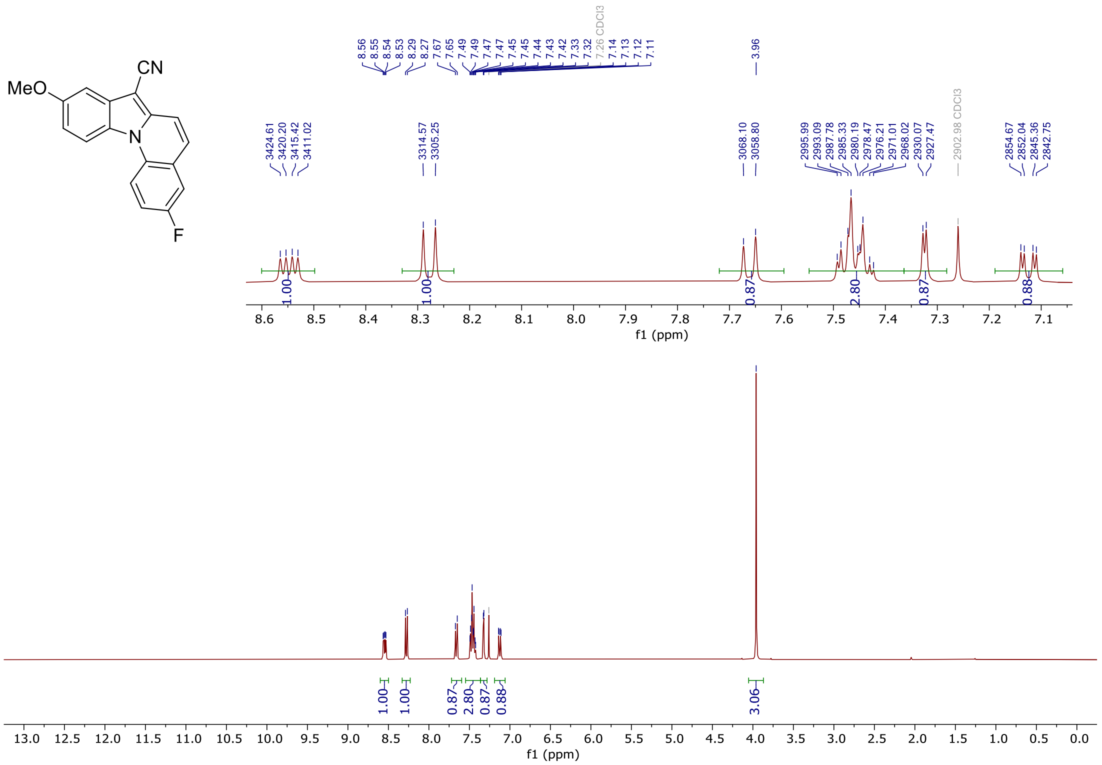

^{13}C{^{1}H} NMR spectrum (100 MHz, $CDCl_3$) of compound **5cd**

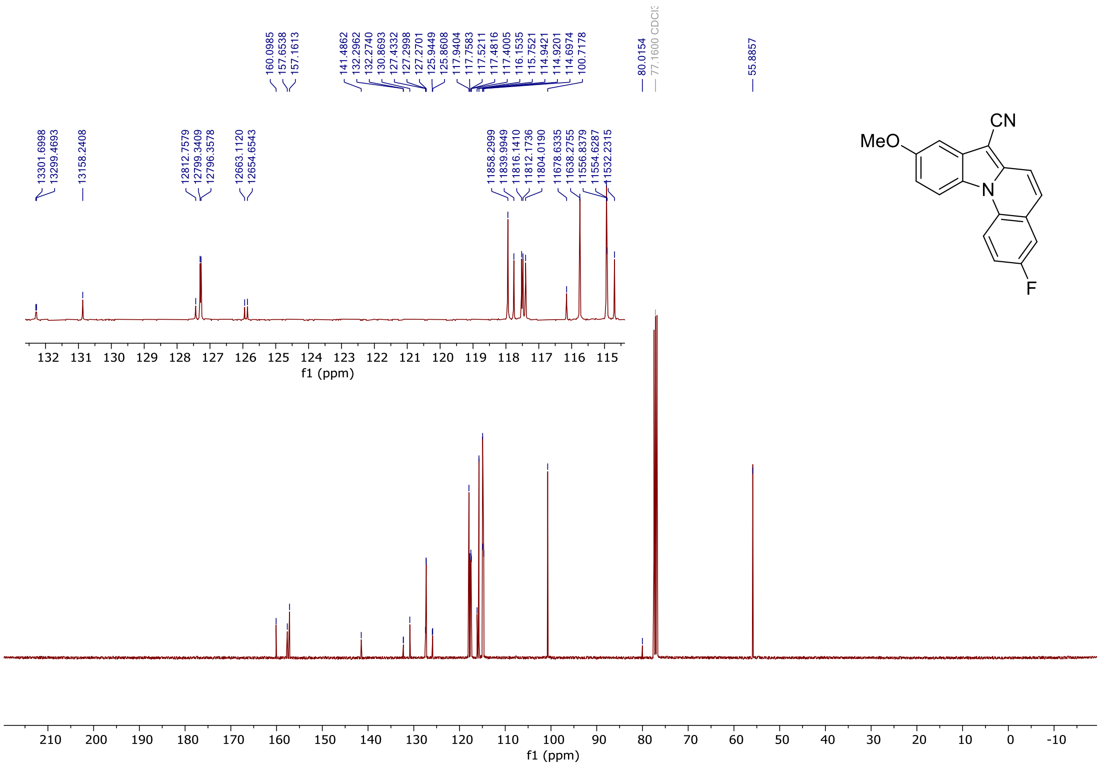

^{19}F NMR spectrum (500 MHz, $CDCl_3$) of compound **5cd**

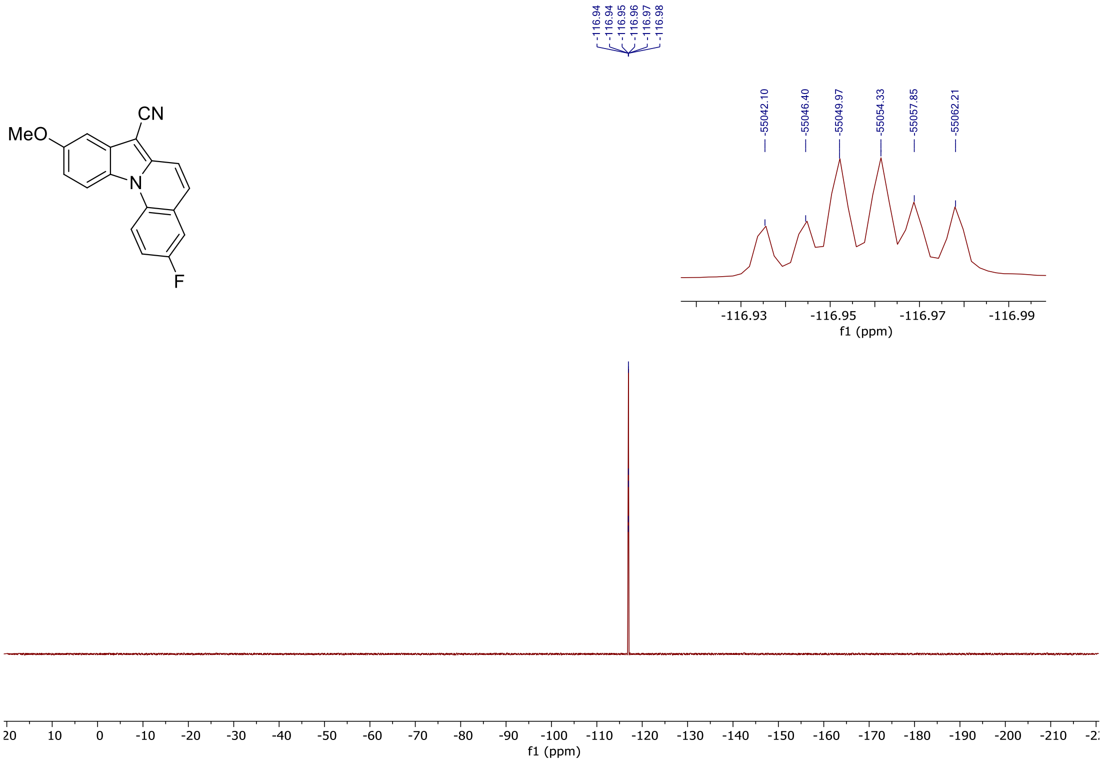

^{19}F NMR spectrum (500 MHz, $CDCl_3$) of compound **5cd** with an internal standard (fluorobenzene)

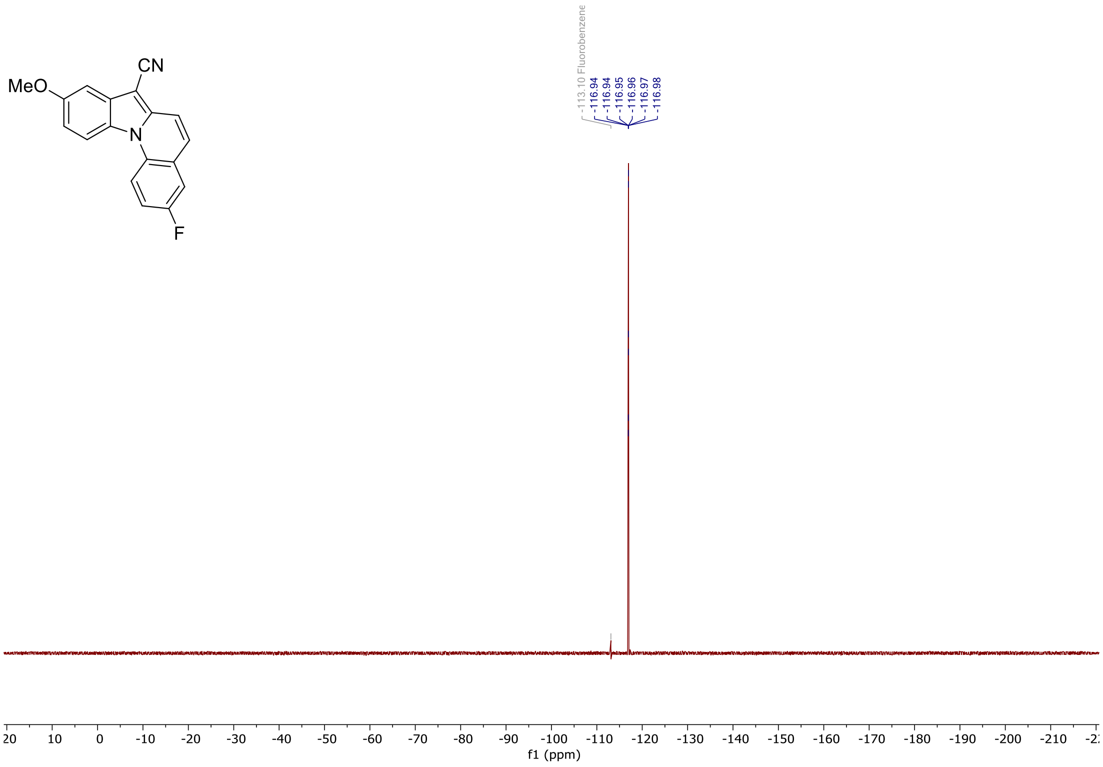

IR spectrum (neat) of compound **5cd**

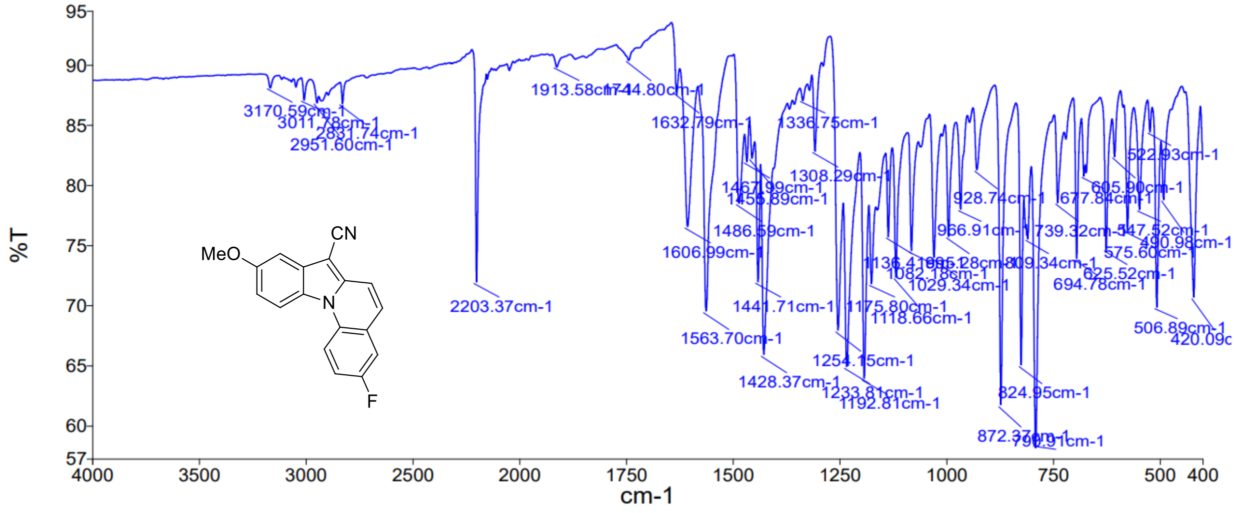

^{1}H NMR spectrum (400 MHz, $CDCl_3$) of compound **5ce**

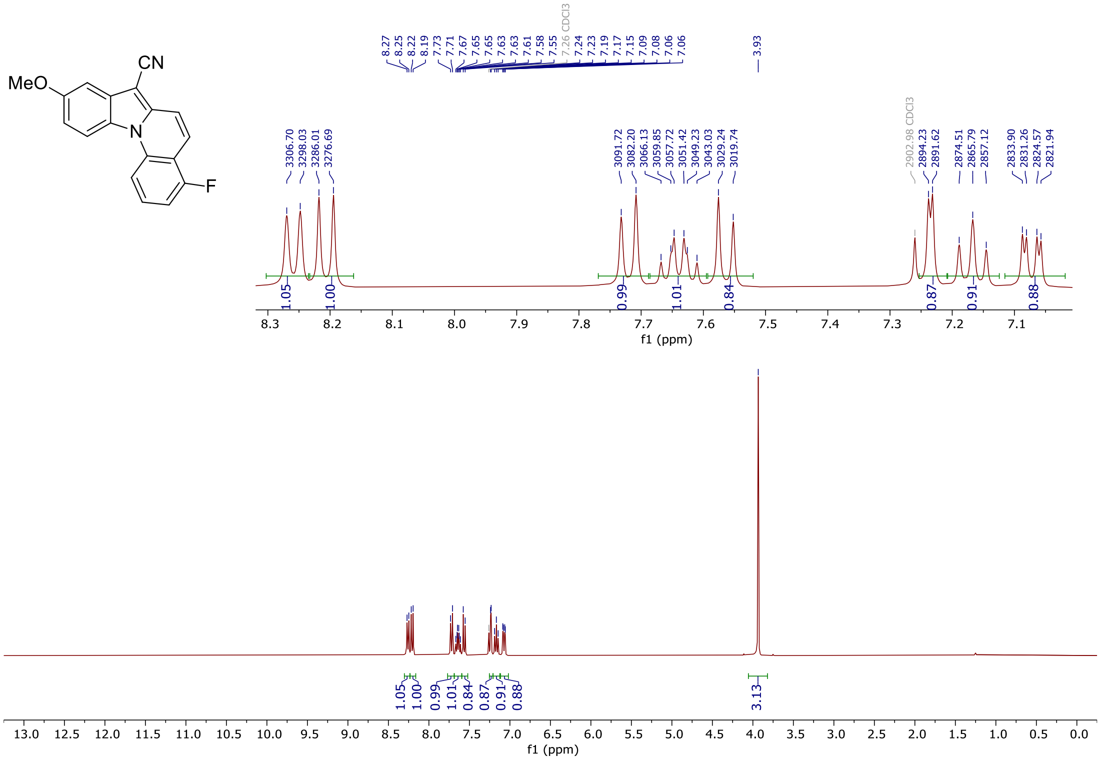

$^{13}C\{^{1}H\}$ NMR spectrum (100 MHz, $CDCl_3$) of compound **5ce**

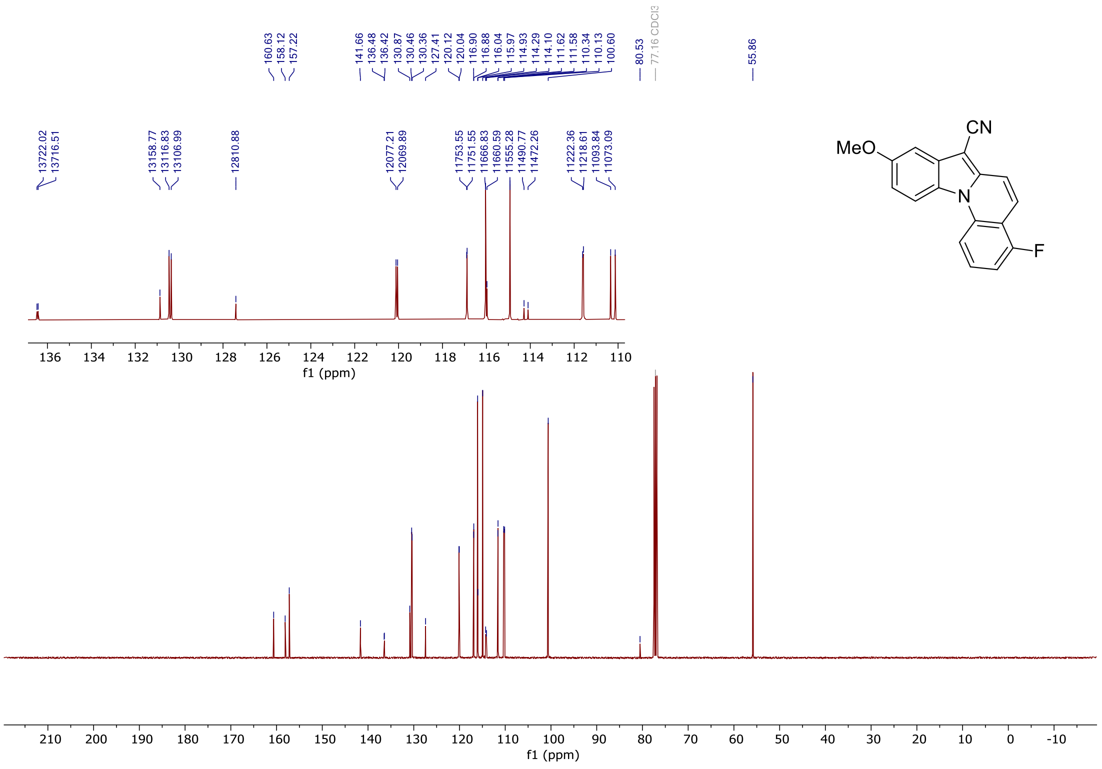

^{19}F NMR spectrum (500 MHz, $CDCl_3$) of compound **5ce**

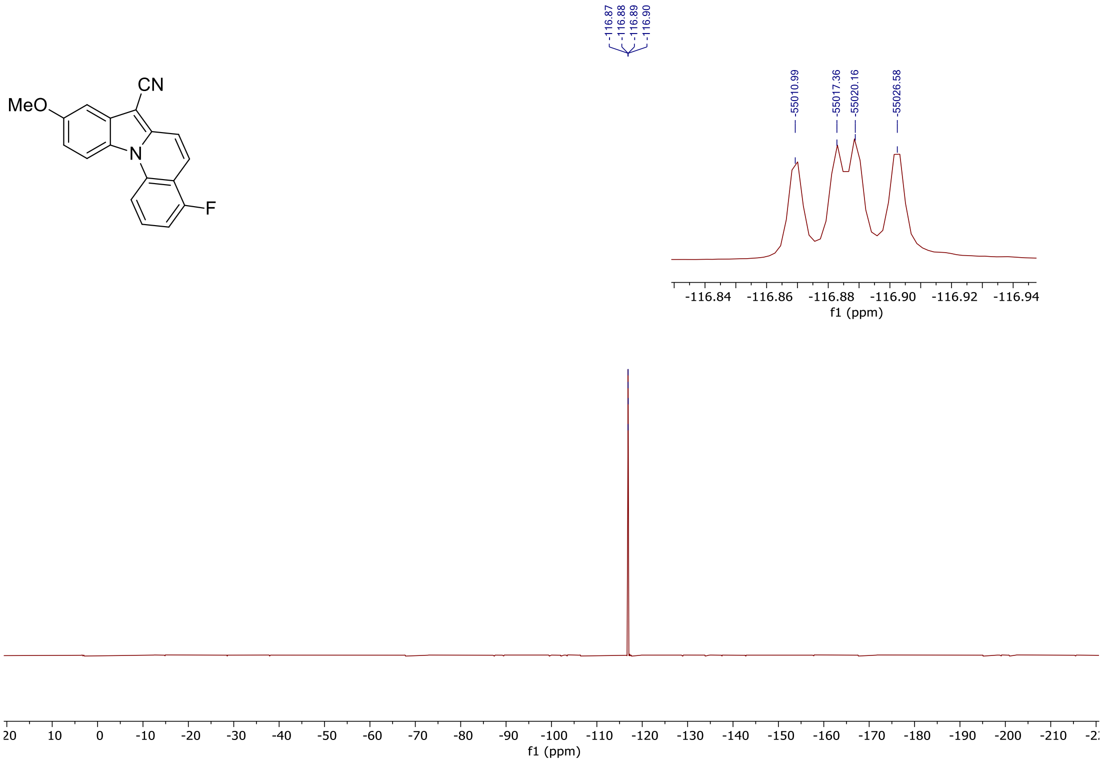

^{19}F NMR spectrum (500 MHz, $CDCl_3$) of compound **5ce** with an internal standard (fluorobenzene)

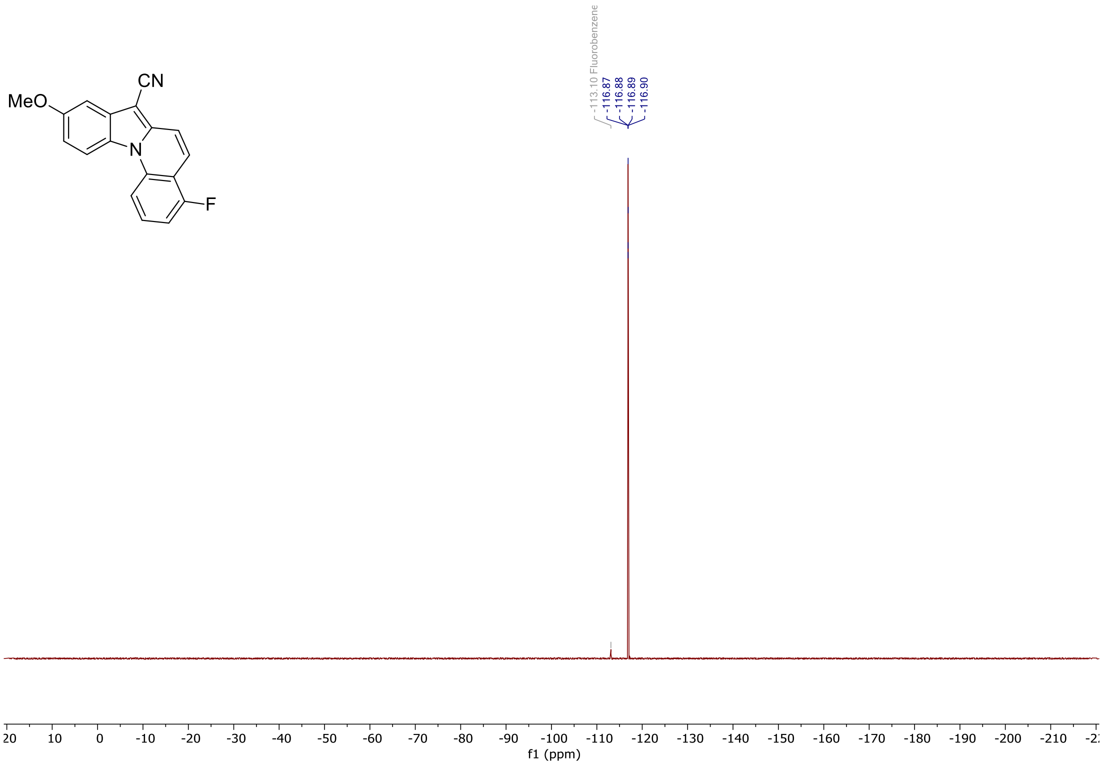

IR spectrum (neat) of compound **5ce**

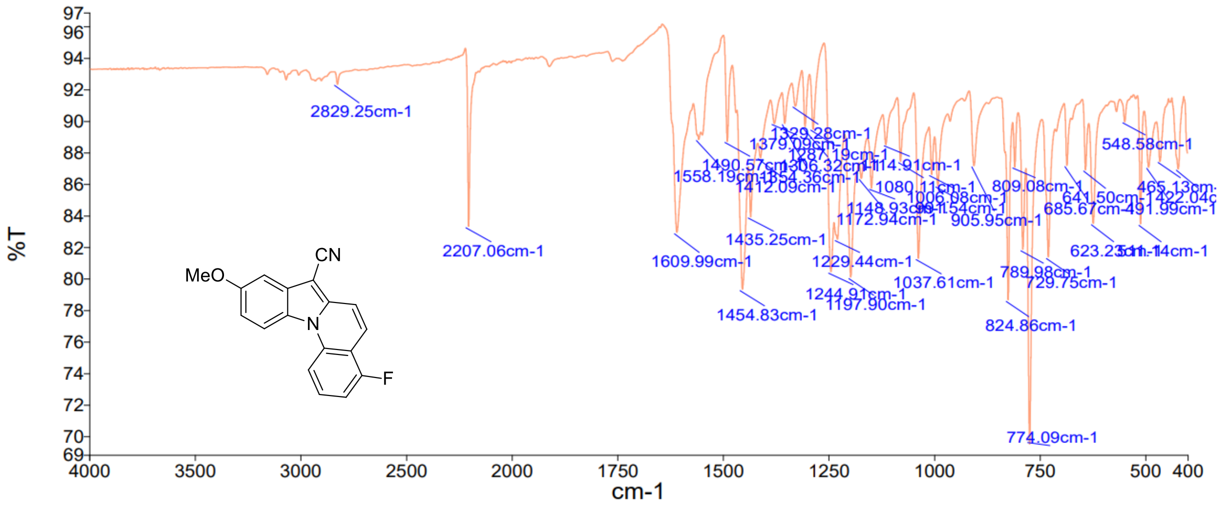

^{1}H NMR spectrum (400 MHz, $CDCl_3$) of compound **5cf**

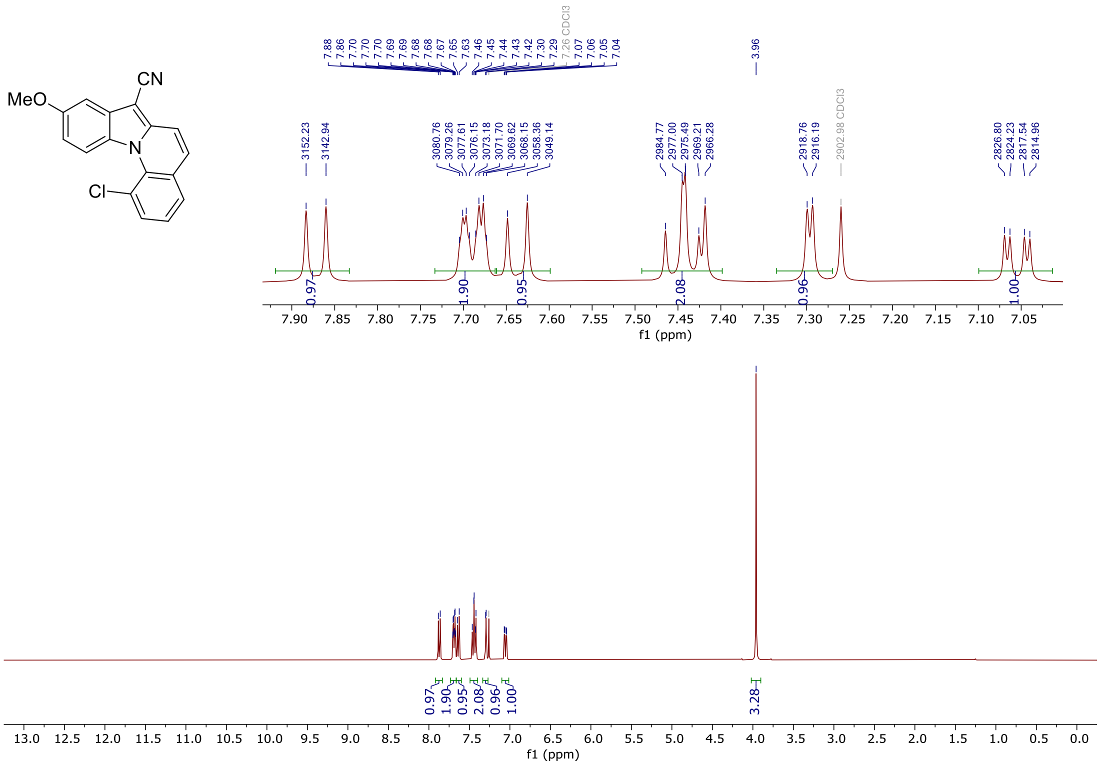

^{13}C{^{1}H} NMR spectrum (100 MHz, $CDCl_3$) of compound **5cf**

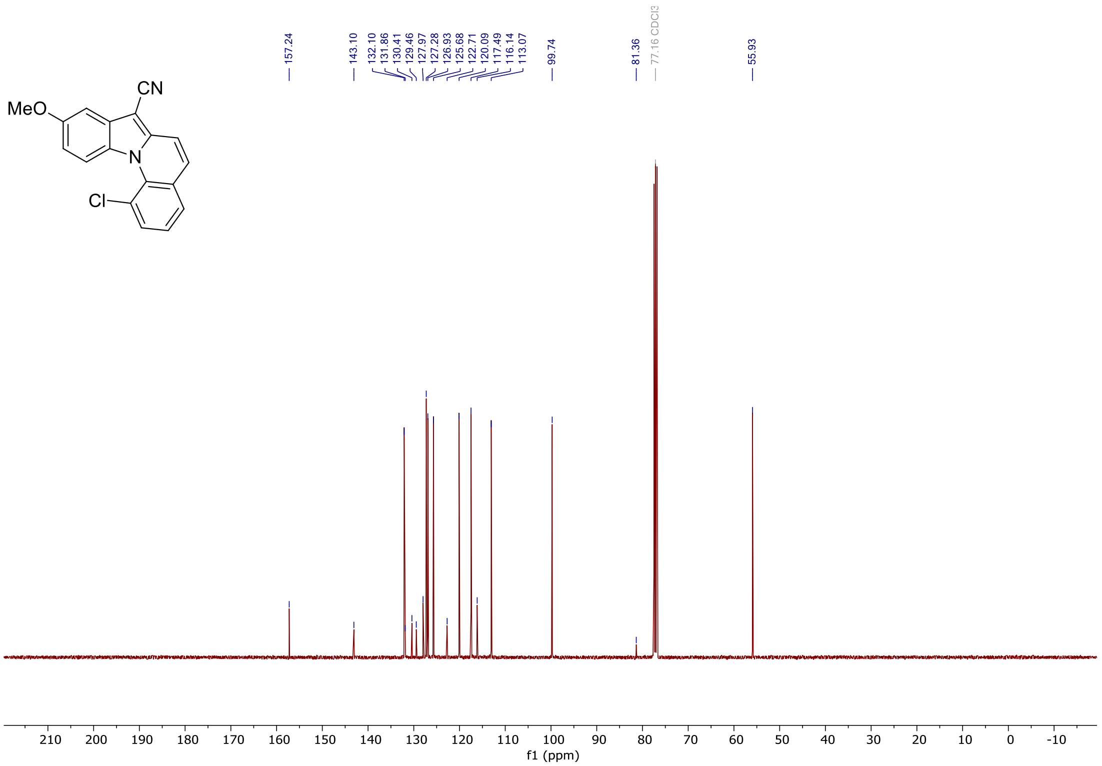

IR spectrum (neat) of compound **5cf**

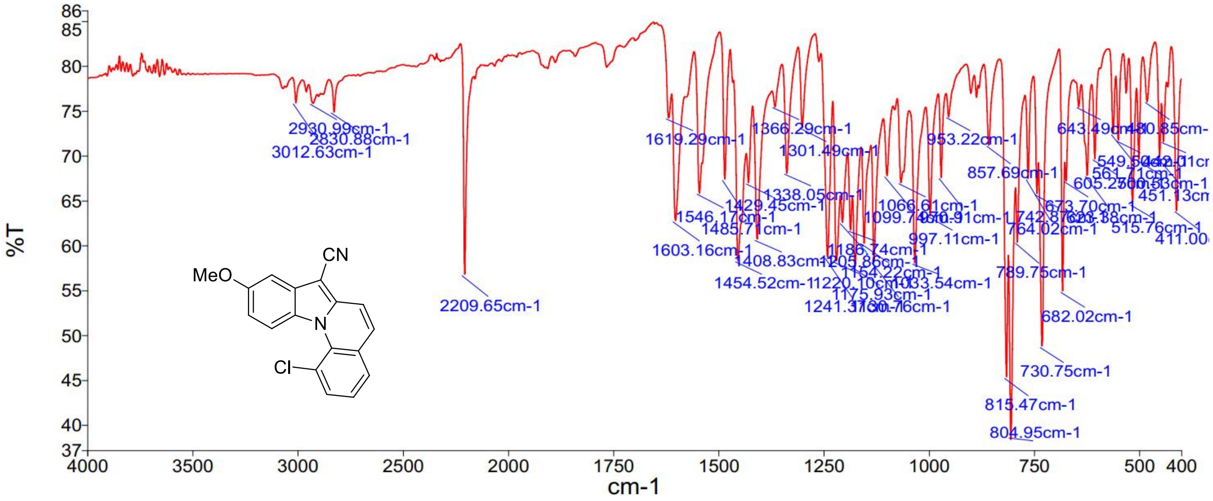

^{1}H NMR spectrum (400 MHz, $CDCl_3$) of compound **5cg**

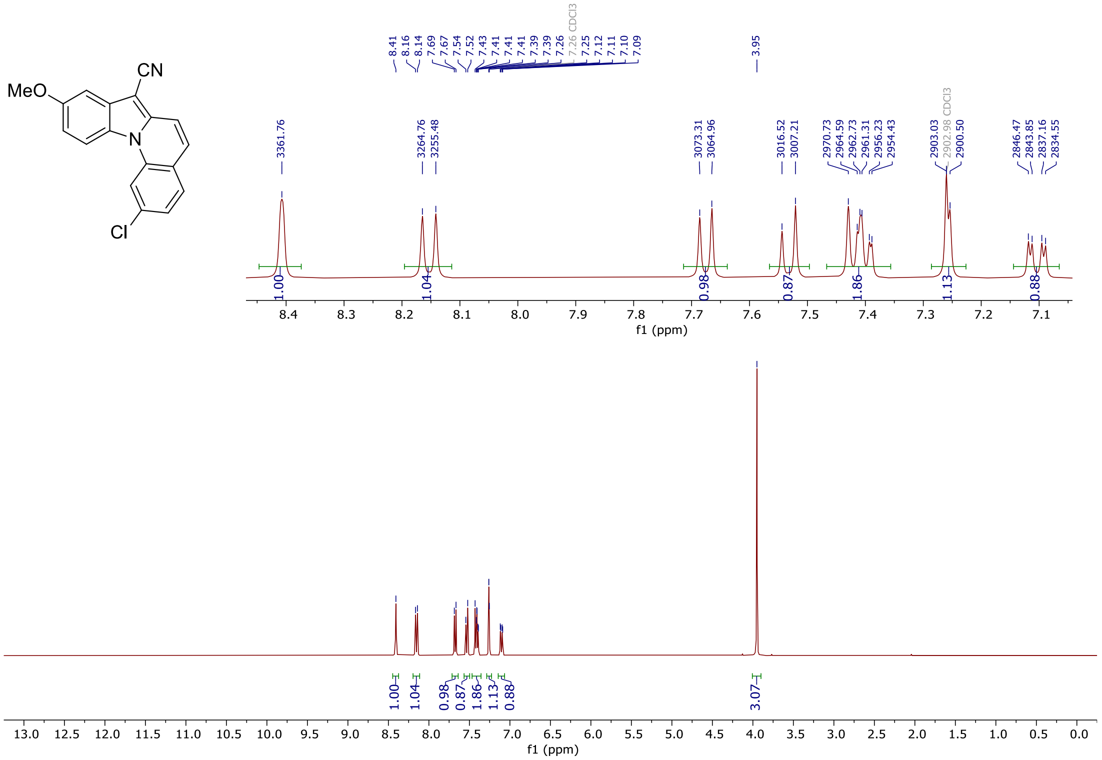

$^{13}C\{^{1}H\}$ NMR spectrum (100 MHz, $CDCl_3$) of compound **5cg**

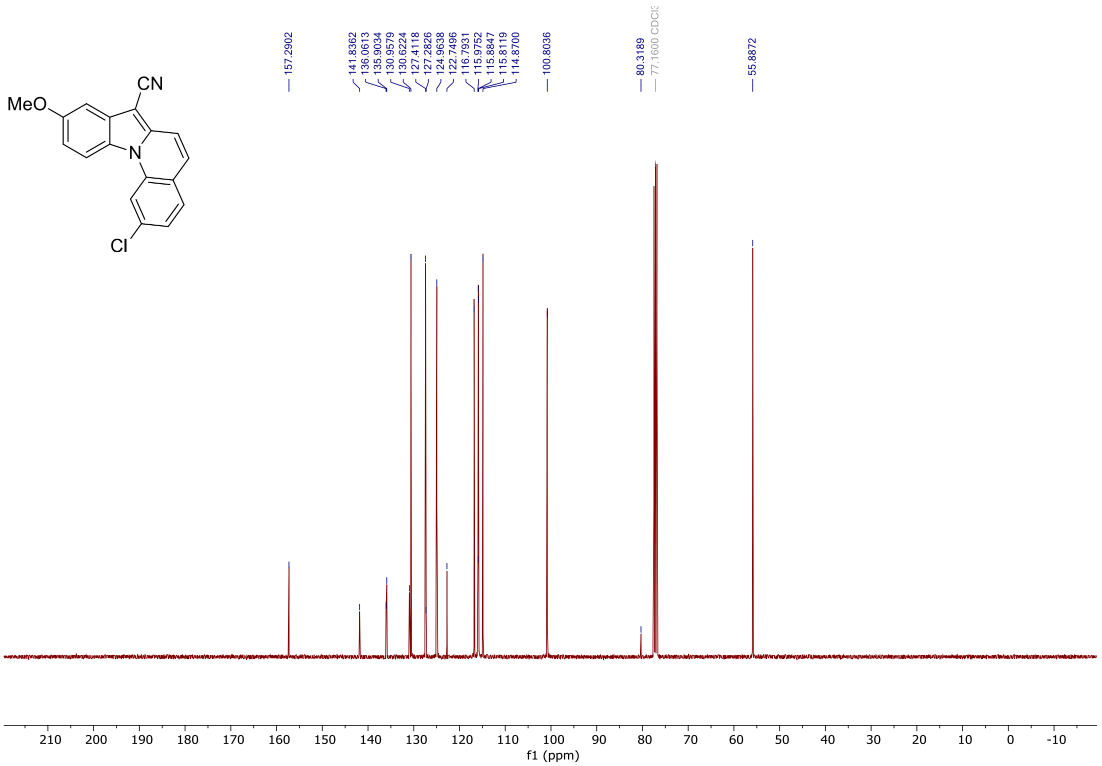

IR spectrum (neat) of compound **5cg**

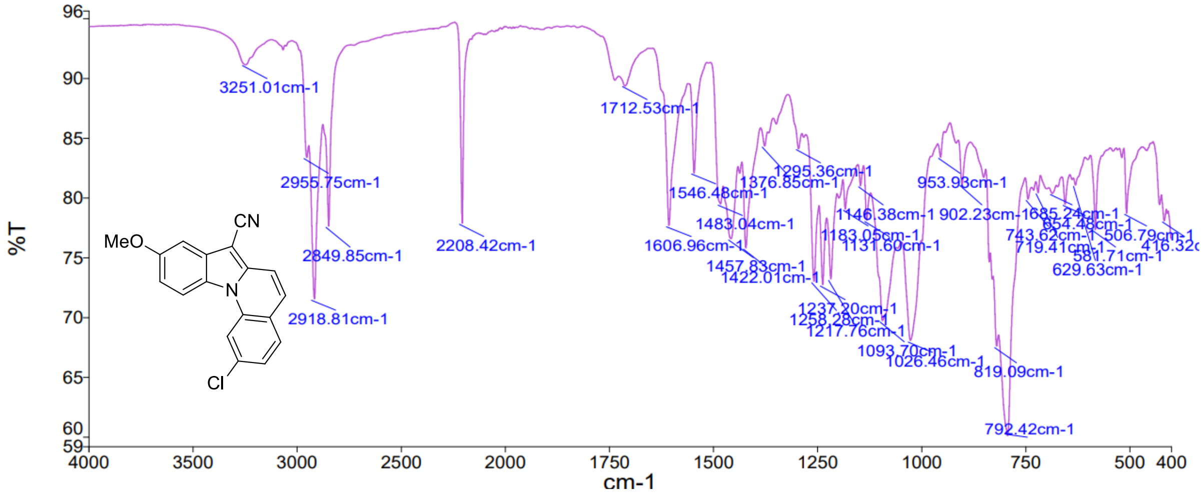

^{1}H NMR spectrum (400 MHz, $CDCl_3$) of compound **5ch**

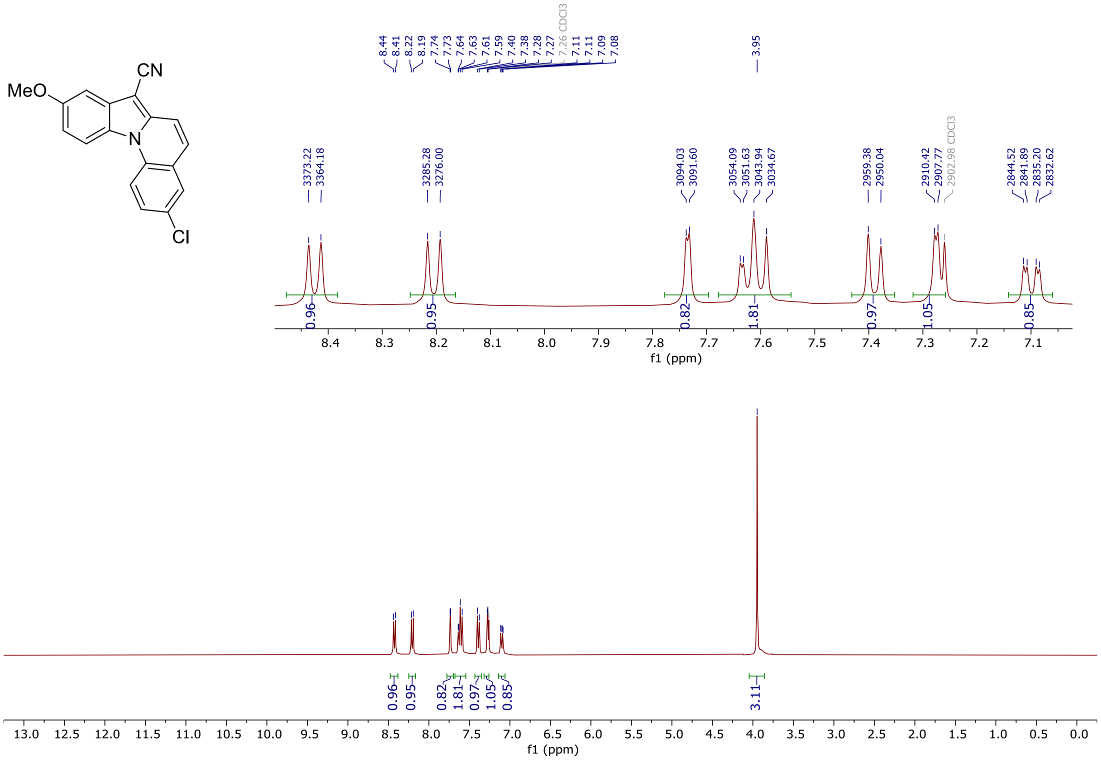

^{13}C{^{1}H} NMR spectrum (100 MHz, $CDCl_3$) of compound **5ch**

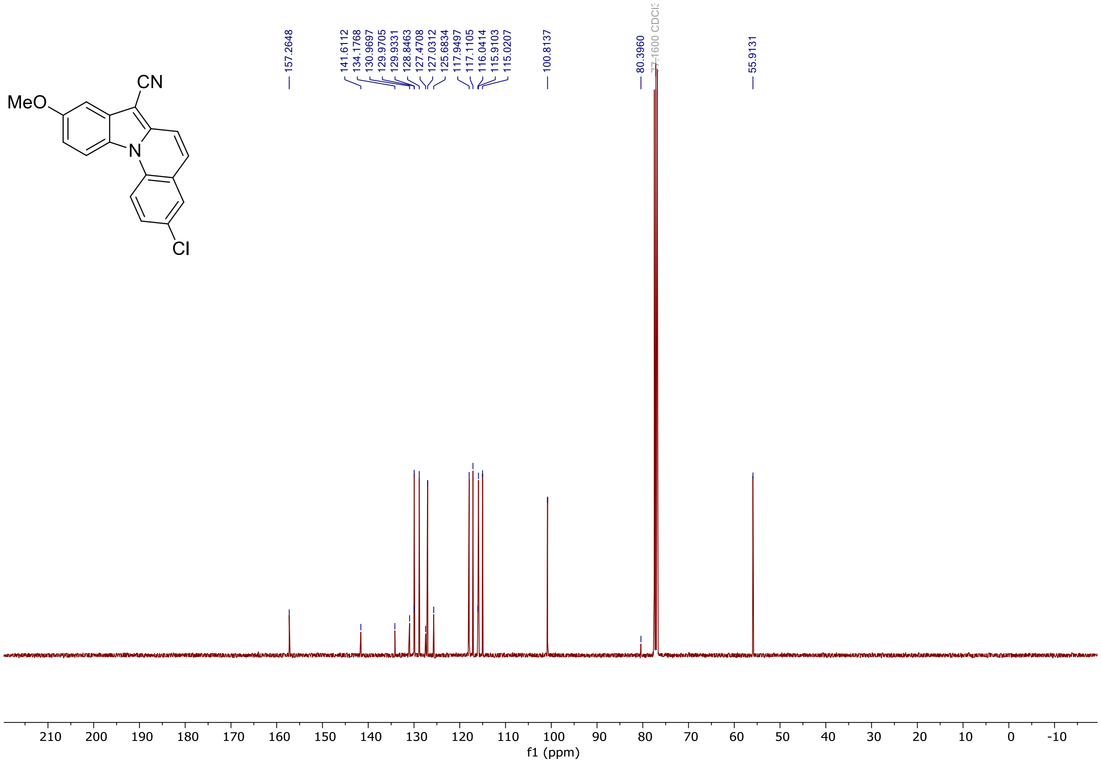

IR spectrum (neat) of compound **5ch**

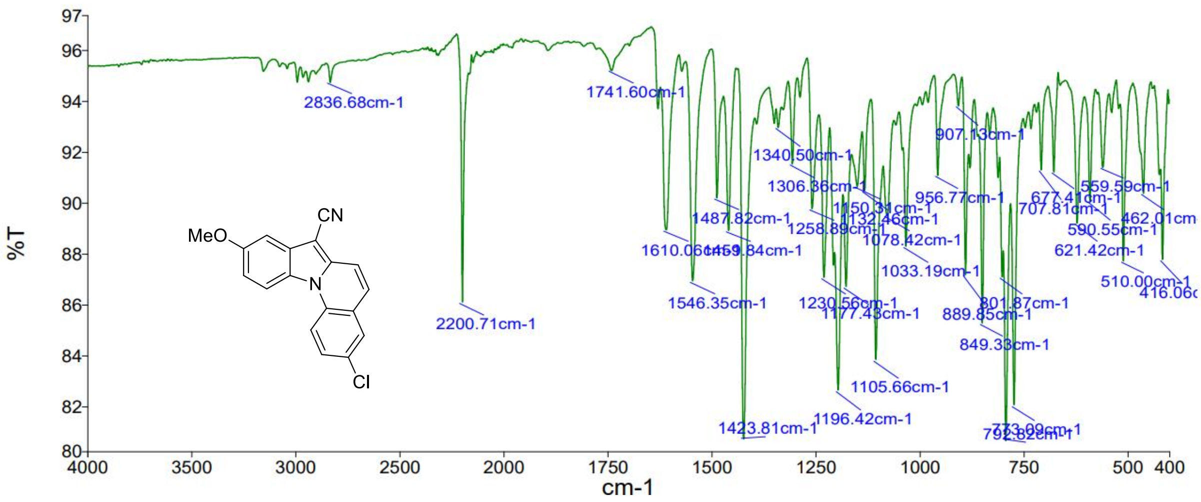

^{1}H NMR spectrum (400 MHz, $CDCl_3$) of compound **5ci**

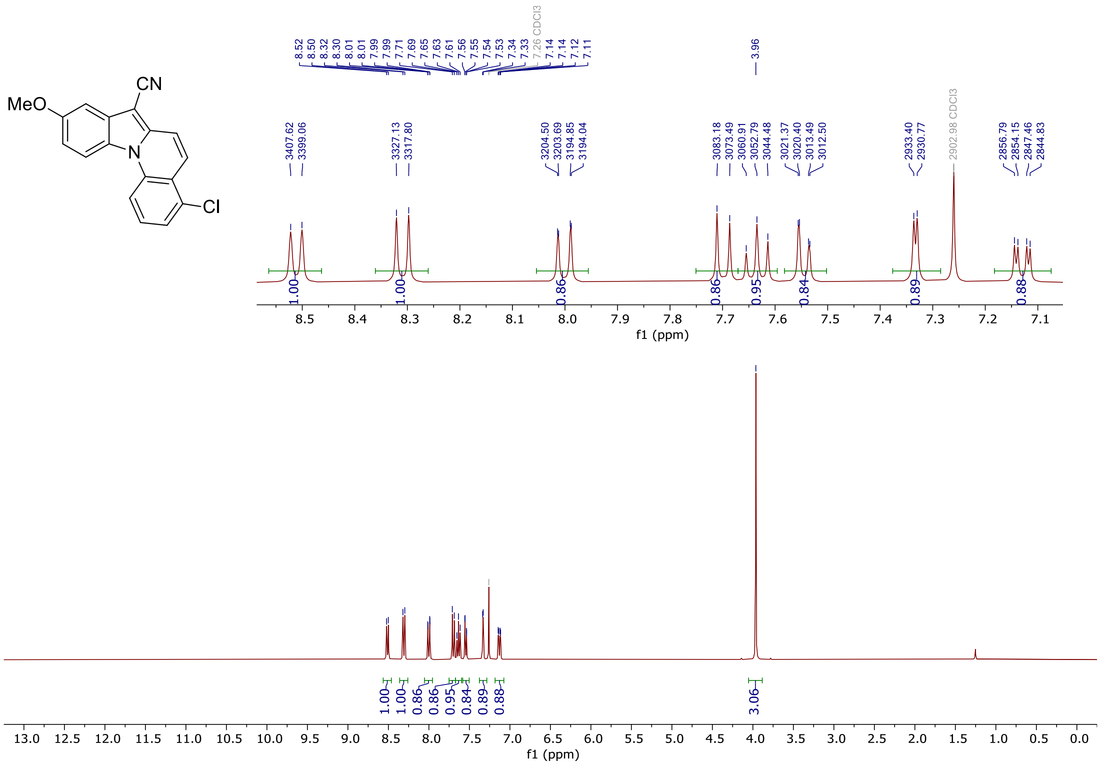

$^{13}C\{^{1}H\}$ NMR spectrum (100 MHz, $CDCl_3$) of compound **5ci**

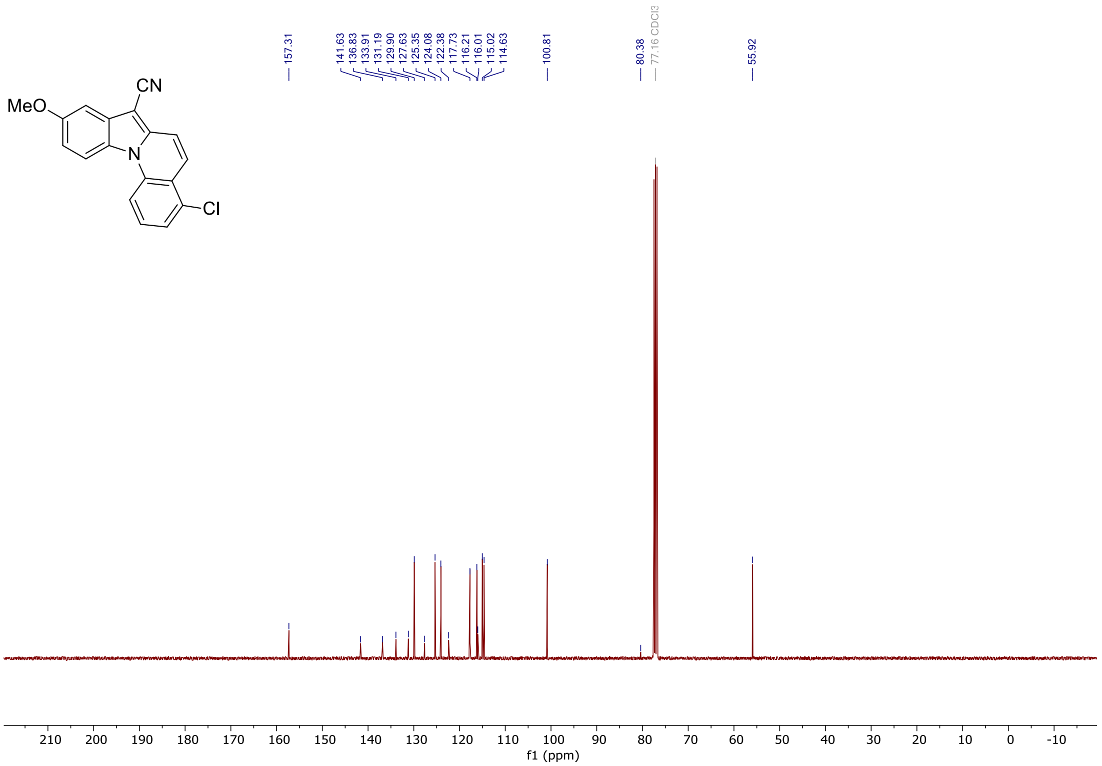

IR spectrum (neat) of compound **5ci**

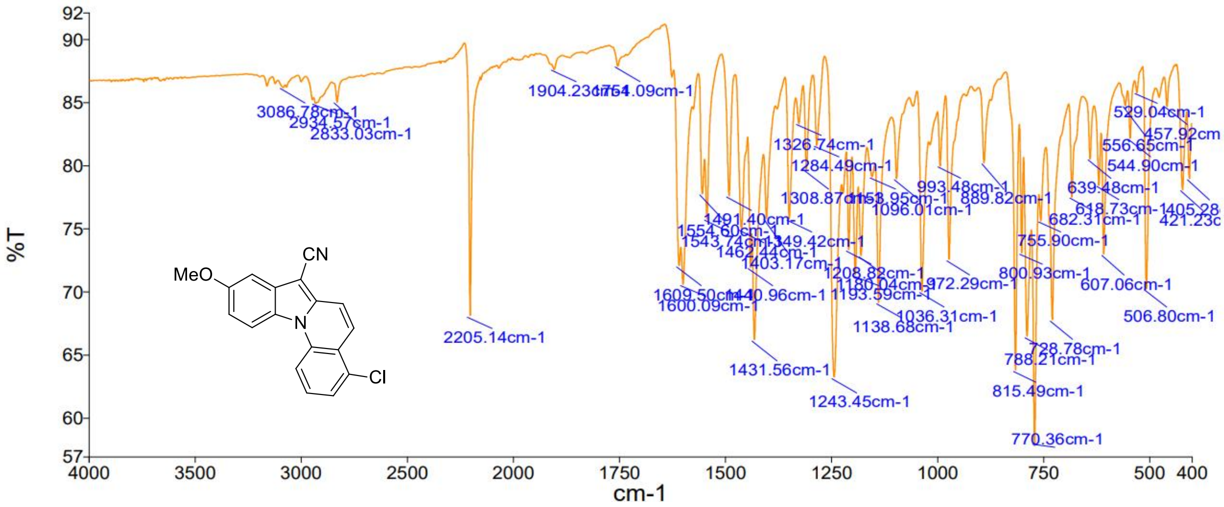

^{1}H NMR spectrum (400 MHz, $CDCl_3$) of compound **5ck**

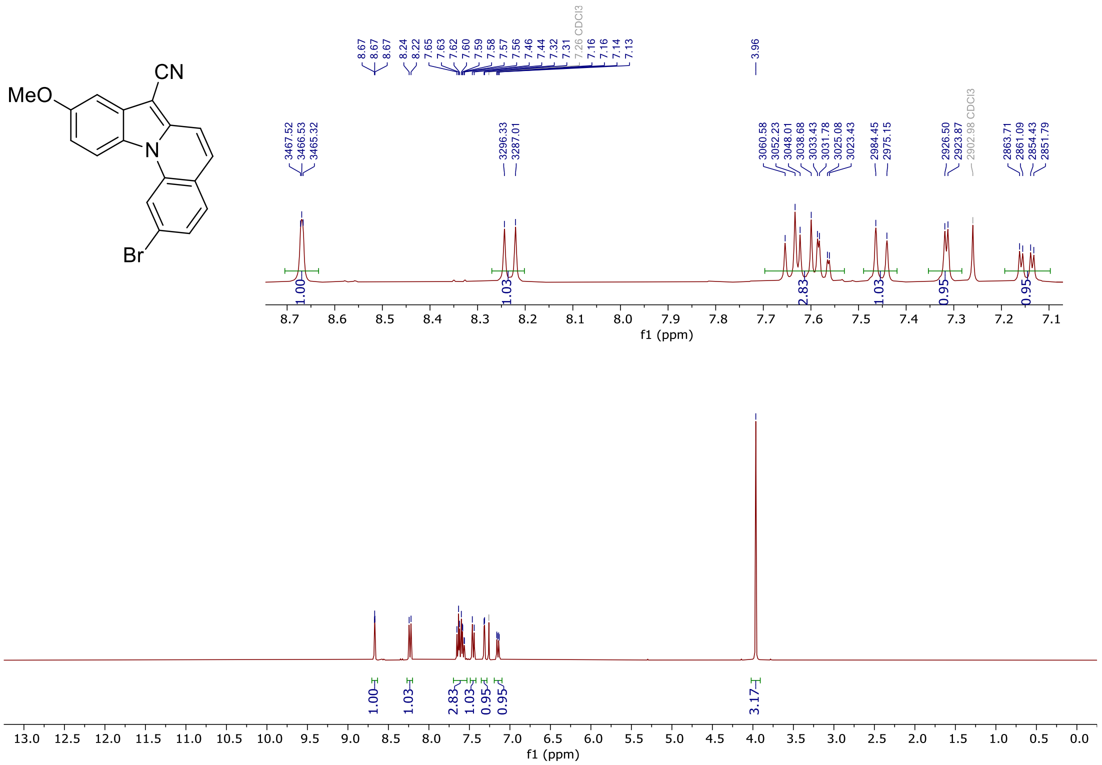

$^{13}C\{^{1}H\}$ NMR spectrum (100 MHz, $CDCl_3$) of compound **5ck**

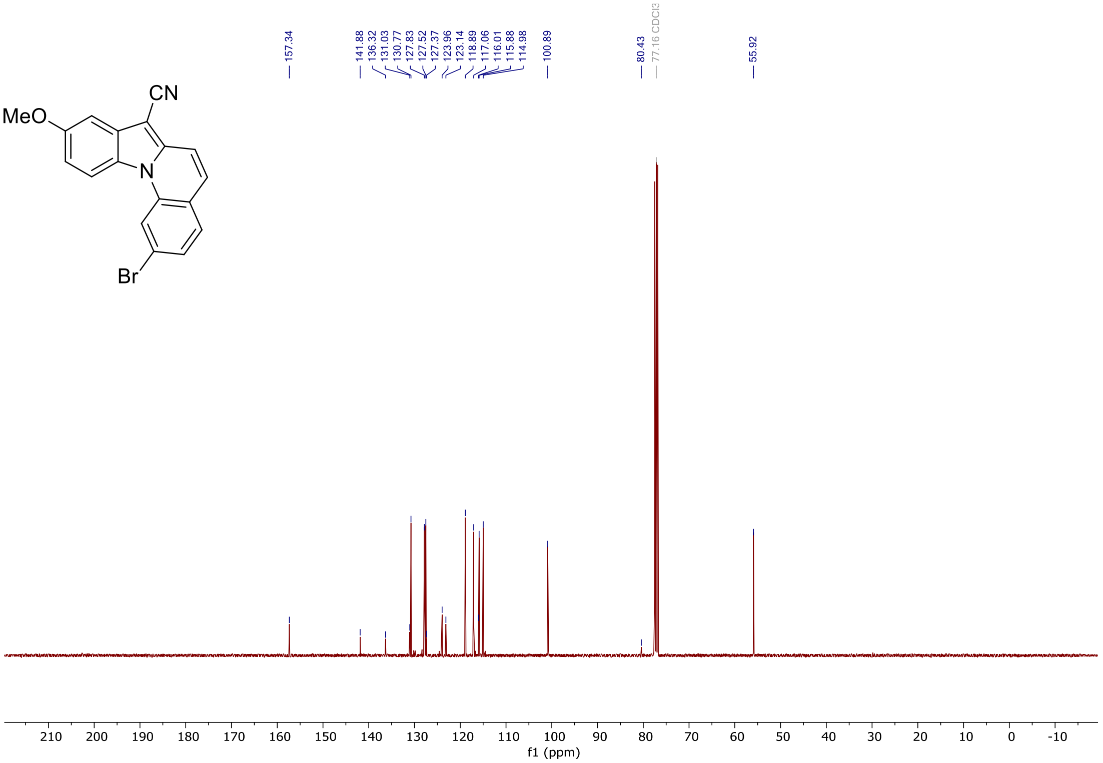

IR spectrum (neat) of compound **5ck**

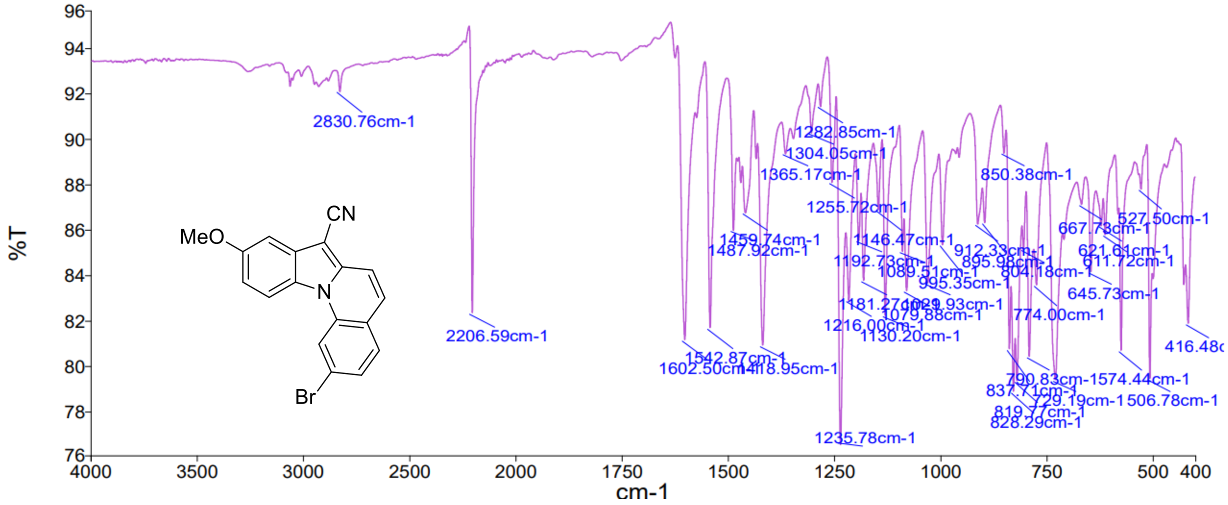

^{1}H NMR spectrum (400 MHz, $CDCl_3$) of compound **5cm**

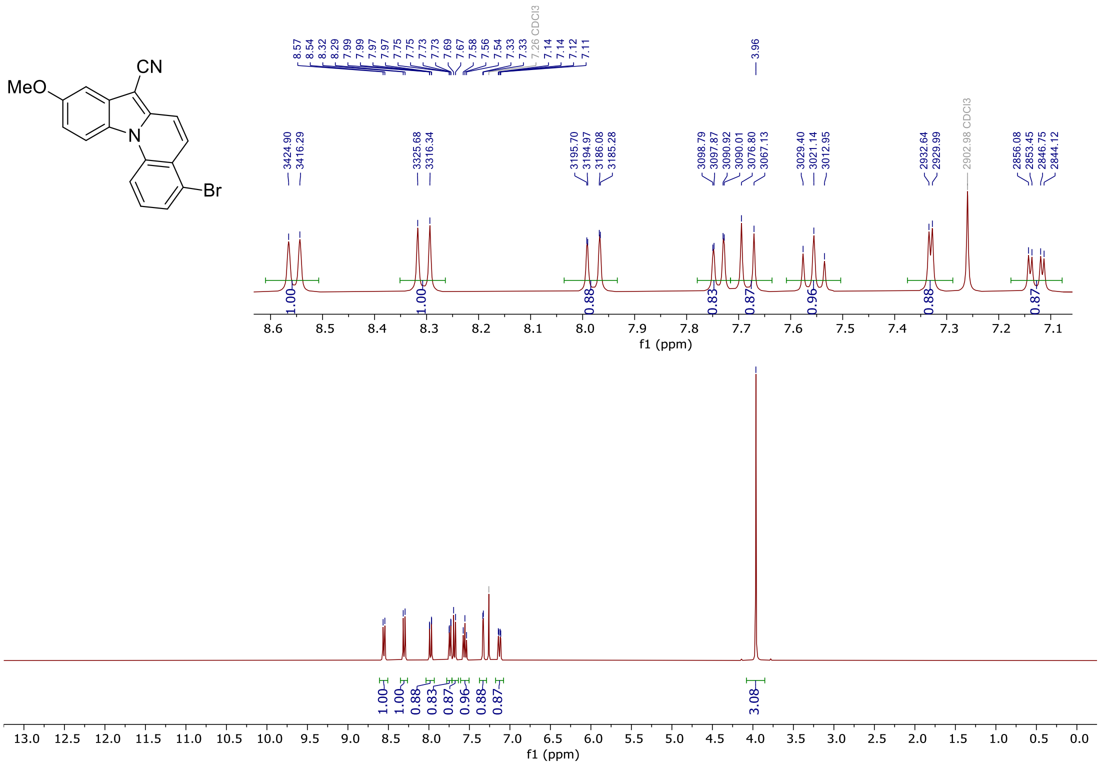

$^{13}C\{^{1}H\}$ NMR spectrum (100 MHz, $CDCl_3$) of compound **5cm**

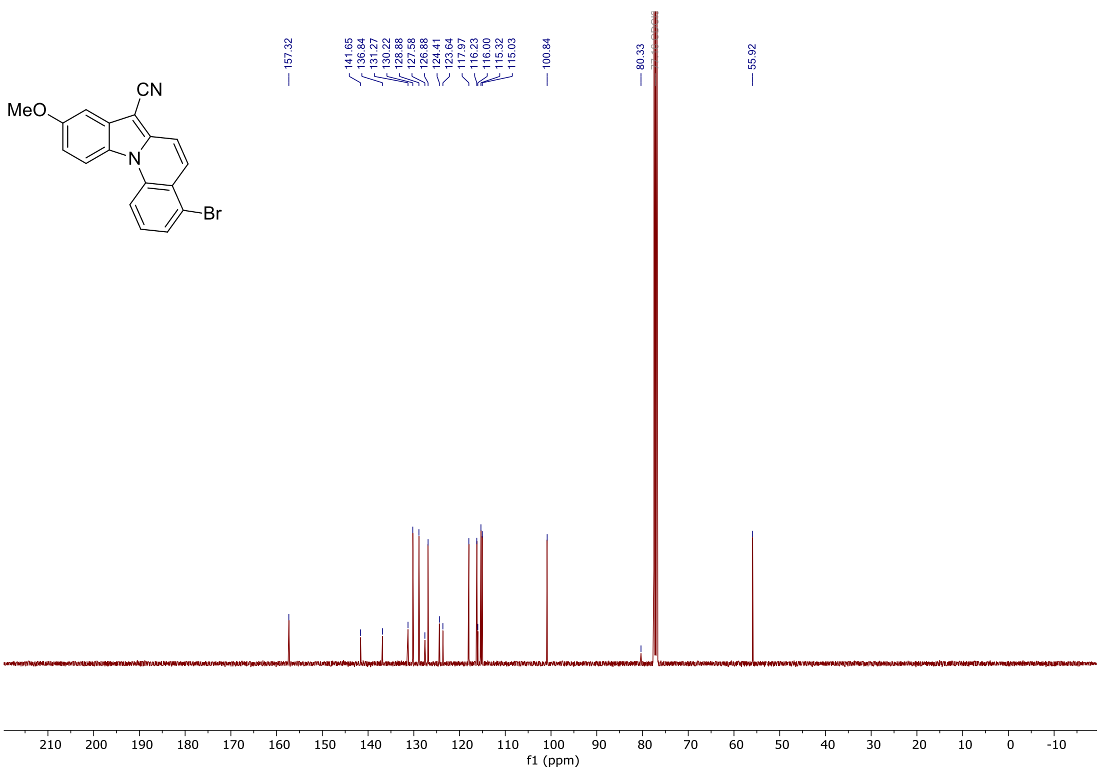

IR spectrum (neat) of compound **5cm**

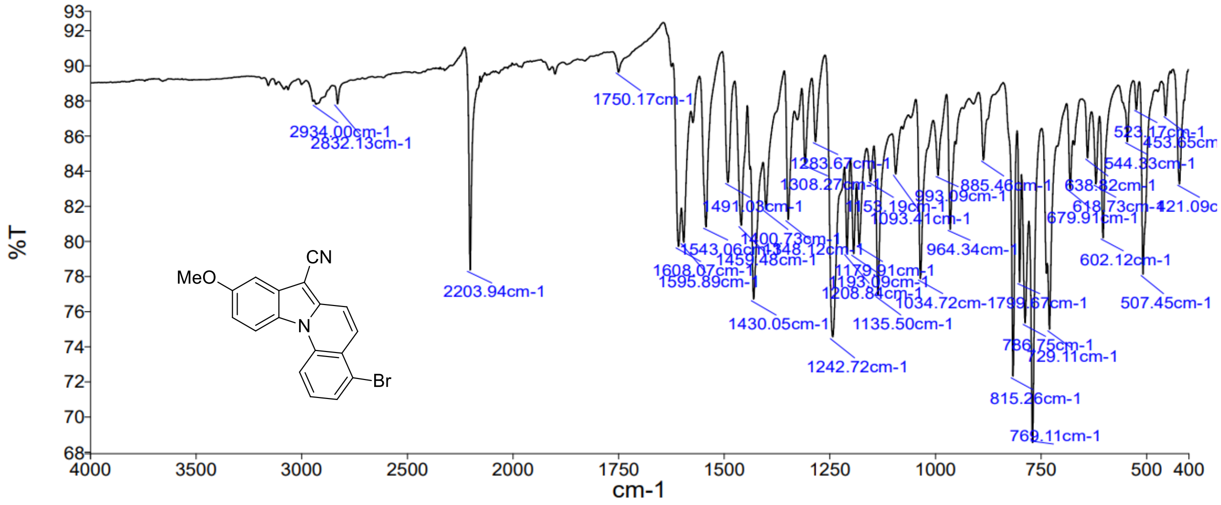

^{1}H NMR spectrum (400 MHz, $CDCl_3$) of compound **5co**

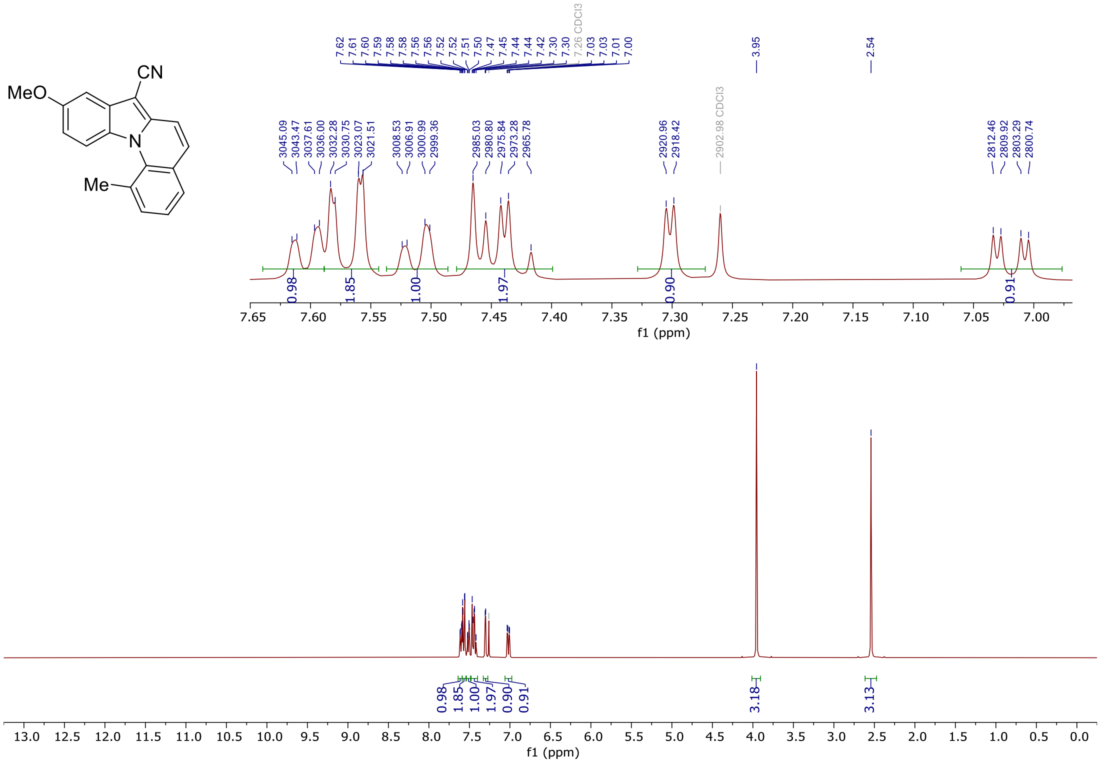

^{13}C{^{1}H} NMR spectrum (100 MHz, $CDCl_3$) of compound **5co**

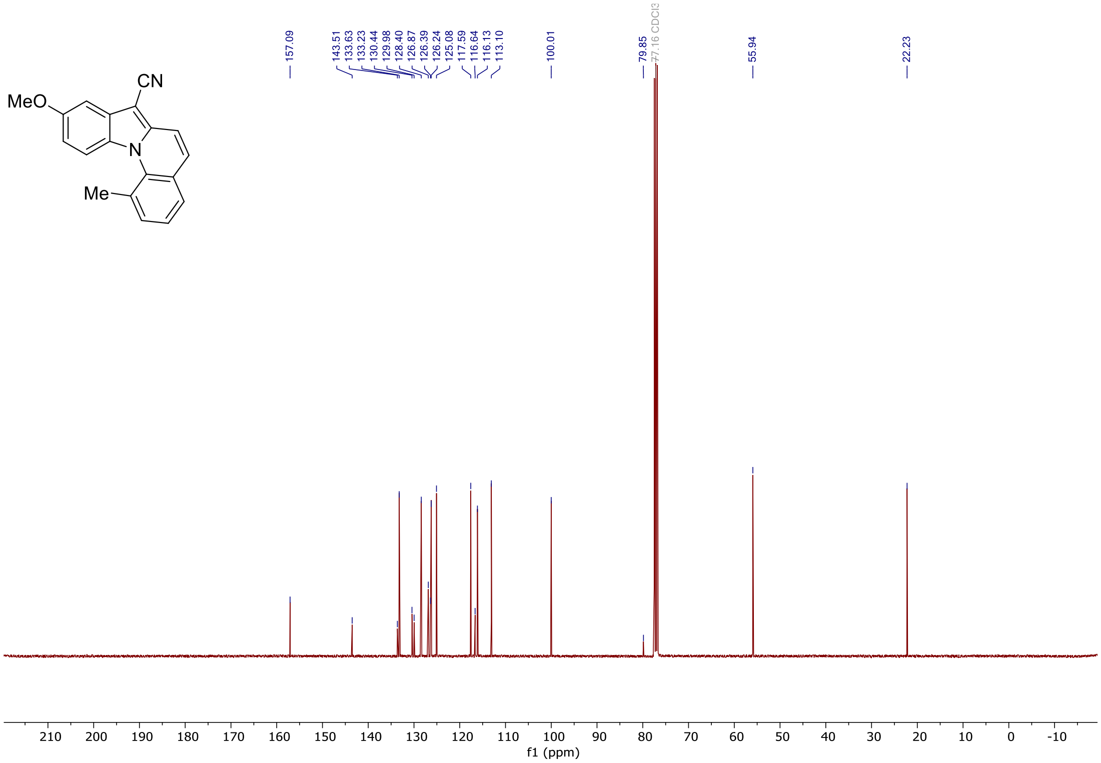

IR spectrum (neat) of compound **5co**

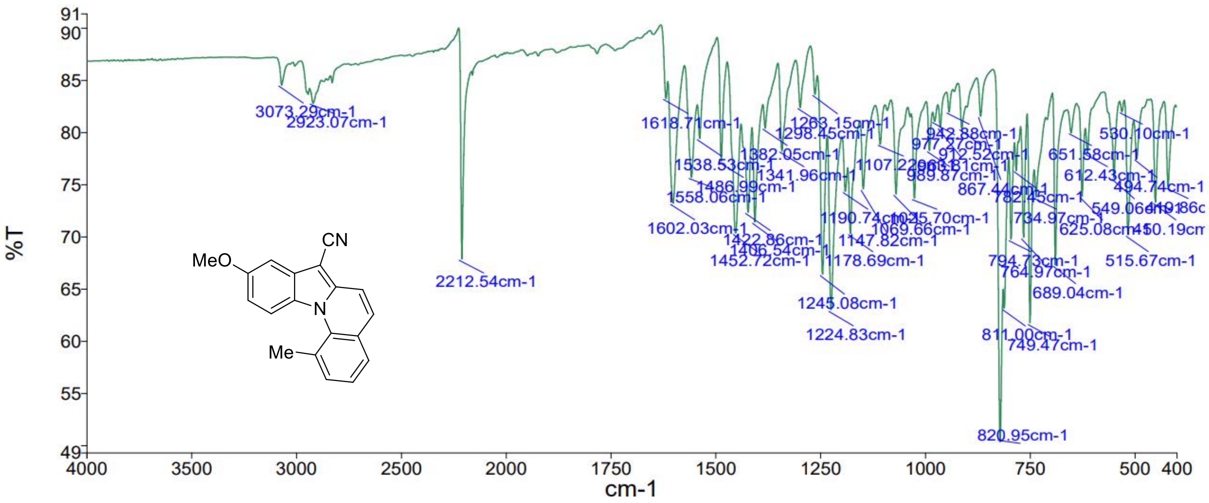

^{1}H NMR spectrum (400 MHz, $CDCl_3$) of compound **5cq**

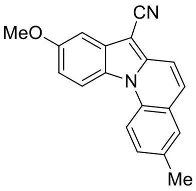

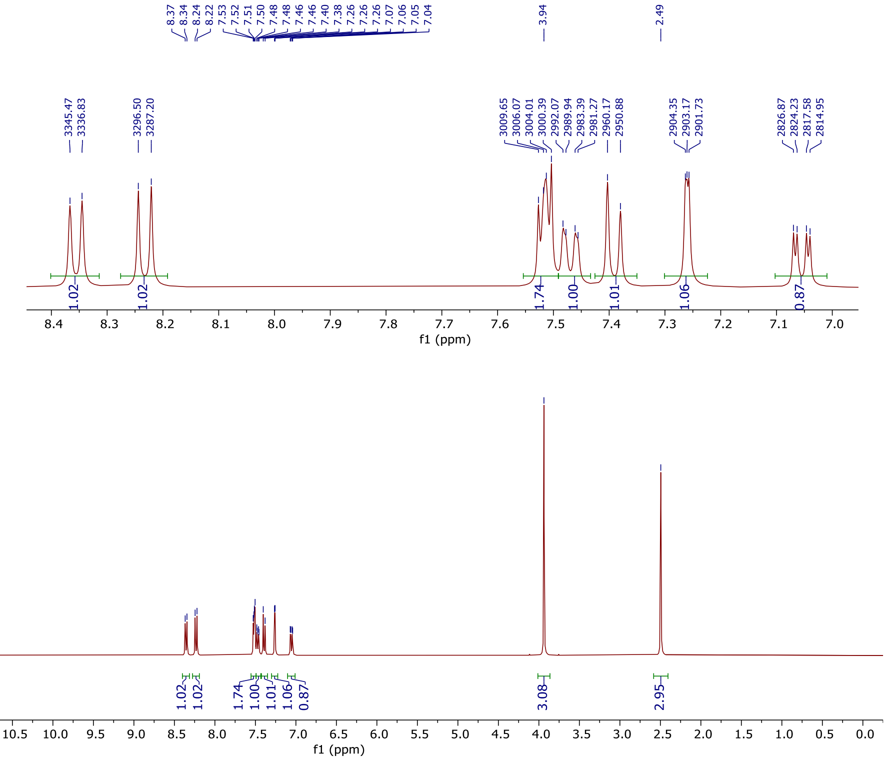

$^{13}C\{^{1}H\}$ NMR spectrum (100 MHz, $CDCl_3$) of compound **5cq**

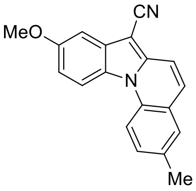

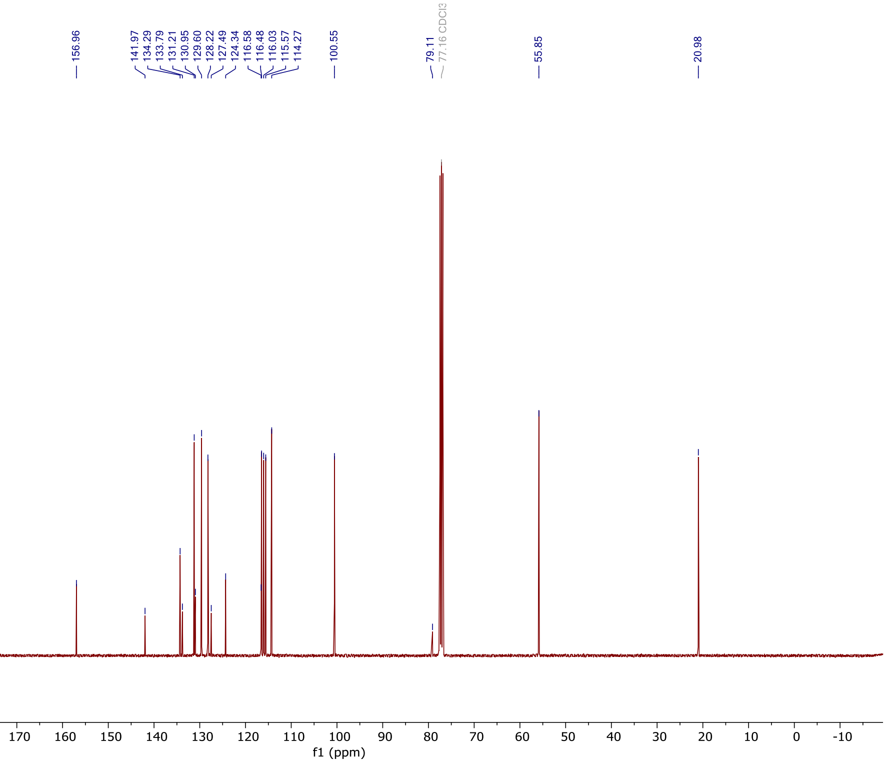

IR spectrum (neat) of compound **5cq**

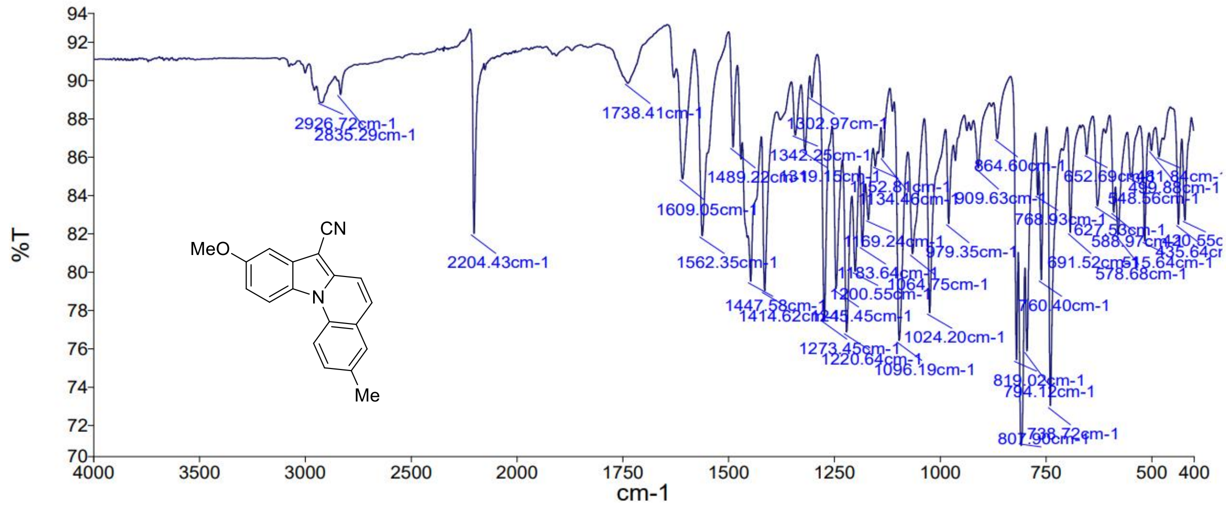

^{1}H NMR spectrum (400 MHz, $CDCl_3$) of compound **5cr**

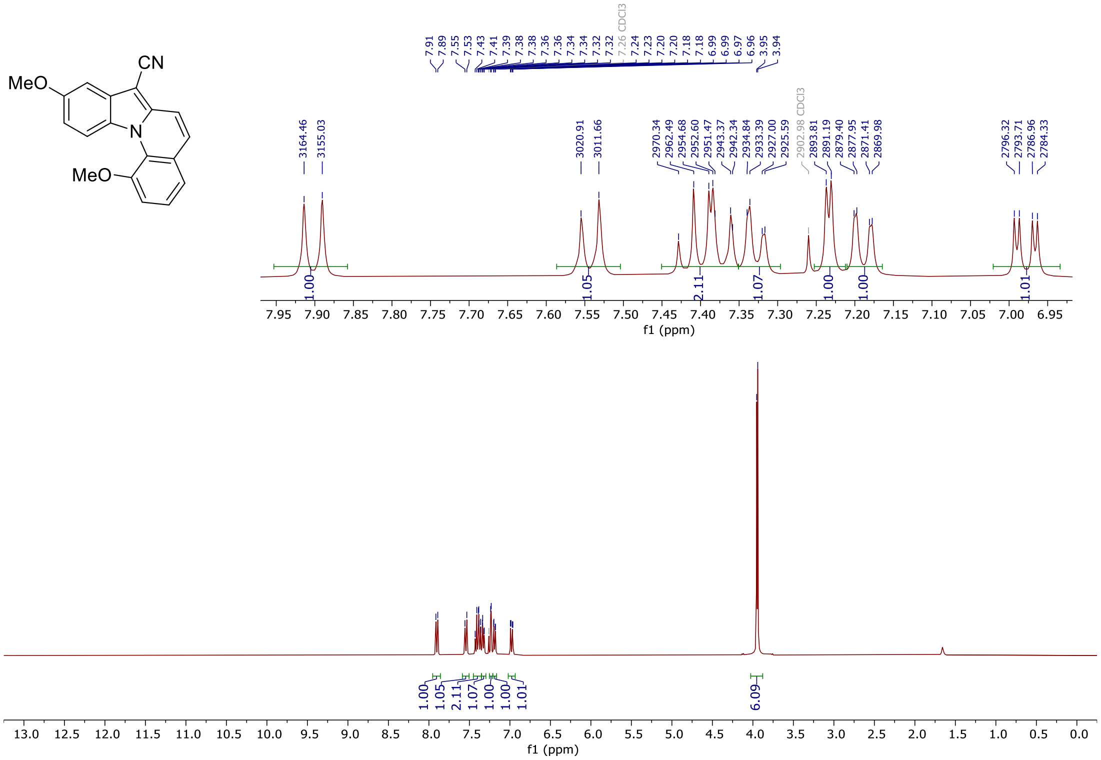

^{13}C{^{1}H} NMR spectrum (100 MHz, $CDCl_3$) of compound **5cr**

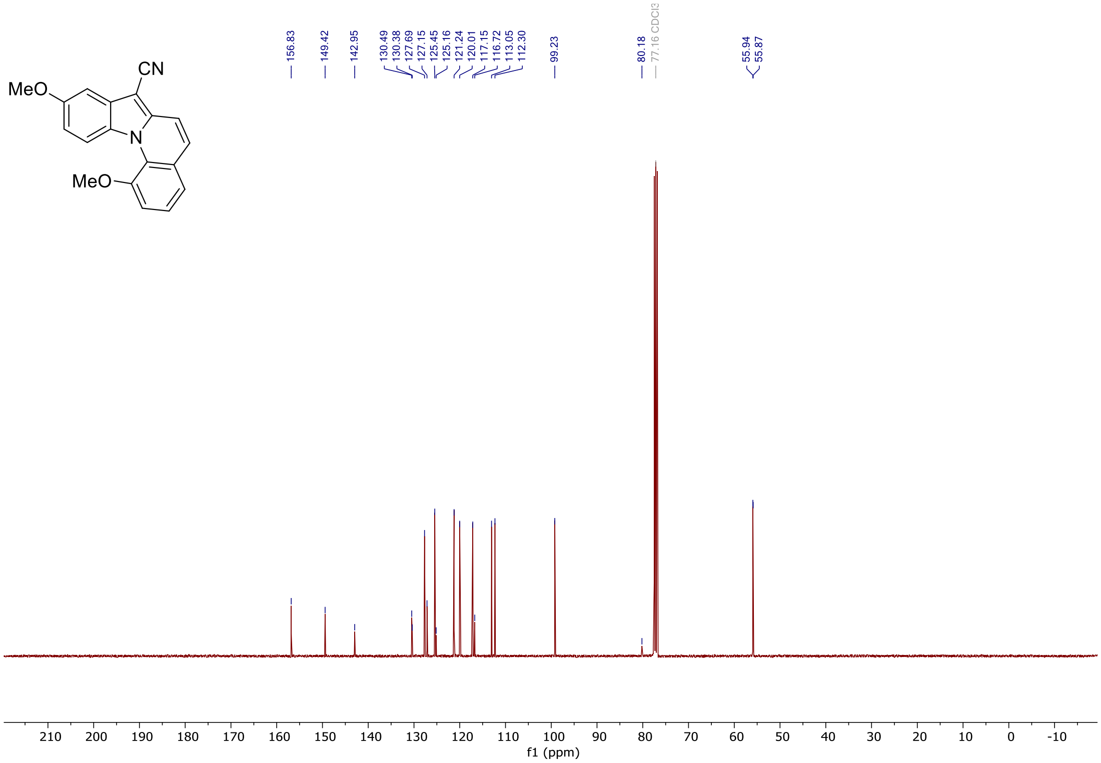

IR spectrum (neat) of compound **5cr**

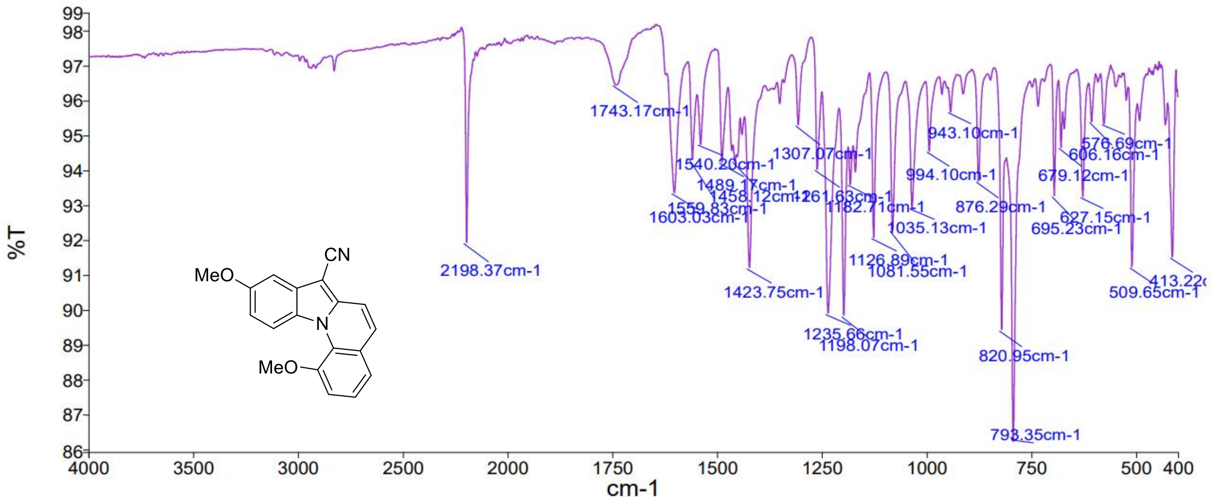

^{1}H NMR spectrum (400 MHz, $CDCl_3$) of compound **5cs**

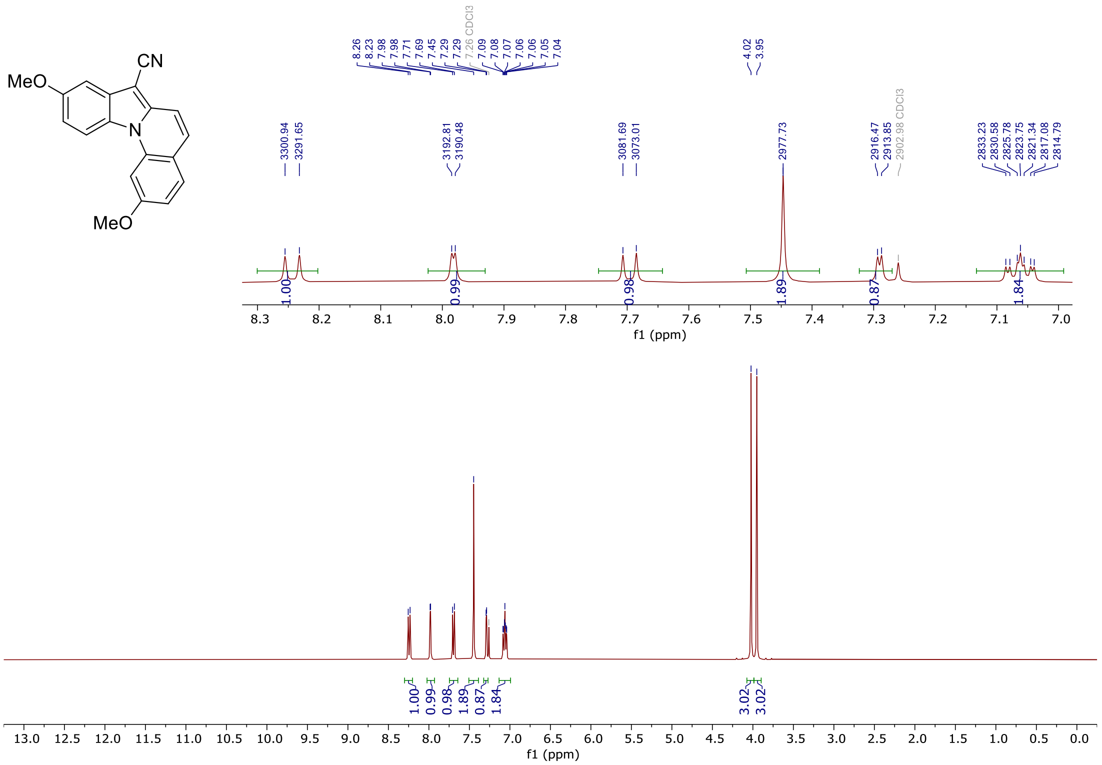

^{13}C{^{1}H} NMR spectrum (100 MHz, $CDCl_3$) of compound **5cs**

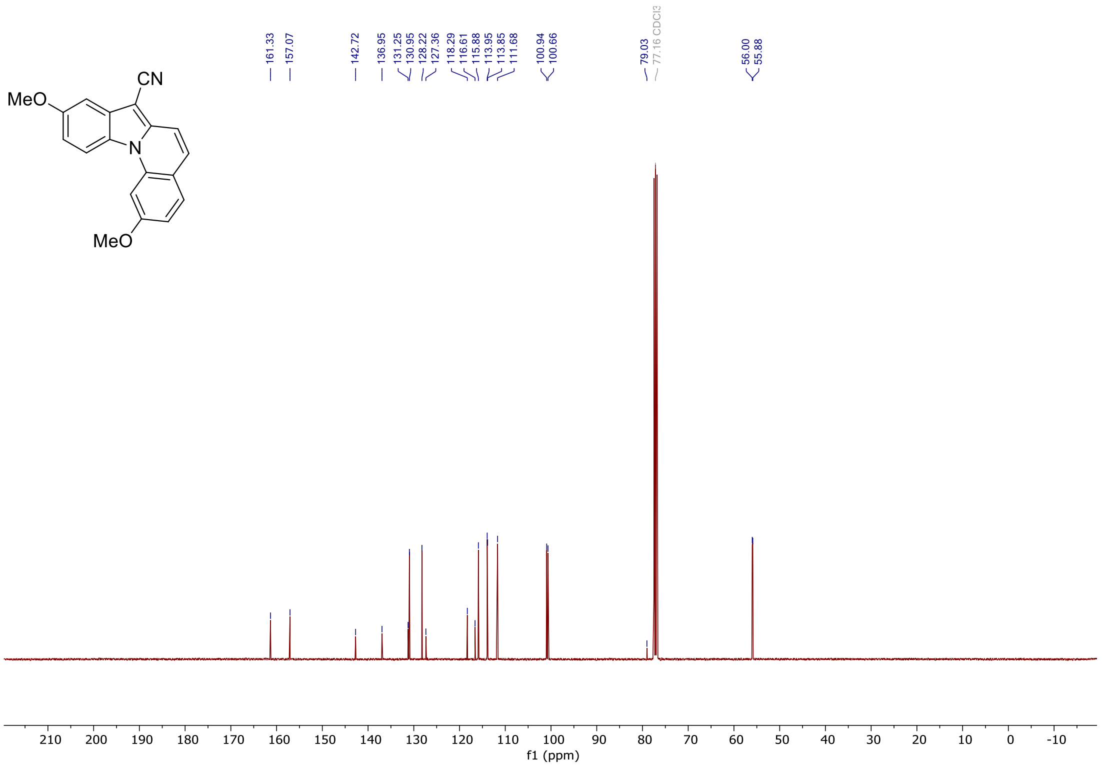

IR spectrum (neat) of compound **5cs**

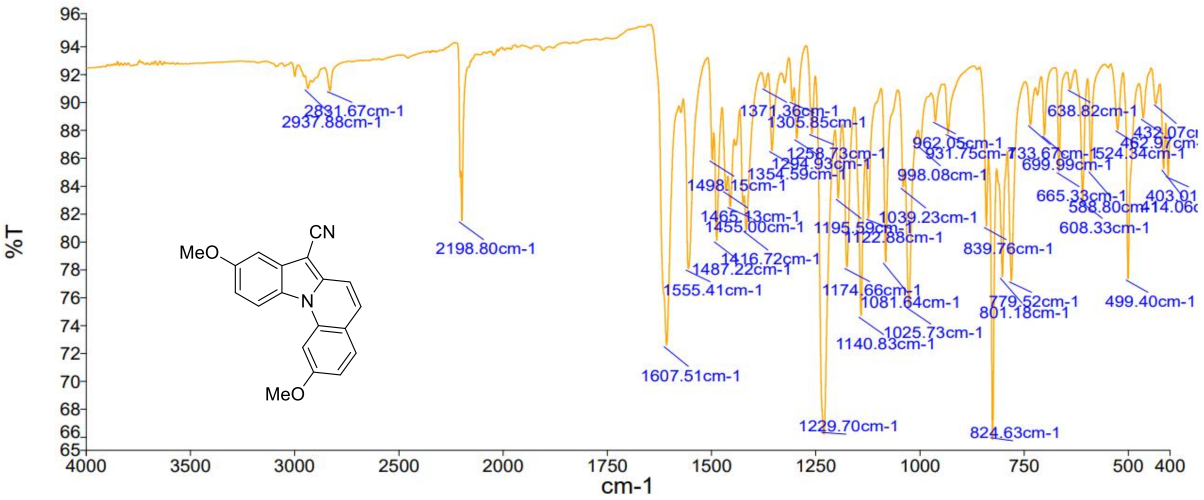

^{1}H NMR spectrum (400 MHz, $CDCl_3$) of compound **5ct**

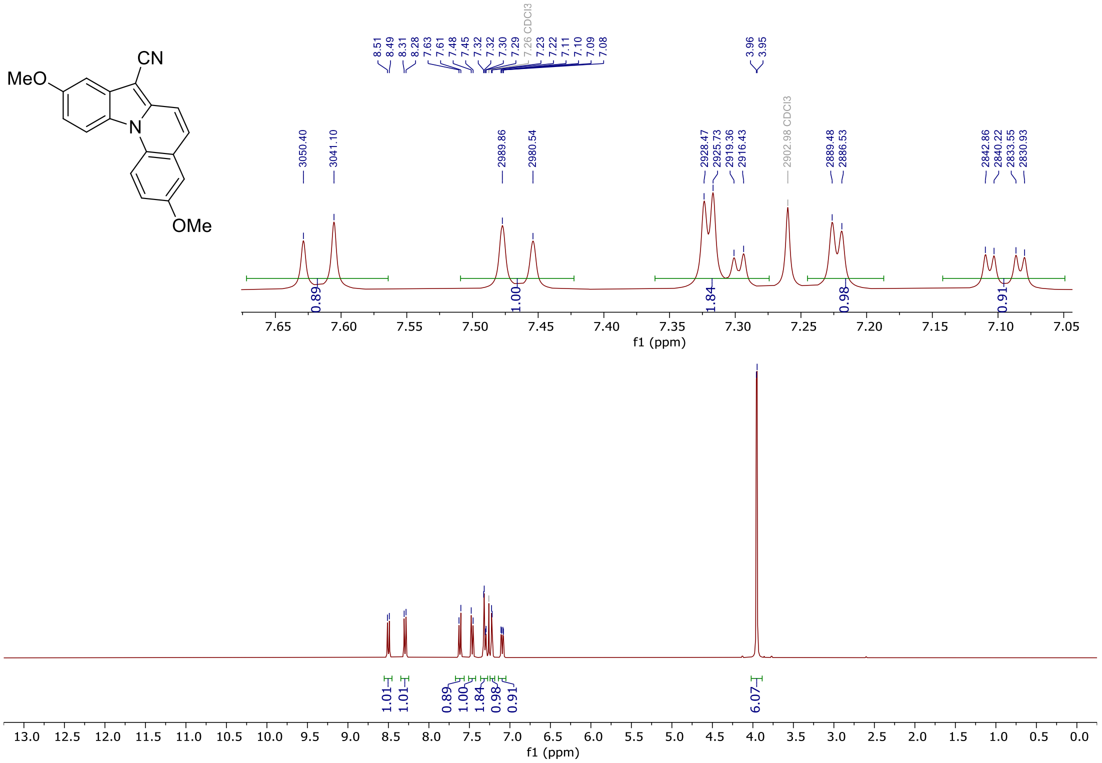

$^{13}C\{^1H\}$ NMR spectrum (100 MHz, $CDCl_3$) of compound **5ct**

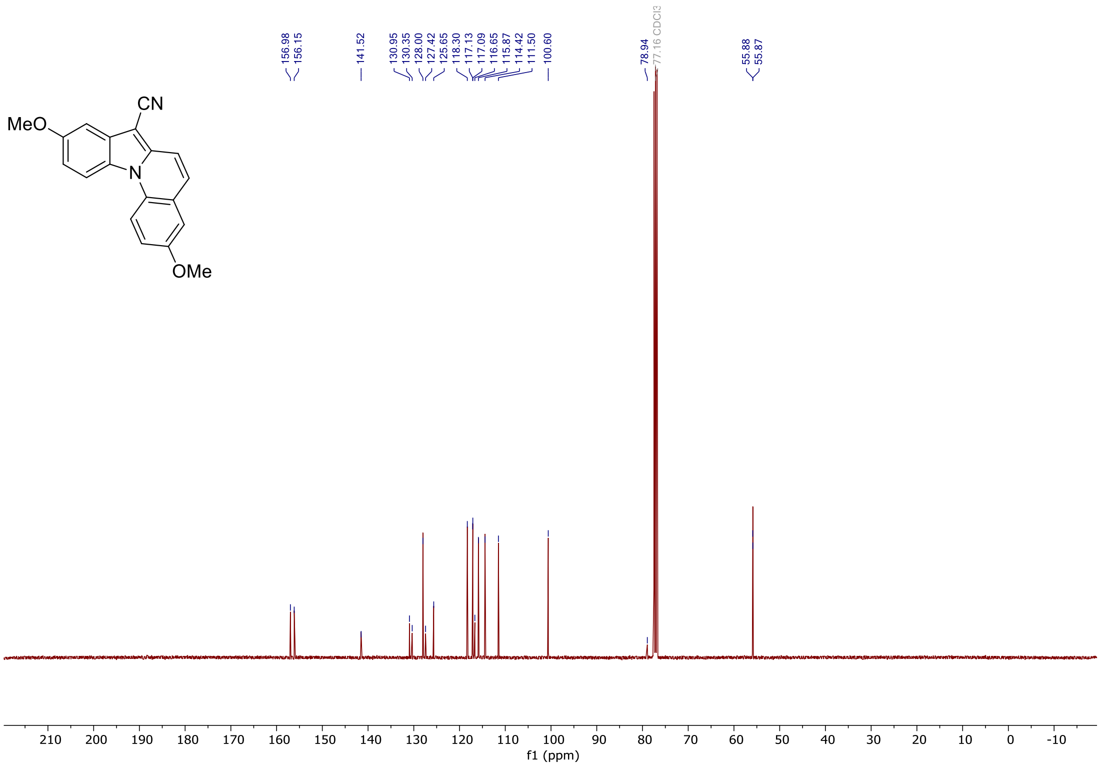

IR spectrum (neat) of compound **5ct**

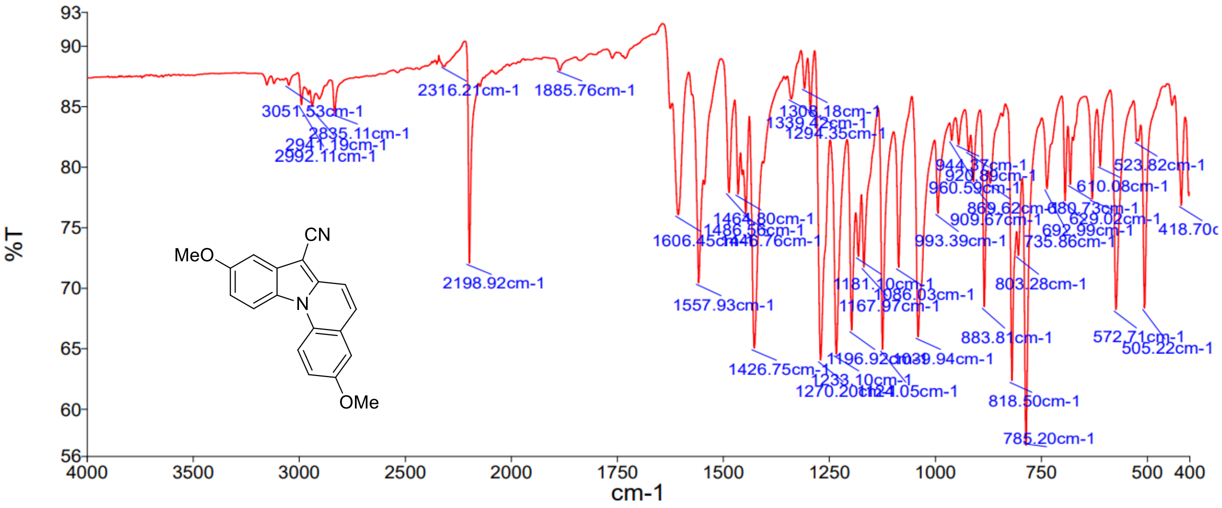

^{1}H NMR spectrum (400 MHz, $CDCl_3$) of compound **5cu**

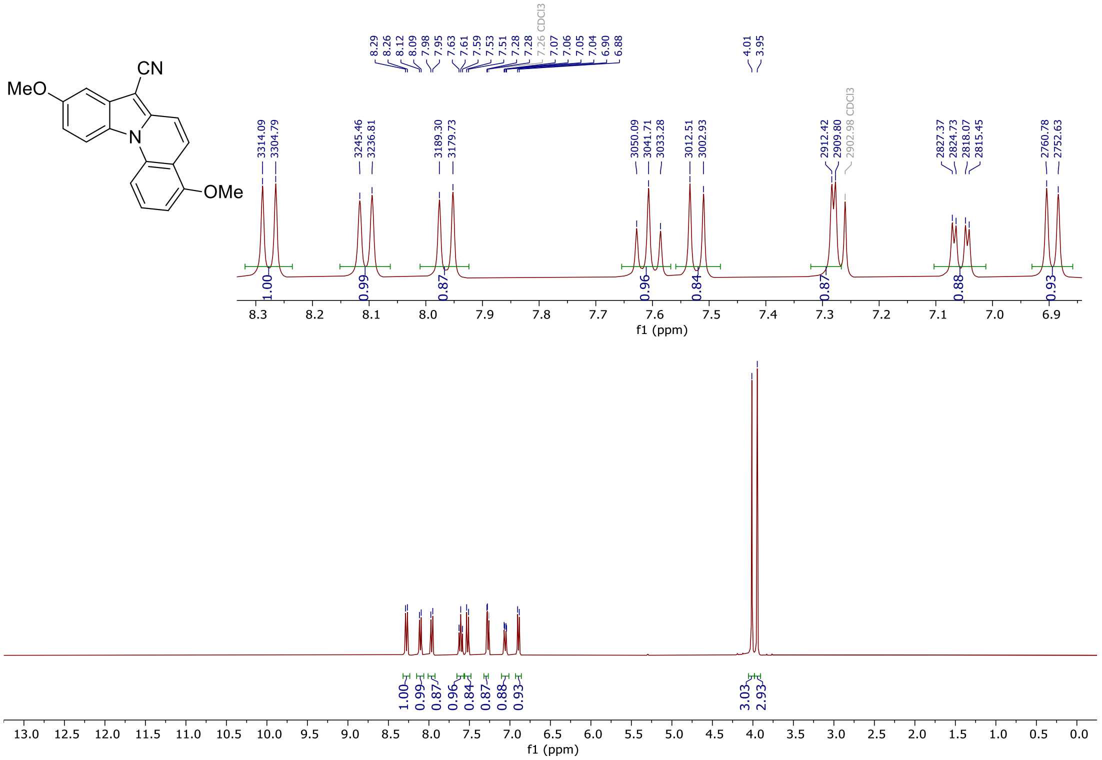

$^{13}C\{^{1}H\}$ NMR spectrum (100 MHz, $CDCl_3$) of compound **5cu**

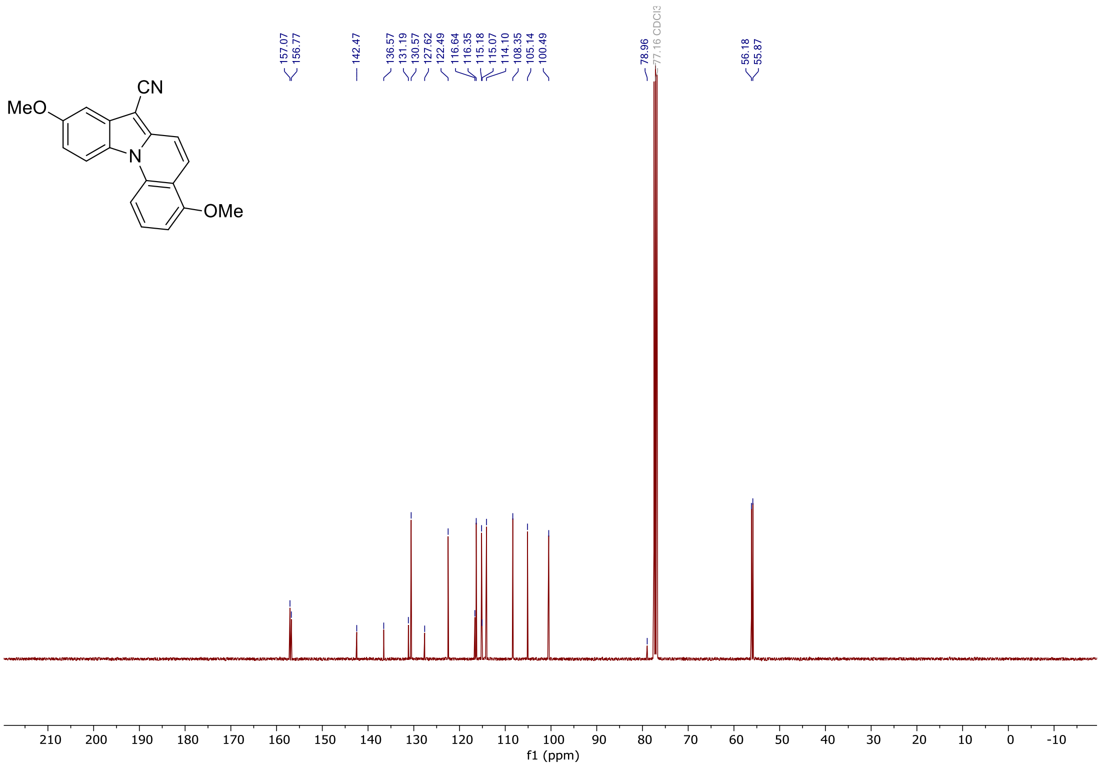

IR spectrum (neat) of compound **5cu**

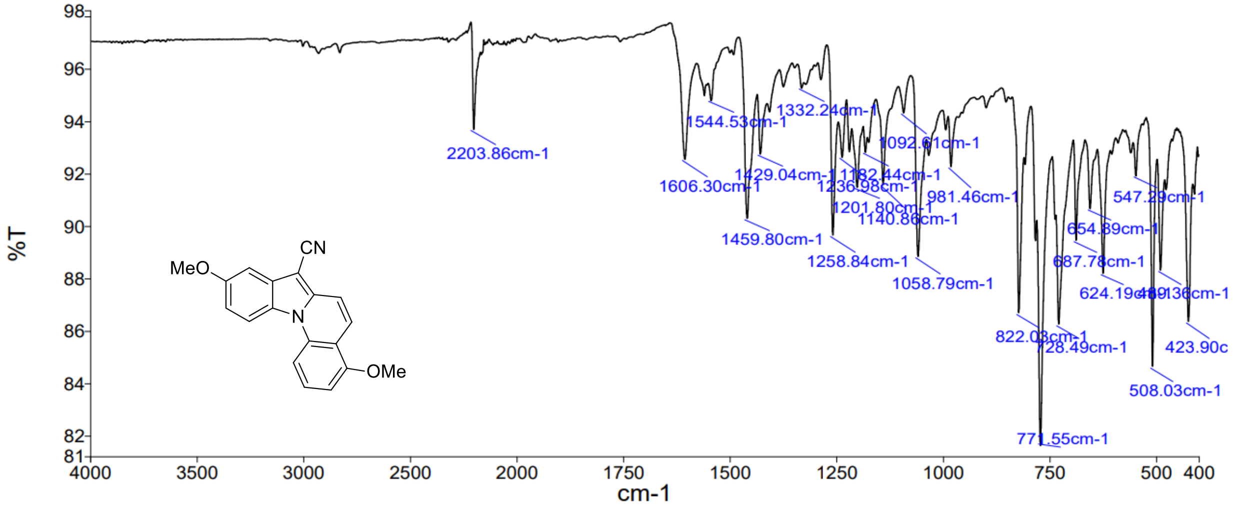

SAR analysis and primary screening data of indolo[1,2-*a*]quinoline derivatives for anticancer activity

Table S1. Primary screening data (% cell viability at 10 μM) of HCT-116, HT-29, and MCF-7 cancer cell lines categorized by the substituent position on C5 of the indole ring.

Compound	R^1	R^2	% Cancer cell viability			
			HCT-116	HT-29	MCF-7	Mean
**5aa**	Me	-		43.56		52.63
**5ab**	Me	1-F	45.86	48.46	75.61	
**5ac**	Me	2-F	24.68	20.48	33.47	
**5ae**	Me	4-F	91.06	39.77	40.66	
**5af**	Me	1-Cl	81.88	42.36	77.84	
**5ag**	Me	2-Cl	99.73	43.16	66.19	
**5ah**	Me	3-Cl	26.02	25.48	36.94	
**5ai**	Me	4-Cl	82.22	71.44	53.55	
**5aj**	Me	1-Br	58.54	64.54	73.63	
**5ak**	Me	2-Br	51.7	61.55	66.44	
**5al**	Me	3-Br	27.02	24.78	39.17	
**5am**	Me	4-Br	65.04	27.78	78.34	
**5an**	Me	3-I	29.18	28.57	42.39	
**5ao**	Me	1-Me	100	77.13	82.06	
**5ap**	Me	2-Me	31.52	35.17	36.44	
**5aq**	Me	3-Me	52.53	51.55	87.51	
**5ar**	Me	1-OMe	60.2	51.36	76.85	
**5as**	Me	2-OMe	55.87	65.74	59.50	
**5at**	Me	3-OMe	19.51	28.87	45.86	
**5au**	Me	4-OMe	30.85	31.97	62.97	
**5ba**	H	-		26.48		42.50
**5bb**	H	1-F	98.39	69.94	61.98	
**5bc**	H	2-F	19.01	**16.79**	32.23	
**5bd**	H	3-F	30.19	**16.09**	30.99	
**5be**	H	4-F	56.7	28.77	37.68	
**5bf**	H	1-Cl	39.52	55.95	77.10	
**5bg**	H	2-Cl	24.68	31.67	25.29	
**5bh**	H	3-Cl	25.35	32.57	**13.39**	
**5bi**	H	4-Cl	100	25.88	28.51	
**5bk**	H	2-Br	13.34	**15.69**	**12.89**	
**5bl**	H	3-Br	22.85	20.18	**11.40**	
**5bm**	H	4-Br	62.54	27.98	24.05	
**5bn**	H	3-I	75.88	55.45	42.14	
**5bo**	H	1-Me	86.22	65.84	67.93	
**5bp**	H	2-Me	26.52	26.38	37.43	
**5bq**	H	3-Me	35.19	34.97	58.75	
**5br**	H	1-OMe	100	60.85	57.02	
**5bs**	H	2-OMe	28.35	20.88	59.50	
**5bt**	H	3-OMe	56.87	41.76	64.70	
**5bu**	H	4-OMe	39.52	45.76	60.74	

Table S1. Primary screening data (% cell viability at 10 μM) of HCT-116, HT-29, and MCF-7 cancer cell lines categorized by the substituent position on C5 of the indole ring (continued).

Compound	R^1	R^2	% Cancer cell viability			
			HCT-116	HT-29	MCF-7	Mean
**5ca**	OMe	-		**13.69**		38.95
**5cb**	OMe	1-F	32.85	21.48	34.71	
**5cc**	OMe	2-F	21.51	21.78	51.81	
**5cd**	OMe	3-F	7.34	**9.89**	25.53	
**5ce**	OMe	4-F	32.69	21.38	52.80	
**5cf**	OMe	1-Cl	24.18	44.46	48.09	
**5cg**	OMe	2-Cl	33.85	22.28	62.97	
**5ch**	OMe	3-Cl	8.01	**3.00**	22.81	
**5ci**	OMe	4-Cl	58.2	33.37	78.09	
**5ck**	OMe	2-Br	27.52	**14.49**	53.05	
**5cm**	OMe	4-Br	100	61.85	62.22	
**5co**	OMe	1-Me	74.21	51.85	57.02	
**5cq**	OMe	3-Me	48.86	44.66	70.41	
**5cr**	OMe	1-OMe	58.54	46.16	50.33	
**5cs**	OMe	2-OMe	30.69	**19.78**	36.19	
**5ct**	OMe	3-OMe	23.35	30.97	48.09	
**5cu**	OMe	4-OMe	31.35	30.77	49.33	

Table S2. Primary screening data (% cell viability at 10 μM) of HCT-116, HT-29, and MCF-7 cancer cell lines categorized by the substituent position on the outer aromatic D-ring.

Compound	R^1	R^2	% Cancer cell viability											
			Position 1			Position 2			Position 3			Position 4		
			HCT-116	HT-29	MCF-7	HCT-116	HT-29	MCF-7	HCT-116	HT-29	MCF-7	HCT-116	HT-29	MCF-7
**5ab**	Me	1-F	45.86	48.46	75.61									
**5ac**	Me	2-F				24.68	20.48	33.47						
**5ae**	Me	4-F										91.06	39.77	40.66
**5af**	Me	1-Cl	81.88	42.36	77.84									
**5ag**	Me	2-Cl				99.73	43.16	66.19						
**5ah**	Me	3-Cl							26.02	25.48	36.94			
**5ai**	Me	4-Cl										82.22	71.44	53.55
**5aj**	Me	1-Br	58.54	64.54	73.63									
**5ak**	Me	2-Br				51.70	61.55	66.44						
**5al**	Me	3-Br							27.02	24.78	39.17			
**5am**	Me	4-Br										65.04	27.78	78.34
**5an**	Me	3-I							29.18	28.57	42.39			
**5ao**	Me	1-Me	100.00	77.13	82.06									
**5ap**	Me	2-Me				31.52	35.17	36.44						
**5aq**	Me	3-Me							52.53	51.55	87.51			
**5ar**	Me	1-OMe	60.20	51.36	76.85									
**5as**	Me	2-OMe				55.87	65.74	59.50						
**5at**	Me	3-OMe							19.51	28.87	45.86			
**5au**	Me	4-OMe										30.85	31.97	62.97
**5bb**	H	1-F	98.39	69.94	61.98									
**5bc**	H	2-F				19.01	16.79	32.23						
**5bd**	H	3-F							30.19	16.09	30.99			
**5be**	H	4-F										56.70	28.77	37.68
**5bf**	H	1-Cl	39.52	55.95	77.10									
**5bg**	H	2-Cl				24.68	31.67	25.29						
**5bh**	H	3-Cl							25.35	32.57	13.39			
**5bi**	H	4-Cl										100.00	25.88	28.51
**5bk**	H	2-Br				13.34	15.69	12.89						
**5bl**	H	3-Br							22.85	20.18	11.40			
**5bm**	H	4-Br										62.54	27.98	24.05
**5bn**	H	3-I							75.88	55.45	42.14			
**5bo**	H	1-Me	86.22	65.84	67.93									
**5bp**	H	2-Me				26.52	26.38	37.43						

Table S2. Primary screening data (% cell viability at 10 μM) of HCT-116, HT-29, and MCF-7 cancer cell lines categorized by the substituent position on the outer aromatic D-ring (continued).

Compound	R^1	R^2	% Cancer cell viability											
			Position 1			Position 2			Position 3			Position 4		
			HCT-116	HT-29	MCF-7	HCT-116	HT-29	MCF-7	HCT-116	HT-29	MCF-7	HCT-116	HT-29	MCF-7
**5bq**	H	3-Me							35.19	34.97	58.75			
**5br**	H	1-OMe	100.00	60.85	57.02									
**5bs**	H	2-OMe				28.35	20.88	59.50						
**5bt**	H	3-OMe							56.87	41.76	64.70			
**5bu**	H	4-OMe										39.52	45.76	60.74
**5cb**	OMe	1-F	32.85	21.48	34.71									
**5cc**	OMe	2-F				21.51	21.78	51.81						
**5cd**	OMe	3-F							7.34	9.89	25.53			
**5ce**	OMe	4-F										32.69	21.38	52.80
**5cf**	OMe	1-Cl	24.18	44.46	48.09									
**5cg**	OMe	2-Cl				33.85	22.28	62.97						
**5ch**	OMe	3-Cl							8.01	3.00	22.81			
**5ci**	OMe	4-Cl										58.20	33.37	78.09
**5ck**	OMe	2-Br				27.52	14.49	53.05						
**5cm**	OMe	4-Br										100.00	61.85	62.22
**5co**	OMe	1-Me	74.21	51.85	57.02									
**5cq**	OMe	3-Me							48.86	44.66	70.41			
**5cr**	OMe	1-OMe	58.54	46.16	50.33									
**5cs**	OMe	2-OMe				30.69	19.78	36.19						
**5ct**	OMe	3-OMe							23.35	30.97	48.09			
**5cu**	OMe	4-OMe										31.35	30.77	49.33
Mean			66.18	53.88	64.63	34.93	29.70	45.24	32.54	29.92	42.67	62.51	37.23	52.41
			61.56			36.62			35.04			50.72		

Evaluation of IC_{50} values

Table S3. Half-maximal inhibitory concentration (IC_{50}) values of active compounds against HCT-116 cancer cells from independent biological experiments (N = 2, each n = 3), including mean, standard deviation (SD), and standard error (SE).

Entry	Compound	IC_{50} (μM)				
		N1	N2	mean	SD	SE
1	**5at**	3.13	6.12	4.62	2.12	1.50
2	**5bc**	4.76	5.14	4.95	0.27	0.19
3	**5bk**	3.13	4.15	3.64	0.72	0.51
4	**5cd**	4.61	2.41	3.51	1.55	1.10
5	**5ch**	3.13	1.19	2.16	1.37	0.97

Table S4. Chemical structures, IC_{50} values (mean ± SD), and representative concentration-response curves (% cell viability) of active compounds against HCT-116 cells.

Entry	Compound	IC_{50} (μM)	Representative % cell viability graph
1	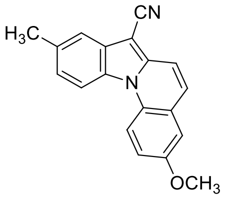<br>**5at**	4.62 ± 2.12	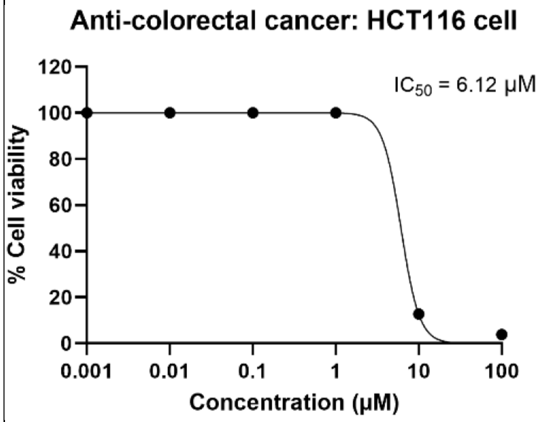
2	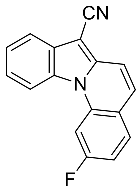<br>**5bc**	4.95 ± 0.27	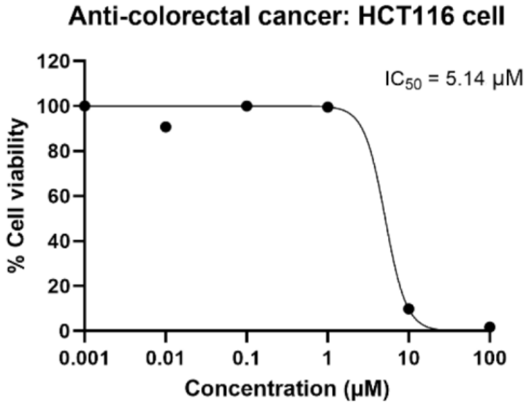

Table S4. Chemical structures, IC_{50} values (mean ± SD), and representative concentration-response curves (% cell viability) of active compounds against HCT-116 cells (continued).

Entry	Compound	IC_{50} (μM)	Representative % cell viability graph
3	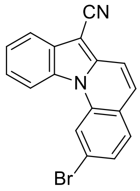 **5bk**	3.64 ± 0.72	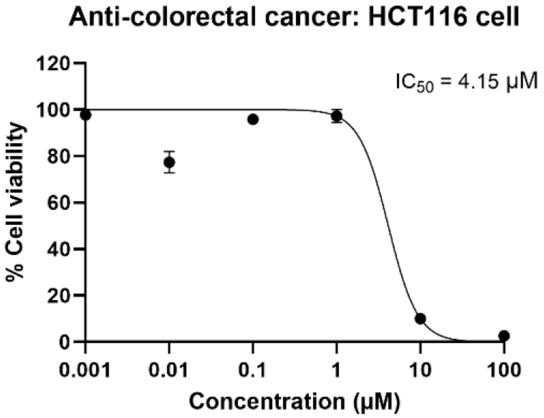
4	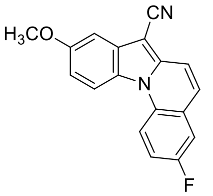 **5cd**	3.51 ± 1.55	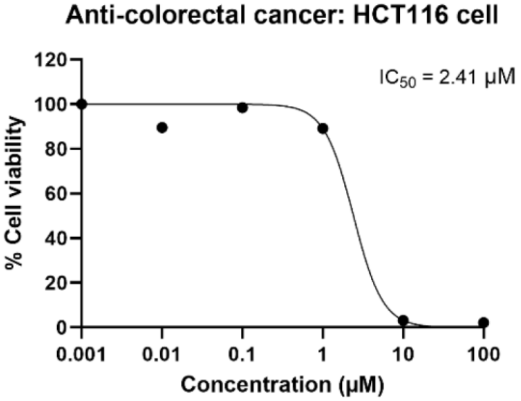
5	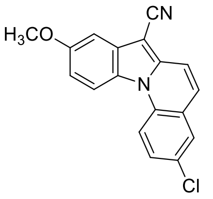 **5ch**	2.16 ± 1.37	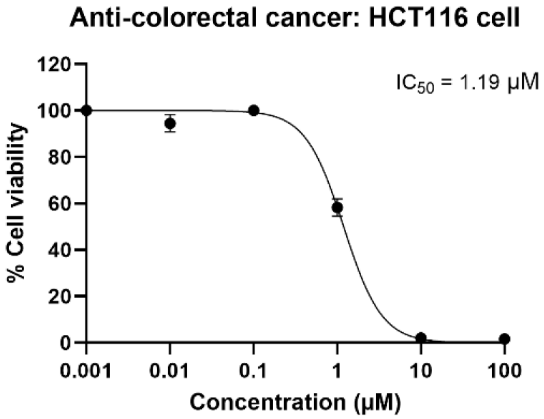

Table S5. Half-maximal inhibitory concentration (IC_{50}) values of active compounds against HCT-29 cancer cells from independent biological experiments (N = 2, each n = 3), including mean, standard deviation (SD), and standard error (SE).

Entry	Compound	IC_{50} (μM)				
		N1	N2	mean	SD	SE
1	**5bc**	3.22	4.63	3.93	1.00	0.71
2	**5bd**	3.13	5.65	4.39	1.79	1.26
3	**5bk**	6.59	3.99	5.29	1.83	1.30
4	**5ca**	9.30	7.05	8.18	1.59	1.12
5	**5cd**	7.53	3.35	5.44	2.96	2.09
6	**5ch**	3.13	1.67	2.40	1.03	0.73
7	**5ck**	3.48	4.77	4.13	0.91	0.64
8	**5cs**	3.13	1.41	2.27	1.21	0.86

Table S6. Chemical structures, IC_{50} values (mean ± SD), and representative concentration-response curves (% cell viability) of active compounds against HCT-29 cells.

Entry	Compound	IC_{50} (μM)	Representative % cell viability graph
1	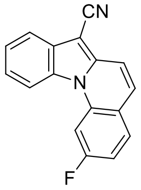 **5bc**	3.93 ± 1.00	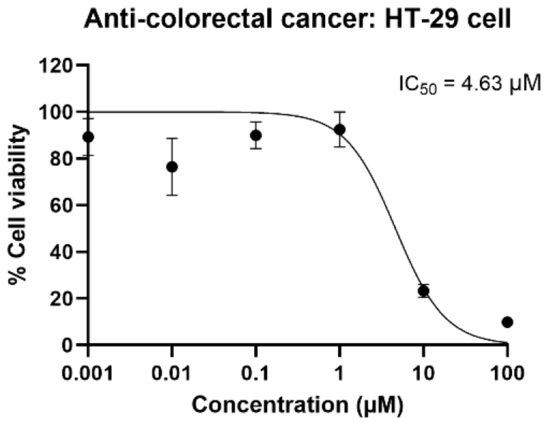
2	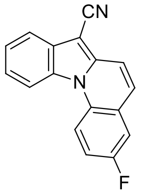 **5bd**	4.39 ± 1.79	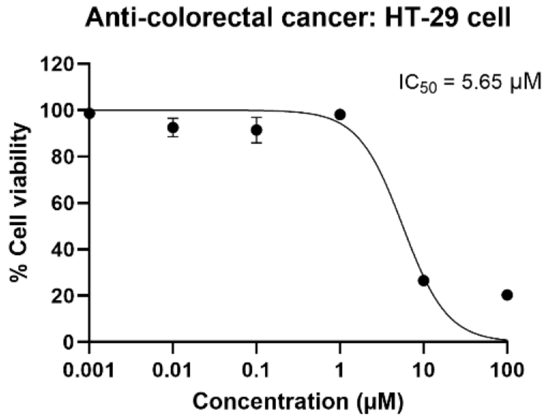

Table S6. Chemical structures, IC_{50} values (mean ± SD), and representative concentration-response curves (% cell viability) of active compounds against HCT-29 cells (continued).

Entry	Compound	IC_{50} (μM)	Representative % cell viability graph
3	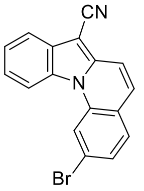<br>**5bk**	5.29 ± 1.83	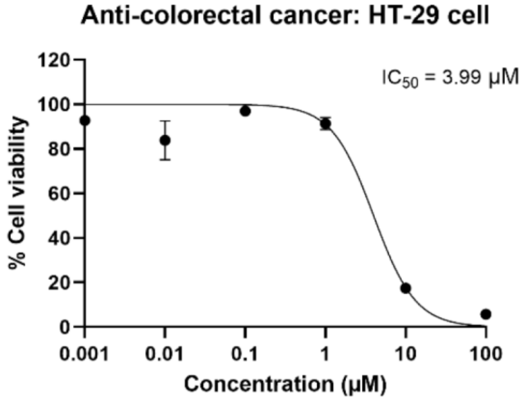
4	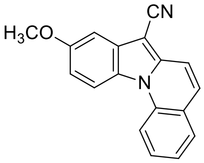<br>**5ca**	8.18 ± 1.59	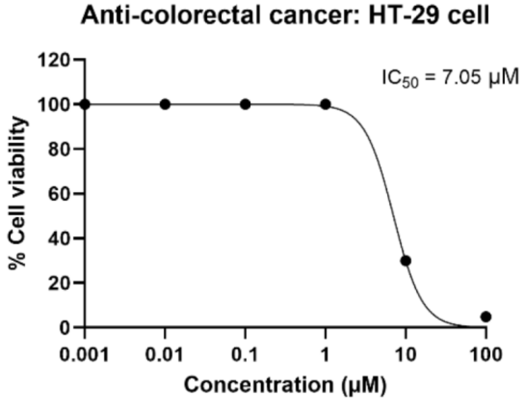
5	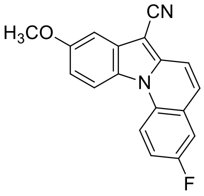<br>**5cd**	5.44 ± 2.96	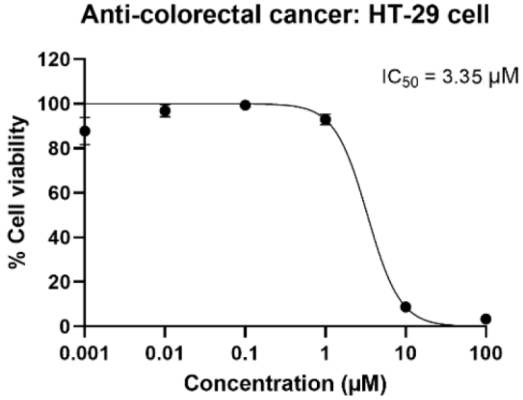

Table S6. Chemical structures, IC_{50} values (mean ± SD), and representative concentration-response curves (% cell viability) of active compounds against HCT-29 cells (continued).

Entry	Compound	IC_{50} (μM)	Representative % cell viability graph
6	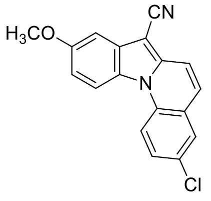<br>**5ch**	2.40 ± 1.03	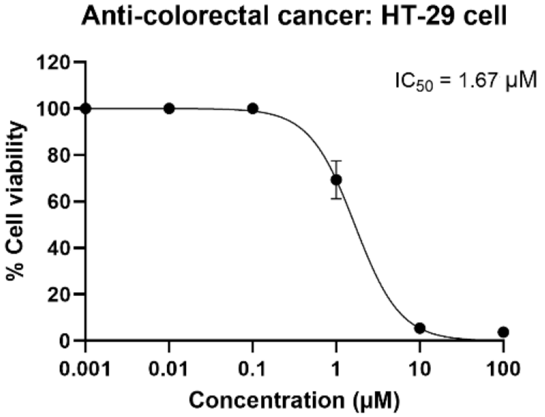
7	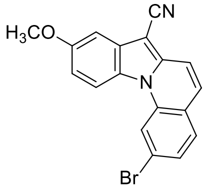<br>**5ck**	4.13 ± 0.91	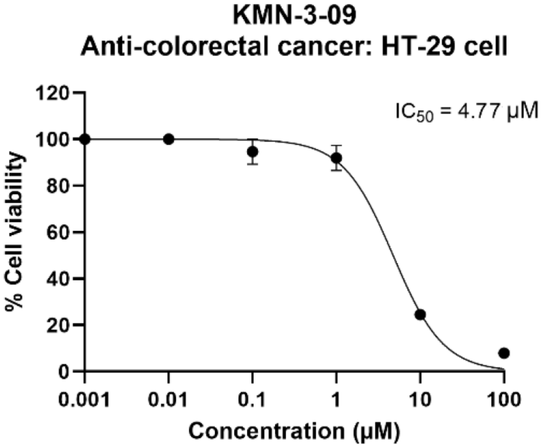
8	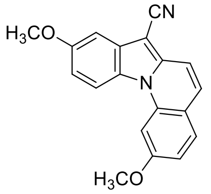<br>**5cs**	2.27 ± 1.21	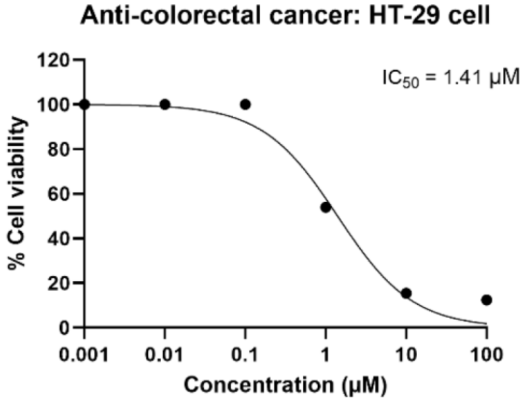

Table S7. Half-maximal inhibitory concentration (IC_{50}) values of active compounds against MCF-7 cancer cells from independent biological experiments (N = 2, each n = 3), including mean, standard deviation (SD), and standard error (SE).

Entry	Compound	IC_{50} (μM)				
		N1	N2	mean	SD	SE
1	**5bh**	4.53	6.82	5.68	1.62	1.15
2	**5bk**	6.17	5.95	6.06	0.16	0.11
3	**5bl**	3.835	3.835	3.835	0.000	0.000

Table S8. Chemical structures, IC_{50} values (mean ± SD), and representative concentration-response curves (% cell viability) of active compounds against MCF-7 cells.

Entry	Compound	IC_{50} (μM)	Representative % cell viability graph
1	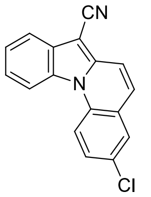 **5bh**	5.68 ± 1.62	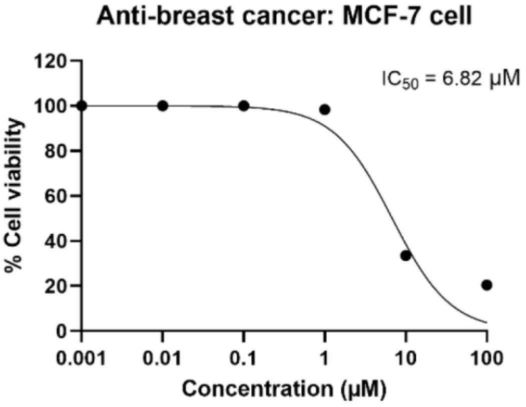
2	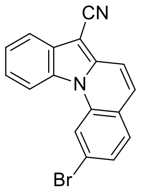 **5bk**	6.06 ± 0.16	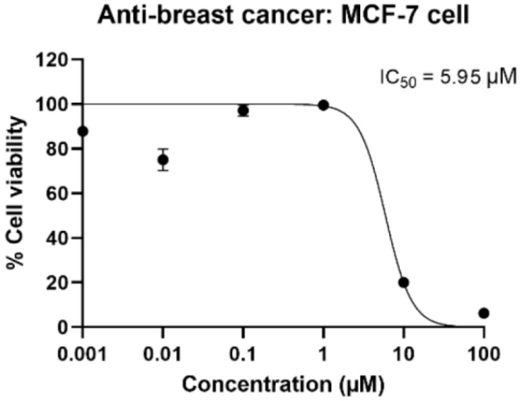

Table S8. Chemical structures, IC_{50} values (mean ± SD), and representative concentration-response curves (% cell viability) of active compounds against MCF-7 cells (continued).

Entry	Compound	IC_{50} (μM)	Representative % cell viability graph
3	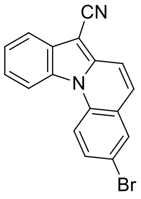 **5bl**	3.84 ± 0.00	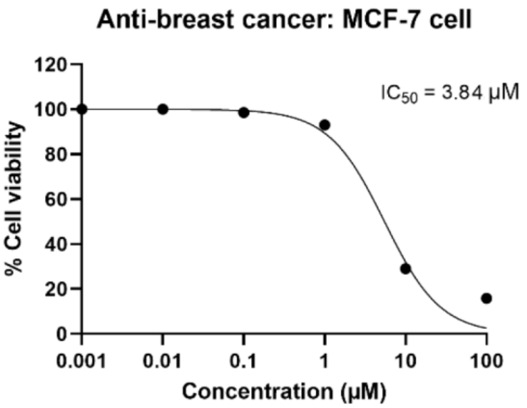

Molecular docking results and 2D/3D interaction poses

Table S9. Molecular docking results of the native ligand, compounds **5a**, **5at**, **5bc**, **5bk**, **5cd**, **5ch**, and doxorubicin within the active site of cyclin-dependent kinase 2 (CDK2) (PDB ID: 1DI8)[a]

Ligand	Binding energy (kcal/mol)	RMSD	Interaction			Others
			H-Bond		Hydrophobic	
			Number	Residue		
Native ligand (DTQ)	−8.03	1.43	6	HIS:84, LEU:83, GLU:81, PHE:80, ASP:86, GLN:131	ILE:10, PHE:82, LEU:134, ALA:31, VAL:64, VAL:18, LYS:33, ALA:144, PHE:80	Electrostatic interaction (ASP:145)
**5a**	−8.67		1	ASP:145	VAL:18, ALA:144, ILE:10, ALA:31, VAL:64, LEU:134	-
**5at**	−8.58		2	ASP:145, LEU:83	ALA:144, LEU:134, ALA:31, PHE:82, VAL:18, VAL:64	-
**5bc**	−8.06		1	ASP:145	ALA:144, LEU:134, VAL:64, ALA:31, VAL:18	-
**5bk**	−8.79		1	ASP:145	VAL:64, LEU:134, ALA:31, PHE:82, ILE:10, ALA:144	-
**5cd**	−8.33		4	LYS:33, ASP:145, LEU:83, ASN:132	VAL:18, ILE:10. PHE:82, ALA:31, PHE:80, LEU:134, VAL:64, ALA:144	Halogen (fluorine) interaction (LEU:83)
**5ch**	−8.39		1	LEU:83	ILE:10, VAL:18, PHE:80, ALA:144, LEU:148, LYS:33, ALA:31, VAL:64, LEU:134	-
Doxorubicin	−8.83		4	LEU:83, ASP:145, ASN:132, GLU:12	LYS:89, LEU:134, PHE:82, ILE:10, VAL:18, ALA:31	-

[a] Grid box: 40 points (spacing 0.375 Å); grid center coordinates (x, y, z) = −8.615, −49.545, 12.689

DTQ (Native ligand)

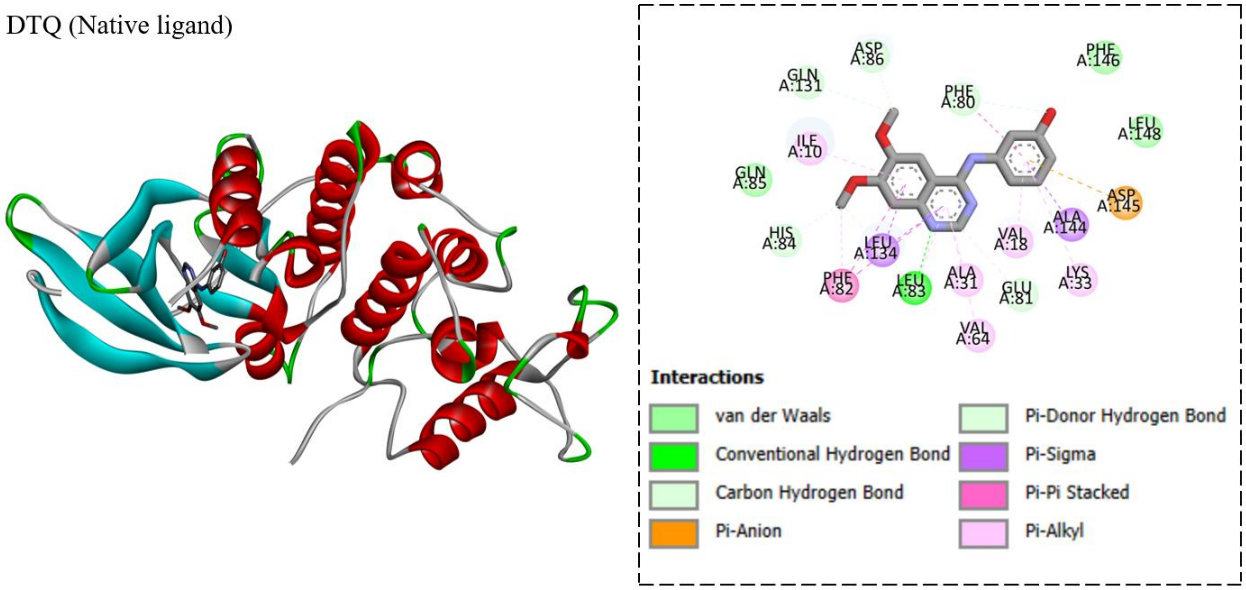

Figure S1. Predicted 3D binding interactions of DTQ (native ligand) with CDK2 (PDB ID: 1DI8), showing 3D docking poses (left) and 2D ligand-residue interaction diagrams (right).

5at

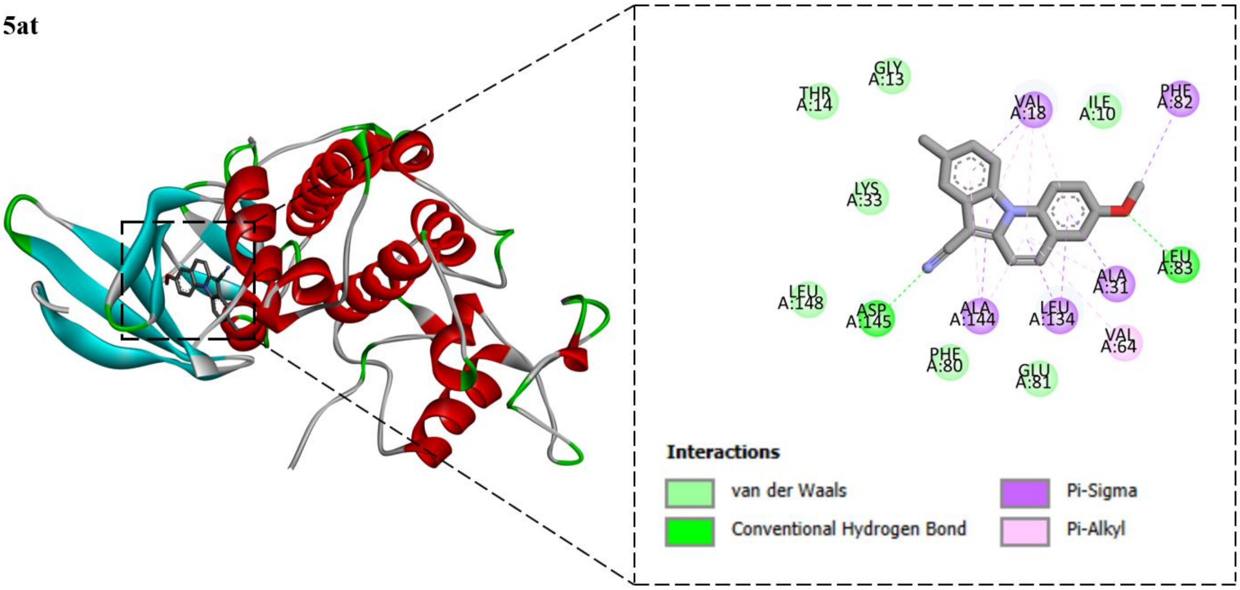

Figure S2. Predicted 3D binding interactions of compound **5at** with CDK2 (PDB ID: 1DI8), showing 3D docking poses (left) and 2D ligand-residue interaction diagrams (right).

5bc

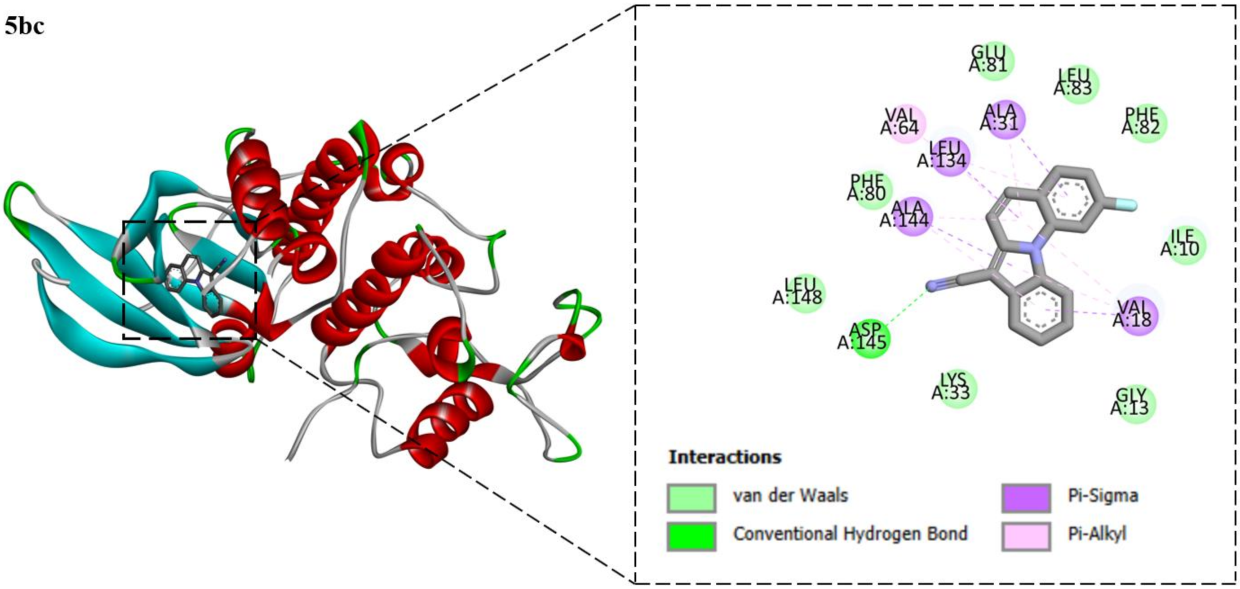

Figure S3. Predicted 3D binding interactions of compound **5bc** with CDK2 (PDB ID: 1DI8), showing 3D docking poses (left) and 2D ligand-residue interaction diagrams (right).

5cd

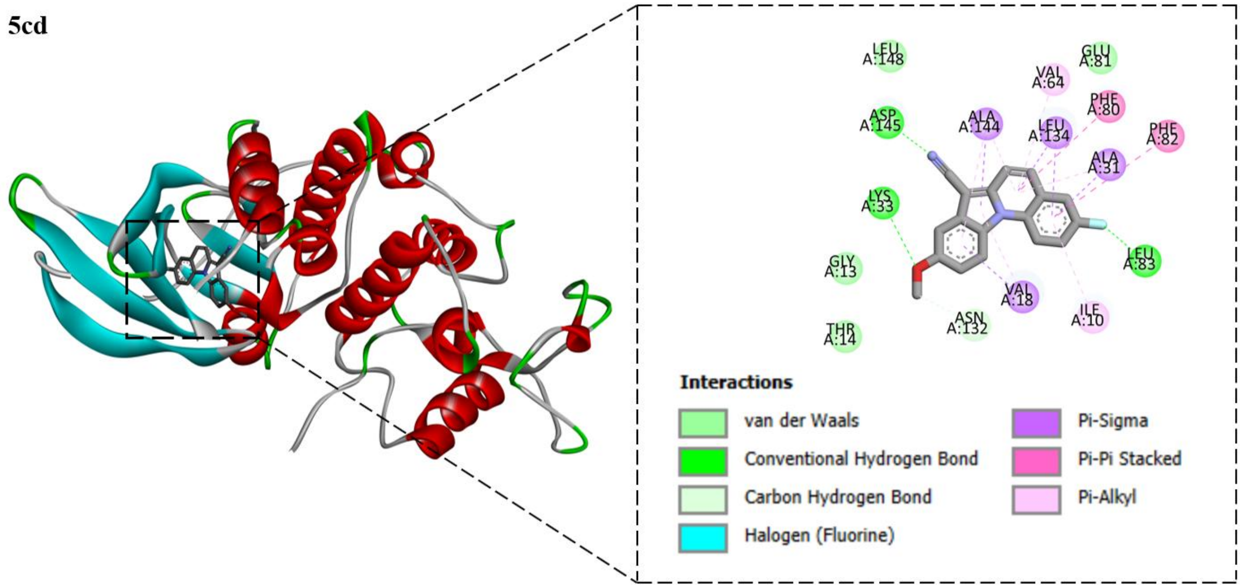

Figure S4. Predicted 3D binding interactions of compound **5cd** with CDK2 (PDB ID: 1DI8), showing 3D docking poses (left) and 2D ligand-residue interaction diagrams (right).

5ch

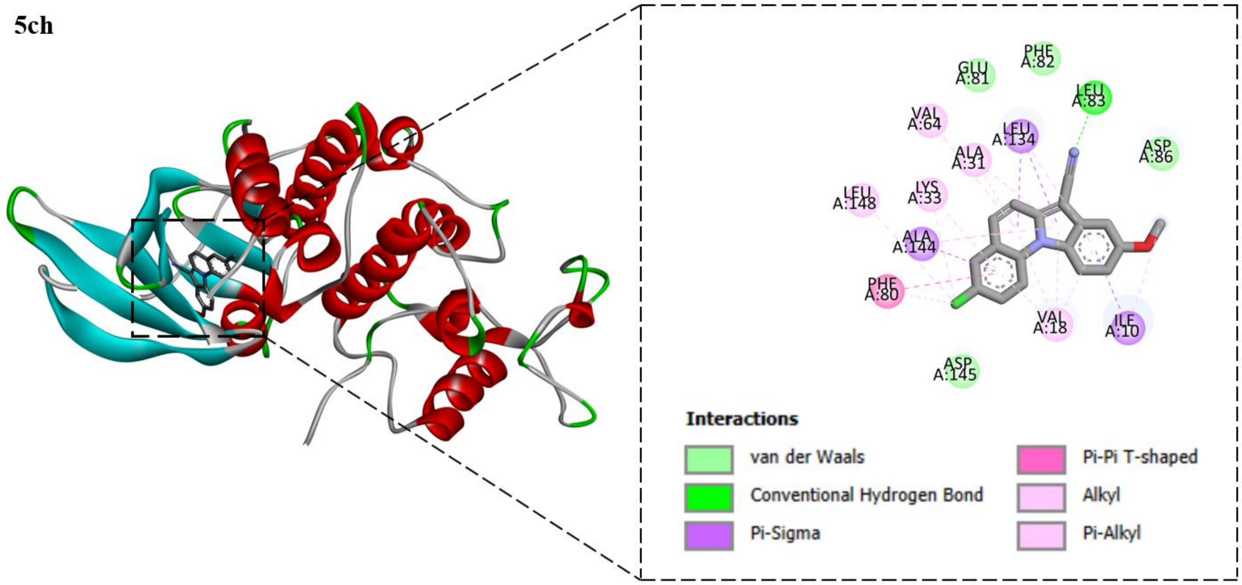

Figure S5. Predicted 3D binding interactions of compound **5ch** with CDK2 (PDB ID: 1DI8), showing 3D docking poses (left) and 2D ligand-residue interaction diagrams (right).

Table S10. Molecular docking results of native ligand, compounds **5bc**, **5bd**, **5bk**, **5ca**, **5cd**, **5ch**, **5ck**, **5cs**, and doxorubicin within the active site of cyclin-dependent kinase 6 (CDK6) (PDB ID: 5L2I)[a]

Ligand	Binding energy (kcal/mol)	RMSD	Interaction			
			H-Bond		Hydrophobic	Others
			Number	Residue		
Native ligand (LQQ)	−11.04	0.83	5	GLU:99, VAL:101, HIS:100, ASP:104, ASP:163	PHE:98, LEU:152, VAL:77, ALA:41, ILE:19, VAL:27, ALA:162	-
**5bc**	−7.77		2	VAL:101, HIS:100	LEU:152, ILE:19, VAL:27	Halogen (fluorine) interaction (GLN:149)
**5bd**	−7.85		2	VAL:101, HIS:101	ILE:19, LEU:152, ALA:41, PHE:98, VAL:77, ALA:162	Halogen (fluorine) interaction (GLN:149)
**5bk**	−9.49		1	LYS:43	VAL:27, ILE:19, LEU:152, ALA:41, VAL:77, PHE:98	-
**5ca**	−7.81		1	ASP:163, VAL:101, HIS:100	VAL:77, PHE:98, ALA:162, ILE:19, LEU:152	-
**5cd**	−7.73		2	VAL:101, ASP:163	ILE:19, LEU:152, VAL:77, PHE:98 ALA:162	Electrostatic interaction (ASP:104), Halogen (fluorine) interaction (ASP:104)
**5ch**	−8.04		2	VAL:101, ASP:163	ILE:19, LEU:152, VAL:77, PHE:98, ALA:162	Electrostatic interaction (ASP:104)
**5ck**	−8.56		-	-	ALA:162, HIS:100, ILE:19, VAL:77, ALA:41, VAL:101, LEU:152	-
**5cs**	−8.48		2	ASP:163, ASP:104	ALA:162, PHE:98, VAL:77, VAL:101, ALA:41, LEU:152, VAL:27, ILE:19	-
Doxorubicin	−10.96		6	ASP:163, GLN:149, ASP:104, THR:107, VAL:101, HIS:100	ALA:162, VAL:77, ALA:41, LEU:152, ILE:19	-

[a] Grid box: 40 points (spacing 0.375 Å); grid center coordinates (x, y, z) = 13.654, 27.991, 9.706

LQQ (Native ligand)

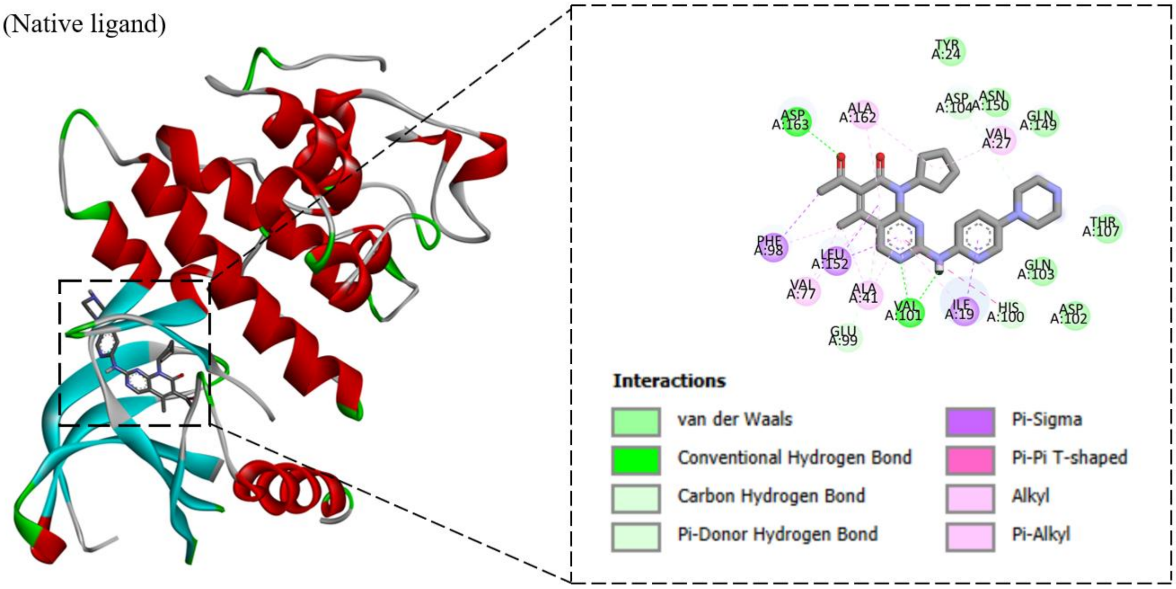

Figure S6. Predicted 3D binding interactions of LQQ (native ligand) with CDK6 (PDB ID: 5L2I), showing 3D docking poses (left) and 2D ligand-residue interaction diagrams (right).

5bc

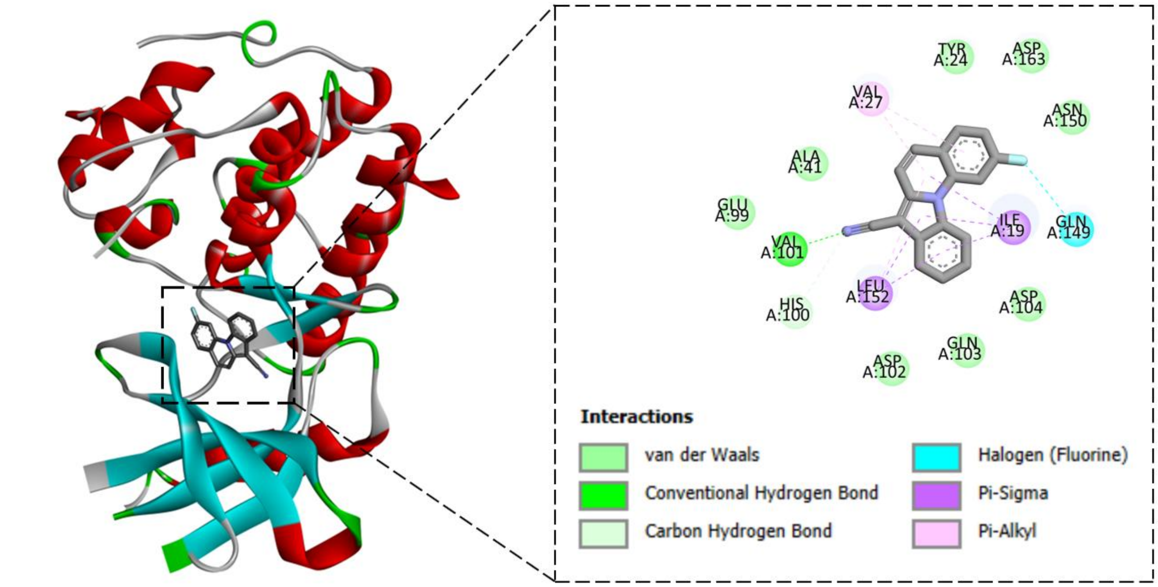

Figure S7. Predicted 3D binding interactions of compound **5bc** with CDK6 (PDB ID: 5L2I), showing 3D docking poses (left) and 2D ligand-residue interaction diagrams (right).

5bd

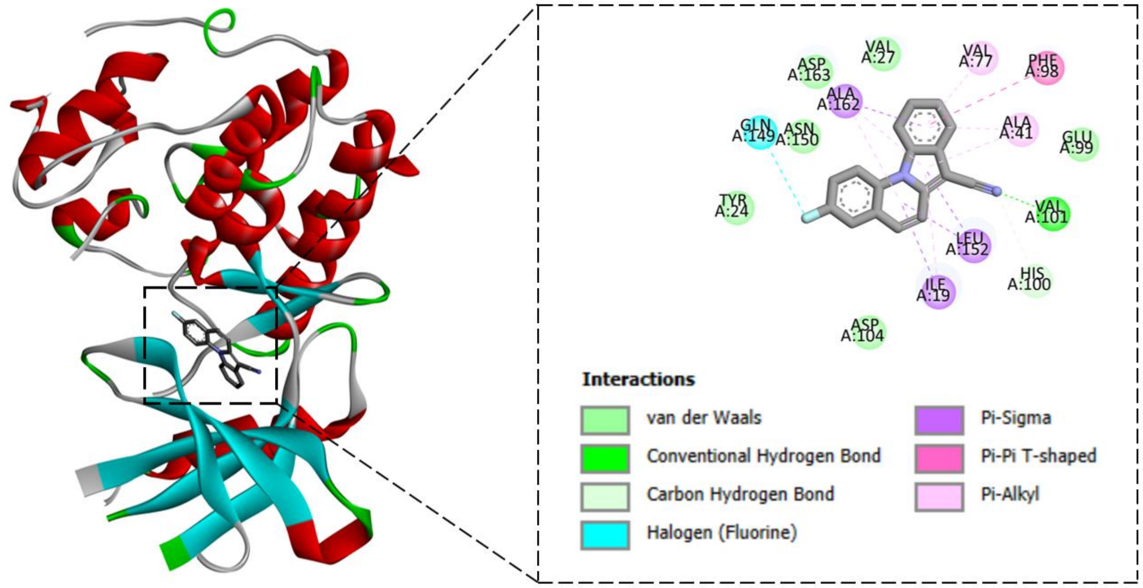

Figure S8. Predicted 3D binding interactions of compound **5bd** with CDK6 (PDB ID: 5L2I), showing 3D docking poses (left) and 2D ligand-residue interaction diagrams (right).

5ca

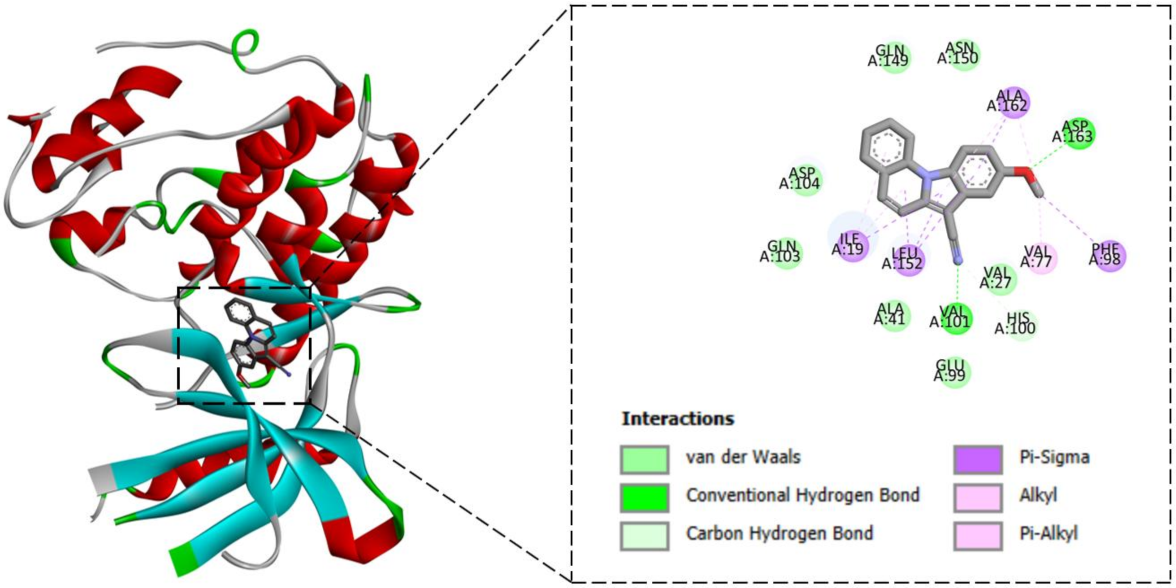

Figure S9. Predicted 3D binding interactions of compound **5ca** with CDK6 (PDB ID: 5L2I), showing 3D docking poses (left) and 2D ligand-residue interaction diagrams (right).

5cd

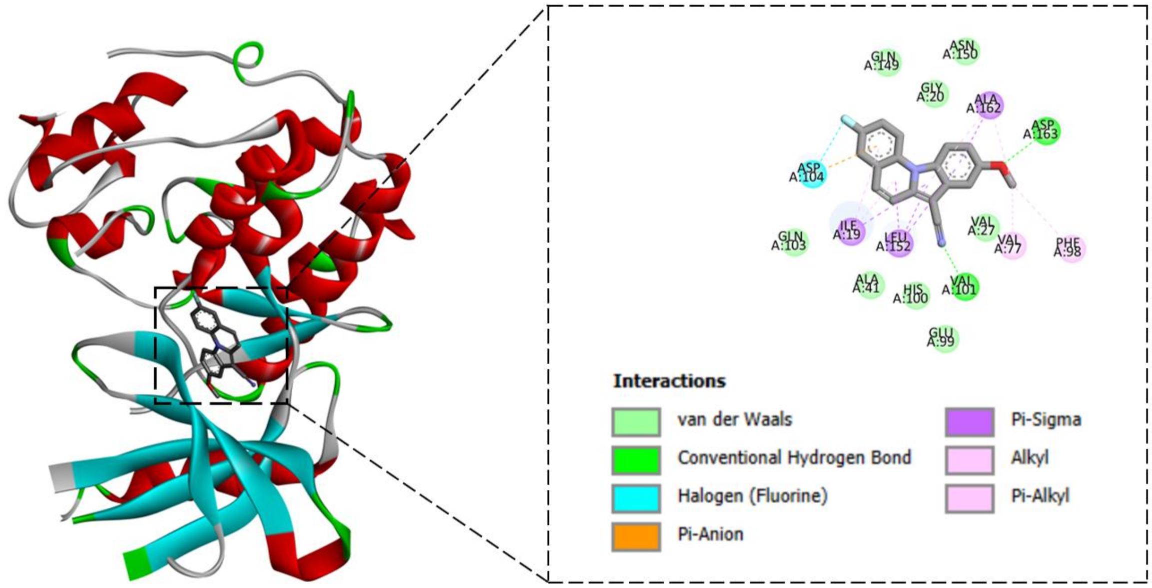

Figure S10. Predicted 3D binding interactions of compound **5cd** with CDK6 (PDB ID: 5L2I), showing 3D docking poses (left) and 2D ligand-residue interaction diagrams (right).

5ch

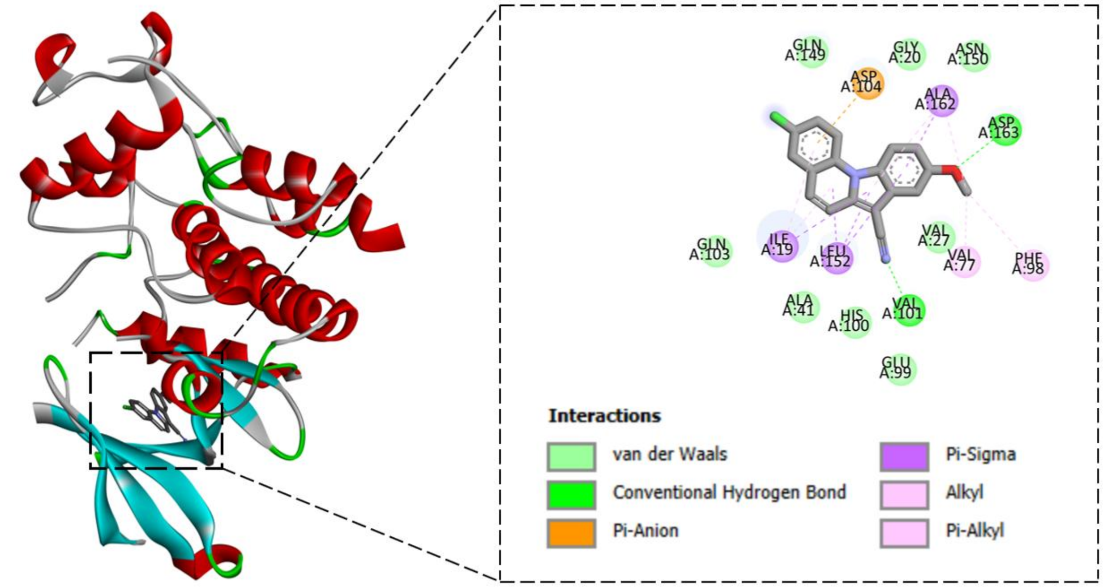

Figure S11. Predicted 3D binding interactions of compound **5ch** with CDK6 (PDB ID: 5L2I), showing 3D docking poses (left) and 2D ligand-residue interaction diagrams (right).

5ck

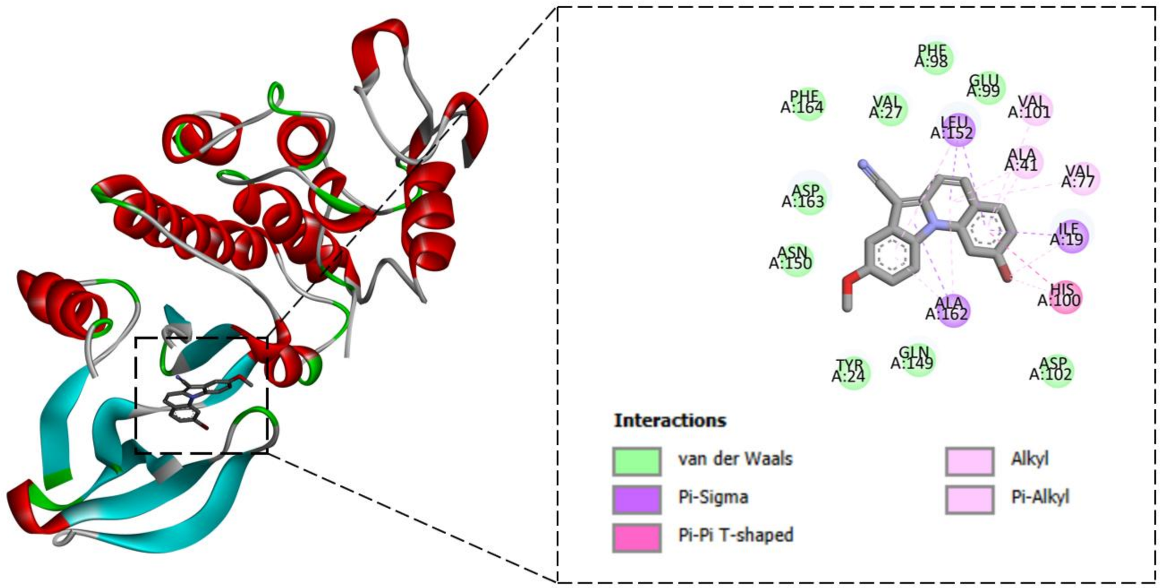

Figure S12. Predicted 3D binding interactions of compound **5ck** with CDK6 (PDB ID: 5L2I), showing 3D docking poses (left) and 2D ligand-residue interaction diagrams (right).

5cs

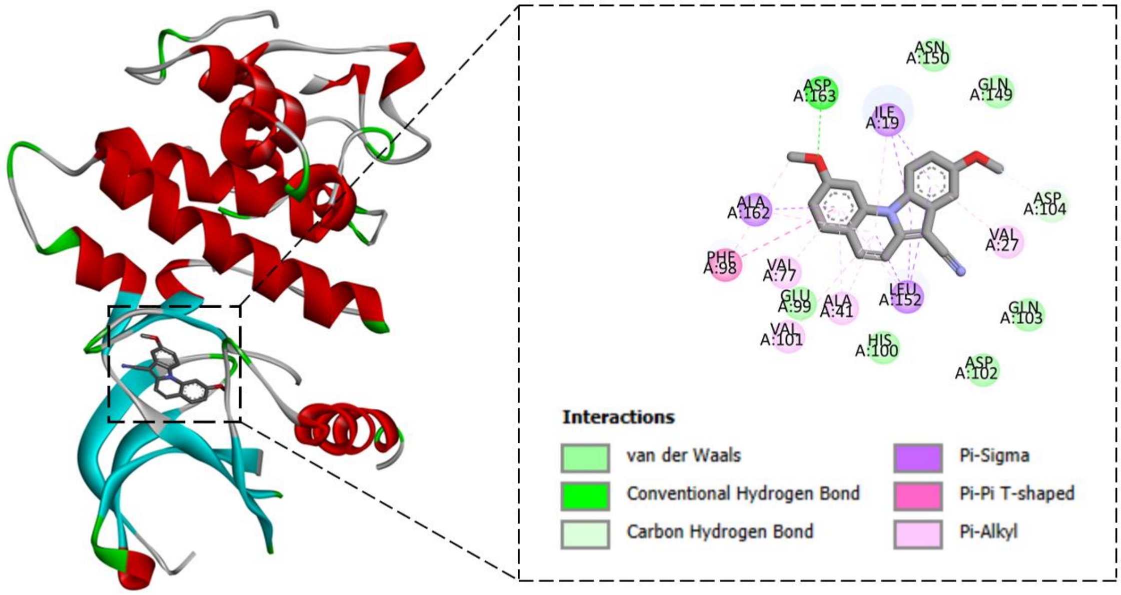

Figure S13. Predicted 3D binding interactions of compound **5cs** with CDK6 (PDB ID: 5L2I), showing 3D docking poses (left) and 2D ligand-residue interaction diagrams (right).

Table S11. Molecular docking results of native ligand, compounds **5bh**, **5bk**, **5bl**, and doxorubicin within the active site of epidermal growth factor receptor (EGFR) kinase (PDB: 1M17)[a]

Ligand	Binding energy (kcal/mol)	RMSD	Interaction			
			H-Bond		Hydrophobic	Others
			Number	Residue		
Native ligand (AQ4)	-7.68	1.64	3	CYS:773, MET:769, GLN:767	LEU:820, ALA:719, LEU:768, LEU:764, LYS:721, LEU:694	Electrostatic interaction (LYS:721)
**5bh**	-8.40		1	ASP:831	LYS:721, ALA:719, VAL:702, LEU:694, LEU:820	Miscellaneous interaction (sulfur) (MET:742)
**5bk**	-8.02		3	ASP:831, LYS:721 THR:766,	LEU:820, VAL:702, ALA:719, LEU:768, LEU:694, LYS:721	-
**5bl**	-8.61		1	ASP:831	LEU:820, LYS:721, ALA:719, VAL:702, LEU:694	Miscellaneous interaction (sulfur) (MET:742)
Doxorubicin	-11.69		3	ASP:831, LYS:721, MET:769	LEU:820, VAL:702, ALA:719, LEU:694, LEU:768, ARG:817	-

[a] Grid box: 40 points (spacing 0.375 Å); grid center coordinates (x, y, z) = 22.000, 0.300, 52.800

AQ4 (Native ligand)

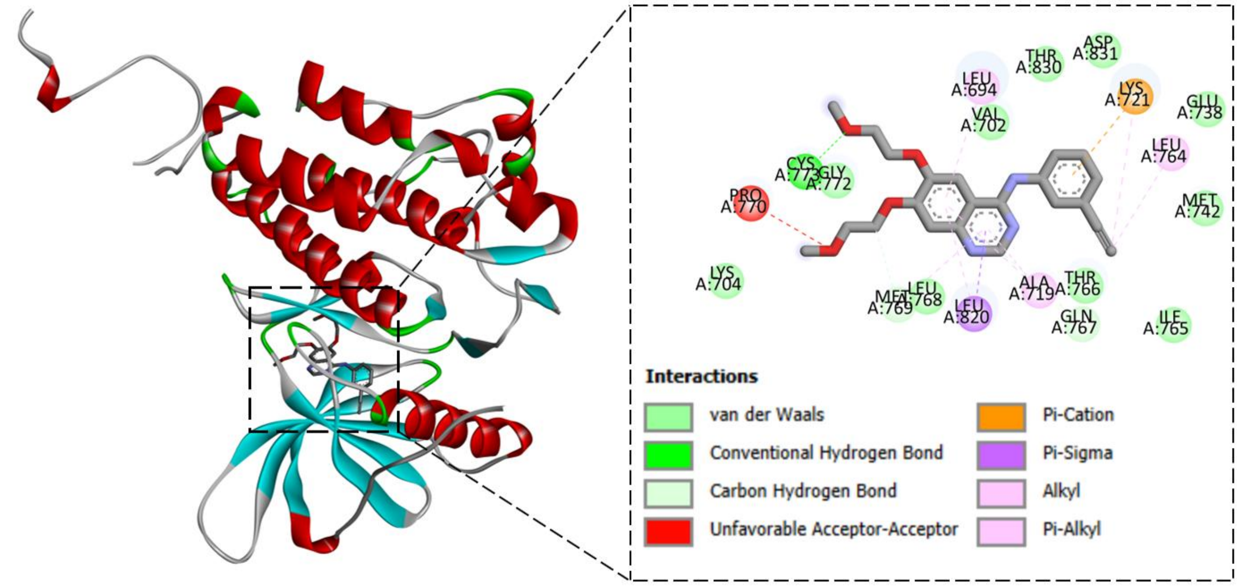

Figure S14. Predicted 3D binding interactions of AQ4 (native ligand) with EGER kinase (PDB: 1M17), showing 3D docking poses (left) and 2D ligand-residue interaction diagrams (right).

5bh

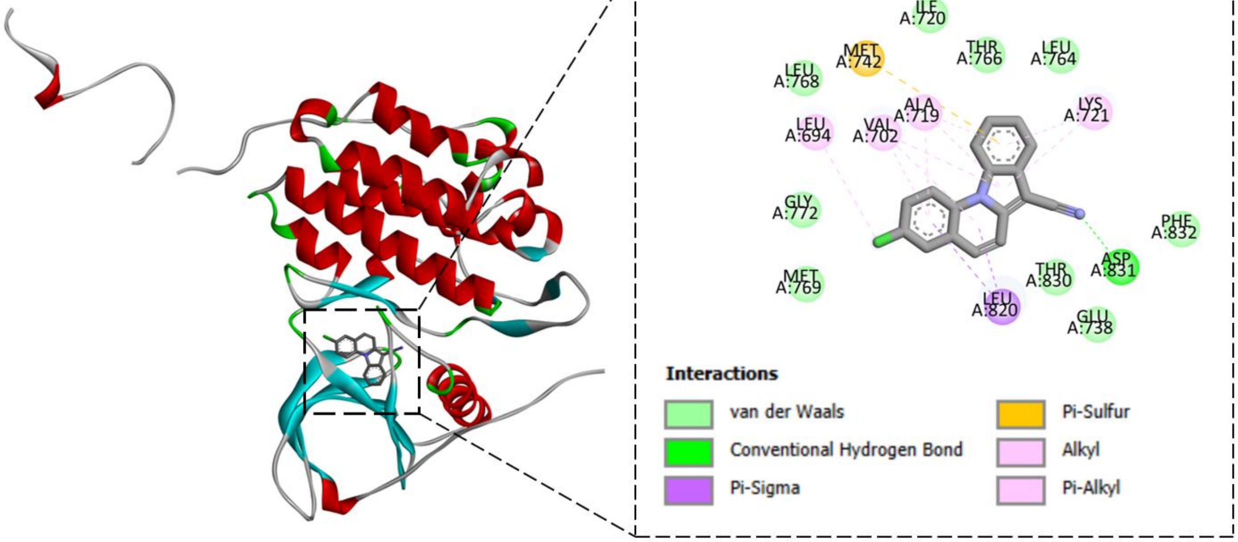

Figure S15. Predicted 3D binding interactions of compound **5bh** with EGER kinase (PDB: 1M17), showing 3D docking poses (left) and 2D ligand-residue interaction diagrams (right).

5bl

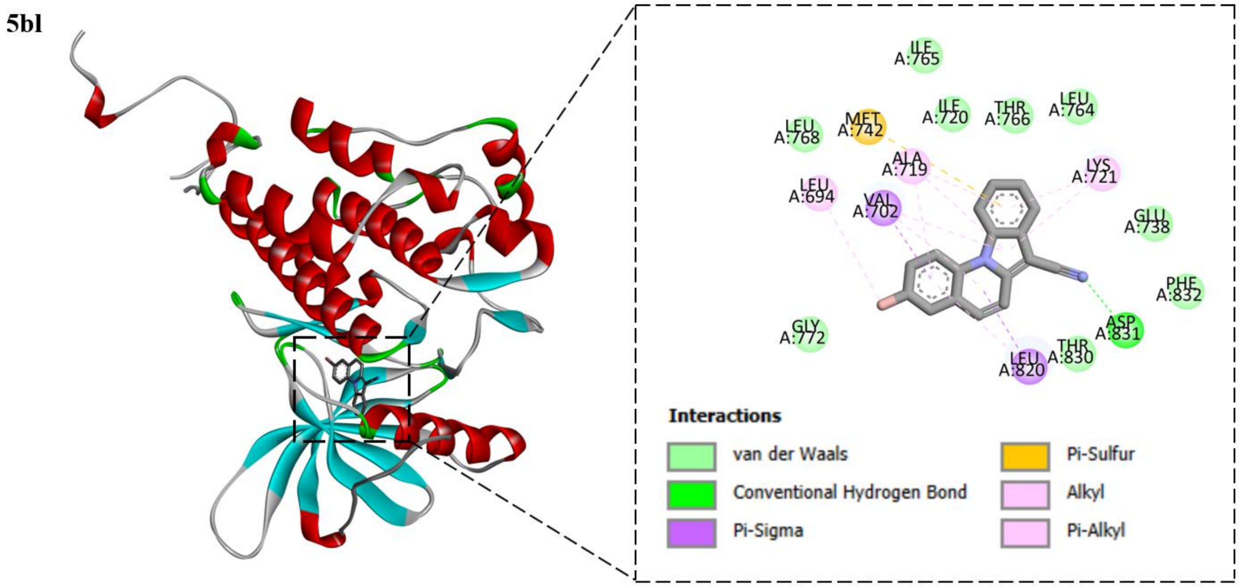

Figure S16. Predicted 3D binding interactions of compound **5bl** with EGER kinase (PDB: 1M17), showing 3D docking poses (left) and 2D ligand-residue interaction diagrams (right).
